# Synthesis and characterization of cortinarins – cryptic cyclic peptides from mushrooms of the genus *Cortinarius*

**DOI:** 10.1039/d6sc02205g

**Published:** 2026-06-23

**Authors:** Zhanyu He, Celine Janssen, Joana-Lysiane Schäfer, Agnes Mühlenweg, Simone Kosol, Rene Jarling, Andi Mainz, Bettina G. Keller, Guiyang Yao, Enno Klussmann, Roderich D. Süssmuth

**Affiliations:** a Institut für Chemie, Technische Universität Berlin Straße des 17. Juni 115 D-10623 Berlin Germany roderich.suessmuth@tu-berlin.de; b Max-Delbrück-Center for Molecular Medicine in the Helmholtz Association (MDC) Robert-Rössle Straße 10 D-13125 Berlin Germany enno.klussmann@mdc-berlin.de; c Greater Bay Area Institute of Precision Medicine (Guangzhou) Guangzhou 510000 PR China yaoguiyang@ipm-gba.org.cn; d Department of Biology, Chemistry, and Pharmacy, Freie Universität Berlin Königin-Luise Straße 28-30 D-14195 Berlin Germany; e MSB Medical School Berlin, Hochschule für Gesundheit und Medizin Rüdesheimer Straße 50 D-14197 Berlin Germany; f ZE Botanischer Garten und Botanisches Museum Berlin, Freie Universität Berlin Arnimallee 22 D-14195 Berlin Germany; g DZHK (German Centre for Cardiovascular Research), Partner Site Berlin Potsdamer Straße 58 D-10785 Berlin Germany

## Abstract

Cortinarins have been described as cyclic peptides from mushrooms of the genus *Cortinarius*, and brought into context with mushroom intoxication, particularly causing kidney damage. Herein we report the first total synthesis of the bicyclic peptides cortinarin A and B using iodine-mediated Trp–Cys cyclization to establish their characteristic tryptathionine bridge. Peptide atropisomers, termed ansamers, were observed and characterized by NMR spectroscopy and molecular dynamics simulations. Conformational analysis showed that cortinarins adopt β-turn structures. Furthermore, cortinarins induce similar effects like the peptide hormone arginine vasopressin on the localization of aquaporin-2 in renal principal cells, which regulates water and salt balance in the body, but with a different mode of action. This work thus provides synthetic access to the otherwise elusive cortinarins for biological evaluation and as potential lead compounds for the treatment of renal disorders.

## Abstract 2

First total synthesis of cortinarins A–C, cryptic cyclic peptides exhibiting non-classical atropisomerism. They mimic vasopressin-induced aquaporin-2 membrane localization through a distinct mode of action, offering leads for diabetes insipidus.

## Graphical abstract

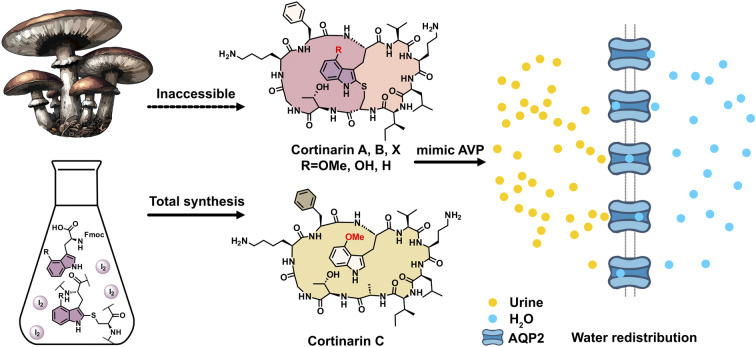

## Introduction

Mushroom poisoning, primarily caused by the accidental consumption of misidentified toxic wild species, represents a significant public health concern worldwide. Amongst others, mushrooms of the genera *Amanita*, *Cortinarius* and *Psilocybe*, account for the most notoriously toxic fungi.^1^ Structurally, the toxins are derived from all classes of natural products, *e.g.* peptides (amanitins, phalloidins),^2^ amino acid analogs (ibotenic acid),^3^ polyketides (anthraquinones)^4^ and terpenoids (illudins).^5^

**Fig. 1:**
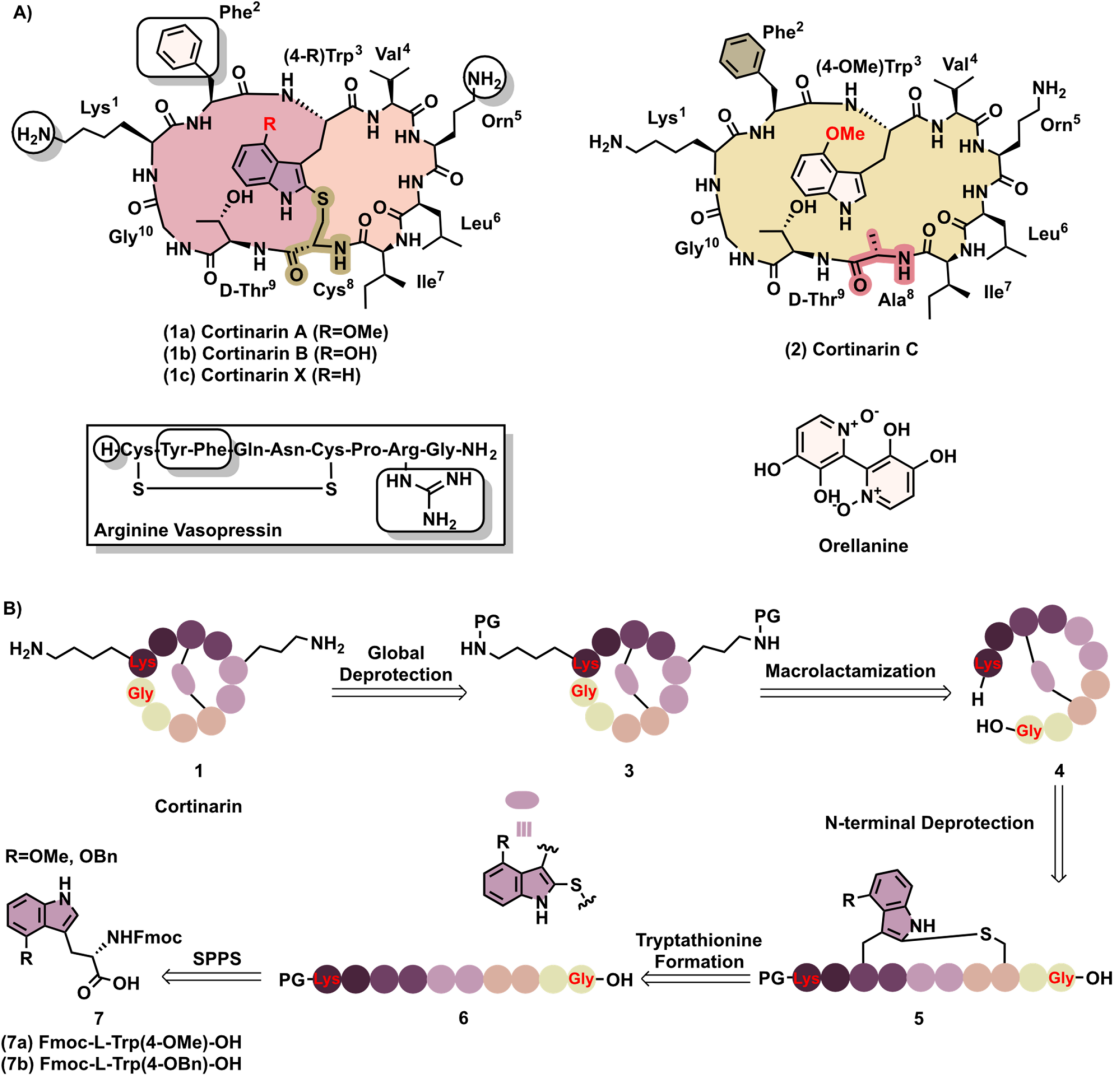
Cortinarins A–C and analog X: structures and retrosynthesis. (A) Natural cortinarins A–C, the simplified model analog cortinarin X, the nephrotoxin orellanine, and the peptide hormone arginine vasopressin (AVP). Structural similarities between cortinarins and AVP are highlighted; key differences among cortinarins A–X are color-coded. (B) Retrosynthetic route to the cortinarins (PG = protecting group).

The genus *Cortinarius* is globally distributed and represents one of the most diverse groups of agaric fungi.^6–10^ With more than 2000 documented species worldwide it exhibits a remarkable ecological adaptability across diverse habitats,^9^ which however correlates with significant toxicological risks. *Cortinarius* poisoning became tangible in the mid-20th century following a catastrophic poisoning event in Poland (1953–1957),^11^ which affected more than 135 individuals primarily linked to the ingestion of *Cortinarius orellanus* (fool's webcap). This incident spurred critical research into its nephrotoxic components. In 1965, a fluorescent bipyridine compound was identified as the principal toxin, later named orellanine (Fig. 1).^12^ Delayed symptom onset, characteristically 2–20 days post-ingestion frequently results in misdiagnosed cases progressing to irreversible renal damage. The clinical presentation typically evolves through sequential phases: initial gastrointestinal distress followed by oliguric acute kidney injury that may progress towards chronic renal insufficiency.^13–17^ The mechanism of action and toxicity was found to involve metal complexation, leading to reactive oxygen species (ROS) generation and redox cycling, which deplete cellular NADPH and cause DNA damage.^18^ Reverse transcription polymerase chain reaction (RT-PCR) of renal tissue showed downregulation of mRNAs coding for enzymes related to oxidative stress responses, *e.g.* glutathione peroxidase GPX3, and the upregulation of inflammatory cytokines, *e.g.* IL-1β, TGF-1β and TNF.^19^ The resulting synergistic effects lead to renal tubular epithelial cellular dysfunctions and apoptosis, which ultimately manifests in renal failure.^20–23^

In 1984, Tebbet and Caddy isolated three further major toxin components from the closely related species *Cortinarius speciosissimus*.^24^ These toxins were characterized as cyclic peptides and termed cortinarins (Fig. 1). The bicyclic peptides cortinarin A (CorA, 1a) and cortinarin B (CorB, 1b) which showed nephrotoxicity in animals, bear a tryptathionine bridge (Trp–Cys linkage), a characteristic structural moiety previously known from the death cap toxins phalloidin and amanitin.^2^ The third component, cortinarin C (CorC, 2), is a monocyclic peptide, where the bridge-forming Cys is replaced by Ala (Fig. 1). Initial bioassays suggested that CorA and CorB induce renal damage in mice through an unknown mechanism.^24,25^ Moreover, similar to arginine-vasopressin (AVP) they acted on the collecting ducts of the nephron resulting in water retention.

**Scheme 1:**
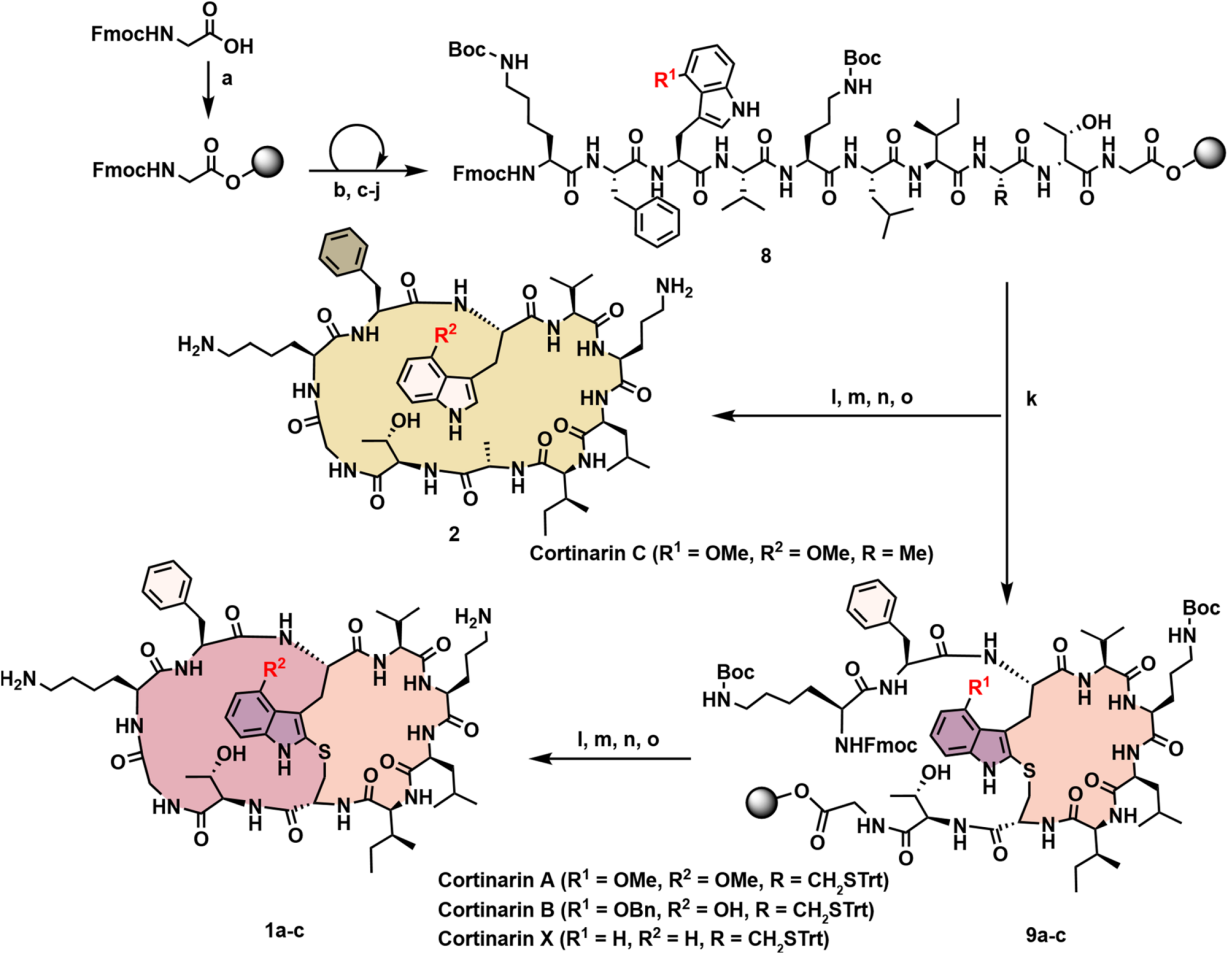
General synthetic route to cortinarins. (a) Resin loading: 2-CTC resin, DIPEA, DCM, 2 h; (b) Fmoc deprotection: piperidine/DMF (1 : 4, v/v); (c–j) peptide chain elongation (coupling): Fmoc-AA-OH, HATU, DIPEA, DMF (with amino acids in the following sequence: d-Thr, l-Cys(Trt), l-Ile, l-Leu, l-Orn(Boc), l-Val, l-Trp(4-OMe/4-OBn/4-H), l-Phe, l-Lys(Boc)); (k) iodine-mediated cyclization: 2 mg mL^−1^ I_2_ in DMF, 3 h; (l) Fmoc deprotection: piperidine/DMF (1 : 4, v/v); (m) cleavage from resin: HFIP/DCM (1 : 4, v/v); (n) macrolactamization: HATU, DIPEA, DMF (peptide concentration: 2 µM), 12 h. (o) Global deprotection: for cortinarin A (1a), C (2), X (1c): TFA/DCM (3 : 7, v/v), 1 h; for cortinarin B (1b): BF_3_·Et_2_O in EtSH, 1 h.

We herein report a strategy for the total synthesis of cortinarins, featuring iodine-mediated Trp–Cys cyclization as a pivotal step (Fig. 1 and Scheme 1).^26^ As shown previously, the tryptathionine bridge in bicyclic peptides like phalloidin and amanitin causes a special case of atropisomerism, which requires analytical differentiation of the two isomers, designated as *P*- and *M*-ansamers.^27,28^ As first proposed by Wieland *et al.*^29^ and later investigated by Kessler and co-workers,^30^ the tryptathionine bridge in bicyclic peptides such as phalloidin and amanitin gives rise to a distinct form of non-classical atropisomerism.^31^ Evidence for the existence of a non-natural amanitin-type atropisomer was first provided by X-ray crystallography in our work.^27^ To enable an unambiguous assignment of these peptide atropisomers, we introduced the ansamer concept, providing a readily applicable framework for determining the conformational topology of bicyclic systems (*e.g.*, amanitin, norbornapeptides, in/out peptides, tryptorubin) as well as monocyclic peptides (*e.g.*, lasso peptides).^27^ We thus analysed the three-dimensional structures of cortinarins in DMSO using 2D NMR spectroscopy and molecular dynamics (MD) simulations, which allowed us to unambiguously assign the synthetic products to the *P*- or *M*-ansamer conformation. While efforts to identify a gene cluster for cortinarin biosynthesis were hampered due to a lack of genomic data, we show that the synthetic cortinarins mimic the cellular effect of the peptide hormone arginine vasopressin (AVP; Fig. 1), to which corresponding structural features can be found, as also previously suggested.^24^

## Results and discussion

### Biosynthetic origin, retrosynthesis and general synthetic route to cortinarins

To gain insight into the biosynthetic origin of the cortinarins, we sought to analyse fungal sample material. Neither the genome sequence of the original producer strain *C*. *speciosissimus* reported by Tebbet and Caddy,^24^ nor sequences of *C. rubellus* (synonymous with *C. speciosissimus*) or *C. orellanus* are currently available in public data bases. We thus collected and analysed various mushroom samples of the genus *Cortinarius* identified by sequencing ITS (internal transcribed spacer) regions (Table S1). However, we were not able to identify the anticipated biosynthetic genes (coding for a precursor peptide and prolyl oligopeptidase^32,33^) in the corresponding genomes (see SI). Likewise, methanol extracts of these strains were devoid of cortinarins as examined by HPLC-ESI-MS (Table S1). Owing to this lack of biological supply, we envisaged to gain synthetic access to cortinarins and to study their biological activity.

The characteristic structural feature of the CorA and CorB is the tryptathionine bridge (Trp–Cys crosslink) which also occurs in the well-known phallotoxins and amatoxins.^34^ While the classical Savige–Fontana reaction remains a benchmark for constructing the tryptathionine bridge,^35,36^ its reliance on Trp-oxidation with dimethyldioxirane (DMDO) poses a potential safety risk and the trifluoroacetic acid (TFA)-mediated cyclization shows limitations for performing solid-phase peptide synthesis (SPPS). To address these drawbacks, our group previously elaborated a smooth iodine-mediated oxidative Trp–Cys coupling strategy that facilitated the total synthesis of phalloidin, amanitin and various analogues thereof.^26,28^ Capitalizing on this advance, we envisioned a convergent synthesis route that could efficiently provide all cortinarins (Fig. 1). In retrosynthetic terms, the cortinarin scaffold 1 was deconstructed *via* sidechain-protected precursor 3 into monocyclic peptide 4 through disconnection of the amide bond linking the C-terminal Gly^10^ to the N-terminal Lys^1^, thus circumventing epimerization during macrolactamization. The synthesis of monocyclic peptide 4, including construction of the tryptathionine bridge (compound 5), was designed to be performed on solid support *via* a linear peptide (compound 6).

To test the principal feasibility of peptide assembly, we first synthesized a simplified derivative without Trp-modifications, which we termed cortinarin X (CorX). In later stages of our synthesis efforts for CorA and CorB, the amino acid building blocks Fmoc-l-Trp(4-OMe)-OH 7a and Fmoc-l-Trp(4-OBn)-OH 7b needed to be prepared individually (Fig. 1 and Scheme S1), as they are not commercially available. The modified tryptophans 7a and 7b (Fig. 1) were obtained over seven steps, from a didehydroamino acid intermediate by asymmetric metal-catalysed hydrogenation.^28,37^ With 7a and 7b in hand, the amino acid building blocks were integrated into the linear peptide 8 (Scheme 1). In all cases of cortinarin syntheses, the tryptathionine-bridge of monocyclic peptides 9a–c was established straightforwardly by iodine in DMF (conc. 2 mg mL^−1^). This was followed by Fmoc deprotection and cleavage of the monocyclic peptide from the resin. Considering that the cleaved peptides all have a strong tendency to aggregate and not well redissolve in various solvents (*e.g.* DMSO, DMF, and MeOH), regeneration of a fluffy amorphous solid state turned out to be required for subsequent synthesis steps (Fig. S1).

The macrolactamization was performed with HATU, and the crude product was purified by reverse-phase automated flash chromatography. The global deprotection, and HPLC purification, afforded the desired cortinarins (Scheme 1). Notably, this approach provides modular access to bicyclic cortinarins (A 1a, B 1b and X 1c) and monocyclic cortinarin C (2) in milligram-quantities, the latter through selective cysteine-to-alanine substitution in SPPS linear peptide synthesis.

### Ansamer formation and conformational analysis of cortinarins

**Fig. 2:**
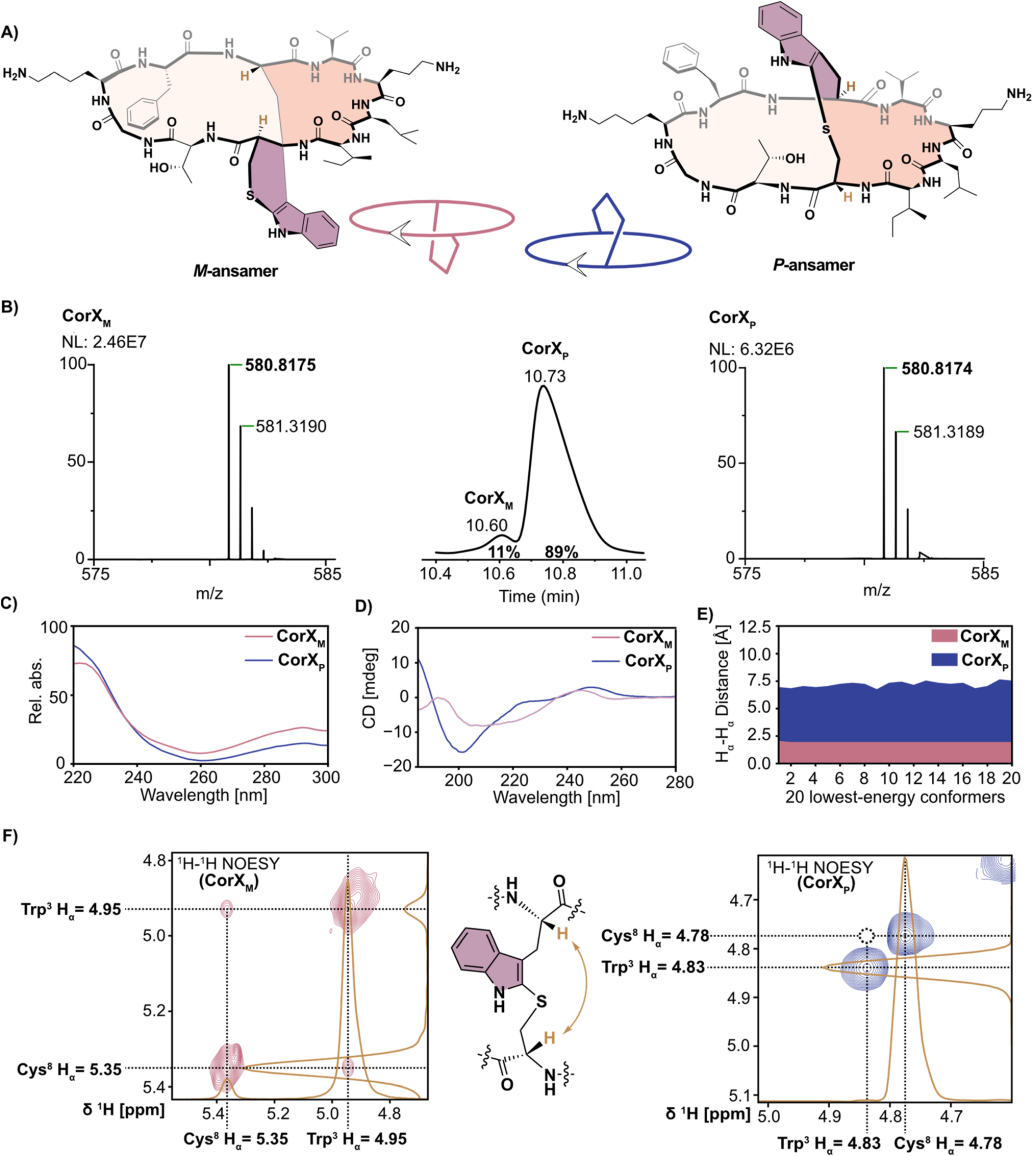
Analytical characterization of CorX ansamers. (A) For clockwise N → C orientation of the peptide, a bridge below the backbone defines *M*-ansamer CorX_*M*_, whereas a bridge above defines *P*-ansamer CorX_*P*_. (B) HPLC chromatogram and HR-ESI-MS of CorX_*M*_ and CorX_*P*_. The ansamer ratio (∼1 : 9) was interpolated by integration of peaks. Mass spectrometric signals correspond to the doubly protonated [M + 2H]^2+^ ion (calculated [C_57_H_87_N_13_O_11_S]^2+^, *m*/*z* 580.8179 Da). (C) UV-absorption spectra and (D) far-UV CD spectra of CorX_*M*_ (red) and CorX_*P*_ (blue) recorded in H_2_O. (E) Distribution of distances between the Hα protons of Trp^3^ and Cys^8^ in the 20 lowest-energy conformers of CorX_*M*_ (red) and CorX_*P*_ (blue). (F) Diagnostic sections of 2D NOESY spectra of CorX_*M*_ (left) and CorX_*P*_ (right) showing the signals of Hα protons of Cys^3^ and Trp^8^. The orange traces depict representative 1D projections (slices) at the corresponding chemical shifts in both compounds. The position of a theoretical NOE cross-peak for CorX_*P*_ is indicated with a dashed circle.

More recently it became increasingly apparent that peptides can exhibit non-classical atropisomerism, which must be taken into account in total synthesis, *e.g.* of darobactin, tryptorubin, phalloidin and amanitin.^26,28,35,36,38,39^ Our group previously introduced the ansamer concept for peptides providing a framework to unambiguously define the conformational topology of bicyclic (amanitin, norbornapeptides, tryptorubin) but also of monocyclic peptides (lasso peptides).^27^ The concept provides a means to differentiate between topological isomers of macrocyclic peptides, depending on whether the bridging molecular feature is positioned above (*P*-ansamer) or below (*M*-ansamer) the macrocyclic plane (by definition the peptide backbone runs clockwise from N- to C-terminus, see Fig. 2A).^27^ This concept also applies to synthetic bicyclic peptides of the cortinarin-type: during the synthesis of non-natural CorX (1c), the formation of ansamers was particularly pronounced, whereas this effect was negligible for CorA (1a) and CorB (1b) (<5%), likely as a result of steric effects of indole modifications. Following macrolactamization and global deprotection, two CorX species, designated as CorX_*M*_ and CorX_*P*_, were observed by HPLC with retention times of 10.60 min and 10.73 min, respectively (Fig. 2B). Subsequent HR-ESI-MS analysis revealed that both species exhibited isobaric masses (Fig. 2B). These two peaks, which occurred in an approximate 1 : 9 ratio, were separated by preparative HPLC (yields of 4.2 mg and 34.5 mg, respectively) and analytically characterized. A systematic scan of alternative macrolactamization sites of CorX (Table S2 and Fig. S2), however, showed no selectivity enhancement toward the *M*-ansamer, CorX_*M*_. To rule out epimerization during peptide assembly, amino acid analysis was performed with Marfey's reagent,^40^ revealing identical amino acid configurations for both compounds (Fig. S3). The UV absorption spectra were characteristic of the tryptathionine scaffold,^27^ albeit with slightly different absorption maxima of *λ* = 291 nm and *λ* = 293 nm for CorX_*M*_ and CorX_*P*_, respectively (Fig. 2C). In contrast, the CD spectra of CorX_*M*_ and CorX_*P*_ differed substantially, consistent with different backbone conformations (Fig. 2D). The CD spectrum of compound CorX_*P*_ showed a pronounced minimum at *λ* = 201 nm and a maximum at 248 nm. In contrast, CorX_*M*_ exhibited a minimum at 208 nm and a maximum at 245 nm. NMR spectroscopic assignments were established for the two CorX isomers in DMSO-*d*_6_ (Tables S9 and S11), giving rise to inter-residual NOE connectivities (Tables S22 and S23). The two CorX isomers clearly differed in diagnostic NOE signals of Hα protons between the bridging residues Trp^3^ and Cys^8^. A prominent NOE cross peak was observed for isomer CorX_*M*_ between Trp^3^ (*δ*_Hα_ = 4.95 ppm) and Cys^8^ (*δ*_Hα_ = 5.35 ppm). In contrast, this correlation was absent in isomer CorX_*P*_ (Trp^3^, *δ*_Hα_ = 4.83 ppm; Cys^8^, *δ*_Hα_ = 4.78 ppm) (Fig. 2F). As previously established for amanitin, the *M*-ansamer locates the corresponding Hα protons in close distance of about 2.7 Å and gives a strong NOE cross peak (as in isomer CorX_*M*_), whereas these protons are oriented away from each other in the *P*-ansamer with a distance of about 7.5 Å and no detectable NOE signal (as in isomer CorX_*P*_). Hence, in combination with UV and CD spectra, the characteristic NOE signal between Hα protons of Trp and Cys serves as a reliable diagnostic marker for the unambiguous assignment of the corresponding ansamer. Based on this method, we identified the synthetic products CorA and CorB as the *P*-ansamers (Fig. S6 and S7), which is consistent with previously synthesized tryptathionine-containing natural products, such as phalloidin and amanitin.^27,28,35,36,41^

Having assigned the topological descriptors, we next investigated the stability of these ansamers. Attempts to induce thermal interconversion between CorX_*P*_ and CorX_*M*_ by heating (DMSO-*d*_6_, up to 50 °C) did not result in significant chemical shift changes in NMR spectra, suggesting that both ansamers remain conformationally locked under the applied conditions (Fig. S14). These findings are further supported by MD simulations in DMSO. Two ansamer-defining dihedrals identifiers *θ*_C,CB_ or *θ*_W,CB_ (Fig. S15) were monitored throughout the 1 µs MD simulations to assess the orientation of the tryptathionine bridge relative to the cyclic backbone (Fig. S15). The resulting dihedral probability density maps showed that each ansamer remained confined to their respective allowed dihedral angles (Fig. S16). No flipping of the tryptathionine bridge and thus no transition between CorX_*P*_ and CorX_*M*_ occurred in the time window of 1 µs.

**Fig. 3:**
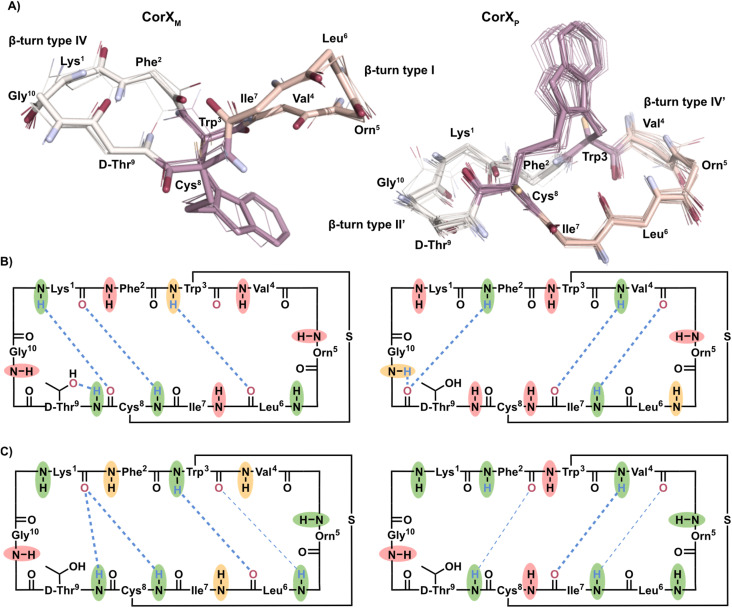
Conformational ensembles and hydrogen bonding patterns of CorX ansamers. (A) NOE-based structure ensembles of CorX_*M*_ (left) and CorX_*P*_ (right) derived from NMR data acquired in DMSO at 298 K (line representation). The lowest-energy conformer is depicted in stick model. Sidechains are omitted for clarity (except for the tryptathionine bridge). Oxygen and nitrogen atoms of peptide bonds are indicated in red and blue, respectively. Types of turn structures are indicated. (B) Simplified representation of the peptide backbone of the lowest-energy conformer of CorX_*M*_ (left) and CorX_*P*_ (right) and observed hydrogen bonds (blue dashed lines). Amide protons exhibiting small temperature coefficients (−Δ*δ*_HN_/Δ*T* < 3.0 ppb K^−1^) are indicative of H-bonds (green), whereas solvent-exposed amides show large temperature coefficients (−Δ*δ*_HN_/Δ*T* > 4.6 ppb K^−1^, red). Intermediate values are shown in yellow. (C) Simplified representation of the cyclic peptide backbone from unrestrained MD simulations (see SI, Section S9) starting from the NMR-derived lowest-energy conformer of CorX_*M*_ (left) and CorX_*P*_ (right). Hydrogen bonds are depicted as blue dashed lines with the line width being proportional to the average population over three 1 µs replicates. Bold dashed lines correspond to hydrogen bonds present in >50% of the simulation, whereas thin dashed lines indicate 10–50% occurrence. Amide groups with low solvent-accessible surface area (SASA < 0.05 nm^2^) are highlighted in green, highly exposed amides (SASA > 0.15 nm^2^) are circled in red, and those with intermediate accessibility are shown in yellow (exact SASA values are listed in Tables S32 and S33).

NOE-restrained structure calculations using the software CYANA^42–47^ afforded conformational ensembles for CorX_*P*_ and CorX_*M*_ in DMSO-*d*_6_ at 298 K (Fig. 3A). Analysis of the lowest-energy conformer of CorX_*P*_ revealed a type II′ β-turn formed by residues Cys^8^ (C=O) and Lys^1^ (NH), alongside a type IV β-turn formed by residues Trp^3^ (C=O) and Leu^6^ (NH). In contrast, the lowest-energy conformer of CorX_*M*_ featured a type IV β-turn established between d-Thr^9^ (C=O) and Phe^2^ (NH), as well as a type I β-turn comprising residues Val^4^ (C=O) and Ile^7^ (NH). These turn motifs were present in the majority of the 20 lowest-energy conformers of each peptide (Fig. 3A), indicating that they represent the dominant conformations of the respective ansamer. Furthermore, the absence of strong NOE signals between adjacent Hα protons indicates that all peptide bonds adopt an s-*trans* configuration.

Temperature-dependent chemical shift variations of amide protons (temperature coefficients −Δ*δ*_HN_/Δ*T*) provide insights into solvent exchange and thus their potential involvement in hydrogen bonding. Small temperature coefficients (−Δ*δ*_HN_/Δ*T* < 3.0 ppb K^−1^) were observed for amide protons of residues Lys^1^, Trp^3^, Leu^6^, Cys^8^ and d-Thr^9^ in CorX_*P*_, and residues Phe^2^, Val^4^ and Ile^7^ in CorX_*M*_, including those amides involved in β-turns (Tables S26 and S29). Hence, these potential hydrogen-bonds are consistent with the NOE-based structure ensembles of both ansamers (Fig. 3B), except for Leu^6^ in CorX_*P*_.

An unrestrained 1 µs MD simulation in DMSO initiated from the lowest-energy conformer of each ansamer revealed an overall conservation of the peptide structures (Fig. 3C). Ensemble-averaged inter-proton distances derived from MD simulations showed good agreement with experimental NOE distances, with the majority of deviations below 2 Å (Fig. S17 and S18). Notably, the diagnostic distances between Hα atoms of Trp^3^ and Cys^8^ were 2.2 Å in CorX_*M*_ and 7.5 Å in CorX_*P*_, consistent with the presence or absence of corresponding NOE signal. Solvent accessible surface area (SASA) analysis (Fig. 3C) revealed some discrepancies with NMR-derived hydrogens bonds (Fig. 3B). In particular residues Phe^2^, Val^4^, and Orn^5^ in CorX_*M*_ and residues Lys^1^, Orn^5^, and d-Thr^9^ in CorX_*P*_ were predicted to have low solvent accessibility, yet exhibited high temperature coefficients, suggesting that these amides may be buried within the hydrophobic core but are not stabilized by specific hydrogen bonds.

### Cortinarins affect the localization of aquaporin-2 in renal principal cells

Cortinarin intoxications are associated with decreased urine output,^24^ which could relate to the action of the peptide hormone AVP in the kidney. In the kidney, AVP binds to vasopressin V2 receptors (V2R) on the basolateral surface of collecting duct principal cells, which activates stimulatory G proteins, G_s_, and adenylyl cyclases to generate cyclic adenosine monophosphate (cAMP).^48–50^ cAMP, in turn, activates protein kinase A (PKA) and downstream signalling, resulting in the translocation of aquaporin-2 (AQP2) water channels from intracellular vesicles into the plasma membrane. The plasma membrane insertion increases the osmotic water permeability of the cells and facilitates water reabsorption from primary urine and thereby fine-tunes body water homeostasis.^51,52^

**Fig. 4:**
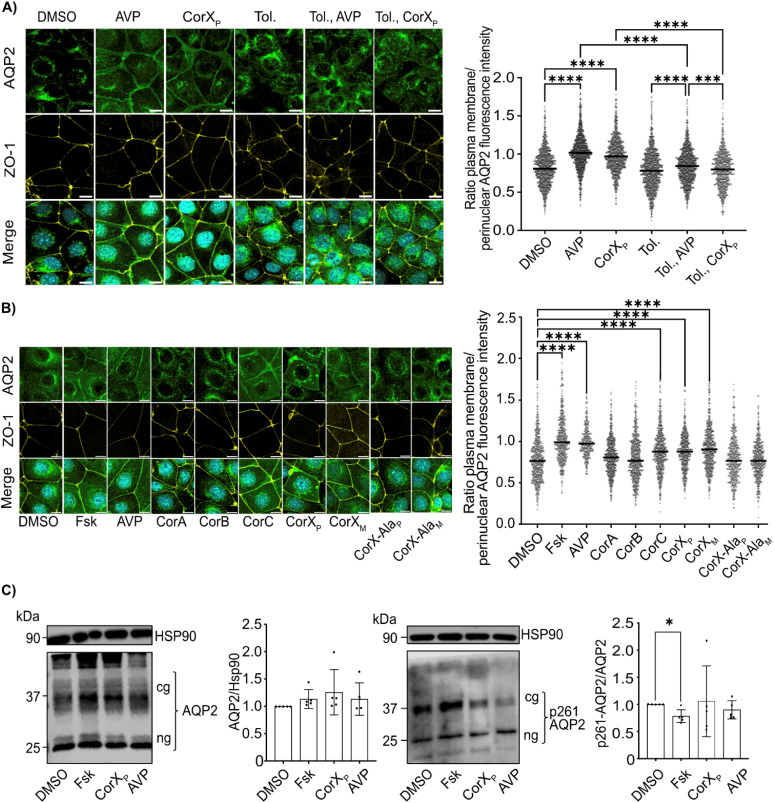
Biological effects of cortinarins and their analogs on the localization of AQP2 in MCD4-V2R cells. (A and B) Left: MCD4-V2R cells were subjected to the indicated treatments: DMSO (0.1%, 0.5 h), AVP (100 nM, 0.5 h), cortinarins (CorX_*P*_ CorX_*M*_, CorA, CorB, CorC, CorX-Ala_*P*_, CorX-Ala_*M*_; 20 µM, 1 h), tolvaptan (1 µM, 1 h). When agents were combined, the total incubation time was 1.5 h. AQP2 was detected using specific primary antibody (AQP2-H27) and Cy3-coupled anti-rabbit secondary antibody (green). Nuclei were stained with DAPI (blue). ZO-1 plasma membrane marker was detected with Alexa 647-coupled anti-rat antibody (yellow). The images were obtained under 40× magnification. Scale bar, 10 µm. Right: AQP2 fluorescence signals were calculated by a single cell segmentation approach. Each data point represents the AQP2 signal from one cell. Membrane/cytoplasm ratios >1 indicate AQP2 localization at the plasma membrane, while ratios <1 indicate AQP2 located intracellularly. Shown are means ± SEM of 3 independent experiments per condition. Kruskal–Wallis test with Dunn's multiple comparisons were used as a non-parametric test for statistical evaluation. Values are presented as mean values ± SEM. Statistically significant differences are shown, ****p < 0.0001, ***p < 0.001, **p < 0.01 and *p < 0.05. (C) MCD4-V2R cells were treated with DMSO (0.1%, 0.5 h), forskolin (Fsk 30 µM, 0.5 h), AVP (100 nM, 0.5 h) or CorX_*P*_ (20 µM, 1 h). The cells were lysed, the proteins separated by SDS-PAGE and AQP2 and AQP2 phosphorylated at serine 261 (pSer261-AQP2) were detected by western blotting. HSP90 served as a loading control. Shown are representative blots of *n* = 3 independent experiments. Semiquantitative analysis of the AQP2 protein abundance relative to HSP90 and of AQP2 phosphorylated at serine 261 (p261-AQP2) relative to unphosphorylated AQP2 and HSP90 was carried out using densitometry. A one-way ANOVA was performed for statistical evaluation of differences compared to control cells (DMSO-treated) are indicated (means ± SEM of *n* = 3). Statistically significant differences are indicated, *P* < 0.05, *P < 0.01.

The structural similarity of cortinarins to AVP, together with the observed reduction in urine output during cortinarin intoxication, prompted us to investigate whether CorX—providing both ansamers (CorX_*M*_ and CorX_*P*_) as a surrogate compound—exerts effects comparable to AVP on renal collecting duct principal cells. To test this, we employed an established culture model of principal cells, namely MCD4-V2R cells.^53^ The cells stably express human AQP2 and the human vasopressin type 2 receptor, V2R. As previously shown,^53,54^ stimulation with AVP induced the redistribution of AQP2 to the plasma membrane (Fig. 4A). Notably, CorX_*P*_ and CorX_*M*_ both demonstrated a comparable effect, showing that they mimic the effect of AVP (Fig. 4A and B). Interestingly, CorC exhibited efficacy equivalent to that of CorX_*P*_ and CorX_*M*_, but CorA and CorB showed no observable effect (Fig. 4B). A synthetic analog of CorX, in which Lys^1^ and Orn^5^ were replaced by Ala (CorX-Ala), likewise populated two ansamer states, *i.e.* CorX-Ala_*M*_ and CorX-Ala_*P*_. For their differentiation and assignment, a prominent NOE cross-peak between Hα protons of Trp^3^ and Cys^8^ was observed for CorX-Ala_*M*_ (Fig. S9), whereas no corresponding cross-peak was detected for CorX-Ala_*P*_ (Fig. S8). Notably, in the AQP2 assay both ansamers completely lost the activity in promoting AQP2 translocation to the plasma membrane (Fig. 4B), indicating that the amino groups are critical for target engagement. These results support our conclusion that the orientation of the tryptathionine bridge does not affect biological efficacy. V2R stimulation causes a rise of cAMP which can be mimicked by direct activation of adenylyl cyclases with forskolin. The cAMP-induced redistribution of AQP2 to the plasma membrane is associated with a decrease of its phosphorylation at Ser261. However, CorX_*P*_ did not affect this phosphorylation, suggesting that its mode of action differs from that of AVP (Fig. 4C). The structural difference of CorX to CorA/B may let assume that the *M*-ansamer is the active component or that the role of the monocyclic CorC for exertion of a physiological effect is even more important than anticipated. Further investigations into the precise mode of action of CorX and the other CorA/B ansamers are critical for understanding its effect on the localization of AQP2 in renal collecting duct principal cells.

## Conclusions

In this study we accomplished the chemical synthesis of previously reported cortinarins A–C.^24^ In the light of prior observations for amanitin/amanullin syntheses,^27,36^ formation of ansamers can also be observed for cyclodecapeptides of the cortinarin-type and is therefore not limited to smaller 7-mer (phallotoxins) and 8-mer (amatoxins) systems. Similar to the amatoxin pair amanitin/amanullin, oxidative modifications of the Trp indole moiety, such as hydroxy and methoxy groups, appear to sterically hinder ansamer formation during synthesis (CorX *vs.* CorA/B). Furthermore, no thermal interconversion was observed for CorX during 1 µs MD simulations, despite the presence of two additional amino acids in the macrocyclic ring relative to amanitins.

The initial description of the cortinarins by Tebbett and co-workers faced scepticism,^24^ when Laatsch *et al.* (1990) reinvestigated *C. speciosissimus* and found no evidence for production of the claimed peptides.^55^ Despite Tebbett's defence in 1991, independent validation of cortinarin production has not been reported yet, and we also could not confirm the existence of cortinarins in related *Cortinarius* mushrooms. This could be due to a questionable taxonomic assignment of the producing species. However, we do observe a similar behaviour regarding conformation and atropisomerism as in phalloidins and amanitins. Under biosynthetic aspects, the cortinarins possibly constitute an extension of the group of tryptathionine-containing peptides, previously confined to amatoxins and phallotoxins.^34^ Finally, in light of mushroom intoxications reported for *Cortinarius* species, we focused our investigation on their characteristic secondary metabolites, the cortinarins. Our cell-based studies indicate that cortinarins act similarly to AVP. While their precise mode of action remains to be elucidated, these cyclic peptides may thus serve as potential lead compounds for the treatment of renal function disorders, Dysregulation inhibiting the plasma membrane insertion of AQP2 causes diabetes insipidus, a disease characterised by a daily loss of up to 20 L of hypotonic urine. Although AVP substitution is available for the treatment of a subset of patients, a medical need for novel drugs for a targeted treatment of the majority of millions of patients persists.^56^ Our observation that CorX promotes the localization of AQP2 to the plasma membrane highlights cortinarin-type cyclopeptides as promising candidates for the development of innovative therapies for diabetes insipidus.

## Author contributions

R. D. S., B. G. K. and E. K. supervised the project and acquired funding. Z. H. and G. Y. conducted the synthesis experiment and data analysis. C. J. conducted biological experiments and data analysis. J.-L. S. performed molecular dynamics simulations. R. J. provided samples of fungi of the genus *Cortinarius*. A. Mü. conducted experiment on molecular biology and bioinformatics. Z. H. conducted NOE-based structure calculations. A. Ma. performed NMR measurements and supervised the conformation calculations together with S. K. All authors contributed to writing, reviewing, and editing the manuscript and gave approval to the final version.

## Conflicts of interest

There are no conflicts to declare.

## Supplementary Material

SC-OLF-D6SC02205G-s001

## Data Availability

Supplementary information (SI): all experimental data, characterisation results, and synthetic procedures. See DOI: https://doi.org/10.1039/d6sc02205g.
