# Supplementary material for: Synthesis and characterization of cortinarins – cryptic cyclic peptides from mushrooms of the genus Cortinarius
Source: Chem Sci. 2026 Jun 23;17(31):14983–92. doi: 10.1039/d6sc02205g (PMC13289786; doi:10.1039/d6sc02205g)
Supplement: Supplementary file 1 [file SC-017-D6SC02205G-s001.pdf]

Supplementary Information (SI) for Chemical Science.
This journal is © The Royal Society of Chemistry 2026

Supplementary information

Synthesis and characterization of cortinarins - cryptic cyclic peptides from mushrooms of the genus *Cortinarius*

Zhanyu He†[a], Celine Janssen†[b], Joana-Lysiane Schäfer[d], Agnes Mühlenweg[a], Simone Kosol[e], Rene Jarling[f], Andi Mainz[a], Bettina G. Keller[d], Guiyang Yao*[c], Enno Klußmann*[b,g], Roderich D. Süssmuth*[a]

[a] Institut für Chemie, Technische Universität Berlin, Straße des 17. Juni 115, D-10623 Berlin, Germany.
[b] Max-Delbrück-Center for Molecular Medicine in the Helmholtz Association (MDC), Robert-Rössle Straße 10, D-13125 Berlin, Germany.
[c] Greater Bay Area Institute of Precision Medicine (Guangzhou), Guangzhou 510000, PR China.
[d] Department of Biology, Chemistry, and Pharmacy, Freie Universität Berlin, *Arnimallee 22*, D-14195 Berlin, Germany.
[e] MSB Medical School Berlin, Hochschule für Gesundheit und Medizin, Rüdesheimer Straße 50, D-14197 Berlin, Germany.
[f] ZE Botanischer Garten und Botanisches Museum Berlin, Freie Universität Berlin, Königin-Luise Straße 6-8, D-14195 Berlin, Germany.
[g] DZHK (German Centre for Cardiovascular Research), partner site Berlin, Potsdamer Straße 58, D-10785 Berlin, Germany.

† These authors contributed equally to this work.

* Corresponding authors

Table of content

1 Genomic DNA isolation and PCR amplification 6
2 Peptide synthesis protocol and characterization data 8
3 Amino acid analysis with Marfey's reagent 29
4 UV spectra of synthetic cortinarin A, B and C 31
5 CD spectra of synthetic cortinarin A, B, and C 31
6 NMR analysis of cortinarin analogs 32
7 CYANA peptide structure calculations 44
8 Variable-temperature (VT) NMR spectroscopy 65
9 Molecular dynamics (MD) simulations 68
10 Immunofluorescence microscopy and Western blotting 73
11 NMR Spectra 74
12 Reference 101
13 Appendix 102

List of supplementary tables

Table S1. Results for ITS-sequencing and LC-MS detection of mushrooms of the genus *Cortinarius.* 6

Table S2. Scan for an optimized macrolactamization site for the significant ansamer selectivity in the synthesis of CorX. 25

Table S3. ^{1}H-NMR chemical shifts [ppm] of CorA$_P$ (1a). 33

Table S4. ^{1}H, ^{13}C-HSQC chemical shifts [ppm] of CorA$_P$ (1a). 33

Table S5. ^{1}H-NMR chemical shifts (ppm) of CorB$_P$ (1b). 35

Table S6. ^{1}H, ^{13}C-HSQC chemical shifts [ppm] of CorB$_P$ (1b). 35

Table S7. ^{1}H-NMR chemical shifts [ppm] of CorC (2). 37

Table S8. ^{1}H, ^{13}C-HSQC chemical shifts [ppm] of CorC (2). 37

Table S9. ^{1}H-NMR chemical shifts [ppm] of CorX$_P$ (1c$_B$). [1] 38

Table S10. ^{1}H, ^{13}C-HSQC chemical shifts [ppm] of CorX$_P$ (1c$_B$). 38

Table S11. ^{1}H-NMR chemical shifts [ppm] of CorX$_M$ (1c$_A$). 39

Table S12. ^{1}H, ^{13}C-HSQC chemical shifts [ppm] of CorX$_M$ (1c$_A$). 39

Table S13. ^{1}H-NMR chemical shifts [ppm] of CorX-Ala$_P$ (S20$_A$). 40

Table S14. ^{1}H, ^{13}C-HSQC chemical shifts [ppm] of CorX-Ala$_P$ (S20$_A$). 40

Table S15. ^{1}H-NMR chemical shifts [ppm] of CorX-Ala$_M$ (S20$_B$). 42

Table S16. ^{1}H, ^{13}C-HSQC chemical shifts [ppm] of CorX-Ala$_M$ (S20$_B$). 42

Table S17. CYANA library file for H-L-Trp(2-SMe)-OH. 44

Table S18. CYANA library file for H-L-Cys(S-CH=CH$_2$)-OH. 45

Table S19. CYANA library file for H-Gly-NHMe. 45

Table S20. CYANA library file for Ac-L-Lys-OH. 46

Table S21. CYANA library file for H-D-Thr-OH. 47

Table S22. Assignment of NOE signals for CorX$_P$ (1c$_B$). 48

Table S23. Assignment of NOE signals for CorX$_M$ (1c$_A$). 51

Table S24. Overview of CYANA structure calculation. 59

Table S25. Summary of the assessment of 20 calculated conformers of CorX$_M$ (1c$_A$) for allowed and disallowed dihedral angles. 60

Table S26. Turn motifs in the lowest-energy conformer of CorX$_M$ (1c$_A$). 61

Table S27. Overview of CYANA structure calculation. 62

Table S28. Summary of the assessment of 20 calculated conformers of CorX$_P$ (1c$_B$) for allowed and disallowed dihedral angles. 63

Table S29. Turn motifs in the lowest-energy conformer of CorX$_P$ (1c$_B$). 64

Table S30. Temperature-dependent chemical shift changes $\Delta\delta_{HN}/\Delta T$ (ppb/K) for amide and hydroxyl protons of CorX$_P$ as observed by VT-NMR 66

Table S31. Temperature-dependent chemical shift changes $\Delta\delta_{HN}/\Delta T$ (ppb/K) for amide and hydroxyl protons of CorX$_M$ as observed by VT-NMR 67

Table S32. SASA analysis for CorX$_P$. 72

Table S33. SASA analysis for CorX$_M$. 72

Table S 34. The SMILES code for cortinarins derivatives 102

List of supplementary schemes

Scheme S1. Synthesis of tryptophan analogs. 10
Scheme S2. Synthesis of Cortinarin A (1a). 16
Scheme S3. Synthesis of cortinarins B (1b). 18
Scheme S4. Synthesis of monocyclic cortinarin C (2). 20
Scheme S5. Synthesis of cortinarin X (1c). 22
Scheme S6. Synthesis of cortinarin X-Ala (S20). 27

List of supplementary figures

Figure S1. Workflow for the regeneration of cleaved decapeptide from ether-precipitates. 15

Figure S3. Marfey analytics of amino acids in $CorX_P$ and $CorX_M$. 30

Figure S4. UV spectra of cortinarin A, cortinarin B, and cortinarin C. 31

Figure S5. Far-UV CD spectra of cortinarin A, cortinarin B, and cortinarin C. 31

Figure S6. Diagnostic NOESY region of the Hα atoms of Cys^8 and (4-OMe)Trp^3 in NMR spectra of $CorA_P$ (blue contours). 34

Figure S7. Diagnostic NOESY signals of the Hα atoms of Cys^8 and (4-OH)Trp^3 in NMR spectra of $CorB_P$ (blue contours). 36

Figure S8. Diagnostic NOE signals of the Hα atoms of Cys^8 and Trp^3 in NMR spectra of CorX-Ala_P (blue contours). 41

Figure S9. Diagnostic NOE signals of the Hα atoms of Cys^8 and Trp^3 in NMR spectra of CorX-Ala_M (red contours). 43

Figure S10. Ramachandran Plot of $CorX_M$ ($1c_A$). 60

Figure S11. NMR structure ensemble showing the 20 lowest energy conformers of $CorX_M$ ($1c_A$). 61

Figure S12. Ramachandran Plot of $CorX_p$ ($1c_B$). 63

Figure S13. NMR structure ensemble showing the 20 lowest energy conformers of $CorX_P$ ($1c_B$). 64

Figure S14. Variable-temperature (VT) ^{1}H-NMR spectroscopy study of $CorX_P$ (*top*) and $CorX_M$ (*bottom*) (temperature range 298 K - 333 K as indicated). 65

Figure S15. Ansamer dihedrals definition. 70

Figure S16. Illustration of ansamer dihedrals probability density. 70

Figure S17. Comparison of NOE and MD data for $CorX_P$. 71

Figure S18. Comparison of NOE and MD data for $CorX_M$. 71

1 Genomic DNA isolation and PCR amplification

Genomic DNA was isolated by pulverizing an approx. 0.5 cm^2 dried or fresh mushroom sample with sea sand in a mortar preheated to 60 °C. After 0.5 mL of 2x CTAB buffer [100 mM Tris (pH 8.0), 1.4 M NaCl, 20 mM EDTA, cetyltrimethylammonium bromide (CTAB, (2% w/v))] was added, the sample was incubated for 30 min at 60 °C with gentle shaking and then centrifuged at 14,000 rpm for 10 min. The aqueous supernatant was mixed with 500 µL phenol/chloroform/isoamyl (25:24:1, pH 7.5-8.0) and vortexed. After a further centrifugation step, 500 µL isopropanol was added to the upper aqueous phase and the sample was carefully inverted. The precipitated genomic DNA was collected with a curved glass pipette, washed with ethanol (70% v/v) and dissolved in TE buffer [10 mM Tris HCl (pH = 8.0), 1 mM EDTA (pH 8.0)]. The ITS (internal transcribed spacer) region was amplified with primer pair ITS1 (5'–TCCGTAGGTGAACCTGCGG–3'/ITS2 (5'–GCTGCGTTCTTCATCGATGC–3')[1] using Q5-Polymerase (New England Biolabs). Amplified PCR products were purified with the GeneJET Exctration Gel Kit (Thermo Fisher Scientific GmbH), sequenced with ITS1 primer and analyzed with Basic Local Alignment Search Tool BlastN.[2]

Following the work of He et al. (2020)[3], the collected strains were screened via PCR for the presence of cyclopeptides of MSDIN family (primer forward (5′-ATGTCNGAYATYAAYGCNACNCG-3′), primer reverse (5′-CCAAGCCTRAYAWRGTCMACAAC-3’). No PCR product could be amplified using the genomic DNA of the strains listed in **Table S1**.

1.1 HPLC-ESI-Mass spectrometry

Mushroom samples were mortared with sea sand as described above and the powder was resuspended in 2 mL methanol. The samples were incubated under shaking for 30 min at room temperature. After centrifugation at 14,000 rpm for 10 min, 5 µL of the supernatant were measured with either an Exactive hybrid quadrupole-orbitrap or an LTQ-Orbitrap XL hybrid ion trap-orbitrap (Thermo Fisher Scientific GmbH, Bremen, Germany).

Table S1. Results for ITS-sequencing and LC-MS detection of mushrooms of the genus *Cortinarius.*

Species (ITS sequencing result)	Homololgy to (genbank accession number)	Location	Herbarium B number [1]	LC-MS detected
Amanita phalloides	PV804301	Germany, Brandenburg, nearby Reisdorf	B 70 0132466	a-amanitin, β-amanitin, phalloidin, phallacidin, phalloin
C. orellanus	MZ005538	Germany, Brandenburg, nearby Rüdersdorf	B 70 0132467	orellanine, phalloidin, phalloin
C. orellanus	MZ005538	Germany, Brandenburg, nearby Rüdersdorf	B 70 0132468	orellanine, phalloidin, phalloin
C. rubellus	PV121635	Germany, Thuringia, Thuringian Forest	B 70 0132469	orellanine, phalloidin, phalloin

C. rubellus	PV121635	Germany, Schleswig-Holstein	B 70 0132470	orellanine, phalloidin, phalloin
C. splendens	AY174833	Germany, Brandenburg, nearby Gransee	B 70 0132471	--
C. alcalinophilus	MK010931	Germany, Brandenburg, nearby Rüdersdorf	B 70 0132472	--
C. cinnabarinus	AY669662	Germany, Mecklemburg-Western Pomerania	B 70 0132473	--
C. subcotoneus	MW010106	Germany, Brandenburg, nearby Eberswalde	B 70 0132474	--
C. xanthophyllus	AY174827	Germany, Brandenburg, nearby Gransee	B 70 0132475	--
C. melanotus	MW010073	Germany, Brandenburg, nearby Neuruppin	B 70 0132476	--
C. traganus	DQ367900	Germany, Thuringia	B 70 0132477	--
C. humicola	KP866157	Germany, Brandenburg, nearby Prenzlau	B 70 0132478	--
C. infractus	KF732523	Germany, Brandenburg, nearby Rüdersdorf	B 70 0132479	--
C. subtortus	MK357371	Germany, Thuringia, nearby Neustadt am Rennsteig	B 70 0132480	--
C. camphoratus	MN751052	Germany, Thuringia, nearby Neustadt am Rennsteig	B 70 0132481	--

[1] Herbarium of the Botanical Garden and Botanical Museum Berlin-Dahlem (B), Berlin

2 Peptide synthesis protocol and characterization data

2.1 Reagents, solutions, chromatographic conditions, mass spectrometry and spectroscopic methods

Commercially available reagents (Carl Roth GmbH and Co. KG, Karlsruhe, Germany; Sigma-Aldrich Taufkirchen, Germany; Iris Biotech GmbH, Marktredwitz, Germany; Orpegen, Heidelberg, Germany; ABCR, Karlsruhe, Germany; Alfa Aesar, Karlsruhe, Germany; Merck, Darmstadt; Germany; TCI, Eschborn, Germany; VWR International GmbH, Darmstadt, Germany; and Acros, Geel, Belgien) and solvents (Fisher Scientific-Acros, Schwerte, Germany) were used without further purification. If necessary, reactions were carried out under an atmosphere of argon or nitrogen and dry solvents. Analytical thin layer chromatography was carried out using aluminium-backed plates coated with silica gel (60, F254; Macherey & Nagel, Düren, Germany). Analysis was performed by visualizing the spots under UV light (λ = 254 nm), and/or by staining with $KMnO_4$ solution (3.0 g $KMnO_4$, 20.0 g K_2CO_3, 300.0 mL dest. H_2O, 5.0 mL NaOH solution (5 %)) and/or with Ninhydrin solution (0.30 g ninhydrin, 3.0 mL AcOH, 100 mL nBuOH). Flash chromatography was carried out with silica gel (particle size 40-63 µm, VWR Chemicals, Darmstadt, Germany).

Analytical HPLC-HRMS spectra were recorded on a Orbitrap XL (Thermo Fisher Scientific, Waltham, Massachusetts, USA) hyphenated to an Agilent 1200 Series HPLC-System (Agilent Technologies, Waldbronn, Germany) equipped with a C18 column (50 x 2 mm, particle size 3.0 µm). HPLC-HRMS chromatograms were obtained with a solvent gradient of 0.1% formic acid in water (Solvent A) and 0.1% formic acid in acetonitrile (Solvent B). The solvent gradient A was: 0-10 min 20%-100% B, 10-12 min 100% B, 12-17 min 20%, Flow rate: 0.30 mL/minThe solvent gradient B was: 0-10 min 50%-100% B, 10-12 min 100% B, 12-17 min 20%, Flow rate: 0.30 mL/min. The solvent gradient C for Marfey's amino acid analysis was: 0-10 min 25%-75% B, 30 min 100% B, 12-17 min 50%, Flow rate: 0.30 mL/min. Xcalibur (Thermo Scientific) was used for the evaluation of the spectra.

Flash chromatography was performed with an automated flash chromatography Rf system (Biotage AB, Uppsala, Sweden) using a Biotage Sfär Bio C18 D column A (pore size: 300 Å, particle size: 20 µm, size: 25 g), and a Biotage Sfär Bio C18 D column B (pore size 300 Å, particle size: 30 µm, size: 60 g) for bicyclic peptide and amino acid purification respectively.

Preparative HPLC was carried out on a 1260 Infinity (Agilent Technologies, Waldbronn, Germany) using a polymeric reversed phase column Phenomenex Luna C18(2) (100 Å, 250 x 21.2 mm, 10 micron).

^{1}H-NMR and ^{13}C-NMR spectroscopy: Spectra were recorded at 298 K using the following spectrometers: Bruker Avance-II 400 MHz, Bruker Avance-III 500 MHz or Bruker Avance III 700 MHz (Bruker, Karlsruhe, Germany). The chemical shifts are reported in ppm using the residual solvent peak as an internal reference (DMSO-d_6, $CDCl_3$). Multiplicity (br. s = broad singlet, s = singlet, d = doublet, dd = doublet of doublet, t = triplet, q = quartet, m = multiplet) and coupling constants (J = Hz) are quoted where possible. **Variable temperature NMR (VT-NMR):** ^{1}H-NMR spectra were acquired from 298-333 K in 10 K increments in DMSO-d_6. **^{1}H, ^{1}H-TOCSY, ^{1}H, ^{1}H-COSY, ^{1}H, ^{1}H-NOESY and ^{1}H,^{13}C-HSQC spectra** were recorded on a Bruker Avance III 700 MHz or 500 MHz spectrometer with a TXI 3 or 5 mm probe. Standard Bruker pulse programs were used, and all spectra were acquired at 298 K. Residual solvent methyl peaks (DMSO-d_6: δ = 2.50 for ^{1}H and δ = 39.0 ppm for ^{13}C) were used for chemical shift referencing.

UV spectroscopy: Spectra were obtained from the UV-trace of HPLC-HRMS runs. Samples were prepared at concentrations of 0.85 mM (dissolved in water), and HPLC-UV chromatograms were obtained employing a solvent gradient for analytical HPLC (solvent gradient was: 0-10 min 20%-100% B, 10-12 min 100% B, 12-17 min 20%, Flow rate: 0.3 mL/min) with 0.1% formic acid in water (Solvent A) and 0.1% formic acid in acetonitrile (Solvent B).

CD spectroscopy was performed using a Jasco J-815 spectrometer (Jasco, Groß-Umstadt, Germany). Cuvette path length: 1.0 mm, data accumulation: 5 spectra, scan speed: 20 nm/min, data pitch: 0.1 nm, digital integration time: 2 s, wavelength: λ = 180-300 nm. Samples were prepared at concentrations of 0.85 mM (dissolved in water).

Abbreviations: 2-CTC, 2-chlorotrityl chloride (resin); CH_3CN, acetonitrile; DCM, dichloromethane; DIPEA, *N*,*N*'-diisopropylethylamine; DMF, *N*,*N*'-dimethylformamide; DBU, 1,8-diazabicyclo[5.4.0]undec-7-ene; DMAP, 4-dimethylaminopyridine; HFIP, 1,1,1,3,3,3-hexafluoroisopropanol; TFA, trifluoroacetic acid; THF, tetrahydrofuran; HATU, *O*-(7-azabenzotriazol-1-yl)-*N*,*N*,*N*',*N*'-tetramethyluronium-hexafluorophosphate; Hyp, *trans*-4-hydroxy-L-proline; Boc_2O, di-*tert*-butyl decarbonate; D, L-FDLA, 1-fluoro-2,4-dinitrophenyl-5-D,L-leucineamide. D-Thr = D-threonine; (4-OMe)Trp = H-L-Trp(4-OMe)-OH; (4-OH)Trp = H-L-Trp(4-OH)-OH; CorA = Cortinarin A; CorB = Cortinarin B; CorC = Cortinarin C; CorX = Cortinarin X; $CorX_P$ = *P*-ansamer of Cortinarin X; $CorX_M$ = *M*-ansamer of Cortinarin X. CorX-Ala_P = *P*-ansamer of cortinarin-Ala; CorX-Ala_M = *M*-ansamer of cortinarin-Ala; $CorA_P$ = *P*-ansamer of cortinarin A; $CorB_P$ = *P*-ansamer of cortinarin B.

2.2 Synthesis of tryptophan analogs

The synthesis of tryptophan analogs was performed according to literature protocols[4]:

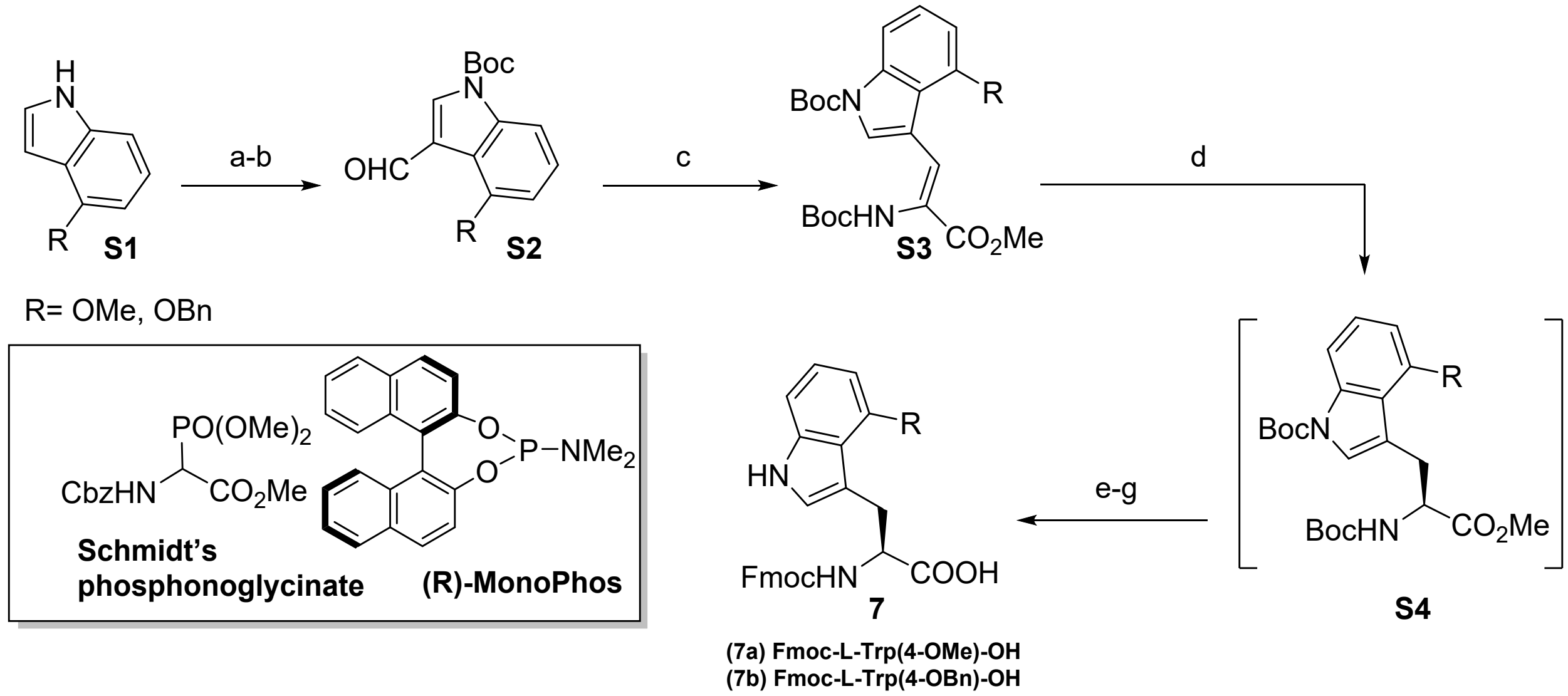

Scheme S1. Synthesis of tryptophan analogs. a) $POCl_3$, DMF, 0 °C; b) Boc_2O, DMAP, DCM, rt; c) Boc-2-phosphonoglycine-methyl dimethyl ester, DBU, DCM, rt; d) $[Rh(COD)_2]BF_4$, (*R*)-MonoPhos, H_2, DCM, rt; e) MeOH, THF, H_2O, LiOH, rt; f) TFA/DCM (1:1); g) Fmoc-OSu, sat. $NaHCO_3$, Dioxane/H_2O (1:1).

2.2.1 Experimental procedures for the synthesis of aldehyde S2

Phosphorous oxychloride (1.40 mL, 15.0 mmol, 1.50 equiv.) was added dropwise to dry DMF (5.00 mL) at 0 °C. At this temperature, the 4-OMe/Bn indole (10.0 mmol, 1.00 equiv.) in dry DMF (5.00 mL) was slowly added, whereby a bright-yellow precipitate was formed. The reaction mixture was heated to 45 °C and stirred for 2 h. The reaction was poured into ice water (200 mL) and kept stirring for 2 h. The aqueous layer was then treated with saturated NaOH-solution until the solution was basic and extracted with DCM (3 x 30.0 mL). The organic extracts were washed with brine, dried (Na_2SO_4) and concentrated under reduced pressure to give the crude product aldehyde without further purification. The crude product was dissolved in DCM (50.0 mL) and treated at room temperature with DMAP (122 mg,1.00 mmol, 0.10 equiv.) and Boc_2O (2.62 g, 12.0 mmol, 1.2 equiv.). After stirring for 1 h, 50.0 mL of DCM was added, and the organic layer was washed by 1 M HCl (3 x 30 mL) and brine (30 mL). Then, the organic phase was dried (Na_2SO_4) and concentrated under reduced pressure. The crude product was purified by flash chromatography (ethyl acetate/hexane, 1:10) to give solid.

***tert*-butyl 3-formyl-4-OMe-1*H*-indole-1-carboxylate (S2a)**

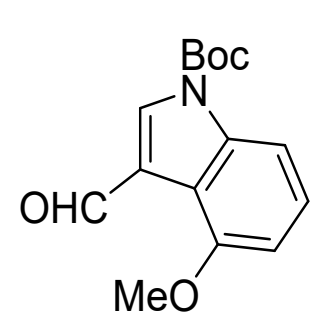

S2a

Following the general synthesis procedure, **S2a** was isolated as a pink solid (60.0%, 1.65 g).

^{1}H-NMR (500 MHz, $CDCl_3$) δ 10.57 (s, 1H), 8.25 (s, 1H), 7.34 (t, *J* = 8.2 Hz, 1H), 6.83 (d, *J* = 8.1 Hz, 1H), 4.02 (s, 3H), 1.70 (s, 7H).

^{13}C-NMR (126 MHz, $CDCl_3$) δ 189.09, 154.10, 148.99, 137.17, 128.71, 126.18, 121.38, 117.21, 108.63, 104.55, 85.43, 55.52, 28.07.

HRMS (ESI): m/z calculated $C_{15}H_{18}NO_4^+$ $[M+H]^+$ 276.1230, found 276.1232.

***tert*-butyl 3-formyl-4-OBn-1*H*-indole-1-carboxylate (S2b)**

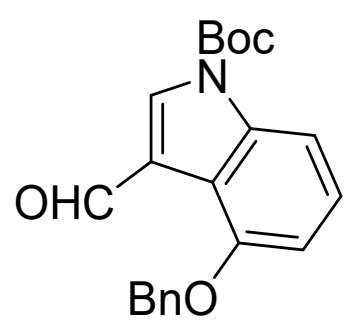

S2b

Following the general synthesis procedure, **S2b** was isolated as a white or pale-yellow solid (57.8%, 2.03 g).

^{1}H-NMR (500 MHz, $CDCl_3$) δ 10.46 (s, 1H), 8.16 (s, 1H), 7.79 (d, *J* = 8.4 Hz, 1H), 7.38 (d, *J* = 7.2 Hz, 3H), 7.32 (t, *J* = 7.2 Hz, 1H), 7.27 (d, *J* = 7.2 Hz, 1H), 7.24 – 7.17 (m, 1H), 6.81 (d, *J* = 8.1 Hz, 1H), 5.17 (s, 3H), 1.60 (s, 8H).

^{13}C-NMR (126 MHz, $CDCl_3$) δ 189.02, 153.12, 148.93, 146.76, 137.28, 136.43, 128.73, 128.21, 127.58, 126.12, 121.32, 117.41, 108.88, 105.79, 85.16, 70.48, 28.03.

HRMS (ESI): m/z calculated $C_{21}H_{22}NO_4^+$ $[M+H]^+$ 352.1543, found 352.1544.

2.2.2 Experimental procedures for the synthesis of 2,3-didehydro amino acid S3

To a solution of Boc-α-phosphonoglycine-methyl dimethyl ester (2.70 g, 9.10 mmol, 1.30 equiv.) in DCM (10.0 mL) was added DBU (1.15 mL, 7.70 mmol, 1.10 equiv.). After stirring for 10 min, aldehyde **S2** (7.00 mmol, 1.00 equiv.) in DCM (10.0 mL) was added slowly and the reaction mixture stirred for further 16 h. Subsequently, the solvent was evaporated under reduced pressure. The residue was dissolved in ethyl acetate (40.0 mL). Then the organic solution was washed with 1 M HCl (2 x 10.0 mL) and brine, dried ($NaSO_4$), and concentrated. The crude product was purified by flash chromatography (ethyl acetate/hexane, 1:5) to give solid.

***tert*-butyl (*Z*)-3-(2-((*tert*-butoxycarbonyl)amino)-3-methoxy-3-oxoprop-1-en-1-yl)-4-methoxy-1*H*-indole-1-carboxylate (S3a)**

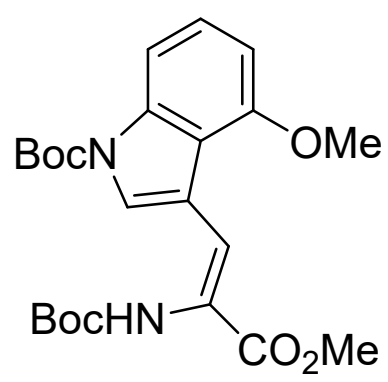

S3a

Following the general synthesis procedure, **S3a** was isolated as a yellow solid (73.1%, 2.28 g).

^{1}H-NMR (500 MHz, $CDCl_3$) δ 8.16 (s, 1H), 7.85 (s, 1H), 7.71 (d, *J* = 8.2 Hz, 1H), 7.22 – 7.00 (m, 1H), 6.64 (d, *J* = 8.1 Hz, 1H), 3.88 (s, 3H), 3.79 (s, 4H), 1.59 (s, 8H), 1.41 (s, 9H).

^{13}C-NMR (126 MHz, $CDCl_3$) δ 166.22, 154.58, 149.24, 136.33, 126.15, 125.72, 118.79, 114.42, 108.29, 104.17, 84.44, 80.73, 55.48, 52.39, 28.29, 28.12.

HRMS (ESI): m/z calculated $C_{23}H_{31}N_2O_7^+$ [M+H]$^+$ 447.2126, found 447.2125.

***tert*-butyl (*Z*)-4-(benzyloxy)-3-(2-((*tert*-butoxycarbonyl)amino)-3-methoxy-3-oxoprop-1-en-1-yl)-1*H*-indole-1-carboxylate (S3b)**

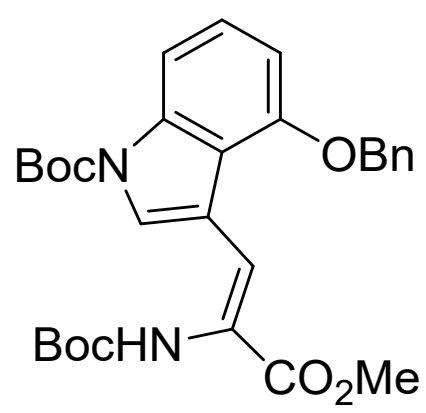

S3b

Following the general synthesis procedure, **S3b** was isolated as a yellow solid (80.2%, 2.93 g).

^{1}H-NMR (500 MHz, $CDCl_3$) δ 8.33 (s, 1H), 7.96 (s, 1H), 7.84 (d, *J* = 8.2 Hz, 1H), 7.55 (d, *J* = 7.9 Hz, 1H), 7.44 (t, *J* = 7.3 Hz, 2H), 7.39 (d, *J* = 7.5 Hz, 1H), 7.31 – 7.23 (m, 1H), 6.85 (d, *J* = 8.1 Hz, 1H), 5.22 (s, 3H), 3.67 (s, 3H), 1.70 (s, 8H), 1.51 (s, 6H).

^{13}C-NMR (126 MHz, $CDCl_3$) δ 166.15, 153.67, 149.25, 136.67, 136.45, 128.52, 127.89, 127.74, 126.38, 126.22, 125.68, 118.98, 114.41, 108.64, 105.36, 84.49, 80.73, 70.42, 52.25, 28.31, 28.14.

HRMS (ESI): m/z calculated $C_{29}H_{35}N_2O_7^+$ [M+H]$^+$ 523.2439, found 523.2432.

2.2.3 Experimental procedures for the synthesis of Fmoc-L-Trp(4-OMe/Bn)-OH (7a and 7b)

To a solution of **S3** (1.04 g, 2.00 mmol, 1 equiv.) in dry, degassed DCM (10.0 mL) was added a solution of [Rh(COD)$_2$]BF_4 (16.2 mg, 40.0 µmol, 0.020 equiv.) and (*R*)-MonoPhos (28.7 mg, 80.0 µmol, 0.0400 equiv.) in dry, degassed DCM (10.0 mL) under argon atmosphere. The resulting mixture was placed in an autoclave and argon was replaced by H_2 (20.0 bar). After stirring for 12 h at room temperature, TLC showed complete conversion, and the solvent was removed under reduced pressure. Crude product **S4** was used in the next step without further purification.

MeOH (4.00 mL), THF (4.00 mL), H_2O (4.00 mL) and LiOH (239 mg, 10.0 mmol) were added to the crude product at 0 °C. The mixture was allowed to warm up to room temperature for 2 h and the solvent was removed in vacuo after TLC showed full consumption of the starting material. The residue was dissolved in water and washed with DCM. Then, the aqueous layer was acidified, using 1.00 M aq. $KHSO_4$, followed by extraction with DCM (3 x 30.0 mL). The resulting organic layers were combined, dried (Na_2SO_4) and the solvent was removed. The resulting product was dissolved in DCM (15.0 mL) at 0 °C followed by adding trifluoroacetic acid (15.0 mL). The reaction mixture was stirred for 1 h at room temperature. The solution was concentrated, and the crude product was dissolved directly in $NaHCO_3$ aqueous solution (15.0 mL). Then Fmoc-OSu (2.09 mmol, 1.02 equiv.) in dioxane (15.0 mL) was added to the solution in the 0°C. The resulting solution was stirred at room temperature for 5 h and then concentrated under reduced pressure, diluted with H_2O (20.0 mL), acidified to pH= 4 with 1 M HCl solution, and extracted with EtOAc (2

x 40.0 mL). The organic layers were combined and washed sequentially with H_2O (2 x 40.0 mL) and brine (40.0 mL), dried over anhydrous Na_2SO_4, filtered and concentrated. The crude product was purified by C18 reversed phase automated flash chromatography (Biotage Sfär Bio C18 D column B; gradient 30-100% CH_3CN/H_2O in 13.60 min; flow rate of 50.0 mL/min; retention time: 6.80-8.50 min) to afford **7a** and **7b**, respectively.

Fmoc-L-Trp(4-OMe)-OH (7a)

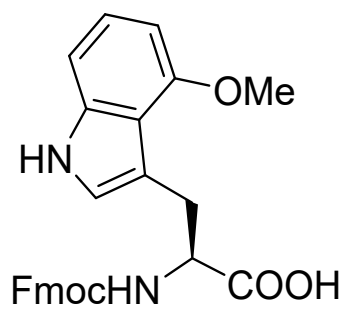

7a

Following the general synthesis procedure, **7a** was isolated as a grey solid (71.3%, 0.650 g).

^{1}H-NMR (500 MHz, DMSO-d_6) δ 12.50 (s, 1H), 10.82 (s, 1H), 7.88 (d, J = 7.9 Hz, 2H), 7.66 (d, J = 7.5 Hz, 1H), 7.62 (d, J = 7.5 Hz, 1H), 7.56 (d, J = 7.9 Hz, 1H), 7.41 (q, J = 8.0 Hz, 2H), 7.32 (t, J = 7.5 Hz, 1H), 7.26 (t, J = 7.5 Hz, 1H), 7.03 (s, 1H), 7.00 – 6.92 (m, 2H), 6.47 (d, J = 7.3 Hz, 1H), 4.34 – 4.27 (m, 1H), 4.19 (d, J = 4.6 Hz, 2H), 3.84 (s, 3H), 3.42 – 3.30 (m, 2H), 3.01 (dd, J = 14.3, 10.3 Hz, 1H).

^{13}C-NMR (126 MHz, DMSO-d_6) δ 174.54, 156.36, 154.39, 144.28, 144.23, 141.14, 138.36, 128.08, 127.54, 125.74, 125.69, 123.15, 122.28, 120.54, 117.26, 110.75, 105.46, 99.34, 66.01, 55.99, 55.45, 47.06, 28.92.

HRMS (ESI): m/z calculated $C_{27}H_{25}N_2O_5^+$ [M+H]$^+$ 457.1758, found 457.1747.

Fmoc-L-Trp(4-OBn)-OH (7b)

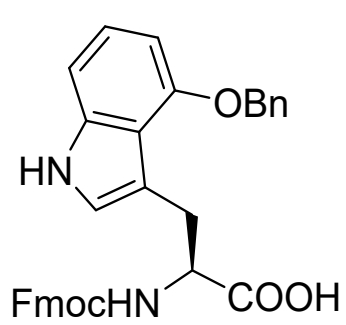

7b

Following the general synthesis procedure, **7b** was isolated as a grey solid (73.3%, 0.780 g).

^{1}H-NMR (500 MHz, DMSO-d_6) δ 12.46 (s, 1H), 10.85 (s, 1H), 7.89 (d, J = 7.6 Hz, 3H), 7.70 – 7.56 (m, 2H), 7.54 (d, J = 7.5 Hz, 3H), 7.41 (q, J = 7.1 Hz, 3H), 7.38 – 7.30 (m, 2H), 7.30 – 7.21 (m, 1H), 7.03 (s, 1H), 6.96 (d, J = 4.6 Hz, 2H), 6.56 (t, J = 4.3 Hz, 1H), 5.22 (s, 2H), 4.42 – 4.32 (m, 1H), 4.27 – 4.10 (m, 2H), 3.43 (dd, J = 14.3, 5.1 Hz, 1H), 3.11 (dd, J = 14.3, 10.0 Hz, 1H).

^{13}C-NMR (126 MHz, DMSO-d_6) δ 174.44, 156.33, 153.28, 144.30, 141.15, 138.54, 137.94, 128.78, 128.08, 127.90, 127.60, 127.55, 125.74, 123.24, 122.17, 120.55, 117.42, 110.67, 105.60, 100.67, 69.44, 66.05, 55.94, 47.07, 28.88.

HRMS (ESI): m/z calculated $C_{33}H_{29}N_2O_5^+$ [M+H]$^+$ 533.2071, found 533.2067.

2.3 General protocols for the synthesis of cortinarins

2.3.1 Resin loading and peptide assembly

2-CTC resin (1.00 g, 1.60 mmol/g) was pre-swollen for 30 min with DCM in a syringe (20.0 mL). After the solvent was drained, Fmoc-Gly-OH (149 mg, 0.50 mmol, 1.00 equiv.) and DIPEA (1.00 mL, 5.74 mmol, 9.00 equiv.) in DCM (15.0 mL) were added to the syringe. The mixture was agitated for 2 h and then the solvent was drained. The resin was rinsed with DMF (5 x 10.0 mL). Then a mixture of MeOH/DIPEA/DCM (1:1:8) was added to cap the remaining 2-chlorotrityl chloride on the resin. The mixture was agitated for 30 min. Then the solvent was drained, and the resin was washed with DMF (5 x 10.0 mL). The resin loading was determined to be 0.50 mmol/g following a literature protocol.[2] For the sequent amino acid couplings, the following procedure were used:

Method A: Removal of the Fmoc group. A solution of 20% piperidine in DMF (5.00 mL) was added to the resin (1.00 g; loading 0.50 mmol/g) and the resulting suspension was shaken for 10 min. Then the solution was removed from the resin. Again, a solution of 20% piperidine in DMF (5.00 mL) was added to the resin and the resulting suspension was shaken for another 10 min. The solution was drained and the resin was washed with DMF (6 x 5.00 mL).

Method B: Amino acid coupling. Protected Fmoc-amino acid (4.00 equiv.) and HATU (4.00 equiv.) were dissolved in dry DMF (5.00 mL). DIPEA (12.00 equiv.) was added dropwise to the DMF solution. After activating for 1 min, the resulting solution was added to the Fmoc-deprotected 2-CTC resin (1.00 g; loading 0.50 mmol/g). The mixture was shaken until the coupling reaction was completed (Method C). Then, the solution was drained and the resin was rinsed with DMF (4 x 5.00 mL).

Method C: Monitoring of peptide coupling (Kaiser Test). During the coupling reaction, a few resin beads were taken out and rinsed with DMF (2 x 1.00 mL). To the resin were added 2-3 drops of reagent A (Preparation: 16.5 mg of KCN dissolved in 25.0 mL of distilled water. Above solution (1.00 ml) was diluted with 49.0 mL of pyridine), 2 to 3 drops of reagent B (ninhydrin (1.00 g) dissolved in 20.0 mL n-butanol) and 2 to 3 drops of reagent C (40.00 g phenol dissolved in 20.0 mL n-butanol). The resulting suspension was allowed to heat at 110 °C for 3 min; blue- to green-stained beads indicated the presence of primary amine.

2.3.2 Protocol for the regeneration of decapeptides from peptide aggregates

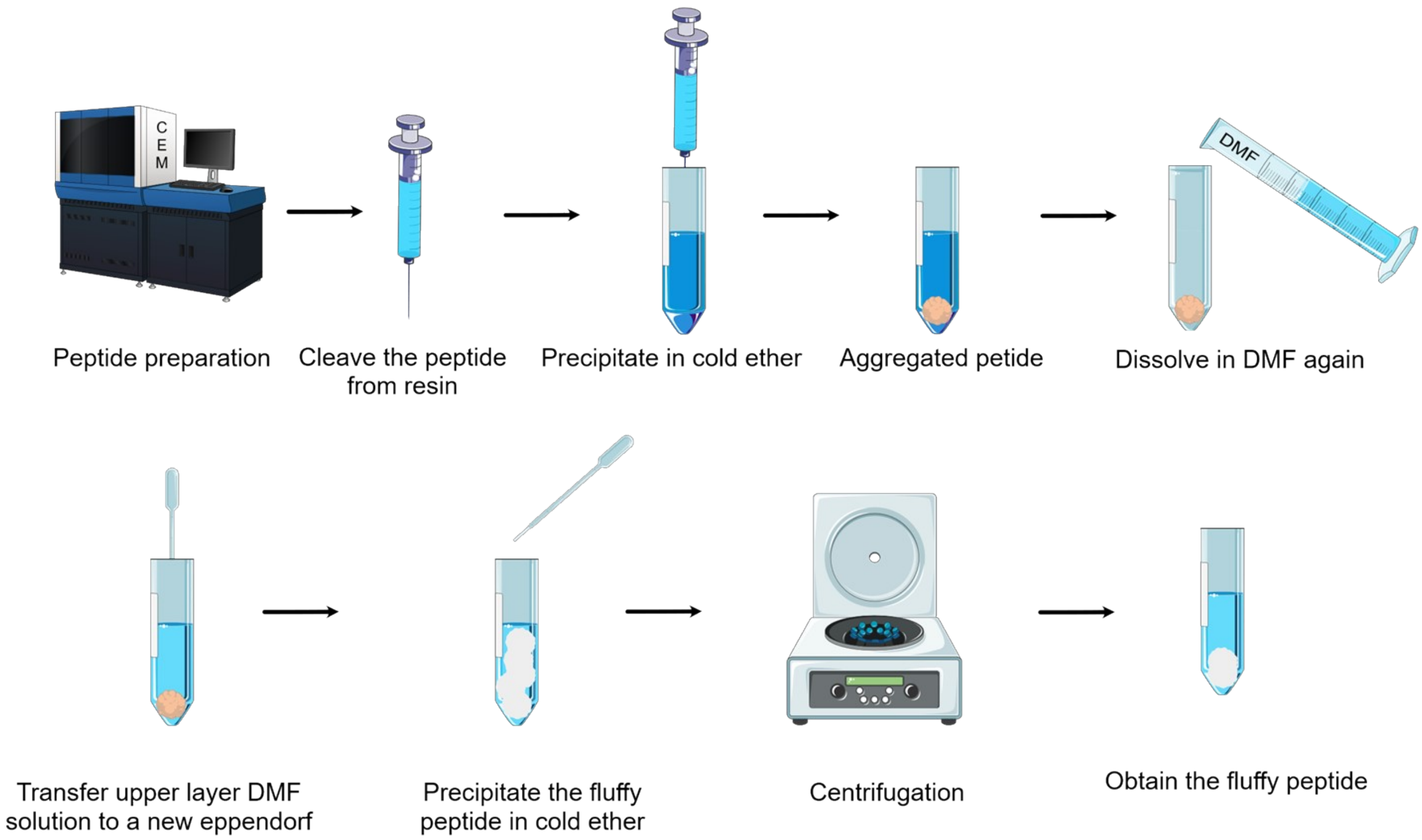

Figure S1. Workflow for the regeneration of cleaved decapeptide from ether-precipitates.

After the addition of cold diethyl ether, precipitation of decapeptides (**S8**, **S11**, **S13**, **S16 and S19**) was observed. The peptides were solubilized by applying the following protocol:

Cold ether supernatant was removed and then 5.00 mL DMF solution (to 100.0 mg peptide) were added under vigorous shaking and centrifugation. The clear DMF supernatant solution was pipetted to a new centrifuge tube with cold diethyl ether (40.0 mL) to induce precipitation. This DMF solubilization -precipitation cycle was repeated until no more aggregated peptide could be dissolved in DMF, and no more peptide precipitated from cold ether. Following centrifugation, the ether was decanted, and samples were dried in the lyophilizer. Finally, crude monocyclic decapeptides were obtained.

2.3.3 Removal of protecting groups

Boc deprotection: Protected Cortinarin (5.00 mg) was dissolved in TFA solution (1.00 mL, TFA/DCM, 3:7) and kept stirring for 1 h. Afterwards, the reaction mixture was evaporated under reduced pressure, and the crude product was purified by preparative HPLC to give the final product.

Debenzylation: Protected Cortinarin was dissolved in EtSH (10.0 mg/mL, cortinarin-to-reagent ratio) and flushed with N_2 followed by addition of BF_3*Et_2O (10.0 equiv.) and was stirred for 30 min. Afterwards, the reaction mixture was evaporated under reduced pressure, and the crude product was purified by preparative HPLC to give the final product.

2.4 Synthesis of cortinarin A (1a)

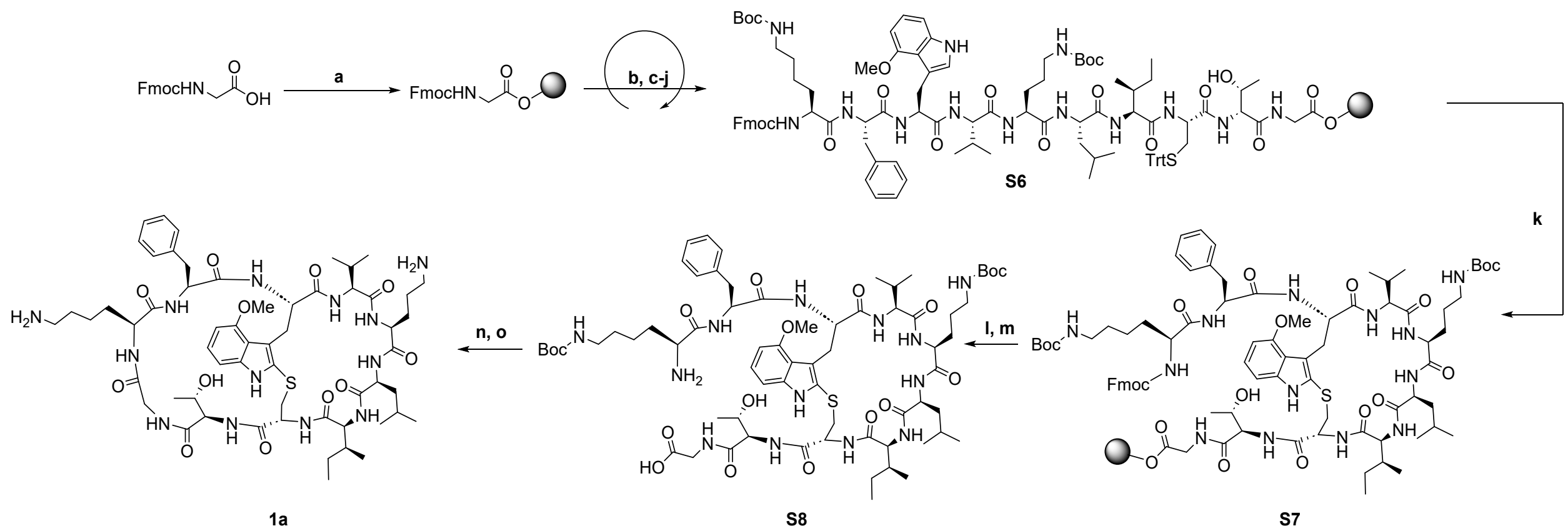

Scheme S2. Synthesis of cortinarin A (1a). a) 2-CTC resin, DIPEA, DCM, 2 h; b) Piperidine/DMF (1:4); c) Fmoc-D-Thr-OH, HATU, DIPEA, DMF; d) Fmoc-L-Cys(Trt)-OH, HATU, DIPEA, DMF; e) Fmoc-L-Ile-OH, HATU, DIPEA, DMF; f) Fmoc-L-Leu-OH, HATU, DIPEA, DMF; g) Fmoc-L-Orn(Boc)-OH, HATU, DIPEA, DMF; h) Fmoc-L-Trp(4-OMe)-OH, HATU, DIPEA, DMF; i) Fmoc-L-Phe-OH, HATU, DIPEA, DMF; j) Fmoc-L-Lys(Boc)-OH, HATU, DIPEA, DMF; k) 2.00 mg/mL I_2 in DMF, 3 h; l) Piperidine/DMF (1:4); m) HFIP/DCM (1:4); n) HATU (2.00 equiv.), DIPEA (5.00 equiv.), DMF (peptide concentration, 2.00 μM), 12 h; o) TFA/DCM (3:7), 1h.

2.4.1 Preparation of resin-bound linear peptide S6

Following the above-described protocol (**subsection 2.3.1**), Fmoc-Gly-OH (149 mg, 0.50 mmol, 1.00 equiv.) was loaded on 2-CTC resin. For the subsequent amino acid couplings, the following procedures were used:

A sequence of alternating Fmoc-deprotections (Method A) and amino acid couplings (Method B) was performed for the following amino acids: Fmoc-D-Thr-OH (341 mg, 1.00 mmol, 2.00 equiv.), Fmoc-L-Cys(Trt)-OH (586 mg, 1.00 mmol, 2.00 equiv.), Fmoc-L-Ile-OH (353 mg, 1.00 mmol, 2.00 equiv.), Fmoc-L-Leu-OH (353 mg, 1.00 mmol, 2.00 equiv.), Fmoc-L-Orn(Boc)-OH (455 mg, 1.00 mmol, 2.00 equiv.), Fmoc-L-Val-OH (339 mg, 1.00 mmol, 2.00 equiv.), Fmoc-L-Trp(4-OMe)-OH (457 mg, 1.00 mmol, 2.00 equiv.), Fmoc-L-Phe-OH (387 mg, 1.00 mmol, 2.00 equiv.), and Fmoc-L-Lys(Boc)-OH (469 mg, 1.00 mmol, 2.00 equiv.) until the sequence of the peptide **S6** was completed.

2.4.2 Preparation of monocyclic decapeptide S8

A freshly prepared solution of iodine in DMF (2.00 mg/mL) was added to the resin of linear peptide **S6**. The mixture was agitated for 3 h, to form monocyclic decapeptide **S7**. After the reaction was completed (HPLC control), the solution was drained, and the resin was washed with DMF (5 x 10.0 mL).

Subsequently, the Fmoc group was removed using Method A. Once the Fmoc deprotection was completed (HPLC control), the resin was washed with DMF (5 x 10.0 mL). Subsequently, the resin was treated with 10.0 mL of a mixture of HFIP/DCM (1:4) for 1 h at room temperature with gentle agitation. After cleavage, the beads were filtered and rinsed with DCM (2 x 10.0 mL). The filtrates were combined and evaporated at room temperature almost to dryness. Then, the aggregated

peptide **S8** was precipitated in cold diethyl ether and regenerated from aggregates, following the general protocol of **subsection 2.3.2**.

2.4.3 Macrolactamization and deprotection to cortinarin A (1a)

To a solution of monocyclic decapeptide **S8** (1.00 equiv.) in solution of DIPEA (5.00 equiv.) in DMF (peptide concentration, 2.00 μM), HATU (2.00 equiv.) was added at room temperature. The solution was stirred for 12 h, then purified by preparative reversed phase automated flash chromatography (Biotage Sfär Bio C18 D column A; gradient 30-100% CH_3CN/H_2O in 10 min; flow rate of 40.0 mL/min; retention time: 4.20-6.30 min). The isolated product was lyophilized to give a crude white solid (35 mg).

The white solid (35.0 mg, 0.025 mmol) was dissolved using a cleavage solution (5.00 mL, TFA/DCM, 3:7) and was stirred for 1 h. Then the mixture was concentrated by rotatory evaporator and purified by preparative HPLC. Subsequently, the fractions eluting between 8.70-9.20 min were unified and lyophilized in a 100 mL flask. Cortinarin A was isolated as a white solid (11.0 mg, total yield 1.80 %).

Cortinarin A (1a)

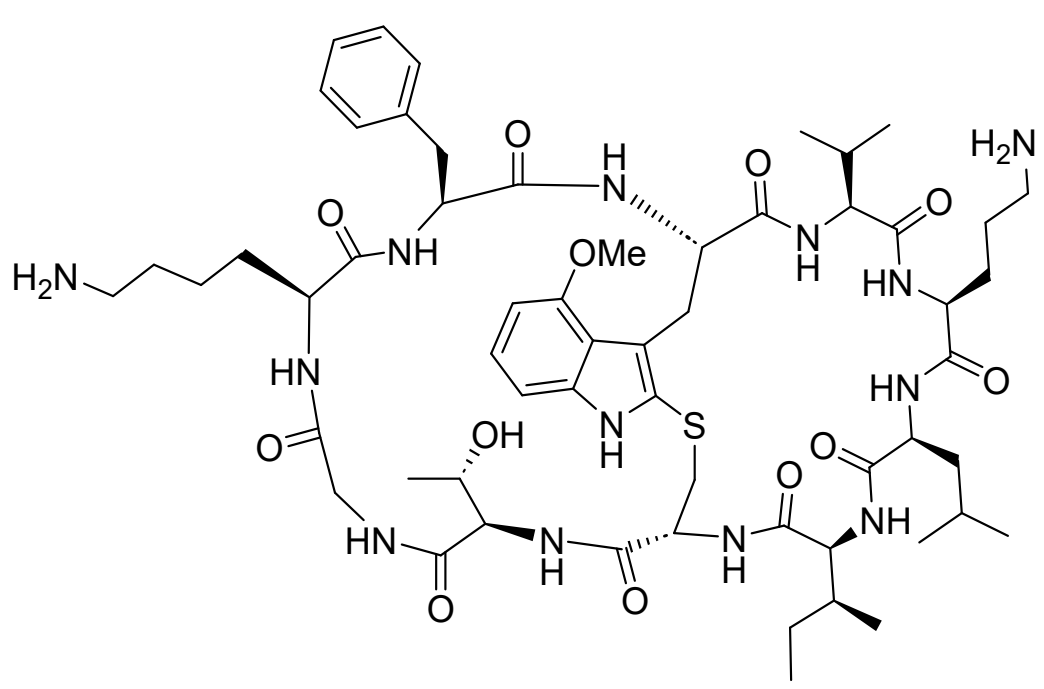

HRMS (ESI): m/z calculated $C_{58}H_{88}N_{13}O_{12}S^{+}$ $[M+H]^{+}$ 1190.6391, found 1190.6368.

Analytical HPLC-MS: retention time R_t = 5.62 min

Preparative HPLC gradient:

Time (min)	Water (%)	Acetonitrile (%)
0	95	5
1	95	5
15	0	100

Waters C18 Column, flow rate (30 min/mL)

2.5 Synthesis of cortinarin B (1b)

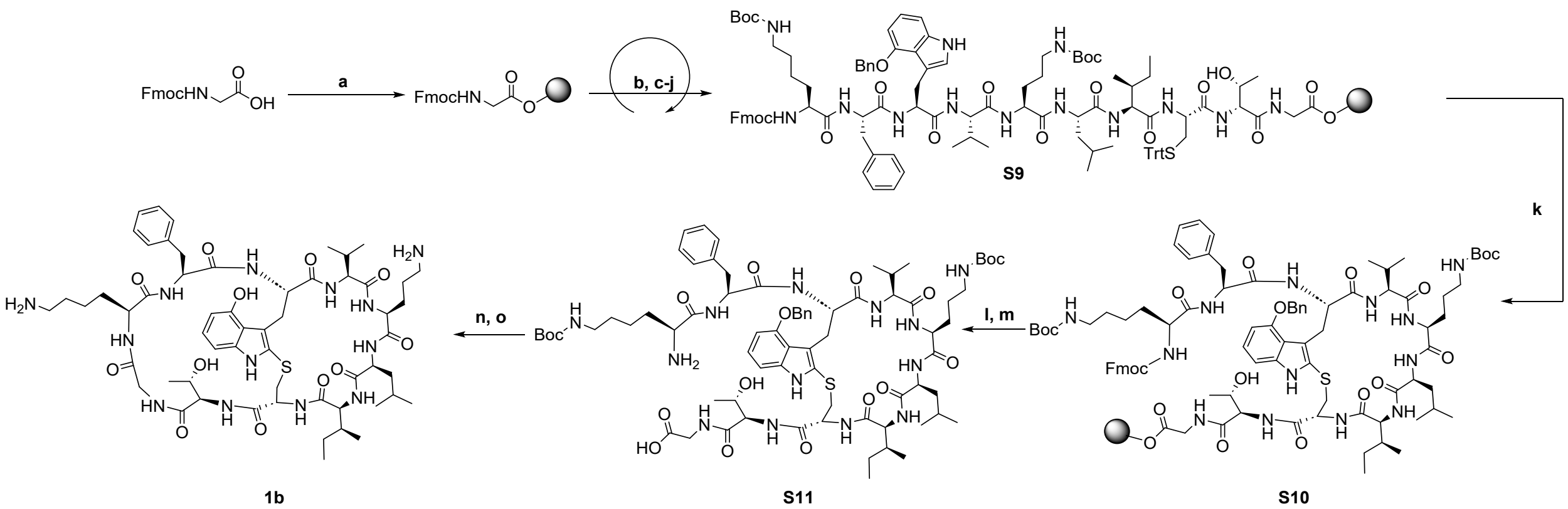

Scheme S3. Synthesis of cortinarins B (1b). a) 2-CTC resin, DIPEA, DCM, 2 h; b) Piperidine/DMF (1:4); c) Fmoc-D-Thr-OH, HATU, DIPEA, DMF; d) Fmoc-L-Cys(Trt)-OH, HATU, DIPEA, DMF; e) Fmoc-L-Ile-OH, HATU, DIPEA, DMF; f) Fmoc-L-Leu-OH, HATU, DIPEA, DMF; g) Fmoc-L-Orn(Boc)-OH, HATU, DIPEA, DMF; h) Fmoc-L-Trp(4-OBn)-OH, HATU, DIPEA, DMF; i) Fmoc-L-Phe-OH, HATU, DIPEA, DMF; j) Fmoc-L-Lys(Boc)-OH, HATU, DIPEA, DMF; k) 2.00 mg/mL I_2 in DMF, 3 h; l) Piperidine/DMF (1:4); m) HFIP/DCM (1:4); n) HATU (2.00 equiv.), DIPEA (5.00 equiv.), DMF (peptide concentration, 2.00 µM), 12 h; o) BF_3*Et_2O in EtSH, 1h.

2.5.1 Preparation of resin-bound linear peptide S9

Following the above-described protocol (**subsection 2.3.1**), Fmoc-Gly-OH (149 mg, 0.50 mmol, 1.00 equiv.) was loaded on 2-CTC resin. For subsequent amino acid couplings, the following procedures were used:

A sequence of alternating Fmoc-deprotections (Method A) and amino acid couplings (Method B) was performed for the following amino acids: Fmoc-D-Thr-OH (341 mg, 1.00 mmol, 2.00 equiv.), Fmoc-L-Cys(Trt)-OH (586 mg, 1.00 mmol, 2.00 equiv.), Fmoc-L-Ile-OH (353 mg, 1.00 mmol, 2.00 equiv.), Fmoc-L-Leu-OH (353 mg, 1.00 mmol, 2.00 equiv.), Fmoc-L-Orn(Boc)-OH (455 mg, 1.00 mmol, 2.00 equiv.), Fmoc-L-Val-OH (339 mg, 1.00 mmol, 2.00 equiv.), Fmoc-L-Trp(4-OBn)-OH (532 mg, 1.00 mmol, 2.00 equiv.), Fmoc-L-Phe-OH (387 mg, 1.00 mmol, 2.00 equiv.), and Fmoc-L-Lys(Boc)-OH (469 mg, 1.00 mmol, 2.00 equiv.) until the sequence of the peptide **S9** was completed.

2.5.2 Preparation of monocyclic decapeptide S11

A freshly prepared solution of iodine in DMF (2.00 mg/mL) was added to the resin of linear peptide **S9**. The mixture was agitated for 3 h, to form monocyclic decapeptide **S10**. After the reaction was completed (HPLC control) the solution was drained, and the resin was washed with DMF (5 x 10.0 mL).

The Fmoc group was removed using Method A. Once the Fmoc deprotection was completed (HPLC control), the resin was washed with DMF (5 x 10.0 mL). Subsequently, the resin was treated with 10.0 mL of a mixture of HFIP/DCM (1:4) for 1 h at room temperature with gentle agitation. After cleavage, the beads were filtered and rinsed with DCM (2 x 10.0 mL). The filtrates were combined and evaporated at room temperature almost to dryness. Then, the aggregated

peptide **S11** were precipitated in cold diethyl ether and regenerated from aggregates, following the general method of **subsection 2.3.2**.

2.5.3 Macrolactamization and deprotection to cortinarin B (1b)

To a solution of monocyclic decapeptide **S11** (1.00 equiv.) in solution of DIPEA (5.00 equiv.) in DMF (peptide concentration, 2.00 μM), HATU (2.00 equiv.) was added at room temperature. The solution was stirred for 12 h, then purified by preparative reversed phase automated flash chromatography (Biotage Sfär Bio C18 D column A; gradient 30-100% CH_3CN/H_2O in 10 min; flow rate of 40.0 mL/min; retention time: 2.80-5.30 min). The isolated product was lyophilized to give a crude white solid (20 mg).

The white solid (20.0 mg, 0.015 mmol) was dissolved in EtSH (10.0 mg/mL, sample-to-solvent ratio). Following, $BF_{3*}Et_2O$ (0.15 mmol, 10.0 equiv.) was added. The mixture was stirred for 30 min. Then the mixture was concentrated at the rotatory evaporator and purified by preparative HPLC. Subsequently, HPLC fractions eluting between 8.00-8.60 min were lyophilized. Cortinarin B was isolated as a white solid (0.5 mg, total yield 0.1%).

Cortinarin B (1b)

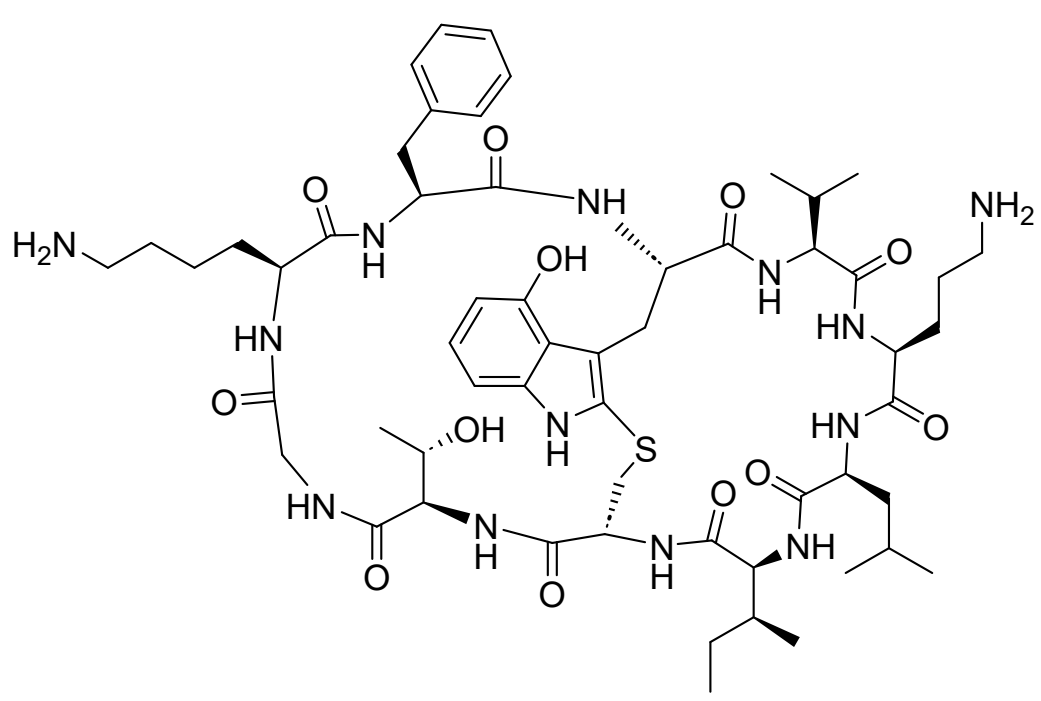

HRMS (ESI): m/z calculated $C_{57}H_{86}N_{13}O_{12}S^+$ $[M+H]^+$ 1176.6234, found 1176.6224.

Analytical HPLC-MS: retention time R_t = 4.62 min

Preparative HPLC gradient:

Time (min)	Water (%)	Acetonitrile (%)
0	95	5
1	95	5
15	0	100

Waters C18 Column, flow rate (30 min/mL)

2.6 Synthesis of cortinarin C (2)

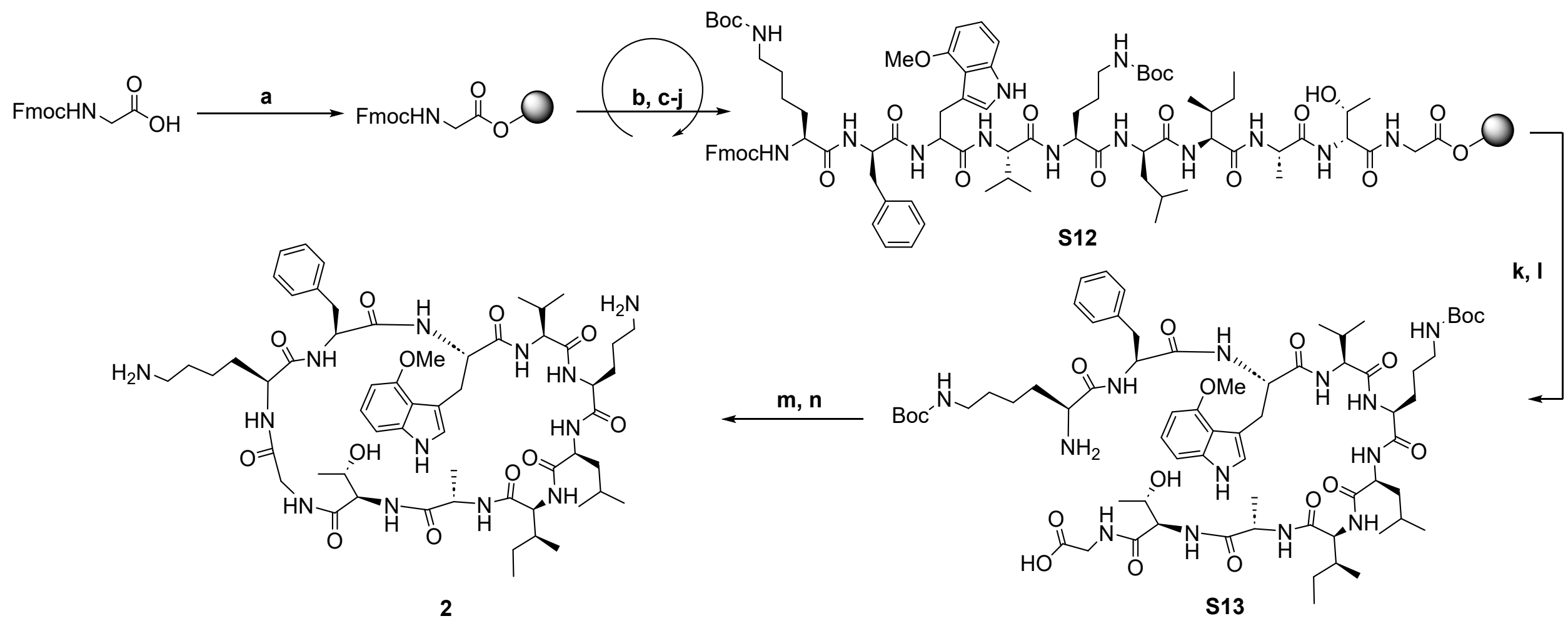

Scheme S4. Synthesis of monocyclic cortinarin C (2). a) 2-CTC, DIPEA, DCM, 2 h; b) Piperidine/DMF (1:4); c) Fmoc-D-Thr-OH, HATU, DIPEA, DMF; d) Fmoc-L-Ala-OH, HATU, DIPEA, DMF; e) Fmoc-L-Ile-OH, HATU, DIPEA, DMF; f) Fmoc-L-Leu-OH, HATU, DIPEA, DMF; g) Fmoc-L-Orn(Boc)-OH, HATU, DIPEA, DMF; h) Fmoc-L-Trp(4-OMe)-OH, HATU, DIPEA, DMF; i) Fmoc-L-Phe-OH, HATU, DIPEA, DMF; j) Fmoc-L-Lys(Boc)-OH, HATU, DIPEA, DMF; k) Piperidine/DMF (1:4); l) HFIP/DCM (1:4); m) HATU (2.00 equiv.), DIPEA (5.00 equiv.), DMF (peptide concentration, 2.00 µM), 12 h; n) TFA/DCM (3:7), 1 h.

2.6.1 Preparation of resin-bound linear peptide S12

Following the above protocol (**subsection 2.3.1**), Fmoc-Gly-OH (149 mg, 0.50 mmol, 1.00 equiv.) was loaded on 2-CTC resin. For the subsequent amino acid couplings, the following procedures were used:

A sequence of alternating Fmoc-deprotections (Method A) and amino acid couplings (Method B) was performed for the following amino acids: Fmoc-D-Thr-OH (341 mg, 1.00 mmol, 2.00 equiv.), Fmoc-L-Ala-OH (311 mg, 1.00 mmol, 2.00 equiv.), Fmoc-L-Ile-OH (353 mg, 1.00 mmol, 2.00 equiv.), Fmoc-L-Leu-OH (353 mg, 1.0 0mmol, 2.00 equiv.), Fmoc-L-Orn(Boc)-OH (455 mg, 1.00 mmol, 2.00 equiv.), Fmoc-L-Val-OH (339 mg, 1.00 mmol, 2.00 equiv.), Fmoc-L-Trp(4-OMe)-OH (457 mg, 1.00 mmol, 2.00 equiv.), Fmoc-L-Phe-OH (387 mg, 1.00 mmol, 2.00 equiv.), and Fmoc-L-Lys(Boc)-OH (469 mg, 1.00 mmol, 2.00 equiv.) until the sequence of the peptide **S12** was completed.

2.6.2 Preparation of linear peptide S13

The Fmoc group of linear peptide **S12** was removed using Method A. Once the Fmoc deprotection was completed, determined by HPLC, the resin was treated with 10.0 mL of a mixture of HFIP/DCM (1:4) for 1 h at room temperature with gentle agitation. After cleavage, the beads were filtered and rinsed with DCM (2 x 10.0 mL). The filtrates were combined and concentrated. Then, the aggregated yellow peptide **S13** was precipitated in the cold diethyl ether and regenerated from aggregates, following the general protocol of **subsection 2.3.2**.

2.6.3 Macrolactamization and deprotection of protected cortinarin C

To a solution of monocyclic decapeptide **S13** (1.00 equiv.) in solution of DIPEA (5.00 equiv.) in DMF (peptide concentration, 2.00 μM), HATU (2.00 equiv.) was added at room temperature. The solution was stirred for 12 h, then purified by preparative reversed phase automated flash chromatography (Biotage Sfär Bio C18 D column A; gradient 30-100% CH_3CN/H_2O in 10 min; flow rate of 40.0 mL/min; retention time: 4.20-6.30 min). The isolated product was lyophilized to give a crude white solid (60.0 mg).

The white solid (30.0 mg, 0.0200 mmol) was dissolved using a TFA buffer (5.00 mL, TFA/DCM, 3:7) and was stirred for 1 h. Then the mixture was concentrated by rotatory evaporator and purified by preparative HPLC. Subsequently, HPLC fractions eluting between 10.20-10.80 min were collected and lyophilized. cortinarin C was isolated as a white solid (18.0 mg, total yield 3.00 %).

cortinarin C (2)

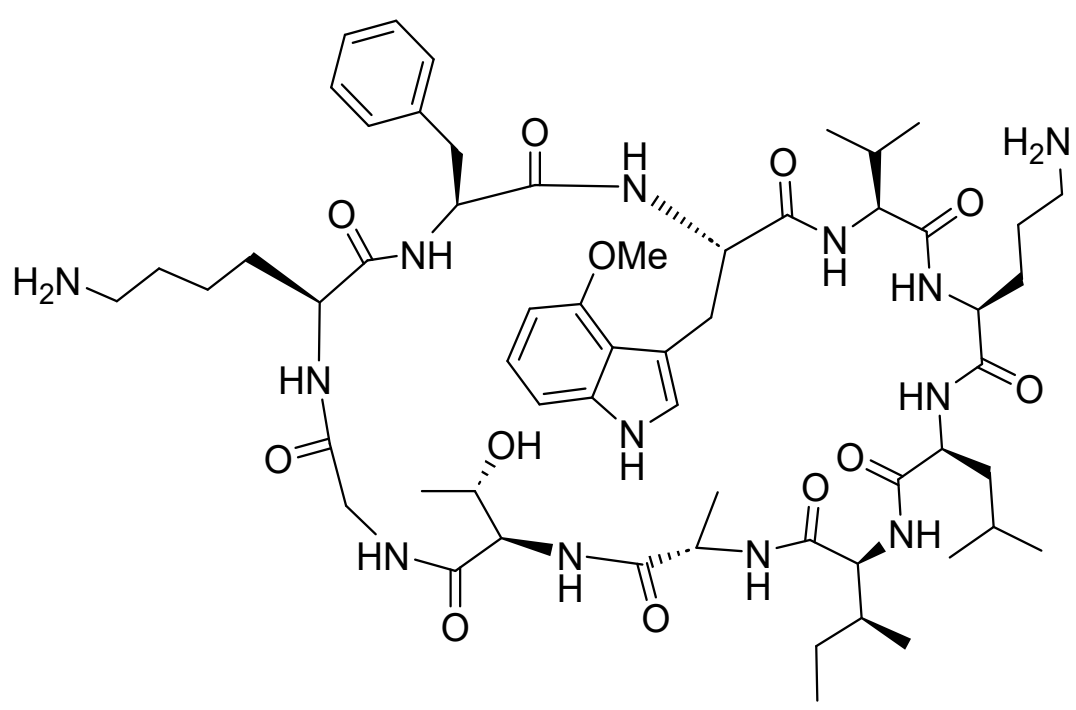

HRMS (ESI): m/z calculated $C_{58}H_{90}N_{13}O_{12}^{+}$ $[M+H]^{+}$ 1160.6826, found 1160.6831.

Analytical HPLC-MS: retention time R_t = 6.08 min

Preparative HPLC gradient:

Time (min)	Water (%)	Acetonitrile (%)
0	60	40
1	60	40
15	30	70

Waters C18 Column, flow rate (30 min/mL)

2.7 Synthesis of cortinarin X (1c)

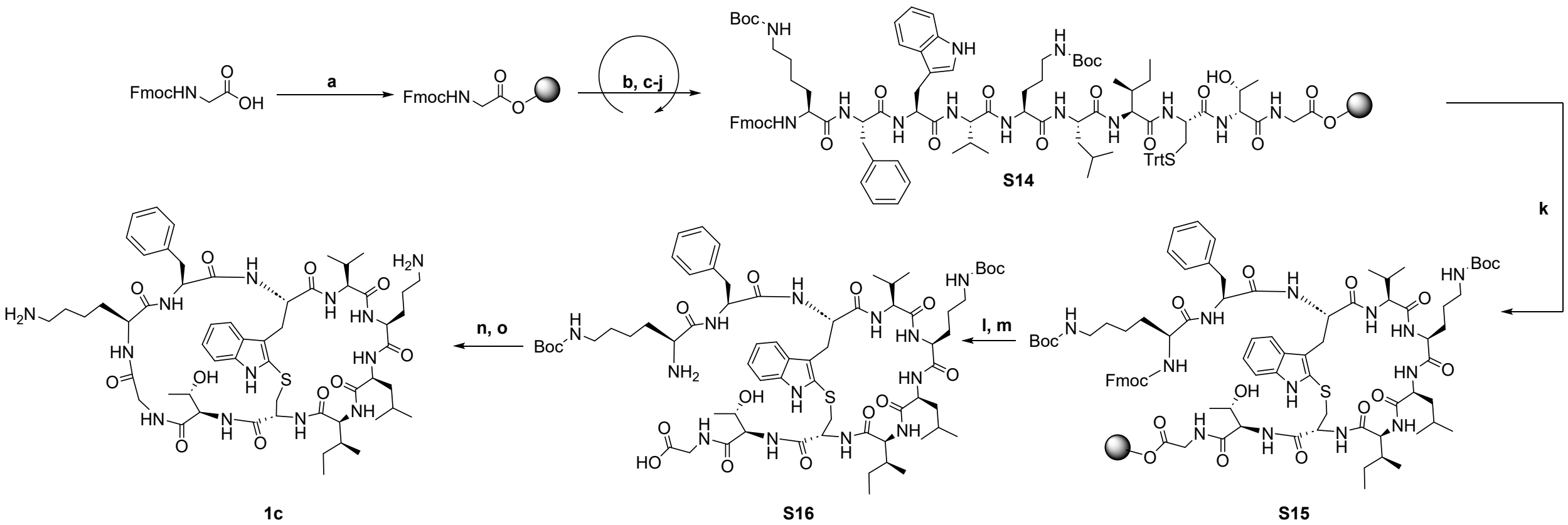

Scheme S5. Synthesis of cortinarin X (1c). a) 2-CTC resin, DIPEA, DCM, 2 h; b) Piperidine/DMF (1:4); c) Fmoc-D-Thr-OH, HATU, DIPEA, DMF; d) Fmoc-L-Cys(Trt)-OH, HATU, DIPEA, DMF; e) Fmoc-L-Ile-OH, HATU, DIPEA, DMF; f) Fmoc-L-Leu-OH, HATU, DIPEA, DMF; g) Fmoc-L-Orn(Boc)-OH, HATU, DIPEA, DMF; h) Fmoc-L-Trp-OH, HATU, DIPEA, DMF; i) Fmoc-L-Phe-OH, HATU, DIPEA, DMF; j) Fmoc-L-Lys(Boc)-OH, HATU, DIPEA, DMF; k) 2.00 mg/mL I_2 in DMF, 3 h; l) Piperidine/DMF (1:4); m) HFIP/DCM (1:4); n) HATU (2.00 equiv.), DIPEA (5.00 equiv.), DMF (peptide concentration, 2.00 μM), 12 h; o) TFA/DCM (3:7), 1 h.

2.7.1 Preparation of linear peptide S14

Following the above protocol (**subsection 2.3.1**), Fmoc-Gly-OH (149 mg, 0.50 mmol, 1.00 equiv.) was loaded on 2-CTC resin. For subsequent amino acid couplings, the following procedures were used:

A sequence of alternating Fmoc-deprotections (Method A) and amino acid couplings (Method B) was performed for the following amino acids: Fmoc-D-Thr-OH (341 mg, 1.00 mmol, 2.00 equiv.), Fmoc-L-Cys(Trt)-OH (586 mg, 1.00 mmol, 2.00 equiv.), Fmoc-L-Ile-OH (353 mg, 1.00 mmol, 2.00 equiv.), Fmoc-L-Leu-OH (353 mg, 1.00 mmol, 2.00 equiv.), Fmoc-L-Orn(Boc)-OH (455 mg, 1.00 mmol, 2.00 equiv.), Fmoc-L-Val-OH (339 mg, 1.00 mmol, 2.00 equiv.), Fmoc-L-Trp-OH (426 mg, 1.00 mmol, 2.00 equiv.), Fmoc-L-Phe-OH (387 mg, 1.00 mmol, 2.00 equiv.), and Fmoc-L-Lys(Boc)-OH (469 mg, 1.00 mmol, 2.00 equiv.) until the sequence of the peptide **S14** was completed.

2.7.2 Preparation of monocyclic decapeptide S16

A newly prepared solution of iodine in DMF (2.00 mg/mL) was added to the resin with the linear peptide **S14**. The mixture was agitated for 3 h, to form monocyclic decapeptide **S15**. After the reaction was completed (HPLC control), the solution was drained, and the resin was washed with DMF (5 x 10.0 mL).

The Fmoc group was removed using Method A. Once the Fmoc deprotection was completed (HPLC control) the resin was washed with DMF (5 x 10.0 mL). Following, the resin was treated with 10.0 mL of a mixture of HFIP/DCM (1:4) for 1 h at room temperature with gentle agitation. After cleavage, the beads were filtered and rinsed with DCM (2 x 10.0 mL). The filtrates were combined and evaporated at room temperature almost to dryness. Then, the aggregated peptide

S16 was precipitated in cold diethyl ether and regenerated from aggregates, following the general protocol of **subsection 2.3.2**.

2.7.3 Macrolactamization and final deprotection to cortinarin X

To a solution of monocyclic decapeptide **S16** (1.00 equiv) in solution of DIPEA (5.00 equiv.) in DMF (peptide concentration, 2.00 μM), HATU (2.00 equiv.) was added at room temperature. The solution was stirred for 12 h, then purified by preparative reversed phase automated flash chromatography (Biotage Sfär Bio C18 D column A; gradient 30-100% CH_3CN/H_2O in 10.0 min; flow rate of 40.0 mL/min; retention time: 4.20-6.30 min). The isolated product was lyophilized to give a crude white solid (65.0 mg).

The white solid (65.0 mg, 0.047 mmol) was dissolved using a TFA buffer (5.00 mL, TFA/DCM, 3:7) and was stirred for 1 h. Then the mixture was concentrated by rotatory evaporator and purified by preparative HPLC. Subsequently, HPLC fractions eluting between 10.40-10.65 min *M*-ansamer of cortinarin X ($1c_A$, $CorX_M$) and 10.70-11.00min *P*-ansamer of cortinarin X ($1c_B$, $CorX_P$) were collected, unified, and lyophilized. $1c_A$ was isolated as a white solid (34.5 mg, total yield 5.90%), and $1c_B$ was isolated as a white solid (4.20 mg, total yield 0.70 %)

***M*-ansamer of cortinarin X ($1c_A$, $CorX_M$)**

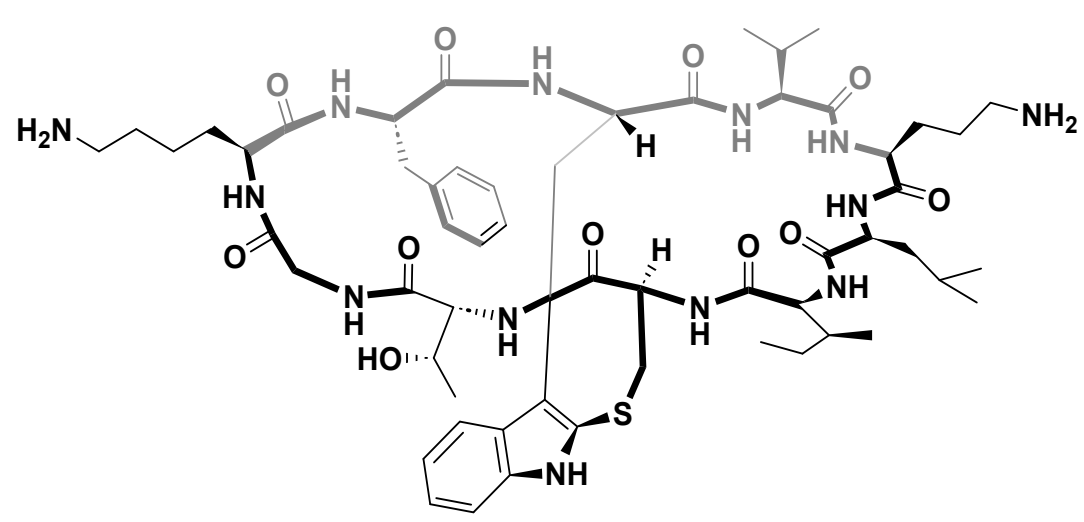

HRMS (ESI): m/z calculated $C_{57}H_{86}N_{13}O_{11}S^+$ $[M+H]^+$ 1160.6285, found 1160.6282.

Analytical HPLC-MS: retention time R_t = 5.19 min

Preparative HPLC gradient:

Time (min)	Water (%)	Acetonitrile (%)
0	95	5
1	95	5
30	0	100

Waters C18 Column, flow rate (30 min/mL)

***P*-ansamer of cortinarin X ($1c_B$, $CorX_P$)**

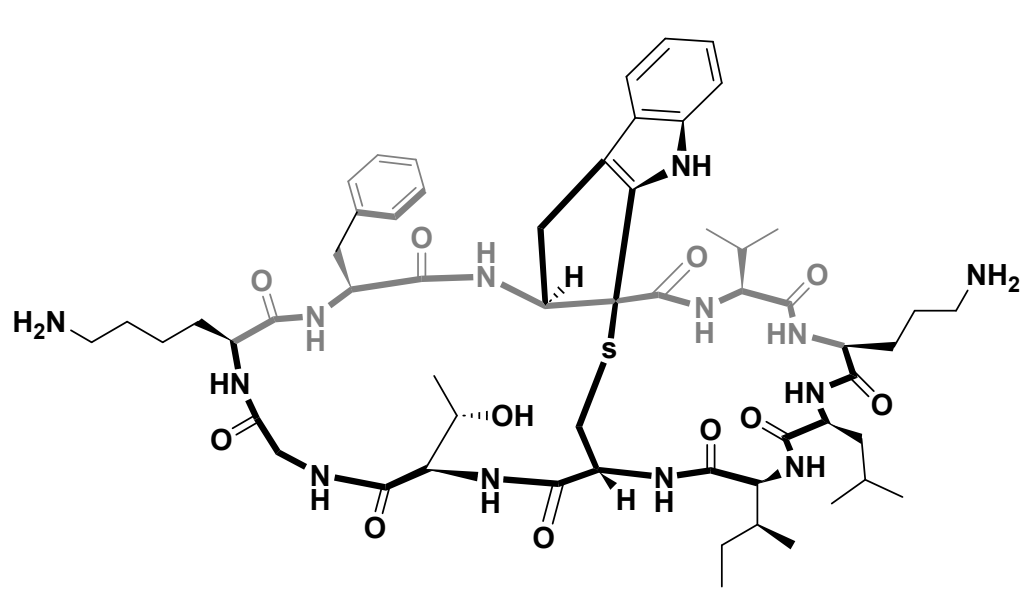

HRMS (ESI): m/z calculated $C_{57}H_{86}N_{13}O_{11}S^+$ $[M+H]^+$ 1160.6285, found 1160.6283.

Analytical HPLC-MS: retention time R_t = 5.04 min

Preparative HPLC gradient:

Time (min)	Water (%)	Acetonitrile (%)
0	95	5
1	95	5
30	0	100

Angilent C18 Column, flow rate (30 min/mL)

2.7.4 Scan of an optimized macrolactamization site in the synthesis of CorX.

Table S2. Scan for an optimized macrolactamization site for the significant ansamer selectivity in the synthesis of CorX.

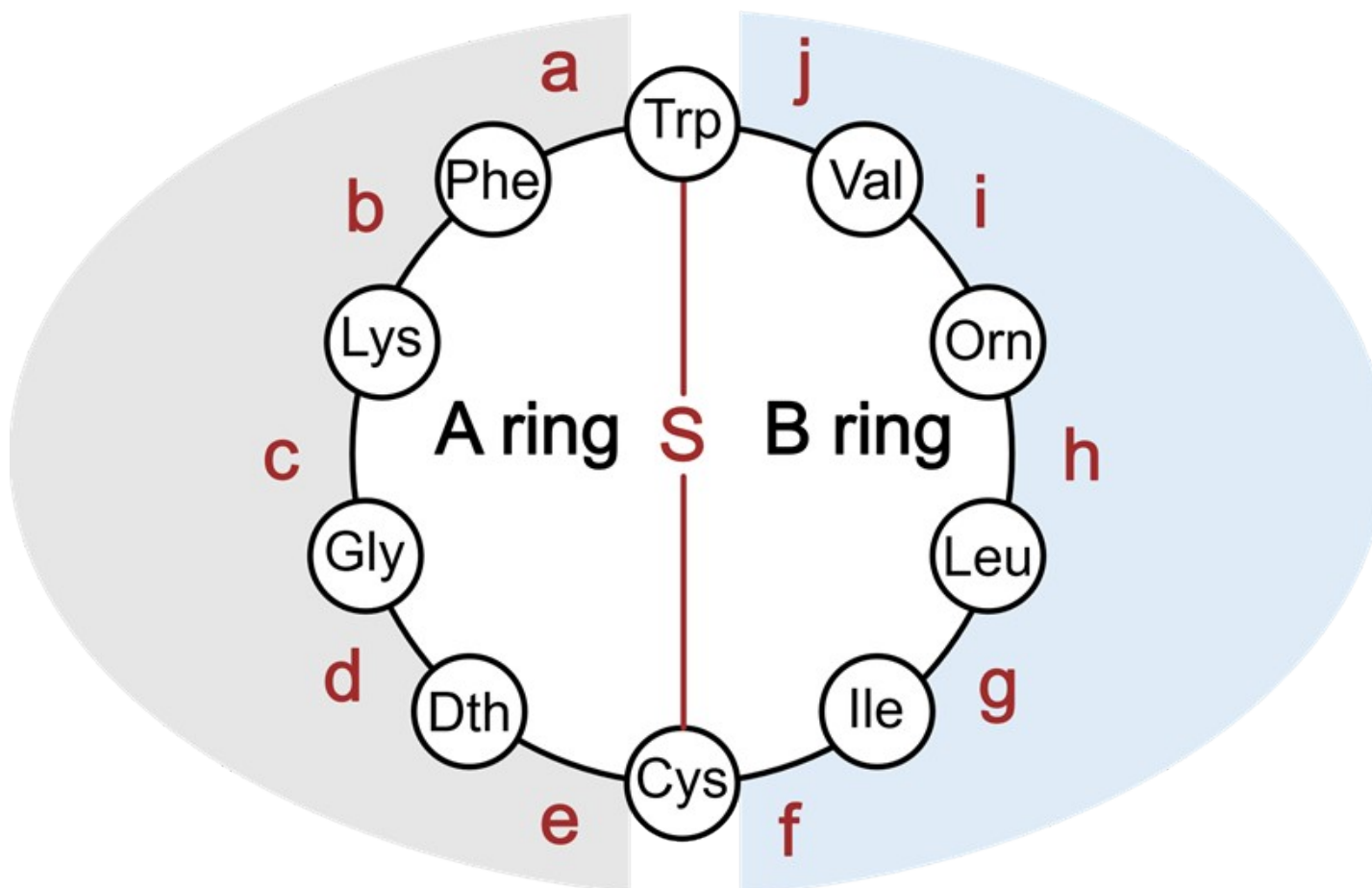

A ring				B ring			
Coupling site	C-terminus	N-terminus	Ansamer ratio (*P*:*M*)	Coupling site	C-terminus	N-terminus	Ansamer ratio (*P*:*M*)
a	Phe	Trp	1:0	f	Ile	Leu	N.D.
b	Lys	Phe	8:2	g	Leu	Orn	N.D.
c*	Gly	Lys	9:1	h	Orn	Val	N.D.
d	Dth	Gly	N.D.	i	Val	Trp	1:0
e	Cys	Dth	1:0	j	Trp	Val	N.D.

N.D. = CorX not detected.
c*: coupling site c, the ansamer ratio chromatogram is shown in Figure 2B.
ansamer ratios were obtained by peak integration via the Xcalibur software (Thermo Scientific).

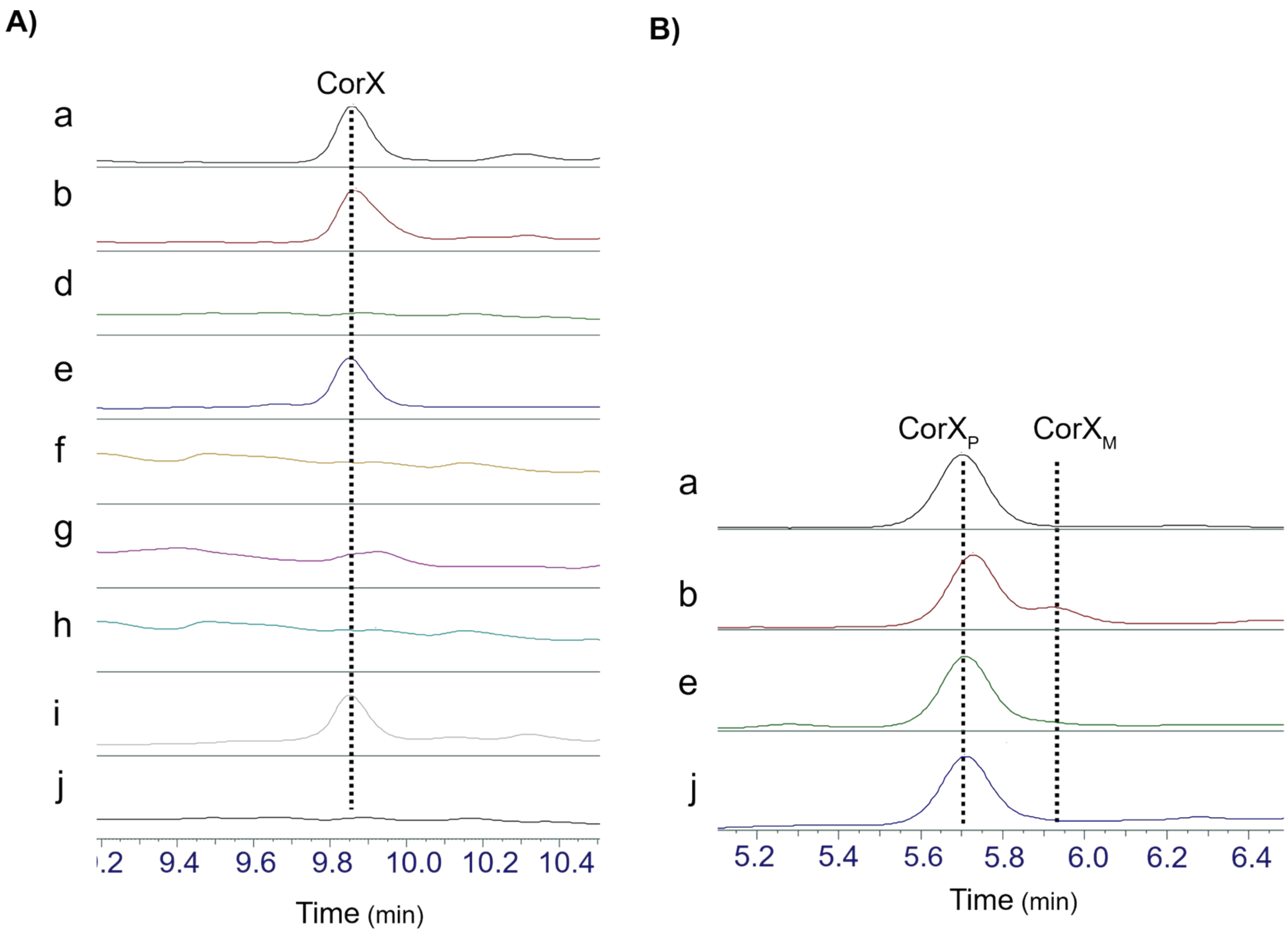

Figure S2.HPLC-HRMS chromatograms of the macrolactamization reactions at different sites. To facilitate the ratio quantification, the Boc-protected derivative (CorX) was analyzed instead of the deprotected final CorX product. Comparison of HPLC-HRMS chromatograms (gradient A) of the macrolactamization experiments (sites a, b, d-j). Further details of the macrolactamization sites for a-j are provided in Table S2. For the chromatogram of c, see Figure 2B (main text). Retention time of CorX$_{P/M}$ R_t = 9.85 min (not resolved). For macrocyclizations d, f, g, h, and j the formation of CorX$_{P/M}$ was not observed. B) Close up of HPLC-HRMS chromatograms (gradient B) of a, b, e, and j. Assignment of CorX$_P$ R_t = 5.71 min and of the CorX$_M$ R_t = 5.92 min.

2.8 Synthesis of cortinarin X-Ala (S20)

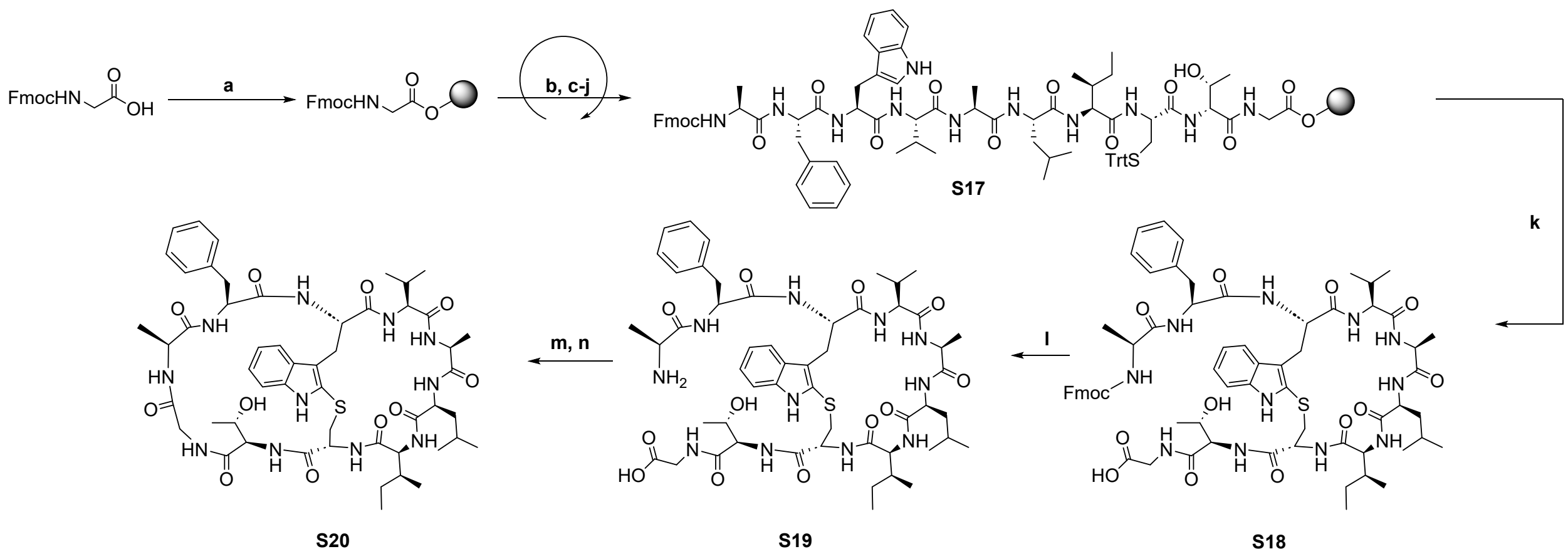

Scheme S6. Synthesis of cortinarin X-Ala (S20). a) 2-CTC resin, DIPEA, DCM, 2 h; b) Piperidine/DMF (1:4); c) Fmoc-D-Thr-OH, HATU, DIPEA, DMF; d) Fmoc-L-Cys(Trt)-OH, HATU, DIPEA, DMF; e) Fmoc-L-Ile-OH, HATU, DIPEA, DMF; f) Fmoc-L-Leu-OH, HATU, DIPEA, DMF; g) Fmoc-L-Ala-OH, HATU, DIPEA, DMF; h) Fmoc-L-Trp-OH, HATU, DIPEA, DMF; i) Fmoc-L-Phe-OH, HATU, DIPEA, DMF; j) Fmoc-L-Ala-OH, HATU, DIPEA, DMF; k) 2.00 mg/mL I_2 in DMF, 3 h; l) Piperidine/DMF (1:4); m) HFIP/DCM (1:4); n) HATU (2.00 equiv.), DIPEA (5.00 equiv.), DMF (peptide concentration, 2.00 µM), 12 h.

2.8.1 Preparation of resin-bound linear peptide S17

Following the above protocol (**subsection 2.3.1**), Fmoc-Gly-OH (149 mg, 0.50 mmol, 1.00 equiv.) was loaded on 2-CTC resin. For the subsequent amino acid couplings, the following procedures were used:

A sequence of alternating Fmoc-deprotections (Method A) and amino acid couplings (Method B) was performed for the following amino acids: Fmoc-D-Thr-OH (341 mg, 1.00 mmol, 2.00 equiv.), Fmoc-L-Cys(Trt)-OH (586 mg, 1.00 mmol, 2.00 equiv.), Fmoc-L-Ile-OH (353 mg, 1.00 mmol, 2.00 equiv.), Fmoc-L-Leu-OH (353 mg, 1.00 mmol, 2.00 equiv.), Fmoc-L-Ala-OH (311 mg, 1.00 mmol, 2.00 equiv.), Fmoc-L-Val-OH (339 mg, 1.00 mmol, 2.00 equiv.), Fmoc-L-Trp-OH (426 mg, 1.00 mmol, 2.00 equiv.), Fmoc-L-Phe-OH (387 mg, 1.00 mmol, 2.00 equiv.), and Fmoc-L-Ala-OH (311 mg, 1.00 mmol, 2.00 equiv.) until the sequence of the peptide **S17** was completed.

2.8.2 Preparation of monocyclic decapeptide S18

A newly prepared solution of iodine in DMF (2.00 mg/mL) was added to the resin with the linear peptide **S17**. The mixture was agitated for 3 h, to form monocyclic decapeptide **S18** was formed. After the reaction was completed, determined by HPLC, the solution was drained, and the resin was washed with DMF (5 x 10.0 mL).

The Fmoc group was removed using Method A. Once the Fmoc deprotection was completed (HPLC control) the resin was washed with DMF (5 x 10.0 mL). Following, the resin was treated with 10 mL of a mixture of HFIP/DCM (1:4) for 1 h at room temperature with gentle agitation. After cleavage, the beads were filtered and rinsed with DCM (2 x 10.0 mL). The filtrates were combined and evaporated at room temperature almost to dryness. Then, the aggregated peptide **S19** was precipitated in cold diethyl ether and regenerated from aggregates, following the general method of **subsection 2.3.2**.

2.8.3 Macrolactamization and deprotection of protected cortinarin X-Ala (S20).

To a solution of monocyclic decapeptide **S19** (1.00 equiv.) in solution of DIPEA (5.00 equiv.) in DMF (peptide concentration 2.00 μM), HATU (2.00 equiv.) was added at room temperature. The solution was stirred for 12 h, then purified by preparative reversed phase automated flash chromatography (Biotage Sfär Bio C18D column A; gradient 30-100% CH_3CN/H_2O in 10 min; flow rate of 40.0 mL/min; retention time: 4.20-6.30 min). The isolated product was lyophilized to give a crude white solid (40.0 mg).

Crude product was purified by preparative HPLC. Subsequently, HPLC fractions eluting between 9.40-10.20 min ($S20_A$, *P*-ansamer of cortinarin X-Ala) and 10.50-11.00 min ($S20_B$, *M*-ansamer of cortinarin-Ala) were collected, unified, and lyophilized. $S20_A$ was isolated as a white solid (16.0 mg, total yield 3.00%), and $S20_B$ was isolated as a white solid (2.00 mg, total yield 0.40%)

***P*-ansamer of cortinarinX-Ala ($S20_A$, CorX-Ala_P)**

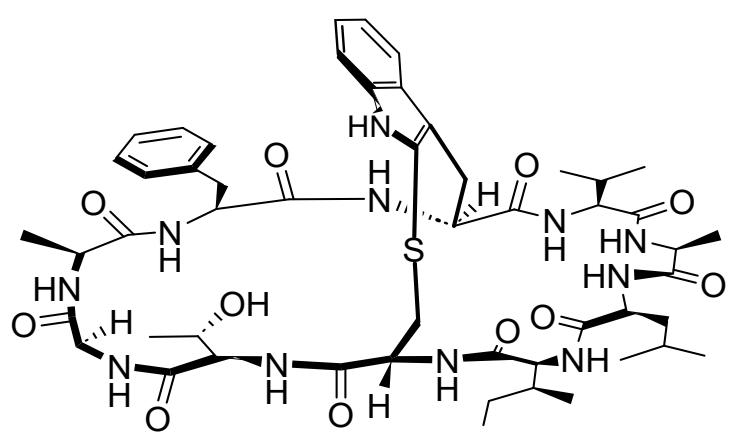

HRMS (ESI): m/z calculated $C_{52}H_{74}N_{11}O_{11}S^+$ $[M+H]^+$ 1060.5284, found 1060.5286

Analytical HPLC-MS: retention time R_t= 7.68 min

Preparative HPLC gradient:

Time (min)	Water (%)	Acetonitrile (%)
0	65	35
1	65	65
15	35	65

Waters C18 Column, flow rate (30 min/mL)

***M*-ansamer of cortinarin X-Ala ($S20_B$, CorX-Ala_M)**

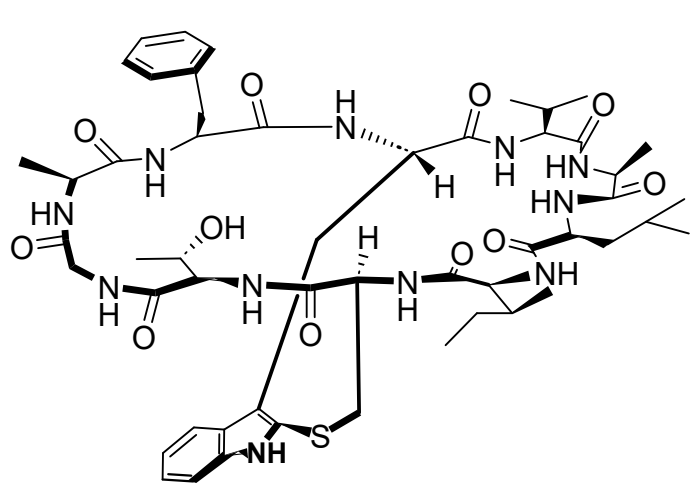

HRMS (ESI): m/z calculated $C_{52}H_{74}N_{11}O_{11}S^+$ $[M+H]^+$ 1060.5284, found 1060.5286

Analytical HPLC-MS: retention time R_t = 7.92 min

Preparative HPLC gradient:

Time (min)	Water (%)	Acetonitrile (%)
0	65	35
1	65	65
15	35	65

Waters C18 Column, flow rate (30 min/mL)

3 Amino acid analysis with Marfey's reagent

Amino acid analysis of CorX$_P$ and CorX$_M$ was performed with Marfey's reagent (1-fluoro-2,4-dinitrophenyl-leucine-amide, FDLA).[5] The absolute configurations of the amino acid units in CorX$_P$ and CorX$_M$ were determined by the advanced Marfey's method.[6] The hydrolysis products (6 M HCl, 110 °C, 12 h) of CorX$_P$ and CorX$_M$ were subjected to D-FDLA and L-/D-FDLA derivatization and analyzed by LC-MS, which showed that the configurations of amino acid residues of CorX$_P$ were identical with CorX$_M$ (**Figure S3**).

Protocol: Ansamers CorX$_P$ and CorX$_M$ (0.1 mg each) were hydrolyzed by stirring in 6 M HCl (200 µL) at 110 °C for 12 h in a 5 mL seal tube. The residual HCl gas was removed under a stream of N_2. Hydrolysates were suspended in H_2O (50 µL) and treated with 1 M $NaHCO_3$ (20.0 µL) and then incubated with D-FDLA and L-/D-FDLA (100 µL of a 10.0 mg/mL solution in acetone). The mixture was stirred at 37 °C for 1 h. The reaction was quenched with 1 M HCl (20.0 µL) and then diluted with MeOH for subsequent analysis by analytical HPLC-HRMS (**gradient C, subsection 2.1**)

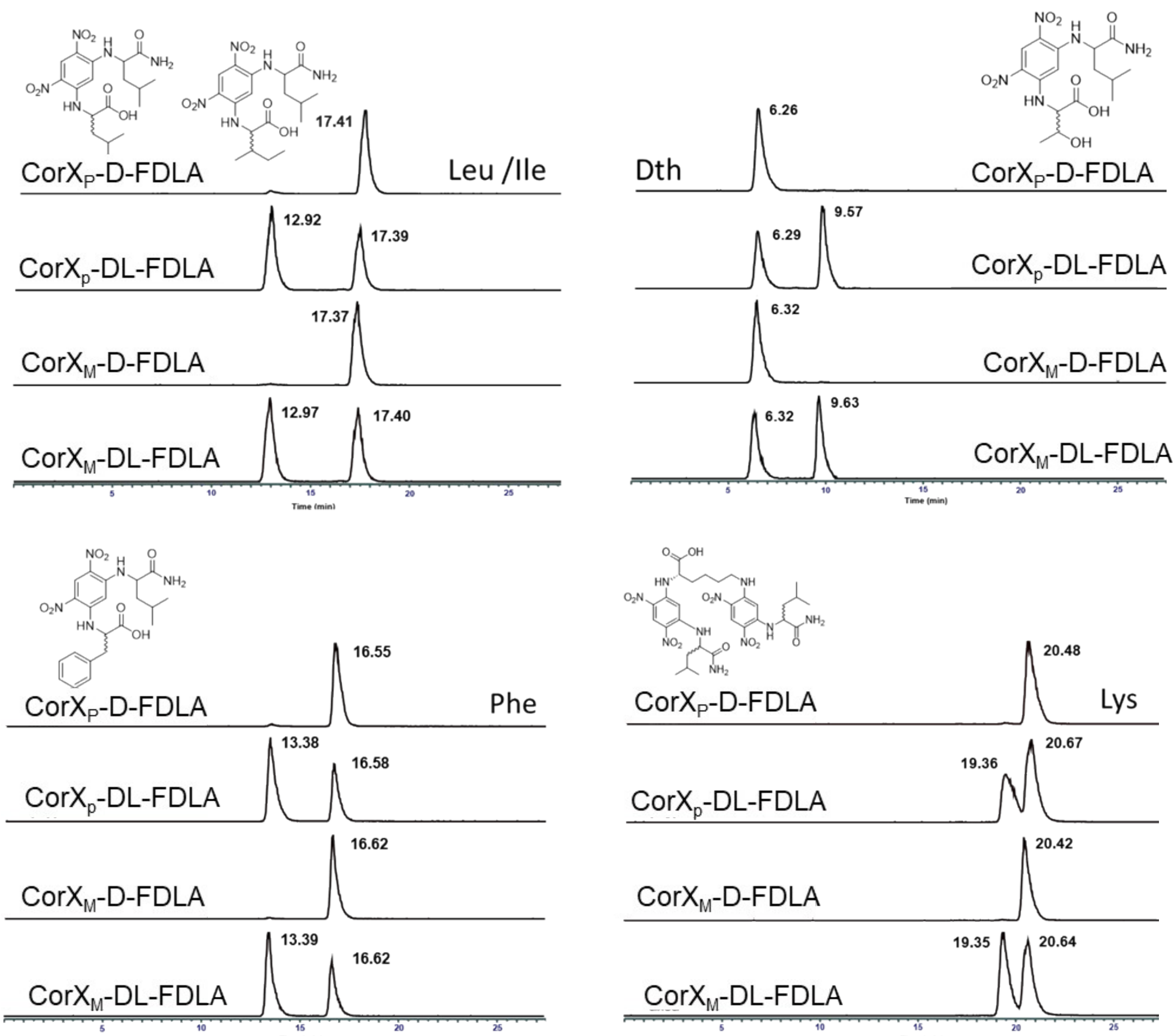

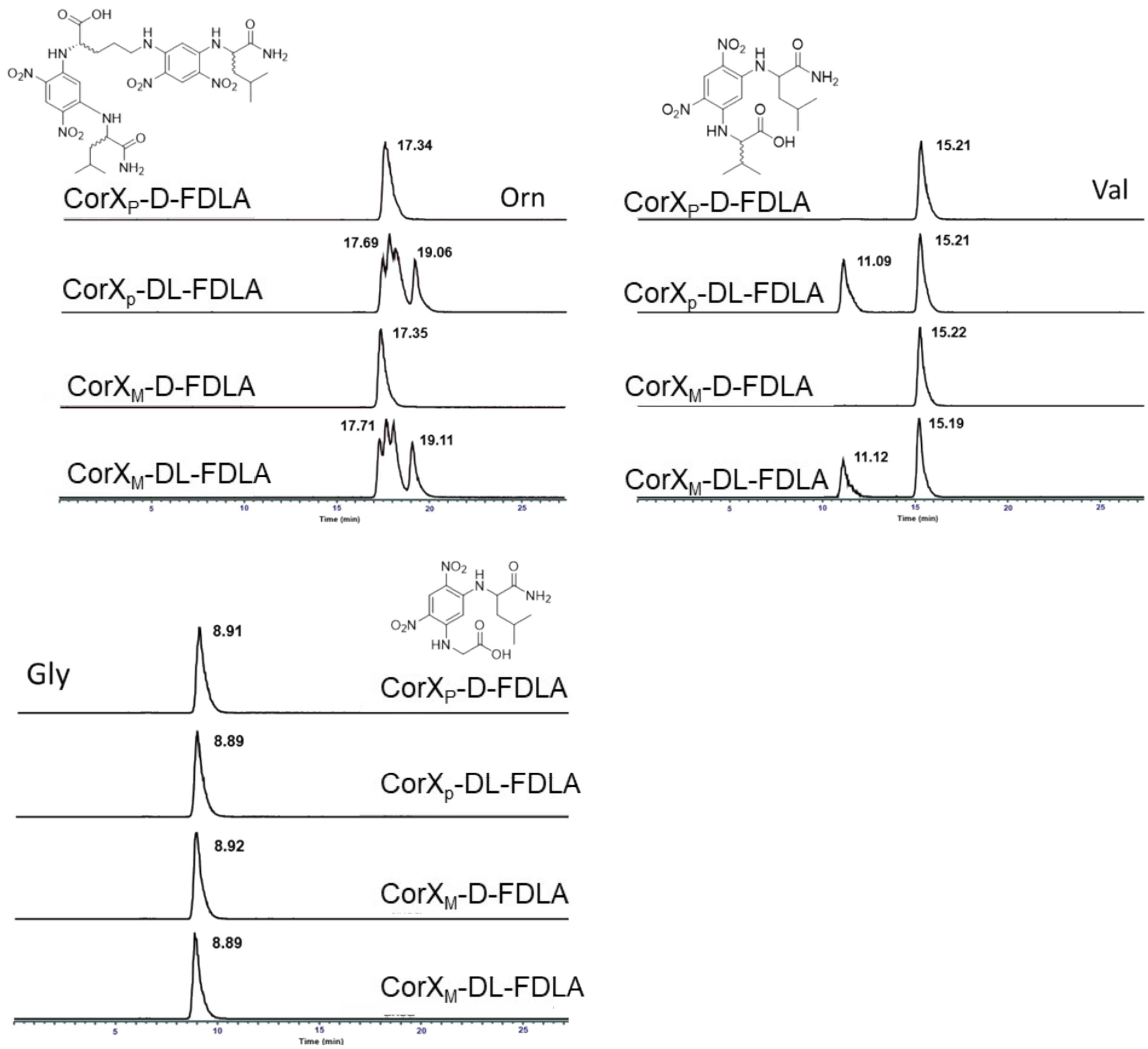

Figure S3. Marfey analytics of amino acids in CorX$_P$ and CorX$_M$. HPLC-MS chromatograms of the amino acid analysis of ansamers CorX$_P$ and CorX$_M$ (total hydrolysis was followed by modification with Marfey's reagent, treated either with D-FDLA or the racemic DL-FDLA reagent).

4 UV spectra of synthetic cortinarin A, B and C

Sample preparation: Synthetic cortinarin A (**1a**, 1.00 mg), B (**1b**, 1.00 mg), and C (**2**, 1.00 mg) were dissolved in H_2O (1 mL) and diluted to a concentration of 0.85 mM. UV spectra were measured as described in the general experimental details section.

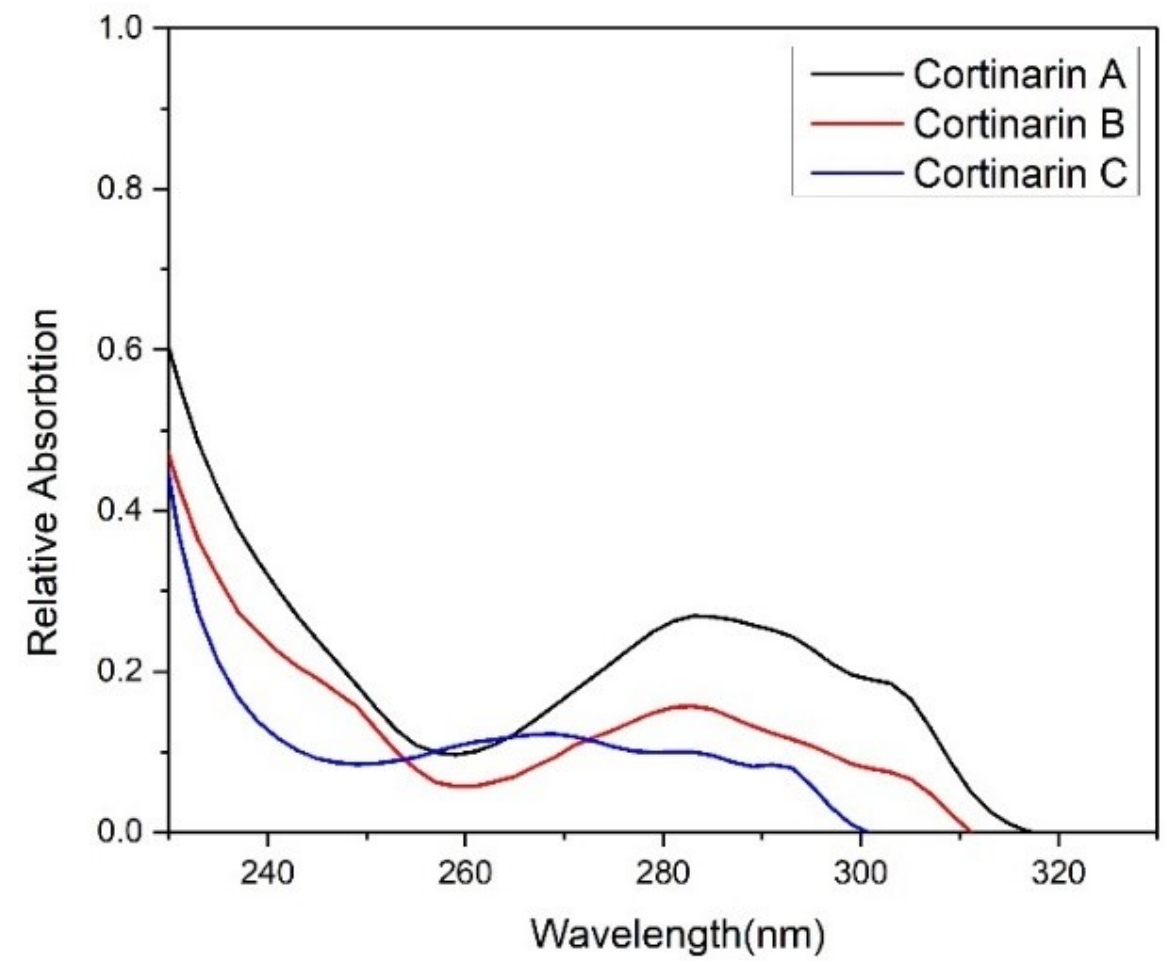

Figure S4. UV spectra of cortinarin A (λ_{min} = 259 nm, λ_{max} = 283 nm), **cortinarin B** (λ_{min} = 259 nm, λ_{max} = 283 nm), **and cortinarin C** (λ_{min} = 249 nm, λ_{max} = 269 nm).

5 CD spectra of synthetic cortinarin A, B, and C

Sample preparation: Synthetic cortinarin A (**1a**, 1.00 mg), B (**1b**, 1.00 mg), and C (**2**, 1.00 mg) were dissolved in H_2O (1 mL) and diluted to a concentration of 0.85 mM.

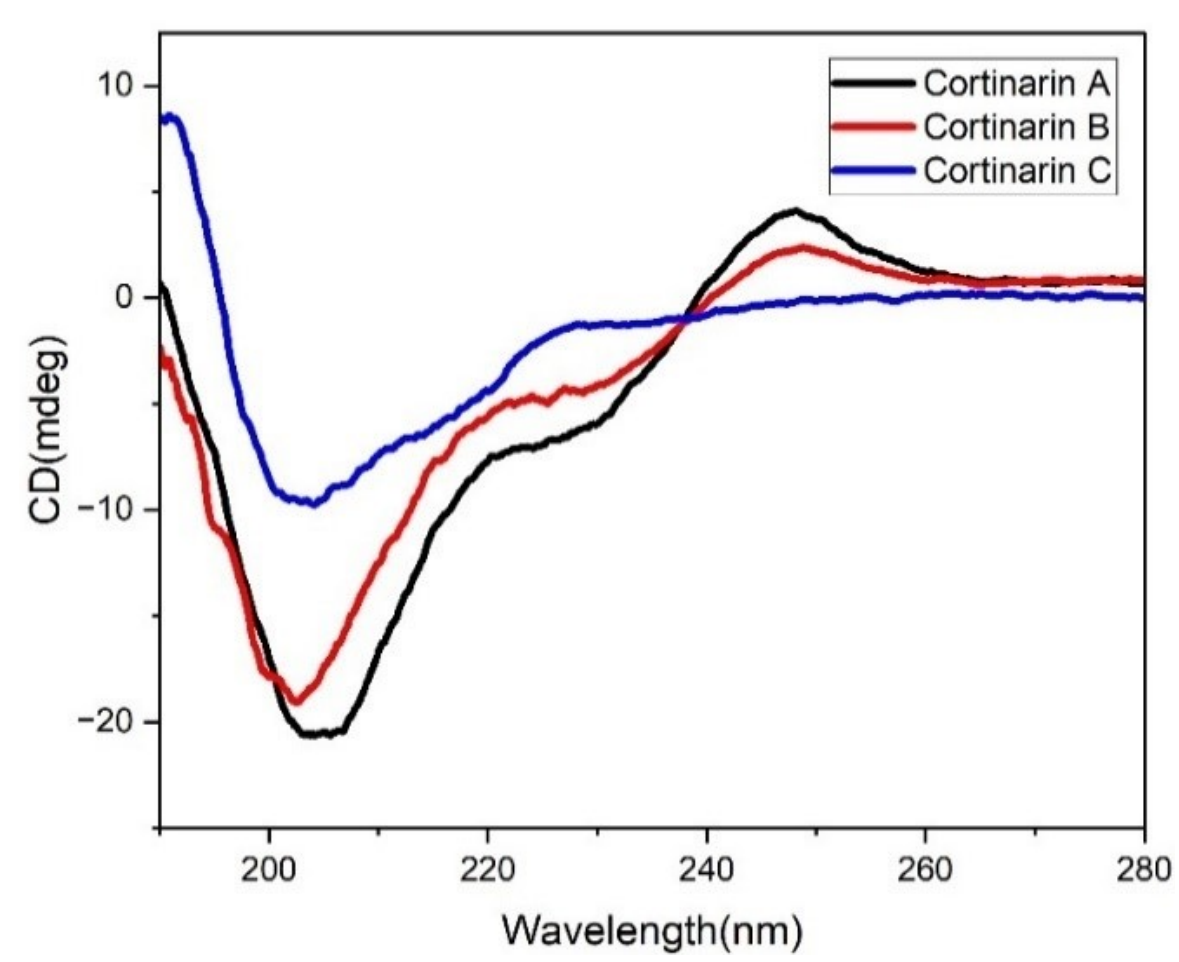

Figure S5. Far-UV CD spectra of cortinarin A (λ_{min} = 204 nm, λ_{max} = 248 nm), **cortinarin B** (λ_{min} = 203 nm, λ_{max} = 249 nm), and **cortinarin C** (λ_{min} = 204 nm, λ_{max} = 229 nm).

6 NMR analysis of cortinarin analogs

To obtain resonance assignments for structure calculations, cortinarin derivatives were dissolved in deuterated DMSO-d_6 (approx. 0.01 M). TOCSY, COSY and NOESY spectra were recorded on a Bruker Avance III 700 MHz or Avance II 500 MHz spectrometer with a TXI 3 or 5 mm probe. Standard Bruker pulse programs were used, and all spectra were acquired at 298 K. NOESY spectra of CorX$_P$ and CorX$_M$ were recorded with a mixing time of 150 ms. Others were recorded with a mixing time of 600 ms. Residual solvent methyl peaks (DMSO-d_6, δ = 2.50 ppm) were used for chemical shift referencing. The spectra were processed and analysed using TopSpin 3.7.0 (Bruker) and CcpNmr 2.5.2. [7]

6.1 Cortinarin A (1a, $CorA_P$)

Table S3. ^{1}H-NMR chemical shifts [ppm] of $CorA_P$ (1a).

Residue	HN	Hα	Hβ	Hγ	Hδ	Hε	Hζ	Others (indole)
**Lys[1]**	6.89	4.26	1.54, 1.30	1.09, 0.99	1.36, 1.32	2.53	7.65	
**Phe[2]**	8.61	4.83	3.09, 2.61		7.10	7.13	7.13	
**(4-OMe)Trp[3]**	8.42	4.76	3.05, 3.30					11.22, 6.88, 6.99, 6.50, 3.87
**Val[4]**	8.05	3.43	2.60	1.04, 0.94				
**Orn[5]**	8.25	4.16	1.70	1.57, 1.48	2.77	7.75		
**Leu[6]**	7.42	4.62	1.80	1.56	0.90			
**Ile[7]**	8.78	3.75	1.72	1.58; 0.85	0.85			
**Cys[8]**	8.24	4.69	3.77, 2.59					
**D-Thr[9]**	7.38	3.96	3.74	5.04, 1.02				
**Gly[10]**	8.59	3.77, 3.42						

Table S4. ^{1}H, ^{13}C-HSQC chemical shifts [ppm] of $CorA_P$ (1a).

Residue	Cα	Cβ	Cγ	Cδ	Cε	Cζ	Others (indole)
**Lys[1]**	50.23	33.36	20.08	26.74	38.71		
**Phe[2]**	54.79	38.02		129.19	127.88	127.88	
**(4-OMe)Trp[3]**	54.79	28.10					104.73, 123.20, 99.94, 55.81
**Val[4]**	64.02	38.02	20.24; 20.80				
**Orn[5]**	53.08	28.45	25.37	39.02			
**Leu[6]**	51.28	42.13	33.24	23.66			
**Ile[7]**	59.92	35.63	25.37; 15.97	11.68			
**Cys[8]**	50.00	38.36					
**D-Thr[9]**	60.94	65.73	20.20				
**Gly[10]**	43.16						

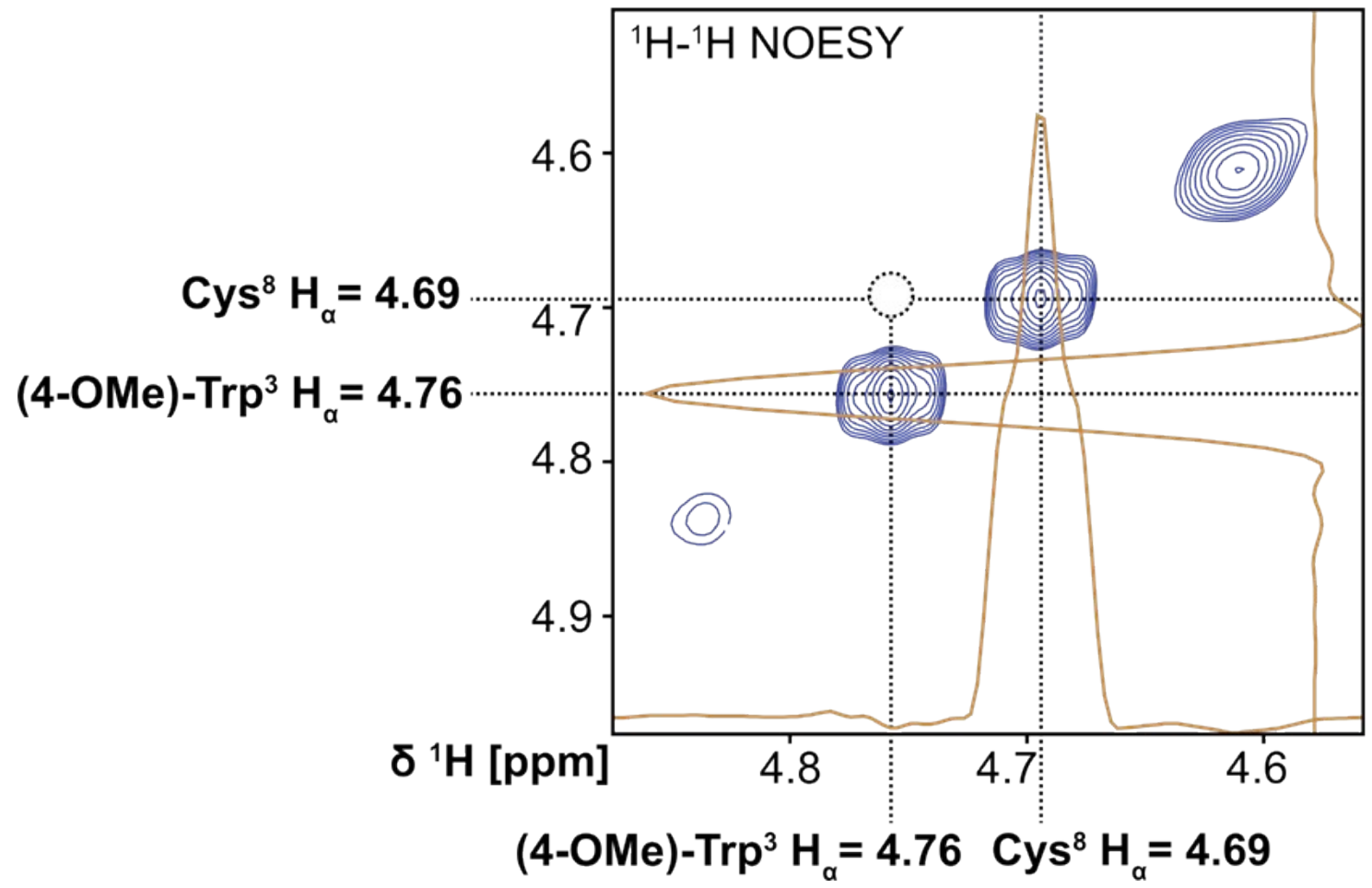

Figure S6. Diagnostic NOESY region of the Hα atoms of Cys^8 and (4-OMe)Trp^3 in NMR spectra of CorA$_P$ (blue contours). NOESY spectra were acquired with a mixing time of 600 ms. The orange curves depict representative 1D projections/slices along the diagonal peaks in the direct and indirect dimension. The position of the theoretical, but missing NOE cross peak between Hα atoms of Cys^8 and (4-OMe)Trp^3 in cortinarin A is indicated with a dashed circle. The product cortinarin A was thus determined to be the *P*-ansamer and designated CorA$_P$.

6.2 Cortinarin B (1b, $CorB_P$)

Table S5. ^{1}H-NMR chemical shifts (ppm) of $CorB_P$ (1b).

Residue	HN	Hα	Hβ	Hγ	Hδ	Hε	Hζ	Others (indole)
**Lys^1**	6.96	4.27	1.28, 1.55	1.10	1.35	2.58	7.65	
**Phe^2**	8.74	4.75	3.21, 2.72		7.16	7.17	7.17	
**(4-OH)Trp^3**	8.24	4.78	3.57, 3.20					11.07, 6.76, 6.89, 6.48, 9.89
**Val^4**	7.43	3.58	2.29	1.08, 0.95				
**Orn^5**	8.19	4.23	1.58, 2.01	1.53	2.78	7.77		
**Leu^6**	6.96	4.65	1.65	1.76	0.87			
**Ile^7**	8.73	3.91	1.74	1.58; 0.84	0.85			
**Cys^8**	8.29	4.73	3.65, 2.54					
**D-Thr^9**	7.54	3.91	3.76	5.05	1.03			
**Gly^{10}**	8.54	3.78, 3.38						

Table S6. ^{1}H, ^{13}C-HSQC chemical shifts [ppm] of $CorB_P$ (1b).

Residue	Cα	Cβ	Cγ	Cδ	Cε	Cζ	Others (indole)
**Lys^1**	50.31	24.63	20.45	26.43	38.69		
**Phe^2**	55.21	38.40		129.07	126.72	126.67	
**(4-OH)Trp^3**	55.45	28.23					103.21,123.59,103.90
**Val^4**	63.67	28.40	20.53				
**Orn^5**	52.71	28.91	23.95	39.02			
**Leu^6**	50.66	27.14	24.38	22.75			
**Ile^7**	59.73	35.59	24.63; 15.90				
**Cys^8**	49.63	39.02					
**D-Thr^9**	61.37	65.72	20.24				
**Gly^{10}**	65.72						

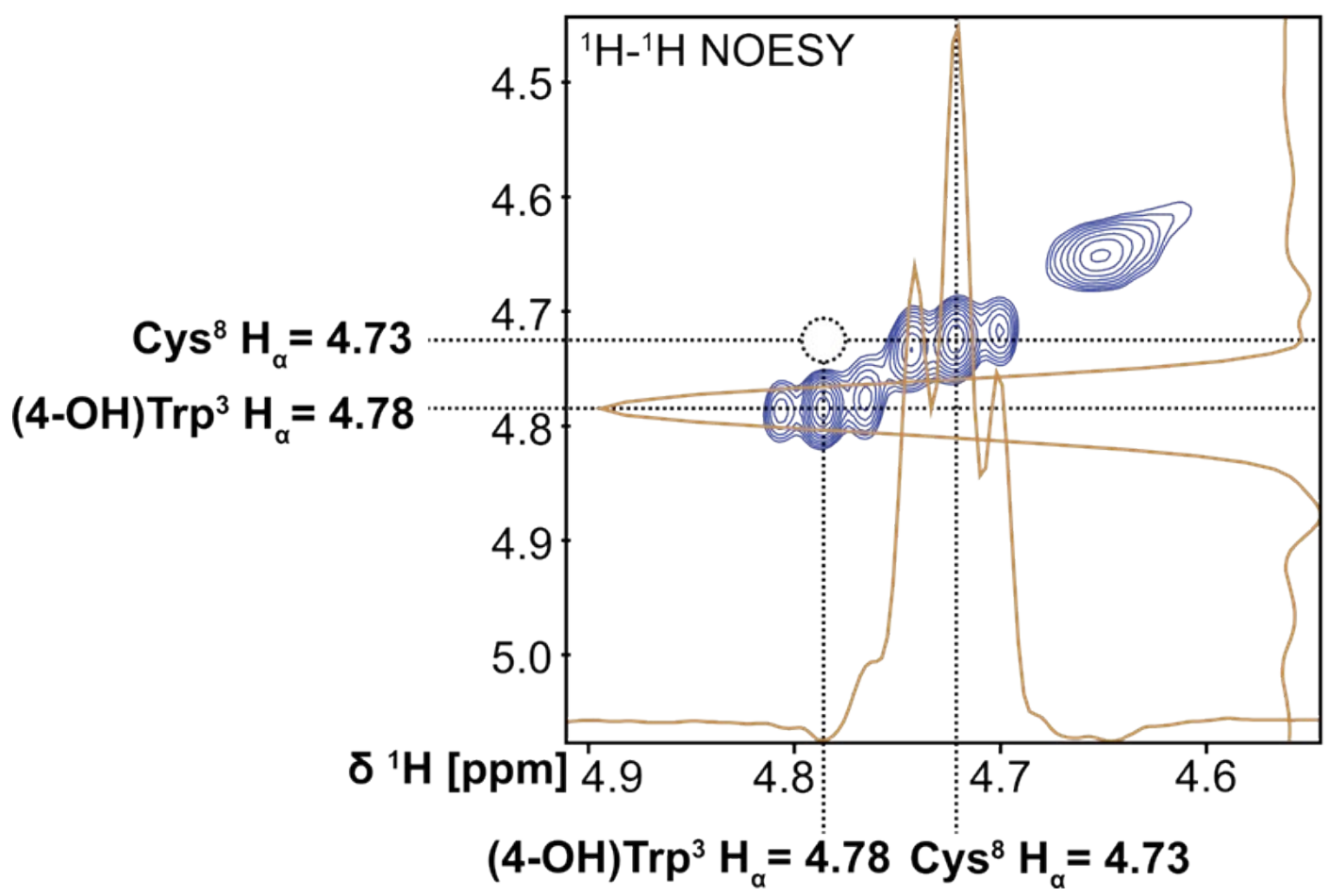

Figure S7. Diagnostic NOESY signals of the Hα atoms of Cys[8] and (4-OH)Trp[3] in NMR spectra of CorB$_P$ (blue contours). NOESY spectra were acquired with a mixing time of 600 ms. The orange curves depict representative 1D projections/slices along the diagonal peaks in the direct and indirect dimension. The position of the theoretical, but missing NOE cross peak between Hα atoms of Cys[8] and (4-OH)Trp[3] in cortinarin B is indicated with a dashed circle. The product cortinarin B was thus determined to be the *P*-ansamer and designated CorB$_P$.

6.3 Cortinarin C (2, CorC)

Table S7. ^{1}H-NMR chemical shifts [ppm] of CorC (2).

Residue	HN	Hα	Hβ	Hγ	Hδ	Hε	Hζ	Others (indole)
**Lys^{1}**	7.96	4.17	1.38	1.14	1.40	2.68	7.70	
**Phe^{2}**	7.90	4.47	3.05, 2.80		7.18	7.23	7.17	
**(4-OMe)Trp^{3}**	8.48	4.42	3.46, 3.16					10.8, 6.93, 6.91, 6.96,6.45, 3.85
**Val^{4}**	7.43	4.10	2.13	0.86, 0.83				
**Orn^{5}**	8.04	4.32	1.63, 1.86	1.52	2.84	7.60		
**Leu^{6}**	8.05	4.34	1.52	1.58	0.84			
**Ile^{7}**	7.60	4.23	1.72	1.04, 1.41; 0.84	0.79			
**Ala^{8}**	8.33	4.30	1.24					
**D-Thr^{9}**	8.02	4.11	4.09; 1.08					
**Gly^{10}**	8.12	3.78, 3.70						

Table S8. ^{1}H, ^{13}C-HSQC chemical shifts [ppm] of CorC (2).

Residue	Cα	Cβ	Cγ	Cδ	Cε	Cζ	Others (indole)
**Lys^{1}**	53.05	31.66	22.43	24.63	39.02		
**Phe^{2}**	54.49	37.81		129.41	128.55	126.67	
**(4-OMe)Trp^{3}**	56.48	28.57					122.39,105.44,122.39,99.28,55.45
**Val^{4}**	66.75	29.77	19.84;18.47				
**Orn^{5}**	52.30	28.74	23.61	39.19			
**Leu^{6}**	52.03	41.24	26.44	19.84;18.47			
**Ile^{7}**	56.99	37.30	24.63;15.56	11.45			
**Ala^{8}**	49.29	17.79					
**D-Thr^{9}**	59.25	59.05	20.36				
**Gly^{10}**	42.95						

6.4 *P*-ansamer of cortinarin X (1c$_B$, CorX$_P$)

Table S9. ^{1}H-NMR chemical shifts [ppm] of CorX$_P$ (1c$_B$). [1]

Residue	HN	Hα	Hβ	Hγ	Hδ	Hε	Hζ	Others (indole)
**Lys^1**	6.90	4.17(4.10)	1.25, 1.36	1.00, 0.89	1.36	2.56	7.62	
**Phe^2**	8.46(6.56)	4.61	2.98, 2.54		7.07	7.15	7.15	
**Trp^3**	7.94(8.29)	4.83(4.22)	3.30, 3.15					11.20, 7.25, 7.08, 7.02, 7.78
**Val^4**	8.60(8.11)	3.71(3.87)	2.27	1.01, 0.93				
**Orn^5**	8.53(9.25)	3.92(3.59)	2.08, 1.73	1.62, 1.51	2.79	7.73		
**Leu^6**	7.19(8.42)	4.56(3.49)	1.54, 1.45	1.72	0.84, 0.79			
**Ile^7**	8.69(7.39)	3.93(3.82)	1.80	1.15; 0.88	0.88			
**Cys^8**	8.35	4.78	3.02, 3.56					
**D-Thr^9**	7.47(8.08)	3.98(4.14)	3.84	5.06; 1.03(1.26)				
**Gly^{10}**	8.43(7.16)	3.74, 3.58						

[1] The chemical shift in parentheses belongs to another observed conformer (25%) due to slow exchange

Table S10. ^{1}H, ^{13}C-HSQC chemical shifts [ppm] of CorX$_P$ (1c$_B$).

Residue	Cα	Cβ	Cγ	Cδ	Cε	Cζ	Others (indole)
**Lys^1**	50.86	29.34	26.07	26.58	38.89		
**Phe^2**	55.14	37.18		129.35	128.67	126.62	
**Trp^3**	52.75	27.78					111.23,122.44. 119.09,119.61
**Val^4**	62.49	28.39	20.08;21.96				
**Orn^5**	53.94	27.44	25.21	38.89			
**Leu^6**	50.86	39.24	24.02	22.65			
**Ile^7**	59.24	35.13	20.17;15.81	11.19			
**Cys^8**	50.18	38.21					
**D-Thr^9**	60.95	66.57	20.25				
**Gly^{10}**	42.66						

6.5 *M*-ansamer of cortinarin X (1c$_{A,}$ CorX$_{M}$)

Table S11. ^{1}H-NMR chemical shifts [ppm] of CorX$_{M}$ (1c$_{A}$).

Residue	HN	Hα	Hβ	Hγ	Hδ	Hε	Hζ	Others (indole)
**Lys^{1}**	8.34	3.97	1.54, 1.32	1.16	1.52	2.77	7.71	
**Phe^{2}**	7.43	4.90	3.06, 2.74		7.17	7.19	7.14	
**Trp^{3}**	8.96	4.95	3.19, 3.09					11.23, 7.61, 7.20, 7.05, 6.94
**Val^{4}**	8.07	4.08	2.30	0.89, 0.84				
**Orn^{5}**	8.00	3.37	1.58, 1.66	1.44, 1.17	2.70	7.67		
**Leu^{6}**	7.65	3.78	1.71, 1.51	1.41	0.84, 0.77			
**Ile^{7}**	7.02	4.35	1.74	1.38, 0.96; 0.66	0.72			
**Cys^{8}**	8.75	5.35	3.29, 2.66					
**D-Thr^{9}**	8.65	4.47	4.14	5.76; 1.12				
**Gly^{10}**	8.26	3.83, 3.60						`

Table S12. ^{1}H, ^{13}C-HSQC chemical shifts [ppm] of CorX$_{M}$ (1c$_{A}$).

Residue	Cα	Cβ	Cγ	Cδ	Cε	Cζ	Others (indole)
**Lys^{1}**	53.76	30.84	22.98	24.43	39.05		
**Phe^{2}**	53.42	40.42		129.70	129.36	126.63	
**Trp^{3}**	56.50	27.76					111.23,122.52, 119.1,120.47
**Val^{4}**	56.84	31.52	20.24;23.66				
**Orn^{5}**	56.19	26.74	22.97	39.05			
**Leu^{6}**	53.42	39.74	25.64	18.18;21.60			
**Ile^{7}**	56.84	37.00	25.03;14.76	11.80			
**Cys^{8}**	53.42	40.42					
**D-Thr^{9}**	57.52	66.08	17.16				
**Gly^{10}**	44.52						

6.6 *P*-ansamer of cortinarin X-Ala (S20$_A$, CorX-Ala$_P$)

Table S13. ^{1}H-NMR chemical shifts [ppm] of CorX-Ala$_P$ (S20$_A$).

Residue	HN	Hα	Hβ	Hγ	Hδ	Hε	Hζ	Others (indole)
**Ala^1**	7.09	4.13	0.99					
**Phe^2**	8.26	4.51	2.96, 2.56		7.07	7.14	7.14	
**Trp^3**	7.61	4.87	3.2					11.21, 7.85, 7.21, 7.08, 7.01
**Val^4**	8.46	3.78	2.19	1.0, 0.92				
**Ala^5**	8.45	4.03	1.35					
**Leu^6**	7.17	4.5	1.44	1.66	0.74, 0.68			
**Ile^7**	8.55	4	1.81	1.56, 1.04	0.88			
**Cys^8**	8.35	4.81	3.34, 3.19					
**D-Thr^9**	7.54	4.01	3.89	1.03				
**Gly^{10}**	8.40	3.73, 3.62						

Table S14. ^{1}H, ^{13}C-HSQC chemical shifts [ppm] of CorX-Ala$_P$ (S20$_A$).

Residue	Cα	Cβ	Cγ	Cδ	Cε	Cζ	Others (indole)
**Ala^1**	47.60	19.89					
**Phe^2**	55.47	40.42		129.36	128.34	126.72	
**Trp^3**	52.39	37.68					111.23,122.181,119.10,119.78
**Val^4**	61.63	29.13	19.89				
**Ala^5**	49.66	17.16					
**Leu^6**	55.47	26.05	24.00	22.97; 23.66			
**Ile^7**	58.75	35.29	25.71;15.79	11.34			
**Cys^8**	51.02	38.71					
**D-Thr^9**	60.26	65.73	19.89				
**Gly^{10}**	43.16						

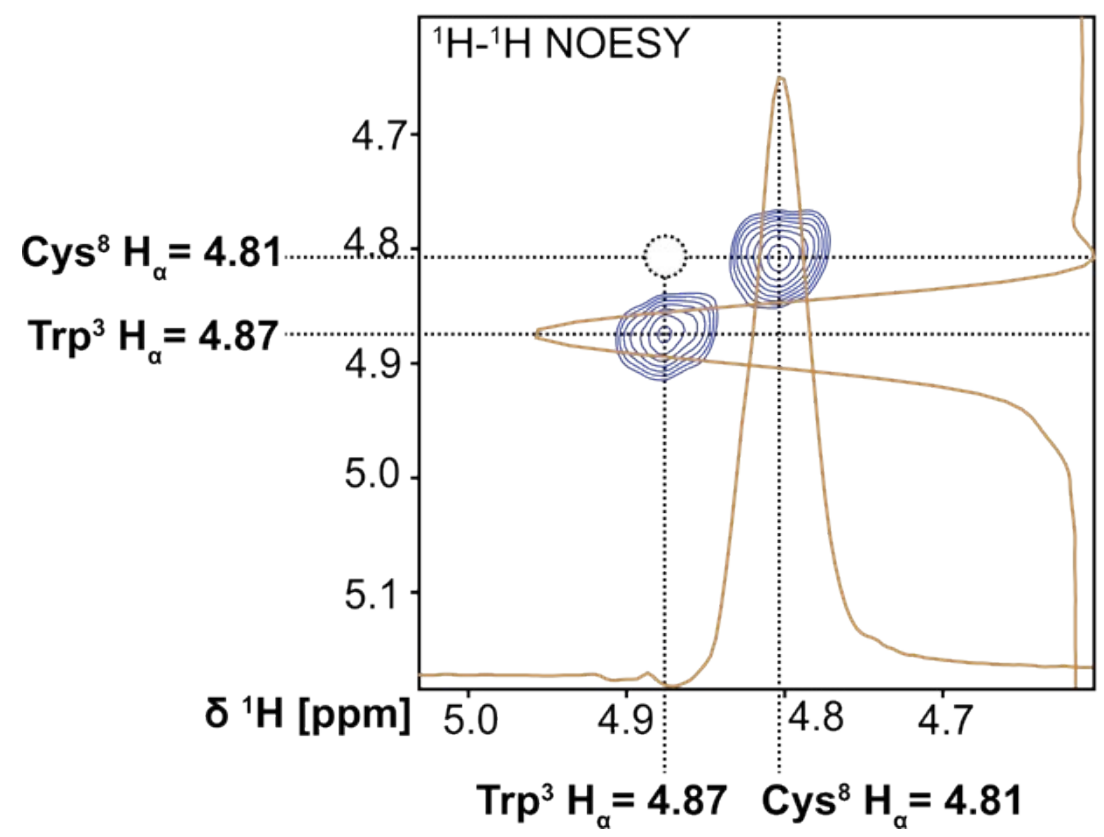

Figure S8. Diagnostic NOE signals of the Hα atoms of Cys⁸ and Trp³ in NMR spectra of CorX-Ala$_P$ (blue contours). NOESY spectra were acquired with a mixing time of 600 ms. The orange curves depict representative 1D projections/slices along the diagonal peaks in the direct and indirect dimension. The position of the theoretical, but missing NOE cross peak between Hα atoms of Cys⁸ and Trp³ in CorX-Ala$_P$ is indicated with a dashed circle. The product S20$_A$ was thus determined to be the *P*-ansamer and designated CorX-Ala$_P$.

6.7 *M*-ansamer of cortinarin X-Ala (S20$_B$, CorX-Ala$_M$)

Table S15. ^{1}H-NMR chemical shifts [ppm] of CorX-Ala$_M$ (S20$_B$).

Residual	NH	Hα	Hβ	Hγ	Hδ	Hε	Hζ	Others (indole)
**Ala^1**	8.47	4.05	1.04					
**Phe^2**	7.41	4.87	3.03, 2.76		7.17	7.21	7.15	
**Trp^3**	8.96	4.92	3.10, 3.17					11.22,7.19, 7.04, 7.93, 7.63
**Val^4**	8.08	4.03	2.33	0.84, 0.88				
**Ala^5**	8.18	3.45	1.19					
**Leu^6**	7.60	3.82	1.73	1.50	0.83, 0.75			
**Ile^7**	7.16	4.36	1.73	1.40, 0.95; 0.66	0.72			
**Cys^8**	8.74	5.35	3.29, 2.62					
**D-Thr^9**	8.78	4.47	4.14	1.14; 5.80				
**Gly^{10}**	8.28	3.58, 3.85						

Table S16. ^{1}H, ^{13}C-HSQC chemical shifts [ppm] of CorX-Ala$_M$ (S20$_B$).

Residue	Cα	Cβ	Cγ	Cδ	Cε	Cζ	Others (indole)
**Ala^1**	49.46	17.79					
**Phe^2**	53.40	40.73		129.92	128.38	126.50	
**Trp^3**	56.48	27.37					111.09,122.56, 118.97,120.68
**Val^4**	56.82	31.31	18.30;19.67				
**Ala^5**	51.86	16.25					
**Leu^6**	53.05	25.02	24.81	18.30;21.38			
**Ile^7**	56.65	37.47	24.81;14.88	11.80			
**Cys^8**	53.91	40.21					
**D-Thr^9**	57.33	66.24	17.27				
**Gly^{10}**	44.67						

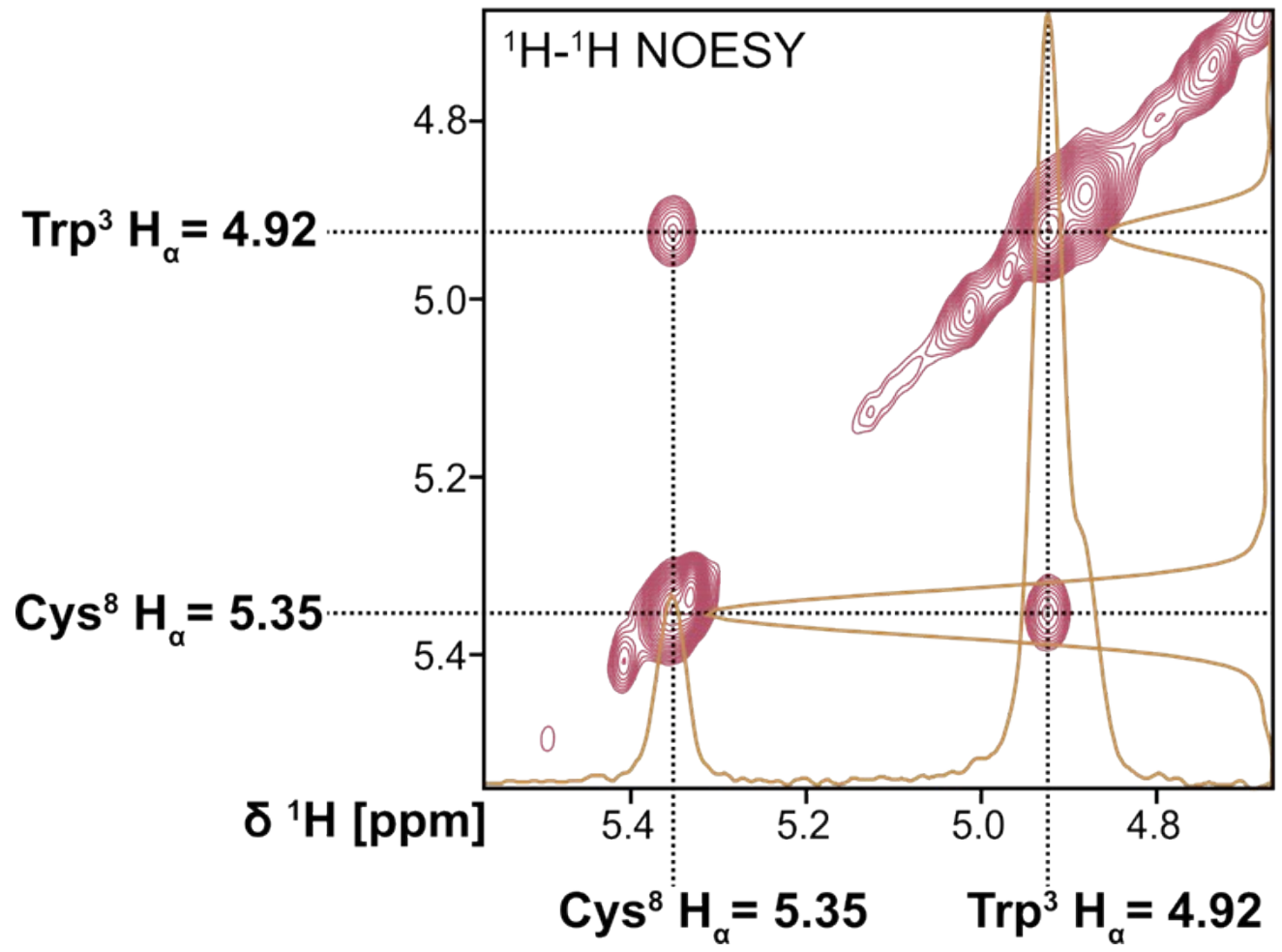

Figure S9. Diagnostic NOE signals of the Hα atoms of Cys^{8} and Trp^{3} in NMR spectra of CorX-Ala$_{M}$ (red contours). NOESY spectra were acquired with a mixing time of 600 ms. The orange curves depict representative 1D projections/slices along the diagonal peaks in the direct and indirect dimension. The position of the theoretical, but missing NOE cross peak between Hα atoms of Cys^{8} and Trp^{3} in CorX-Ala$_{M}$ is indicated with a dashed circle. The product S20$_{A}$ was thus determined to be the *M*-ansamer and designated CorX-Ala$_{M}$.

7 CYANA peptide structure calculations

7.1 Preparation

7.1.1 CYANA library files for the non-canonical amino acids

CYLIB [8] and Automated Force Field Topology Builder (ATB)[9] were used to generate CYANA library files for the non-canonical amino acids, i.e H-L-Trp(2-SMe)-OH, H-L-Cys(S-CH=CH_2)-OH, H-Gly-NHMe, Ac-L-Lys-OH, H-D-Thr-OH (see **Tables S16-S20**).

Table S17. CYANA library file for H-L-Trp(2-SMe)-OH.

RESIDUE TRPS 7 33 3 32												
1	OMEGA	0	0	0	2	1	3	4	0			□
2	PHI	0	0	0	1	3	5	31	0			□
3	CHI1	0	0	0	3	5	7	8	30			□
4	CHI2	0	0	0	5	7	8	9	27			□
5	CHI3	0	0	0	8	9	12	13	17			□
6	CHI4	0	0	0	9	12	13	14	17			□
7	PSI	0	0	0	3	5	31	33	0			□
1	C	C_BYL	0	0	1.4398	-1.2028	0.0553	2	3	0	0	0
2	O	O_BYL	0	0	1.9018	-0.2058	0.6081	1	0	0	0	0
3	N	N_AMI	0	0	0.1620	-1.3100	-0.2940	1	4	5	0	0
4	H	H_AMI	0	0	-0.1467	-2.1305	-0.7320	3	0	0	0	0
5	CA	C_ALI	0	0	-0.7780	-0.2520	-0.0450	3	6	7	31	0
6	HA	H_ALI	0	0	-0.8040	-0.0530	1.0710	5	0	0	0	0
7	CB	C_ALI	0	0	-0.4360	1.0710	-0.7600	5	8	28	29	0
8	CG	C_ARO	0	0	0.8040	1.6820	-0.2660	7	9	18	0	0
9	CD1	C_ARO	0	0	1.0030	2.3030	0.9770	8	10	12	0	0
10	NE1	N_AMO	0	0	2.3110	2.8170	1.0310	9	11	19	0	0
11	HE1	H_AMI	0	0	2.7270	3.2150	1.8320	10	0	0	0	0
12	SE1	DUMMY	0	0	-0.0640	2.3850	2.2830	9	13	0	0	0
13	CZ1	DUMMY	0	0	-0.3820	4.1010	2.4650	12	14	15	16	0
14	HZ11	DUMMY	0	0	-1.1090	4.1760	3.3050	13	0	0	0	17
15	HZ12	DUMMY	0	0	0.5340	4.6770	2.7250	13	0	0	0	17
16	HZ13	DUMMY	0	0	-0.8350	4.5510	1.5520	13	0	0	0	17
17	QZ1	PSEUD	0	0	-0.4700	4.4680	2.5273	0	0	0	0	0
18	CD2	C_ARO	0	0	2.0400	1.7910	-1.0060	8	19	22	0	0
19	CE2	C_ARO	0	0	2.9650	2.5060	-0.1560	10	18	20	0	0
20	CZ2	C_ARO	0	0	4.2720	2.7940	-0.5910	19	21	25	0	0
21	HZ2	H_ARO	0	0	4.9740	3.3400	0.0530	20	0	0	0	0
22	CE3	C_ARO	0	0	2.4530	1.3790	-2.2770	18	23	27	0	0
23	CZ3	C_ARO	0	0	3.7440	1.6660	-2.6880	22	24	25	0	0
24	HZ3	H_ARO	0	0	4.0860	1.3440	-3.6830	23	0	0	0	0
25	CH2	C_ARO	0	0	4.6390	2.3640	-1.8540	20	23	26	0	0
26	HH2	H_ARO	0	0	5.6560	2.5690	-2.2220	25	0	0	0	0
27	HE3	H_ARO	0	0	1.7580	0.8360	-2.9320	22	0	0	0	0
28	HB2	H_ALI	0	0	-0.3660	0.8940	-1.8680	7	0	0	0	30

29	HB3	H_ALI	0	0	-1.2980	1.7730	-0.5830	7	0	0	0	30
30	QB	PSEUD	0	0	-0.8320	1.3335	-1.2255	0	0	0	0	0
31	C	C_BYL	0	0	-2.1820	-0.6690	-0.5620	5	32	33	0	0
32	O	O_BYL	0	0	-2.3582	-1.7651	-1.0915	31	0	0	0	0
33	N	N_AMI	0	0	-3.1561	0.2198	-0.3964	31	0	0	0	0

Table S18. CYANA library file for H-L-Cys(S-CH=CH$_2$)-OH.

RESIDUE	CYSS	6	20	3	19							
1	OMEGA	0	0	0	2	1	3	4	0			□
2	PHI	0	0	0	1	3	5	18	0			□
3	CHI1	0	0	0	3	5	6	7	16			□
4	CHI2	0	0	0	5	6	7	8	13			□
5	CHI3	0	0	0	6	7	8	9	13			□
6	PSI	0	0	0	3	5	18	20	0			□
1	C	C_BYL	0	0	2.2784	-0.0084	-0.0085	2	3	0	0	0
2	O	O_BYL	0	0	2.4454	-0.8835	-0.8565	1	0	0	0	0
3	N	N_AMI	0	0	1.0820	0.3170	0.4700	1	4	5	0	0
4	H	H_AMI	0	0	1.0132	1.0238	1.1454	3	0	0	0	0
5	CA	C_ALI	0	0	-0.1270	-0.3500	0.0160	3	6	17	18	0
6	CB	C_ALI	0	0	-0.1150	-1.8650	0.3120	5	7	14	15	0
7	SG	S_RED	0	0	1.1770	-2.7240	-0.6700	6	8	0	0	0
8	CD	DUMMY	0	0	1.0650	-4.3810	-0.0600	7	9	13	0	0
9	CE	DUMMY	0	0	0.2770	-4.8830	0.8970	8	10	11	0	0
10	HE2	DUMMY	0	0	0.3490	-5.9360	1.1550	9	0	0	0	12
11	HE3	DUMMY	0	0	-0.4540	-4.2960	1.4460	9	0	0	0	12
12	QE	PSEUD	0	0	-0.0525	-5.1160	1.3005	0	0	0	0	0
13	HD	DUMMY	0	0	1.7800	-5.0180	-0.5790	8	0	0	0	0
14	HB2	H_ALI	0	0	0.0790	-2.0230	1.3780	6	0	0	0	16
15	HB3	H_ALI	0	0	-1.0900	-2.2980	0.0650	6	0	0	0	16
16	QB	PSEUD	0	0	-0.5055	-2.1605	0.7215	0	0	0	0	0
17	HA	H_ALI	0	0	-0.2190	-0.1780	-1.0600	5	0	0	0	0
18	C	C_BYL	0	0	-1.3130	0.2770	0.7770	5	19	20	0	0
19	O	O_BYL	0	0	-1.1309	1.1756	1.5969	18	0	0	0	0
20	N	N_AMI	0	0	-2.5163	-0.2098	0.4919	18	0	0	0	0

Table S19. CYANA library file for H-Gly-NHMe.

RESIDUE	CGly	5	17	3	17							
1	OMEGA	0	0	0	2	1	3	4	0			□
2	PHI	0	0	0	1	3	5	9	0			□
3	PHI2	0	0	0	3	5	9	11	0			□
4	PHI3	0	0	0	5	9	11	13	0			□
5	PHI4	0	0	0	9	11	13	16	0			□
1	C	C_BYL	0	0	-0.4106	0.5699	0.4826	2	3	0	0	0

2	O	O_BYL	0	0	-0.5606	1.0396	1.6094	1	0	0	0	0
3	N	N_AMI	0	0	0.7740	0.2080	0.0010	1	4	5	0	0
4	H	H_AMI	0	0	0.8293	-0.1605	-0.9054	3	0	0	0	0
5	CA	C_ALI	0	0	1.9850	0.3450	0.7830	3	6	7	9	0
6	HA2	H_ALI	0	0	2.1390	1.3910	1.0780	5	0	0	0	8
7	HA3	H_ALI	0	0	1.9200	-0.2420	1.7080	5	0	0	0	8
8	QA	PSEUD	0	0	2.0295	0.5745	1.3930	0	0	0	0	0
9	C	C_BYL	0	0	3.1640	-0.1590	-0.0580	5	10	11	0	0
10	O	O_BYL	0	0	2.9780	-0.7580	-1.1220	9	0	0	0	0
11	N2	DUMMY	0	0	4.3790	0.0800	0.4740	9	12	13	0	0
12	H4	DUMMY	0	0	4.4360	0.6330	1.3200	11	0	0	0	0
13	C3	DUMMY	0	0	5.6250	-0.3510	-0.1460	11	14	15	16	0
14	H5	DUMMY	0	0	6.3440	-0.6130	0.6360	13	0	0	0	17
15	H6	DUMMY	0	0	5.4250	-1.2300	-0.7650	13	0	0	0	17
16	H7	DUMMY	0	0	6.0540	0.4340	-0.7750	13	0	0	0	17
17	Q3	PSEUD	0	0	5.9410	-0.4697	-0.3013	0	0	0	0	0

Table S20. CYANA library file for Ac-L-Lys-OH.

RESIDUE NLYS 9 34 1 33												□
1	OMEGA	0	0	0	2	1	3	4	0			□
2	CHI2	0	0	0	1	2	4	5	8			□
3	PHI	0	0	0	2	1	10	32	0			□
4	CHI3	0	0	0	1	10	11	12	30			□
5	CHI4	0	0	0	10	11	12	13	27			□
6	CHI5	0	0	0	11	12	13	14	24			□
7	CHI6	0	0	0	12	13	14	15	21			□
8	CHI7	0	0	0	13	14	15	16	18			□
9	PHI2	0	0	0	1	10	32	34	0			□
1	N	N_AMI	0	0	1.6130	1.9100	0.3590	2	9	10	0	0
2	C3	DUMMY	0	0	2.4340	2.6010	-0.4770	1	3	4	0	0
3	O2	DUMMY	0	0	2.6490	2.2540	-1.6430	2	0	0	0	0
4	C4	DUMMY	0	0	3.0760	3.8420	0.1210	2	5	6	7	0
5	H4	DUMMY	0	0	2.9170	3.9340	1.2000	4	0	0	0	8
6	H5	DUMMY	0	0	4.1510	3.8200	-0.0820	4	0	0	0	8
7	H6	DUMMY	0	0	2.6650	4.7270	-0.3740	4	0	0	0	8
8	Q4	PSEUD	0	0	3.2443	4.1603	0.2480	0	0	0	0	0
9	H	H_AMI	0	0	1.3630	2.3050	1.2590	1	0	0	0	0
10	CA	C_ALI	0	0	0.7830	0.7970	-0.0700	1	11	31	32	0
11	CB	C_ALI	0	0	1.3290	-0.5760	0.3910	10	12	28	29	0
12	CG	C_ALI	0	0	2.7120	-0.9230	-0.1730	11	13	25	26	0
13	CD	C_ALI	0	0	3.1870	-2.3110	0.2750	12	14	22	23	0
14	CE	C_ALI	0	0	4.5840	-2.6670	-0.2390	13	15	19	20	0
15	NZ	N_AMO	0	0	4.9690	-4.0220	0.1940	14	16	17	0	0
16	HZ1	H_AMI	0	0	5.9020	-4.2300	-0.1640	15	0	0	0	18
17	HZ2	H_AMI	0	0	5.0740	-4.0200	1.2100	15	0	0	0	18

18	QZ	PSEUD	0	0	5.4880	-4.1250	0.5230	0	0	0	0	0
19	HE2	H_ALI	0	0	4.5820	-2.6570	-1.3400	14	0	0	0	21
20	HE3	H_ALI	0	0	5.2940	-1.8890	0.0810	14	0	0	0	21
21	QE	PSEUD	0	0	4.9380	-2.2730	-0.6295	0	0	0	0	0
22	HD2	H_ALI	0	0	3.1870	-2.3560	1.3740	13	0	0	0	24
23	HD3	H_ALI	0	0	2.4790	-3.0800	-0.0660	13	0	0	0	24
24	QD	PSEUD	0	0	2.8330	-2.7180	0.6540	0	0	0	0	0
25	HG2	H_ALI	0	0	2.6800	-0.8790	-1.2700	12	0	0	0	27
26	HG3	H_ALI	0	0	3.4380	-0.1630	0.1460	12	0	0	0	27
27	QG	PSEUD	0	0	3.0590	-0.5210	-0.5620	0	0	0	0	0
28	HB2	H_ALI	0	0	1.3590	-0.5850	1.4890	11	0	0	0	30
29	HB3	H_ALI	0	0	0.6070	-1.3480	0.0920	11	0	0	0	30
30	QB	PSEUD	0	0	0.9830	-0.9665	0.7905	0	0	0	0	0
31	HA	H_ALI	0	0	0.7460	0.8210	-1.1630	10	0	0	0	0
32	C	C_BYL	0	0	-0.6230	1.0120	0.5200	10	33	34	0	0
33	O	O_BYL	0	0	-0.8702	1.9953	1.2164	32	0	0	0	0
34	N	N_AMI	0	0	-1.5288	0.0834	0.2312	32	0	0	0	0

Table S21. CYANA library file for H-D-Thr-OH.

RESIDUE	ADTH	6	18	3	17							
1	OMEGA	0	0	0	2	1	3	4	0			□
2	PHI	0	0	0	1	3	5	16	0			□
3	CHI1	0	0	0	3	5	6	7	14			□
4	CHI2	0	0	0	5	6	7	8	11			□
5	CHI3	0	0	0	5	6	12	13	13			□
6	PSI	0	0	0	3	5	16	18	0			□
1	C	C_BYL	0	0	1.5611	-2.2337	-0.6595	2	3	0	0	0
2	O	O_BYL	0	0	2.4121	-1.6263	-1.3075	1	0	0	0	0
3	N	N_AMI	0	0	0.4020	-1.6920	-0.3000	1	4	5	0	0
4	H	H_AMI	0	0	-0.2361	-2.2273	0.2164	3	0	0	0	0
5	CA	C_ALI	0	0	0.0540	-0.3100	-0.6580	3	6	15	16	0
6	CB	C_ALI	0	0	1.0850	0.6480	-0.0580	5	7	12	14	0
7	CG2	C_ALI	0	0	2.4730	0.3130	-0.6080	6	8	9	10	0
8	HG21	H_ALI	0	0	3.2080	0.9950	-0.1800	7	0	0	0	11
9	HG22	H_ALI	0	0	2.7310	-0.7120	-0.3420	7	0	0	0	11
10	HG23	H_ALI	0	0	2.4690	0.4170	-1.6930	7	0	0	0	11
11	QG2	PSEUD	0	0	2.8027	0.2333	-0.7383	0	0	0	0	0
12	OG1	O_HYD	0	0	1.0910	0.5110	1.3650	6	13	0	0	0
13	HG1	H_OXY	0	0	1.7520	1.1300	1.7030	12	0	0	0	0
14	HB	H_ALI	0	0	0.8270	1.6730	-0.3230	6	0	0	0	0
15	HA	H_ALI	0	0	0.0490	-0.2060	-1.7430	5	0	0	0	0
16	C	C_BYL	0	0	-1.3130	0.0200	-0.1160	5	17	18	0	0
17	O	O_BYL	0	0	-1.9575	-0.8142	0.5177	16	0	0	0	0
18	N	N_AMI	0	0	-1.7626	1.2457	-0.3646	16	0	0	0	0

7.1.2 Upper limit distance restraints for macrolactamization and tryptathionine formation in CYANA

Upper limit distance (upl. file) for macrolactamization and tryptathionine formation were obtained via CYANA Lib Linker (atb.uq.edu.au/cyana_linker/)[10], using the library files in **Section 7.1.1**.

7.1.3 NOE assignments originate from CcpNmr analysis

The manually assigned chemical shifts (prot. file) and NOESY peak lists (peak. file) were exported from CcpNmr 2.5.2.[7]

Table S22. Assignment of NOE signals for CorX$_P$ (1c$_B$). In atom column, Q represents chemically equivalent atoms. Upper limited distance is from CYANA analysis.

Residue	Atom	Residue	Atom	Upper limited distance (Å)
Trp	Hε1	Cys	Hβ3	4.51
Trp	Hε1	Trp	Hζ2	4.03
Trp	Hε1	Cys	Hβ2	3.82
Leu	Hα	Ile	HN	3.47
Trp	Hα	Val	HN	3.43
Phe	HN	Phe	Hα	3.99
Cys	HN	Cys	Hα	4.20
Phe	Hα	Cys	HN	4.21
Ile	HN	Ile	Hα	4.36
Ile	HN	Ile	Hβ	3.78
Leu	Hβ2	Ile	HN	4.10
Ile	HN	Ile	Qδ1	4.35
Val	HN	Val	Qγ2	3.81
Val	HN	Val	Qγ1	4.51
Val	HN	Orn	Hβ2	4.54
Val	HN	Val	Hβ	3.73
Trp	Hβ3	Val	HN	4.24
Trp	Hβ2	Val	HN	3.50
Val	HN	Val	Hα	3.70
Orn	HN	Orn	Hα	3.71
Val	Hα	Orn	HN	3.56
Gly	HN	Gly	Hα3	4.02
Gly	HN	Gly	Hα2	3.89
D-Thr	Hα	Gly	HN	3.78
Ile	Hα	Cys	HN	3.30
Lys	Hα	Phe	HN	3.66
Phe	HN	Phe	Hβ2	4.53
Phe	HN	Phe	Hβ3	3.90
Val	Hβ	Orn	HN	4.14
Orn	HN	Orn	Hβ2	4.60
Orn	HN	Orn	Hβ3	4.33
Orn	HN	Orn	Hγ2	4.45

Val	Qγ1	Orn	HN	4.50
Lys	Hγ2	Phe	HN	4.62
Lys	Hβ3	Phe	HN	4.37
Lys	Hβ2	Phe	HN	4.01
Cys	HN	Cys	Hβ2	4.68
Leu	Hβ3	Ile	HN	4.62
Cys	HN	Cys	Hβ3	3.95
Ile	Qδ1	Cys	HN	4.33
Phe	Hα	Trp	HN	3.39
Trp	HN	Trp	Hα	3.90
Trp	Hα	Trp	Hε3	3.13
Trp	HN	Trp	Hβ3	4.13
Phe	Hβ2	Trp	HN	4.44
Trp	Hβ2	Trp	Hε3	3.85
Trp	Hβ3	Trp	Hε3	4.65
D-Thr	HN	D-Thr	Hα	4.13
D-Thr	HN	D-Thr	Hβ	4.07
Cys	Hα	D-Thr	HN	3.62
D-Thr	HN	D-Thr	Hγ1	4.64
D-Thr	HN	D-Thr	Hγ21	4.20
Ile	Qδ1	D-Thr	HN	4.04
Leu	HN	Leu	Hα	4.29
Phe	Hα	Phe	Qδ	4.32
Phe	Hβ2	Phe	Qδ	4.44
Phe	Hβ3	Phe	Qδ	4.21
Lys	HN	Gly	Hα3	4.65
Lys	HN	Gly	Hα2	4.64
Lys	HN	Lys	Hα	4.38
Leu	HN	Leu	Hγ	4.83
Leu	HN	Leu	Hβ2	4.72
Phe	Qε	Ile	Qδ1	4.82
Lys	HN	Lys	Hγ2	4.38
Lys	HN	Lys	Hβ2	4.63
D-Thr	Hγ21	D-Thr	Hγ1	4.54
D-Thr	Hβ	D-Thr	Hγ1	4.19
Trp	Hα	Trp	Hβ2	3.65
Trp	Hα	Trp	Hβ3	4.15
Cys	Hα	Cys	Hβ2	3.68
Cys	Hα	Cys	Hβ3	4.06
Phe	Hα	Phe	Hβ2	3.80
Phe	Hα	Phe	Hβ3	4.25
Leu	Hα	Leu	Hβ3	3.96
Leu	Hα	Leu	Hβ2	4.17
Leu	Hα	Leu	Hγ	4.71

Leu	Hα	Leu	Qδ2	3.93
Lys	Hα	Lys	Hγ2	3.94
Lys	Hα	Lys	Hβ3	4.11
Lys	Hα	Lys	Hβ2	3.69
D-Thr	Hα	D-Thr	Hγ21	3.70
Orn	Hα	Orn	Hγ2	3.72
Ile	Hα	Ile	Hβ	3.79
Orn	Hα	Orn	Hβ2	3.76
Ile	Hα	Ile	Qδ1	3.65
D-Thr	Hβ	D-Thr	Hγ21	3.56
Val	Hα	Val	Qγ1	3.76
Val	Hα	Val	Qγ2	3.67
Val	Hα	Val	Hβ	4.22
Cys	Hβ2	Cys	Hβ3	2.98
Phe	Hβ2	Phe	Hβ3	3.02
Lys	HN	Gly	HN	3.87
Trp	Hζ3	Trp	Hε3	3.44
Orn	Hγ2	Orn	Hδ2	3.78
Val	Hβ	Val	Qγ1	3.75
Val	Hβ	Val	Qγ2	3.66
Orn	Hβ2	Orn	Hβ3	2.80
Lys	Hβ3	Lys	Hγ2	2.40
Orn	Hβ3	Orn	Hγ2	2.73
Orn	HN	Leu	HN	3.87
Orn	Hα	Leu	HN	4.60
Trp	Hα	Trp	Hζ3	5.40
Lys	HN	Lys	Hβ3	4.20
Orn	Hβ2	Orn	Hδ2	4.13
Orn	Hβ3	Orn	Hδ2	4.13
Trp	HN	Trp	Hβ2	4.86
Phe	HN	Ile	Qδ1	4.91
Phe	Qδ	Ile	Hα	5.04
Trp	Hα	Val	Qγ2	4.95
Phe	Hα	Ile	Hα	4.22
Trp	Hβ2	Leu	HN	5.50
Lys	Hα	Lys	Hγ3	4.43
Trp	Hβ3	Leu	Hβ2	4.30
Trp	Hβ3	Leu	Hβ3	4.78
Cys	Hα	D-Thr	Hγ21	5.31
Leu	Hα	Ile	Hα	4.68
D-Thr	Hβ	Gly	HN	4.46
Val	Hα	Leu	HN	5.26
Lys	HN	D-Thr	Hα	5.33
Leu	HN	Leu	Hβ3	5.29

Table S23. Assignment of NOE signals for CorX$_M$ (1c$_A$). In atom column, Q represents chemically equivalent atoms. Upper limited distance is from CYANA analysis.

Residue	Atom	Residue	Atom	Upper limited distance (Å)
Trp	Hε1	Cys	Hβ3	4.09
Trp	Hε1	Cys	Hβ2	4.05
Trp	HN	Val	Qγ2	5.01
Trp	HN	Trp	Hβ2	4.62
Phe	Hβ3	Trp	HN	4.41
Phe	Hβ2	Trp	HN	4.79
Ile	Hα	Cys	HN	2.88
Cys	HN	Cys	Hα	3.87
Trp	Hα	Cys	HN	5.34
Cys	HN	Cys	Hβ3	3.88
Cys	HN	Cys	Hβ2	4.25
Ile	Hβ	Cys	HN	4.57
Ile	HN	Cys	HN	5.15
Phe	HN	D-Thr	HN	4.98
D-Thr	HN	Gly	HN	4.97
Cys	Hα	D-Thr	HN	3.49
Trp	Hα	D-Thr	HN	4.83
D-Thr	HN	D-Thr	Hα	4.46
Phe	HN	Phe	Hα	4.05
Phe	Hα	Phe	Qδ	4.48
D-Thr	HN	D-Thr	Hβ	5.11
Cys	Hβ3	D-Thr	HN	4.93
Ile	Hγ12	Cys	HN	4.44
Ile	Hγ13	Cys	HN	4.83
D-Thr	HN	D-Thr	Hγ22	4.20
Ile	Qδ1	Cys	HN	5.02
Ile	Qγ2	Cys	HN	4.19
Lys	HN	Lys	Hα	3.89

Lys	HN	Gly	Hα3	3.22
Lys	HN	Gly	Hα2	3.93
Lys	HN	Lys	Hβ2	3.67
Lys	HN	Lys	Hγ2	3.78
Lys	HN	Phe	Qδ	5.28
Lys	HN	Phe	HN	3.35
Phe	Hα	Trp	HN	3.72
D-Thr	Hα	Gly	HN	3.28
D-Thr	Hβ	Gly	HN	3.75
Val	H	Ile	HN	4.82
D-Thr	Hγ1	Gly	HN	3.69
Gly	HN	Gly	Hα2	3.03
Gly	HN	Gly	Hα3	3.34
D-Thr	Hγ22	Gly	HN	3.56
Val	HN	Cys	Hα	4.35
Trp	Hα	Val	H	3.18
Val	H	Val	Hα	3.74
Val	Hα	Orn	HN	3.56
Trp	Hβ2	Val	HN	5.36
Trp	Hβ3	Val	HN	5.03
Val	HN	Cys	Hβ2	5.15
Val	HN	Val	Qγ2	4.18
Val	HN	Val	Qγ1	3.72
Val	Qγ2	Orn	HN	4.82
Val	Qγ1	Orn	HN	4.26
Orn	HN	Orn	Hβ3	4.48
Orn	HN	Orn	Hβ2	4.07
Val	Hβ	Orn	HN	3.62
Leu	HN	Leu	Hα	3.34
Trp	Hδ3	Val	Hα	4.44
Val	Hα	Leu	HN	5.41

Trp	Hα	Trp	Hδ3	5.30
Lys	Hα	Phe	HN	3.99
Phe	HN	Gly	Hα3	4.52
Trp	Hζ3	Trp	Hδ3	3.47
Leu	HN	Leu	Hβ3	3.40
Orn	Hβ2	Leu	HN	3.96
Leu	HN	Leu	Hβ2	3.57
Val	Hβ	Leu	HN	5.24
Trp	Hβ2	Trp	Hδ3	3.19
Trp	Hβ3	Trp	Hδ3	3.71
Leu	HN	Leu	Qδ1	3.73
Lys	Hβ3	Phe	HN	4.57
Lys	Hβ2	Phe	HN	4.34
Phe	HN	D-Thr	Hγ22	4.69
Phe	HN	Phe	Hβ2	3.51
Phe	HN	Phe	Hβ3	4.23
Phe	HN	Gly	Hα2	5.32
Phe	HN	Phe	Qδ	4.34
Phe	HN	Cys	Hα	5.50
Ile	HN	Ile	Hα	4.19
Leu	Hα	Ile	HN	4.57
Phe	Hβ3	Phe	Qδ	3.73
Phe	Hβ2	Phe	Qδ	3.59
Trp	Hβ2	Trp	Hζ3	5.35
Lys	Hδ3	Phe	Qδ	4.53
Lys	Hβ2	Phe	Qδ	4.46
Lys	Hγ2	Phe	Qδ	4.85
Phe	Qδ	Val	Qγ2	4.02
Phe	Qδ	Val	Qγ1	4.20
Phe	Qδ	Ile	Qδ1	5.32
Phe	Qδ	Ile	Qγ2	4.70

Val	Qγ1	Ile	HN	3.98
Ile	HN	Ile	Qδ1	5.50
Ile	HN	Ile	Qγ2	4.46
Ile	HN	Ile	Hγ12	5.05
Ile	HN	Ile	Hβ	4.18
D-Thr	Hγ22	D-Thr	Hγ1	3.69
D-Thr	Hβ	D-Thr	Hγ1	3.09
D-Thr	Hα	D-Thr	Hγ1	3.91
Cys	Hα	D-Thr	Hγ22	5.05
Ile	Qγ2	Cys	Hα	5.24
Cys	Hα	Cys	Hβ2	3.81
Trp	Hβ3	Cys	Hα	4.86
Trp	Hα	Cys	Hα	3.23
Trp	Hα	Val	Qγ1	4.96
Trp	Hα	Cys	Hβ2	4.39
Phe	Hα	Phe	Hβ2	4.04
Trp	Hα	Trp	Hβ2	3.72
Phe	Hα	Phe	Hβ3	3.79
Trp	Hα	Trp	Hβ3	3.63
Trp	Hα	Val	Hα	5.38
D-Thr	Hα	D-Thr	Hβ	3.04
D-Thr	Hα	Gly	Hα3	5.15
D-Thr	Hα	Gly	Hα2	5.09
D-Thr	Hα	D-Thr	Hγ22	3.78
Ile	Hα	Ile	Hγ13	4.05
Leu	Qδ1	Ile	Hα	4.99
Ile	Hα	Ile	Qδ1	3.97
Ile	Hα	Ile	Qγ2	3.43
Ile	Hα	Ile	Hγ12	3.72
Ile	Hα	Ile	Hβ	3.32
Val	Hα	Val	Qγ2	3.22

Val	Hα	Val	Qγ1	3.55
D-Thr	Hβ	D-Thr	Hγ22	3.00
Lys	Hα	Lys	Hβ2	3.94
Lys	Hα	Lys	Hγ2	3.58
Val	Hα	Orn	Hβ2	5.34
Val	Hα	Leu	Hβ2	5.26
Val	Hα	Val	Hβ	3.17
Leu	Hα	Leu	Hγ	3.11
Leu	Hα	Leu	Hβ3	4.05
Val	Qγ1	Leu	Hα	4.39
Leu	Hα	Leu	Qδ2	3.18
Leu	Hα	Ile	Hα	5.23
Lys	Hγ2	Gly	Hα3	5.24
D-Thr	Hγ22	Gly	Hα2	4.92
Gly	Hα2	Gly	Hα3	2.40
Cys	Hβ2	Cys	Hβ3	2.71
Trp	Hβ2	Trp	Hβ3	2.44
Phe	Hβ2	Phe	Hβ3	2.69
Val	Hβ	Val	Qγ2	3.12
Val	Hβ	Val	Qγ1	3.16
Phe	Hβ2	Val	Qγ2	4.72
Phe	Hβ2	Leu	Qδ1	4.88
Phe	Hβ3	Leu	Qδ1	4.52
Phe	Hβ3	Val	Qγ2	4.17
Trp	Hβ3	D-Thr	Hγ22	4.54
Orn	Hγ3	Orn	Hδ3	4.51
Val	Hβ	Orn	Hβ2	5.35
Ile	Hβ	Ile	Qδ1	3.87
Ile	Hβ	Ile	Qγ2	3.28
Ile	Hβ	Ile	Hγ13	3.61
Ile	Hβ	Ile	Hγ12	3.87

Leu	Hβ3	Leu	Hγ	2.54
Leu	Hβ3	Leu	Qδ1	3.48
Leu	Hγ	Leu	Qδ1	3.07
Leu	Hγ	Leu	Qδ2	3.11
Lys	Hβ2	Lys	Hδ3	2.67
Ile	Hγ12	Ile	Qδ1	3.44
Ile	Qγ2	Ile	Hγ12	4.10
Ile	Hγ12	Ile	Hγ13	3.05
Lys	Hγ3	Lys	Hδ3	3.37
Lys	Hβ2	Lys	Hγ3	3.04
Orn	Hγ2	Orn	Hγ3	3.35
Val	HN	Val	Qγ2	4.20
Val	HN	Val	Qγ1	4.03
Trp	Hα	Val	Qγ2	5.06
Cys	Hβ2	D-Thr	HN	5.50
Cys	Hα	Cys	Hβ3	4.06
Leu	HN	Leu	Qδ2	4.53
Lys	H	Lys	Hδ3	4.34
Trp	H	Trp	Hδ3	5.50
Trp	H	Val	HN	5.49
Phe	Qδ	Val	HN	5.50
Trp	HN	Cys	Hα	5.50
Trp	Hβ2	Cys	Hα	5.50
Phe	Hβ2	Cys	Hα	5.37
Lys	Hα	Phe	Hα	5.50
Cys	Hα	D-Thr	Hα	5.50
Ile	Hα	Cys	Hα	5.50
Phe	HN	Gly	HN	5.02
Ile	HN	Ile	Hγ13	4.81
Cys	HN	D-Thr	Hα	5.37
Cys	HN	D-Thr	Hβ	5.50

Leu	HN	Ile	HN	2.92
Val	HN	Cys	HN	5.50
Trp	HN	D-Thr	HN	4.82

7.2 Structure calculation and results validation

Structure calculation is using manually assigned NOE peak list method on CYANA (Structure calculation using manually assigned NOESY peak lists - CYANA Wiki).[11] A set of structures (**Figure S11 and Figure S13**) were visually inspected with Pymol (The PyMOL Molecular Graphics System, Version 3.1.0 Schrödinger, LLC). Sets of 20 conformers for each ansamer were calculated. These conformers were validated by RMSD (**Table S24 and Table S27**), Ramachandran plot (**Figure S10 and Figure S12**), temperature-dependent chemical shift variations of amide protons (**Table S30 and Table S31**), turn moiety identification (**Table S26 and Table S29**) in the following subsection.

7.2.1 CorX$_M$ (1c$_A$, *M*-ansamer of cortinarin X)

Table S24. Overview of CYANA structure calculation.

str	target function	#	upper rms	limits max	#	lower rms	limits max	#	van der sum	Waals max
1	0.12	0	0.0134	0.18	0	0.0035	0.01	0	0.6	0.09
2	0.12	0	0.0135	0.14	0	0.0190	0.03	0	0.7	0.08
3	0.12	0	0.0135	0.14	0	0.0193	0.03	0	0.7	0.07
4	0.12	0	0.0135	0.14	0	0.0188	0.03	0	0.7	0.07
5	0.13	0	0.0134	0.14	0	0.0000	0.00	0	0.6	0.10
6	0.13	0	0.0136	0.14	0	0.0000	0.00	0	0.6	0.10
7	0.13	0	0.0135	0.15	0	0.0208	0.03	0	0.8	0.08
8	0.13	0	0.0130	0.13	0	0.0123	0.02	0	0.7	0.08
9	0.13	0	0.0130	0.13	0	0.0115	0.02	0	0.7	0.08
10	0.14	0	0.0138	0.14	0	0.0000	0.00	0	0.6	0.09
11	0.14	0	0.0133	0.13	0	0.0125	0.02	0	0.8	0.08
12	0.14	0	0.0136	0.14	0	0.0194	0.03	0	0.8	0.07
13	0.14	0	0.0137	0.15	0	0.0198	0.03	0	0.8	0.08
14	0.14	0	0.0136	0.15	0	0.0000	0.00	0	0.8	0.10
15	0.15	0	0.0152	0.15	0	0.0328	0.05	0	0.7	0.09
16	0.15	0	0.0152	0.15	0	0.0335	0.05	0	0.7	0.09
17	0.15	0	0.0152	0.15	0	0.0332	0.05	0	0.7	0.09
18	0.15	0	0.0152	0.15	0	0.0332	0.05	0	0.7	0.09
19	0.15	0	0.0152	0.15	0	0.0333	0.05	0	0.7	0.09
20	0.15	0	0.0152	0.15	0	0.0329	0.05	0	0.7	0.09
Ave	0.14	0	0.0140	0.15	0	0.0178	0.03	0	0.7	0.09
+/-	9.89E-03	0	0.0008	0.01	0	0.0123	0.02	0	0.1	0.01
Min	0.12	0	0.0130	0.13	0	0.0000	0.00	0	0.6	0.07
Max	0.15	0	0.0152	0.18	0	0.0335	0.05	0	0.8	0.10
Cut				0.20			0.20			0.20

RMSDs for residues 1..10:

Average backbone RMSD to mean : 0.22 +/- 0.08 A (0.13..0.34 A; 20 structures)

Average heavy atom RMSD to mean : 0.74 +/- 0.21 A (0.50..1.18 A; 20structures)

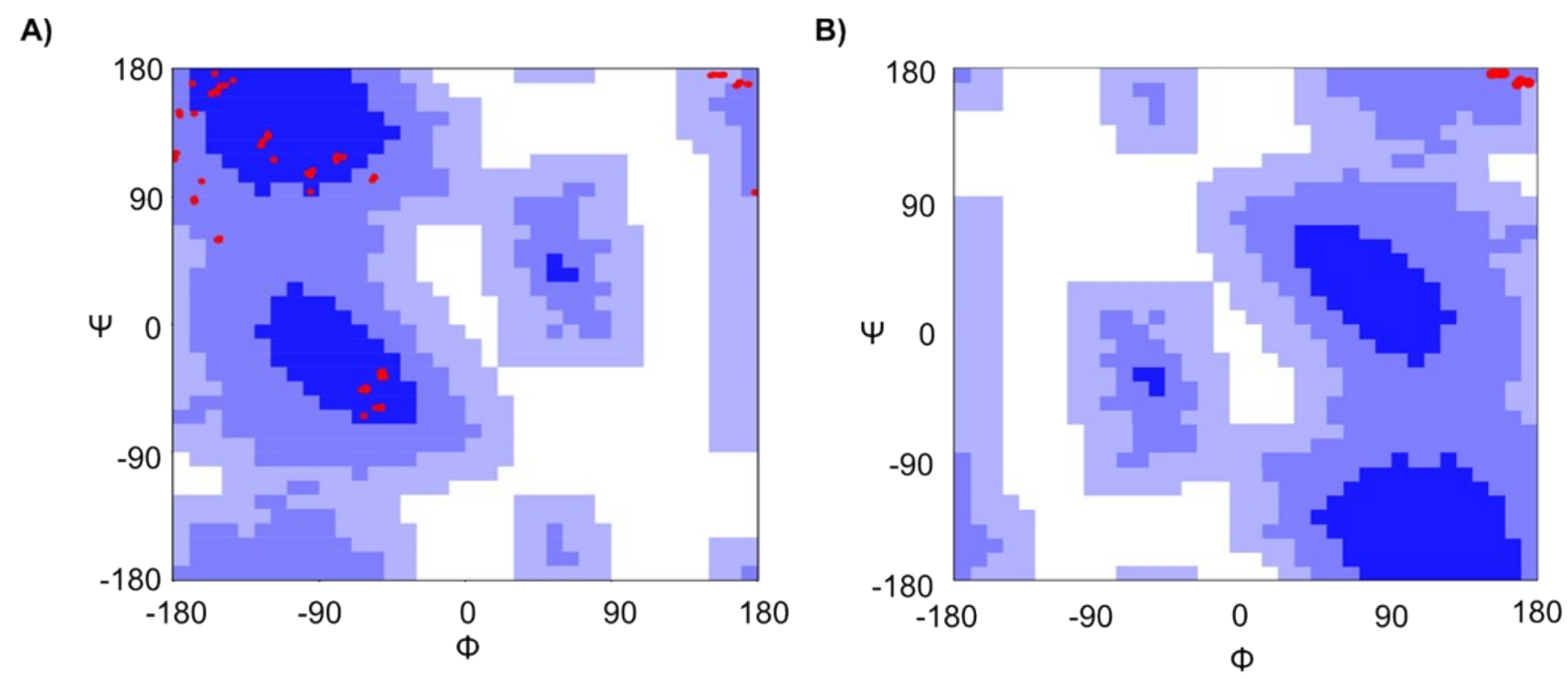

Figure S10. Ramachandran Plot of CorX$_M$ (1c$_A$). A) The blue areas show conformationally allowed regions for L-type amino acids. The red dots represent the dihedrals adopted in the 20 lowest energy conformers from CYANA calculations. B) The blue areas show conformationally allowed regions for D-type amino acids. The red dots represent the dihedrals of D-Thr[9] adopted in the 20 lowest energy conformers from CYANA calculations.

Table S25. Summary of the assessment of 20 calculated conformers of CorX$_M$ (1c$_A$) for allowed and disallowed dihedral angles (generated with CYANA). Favored refers to most favored regions, indicating dihedral angle of residue located in the lowest-energy and the most stable regions of the Ramachandran plot; Additional refers to additionally allowed regions. Generous refers to generously allowed regions; Disallowed refers to disallowed regions. Disallowed AAs refers to those with backbone dihedral angles (Φ and Ψ) falling into the disallowed regions.

Structure	Favored	Additional	Generous	Disallowed	Disallowed residue
1	5	2	1	0	
2	5	2	1	0	
3	5	2	1	0	
4	5	2	1	0	
5	4	4	0	0	
6	4	4	0	0	
7	5	2	1	0	
8	5	2	1	0	
9	5	2	1	0	
10	4	3	1	0	
11	5	2	1	0	
12	5	2	1	0	
13	5	2	1	0	
14	4	4	0	0	
15	5	3	0	0	
16	5	3	0	0	
17	5	3	0	0	
18	5	3	0	0	
19	5	3	0	0	
20	5	3	0	0	
20	5	3	0	0	

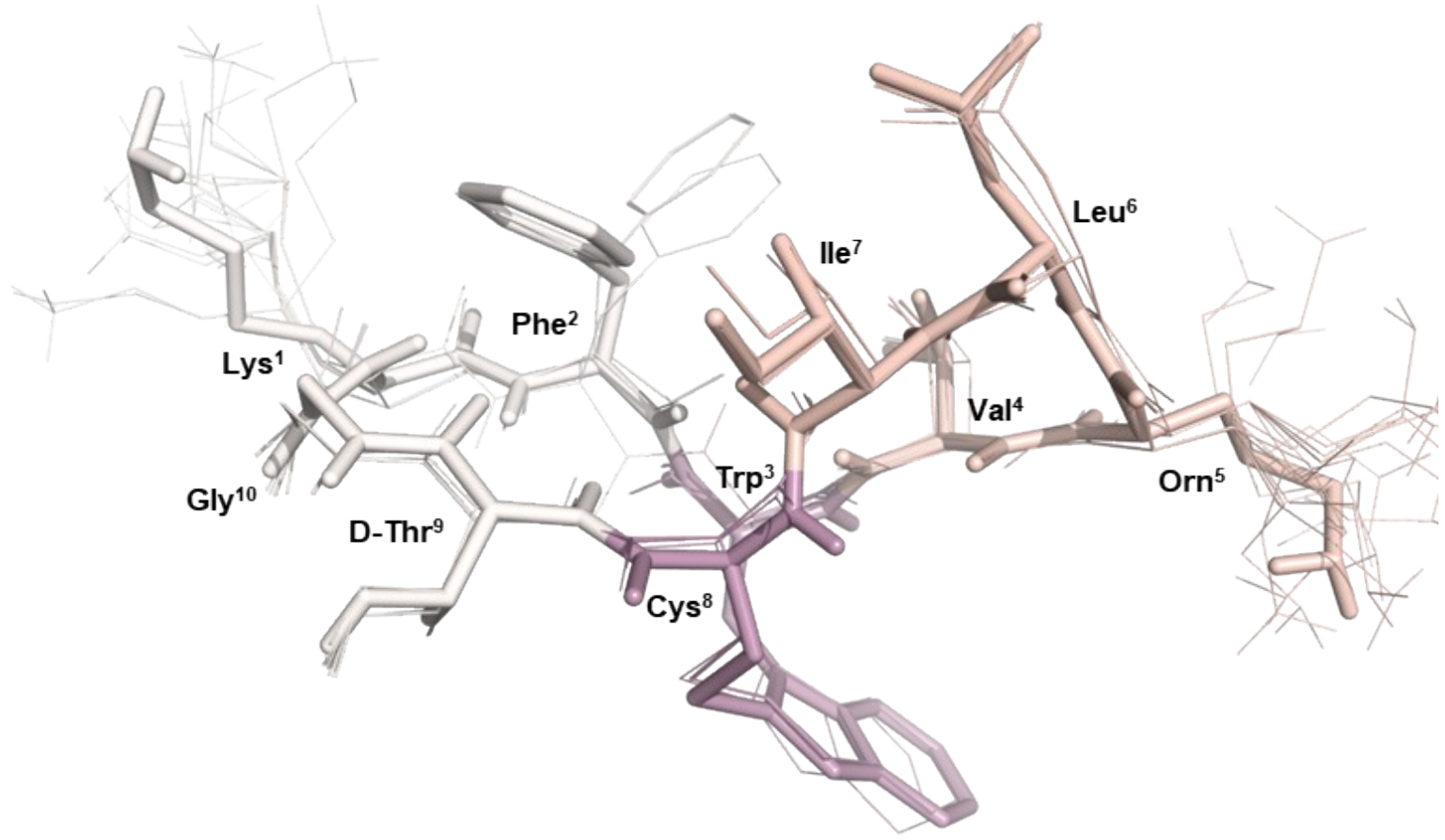

Figure S11. NMR structure ensemble showing the 20 lowest energy conformers of CorX$_M$ (1c$_A$). The conformational ensemble is represented with lines, whereas the lowest-energy conformer is depicted as stick model.

Table S26. Turn motifs in the lowest-energy conformer of CorX$_M$ (1c$_A$). H-bond observed in the lowest-energy conformer is in yellow dash line. H-bond definition: the distance between N and O < 3.5 Å, and the angle of NH⋯O >90 °.[12]

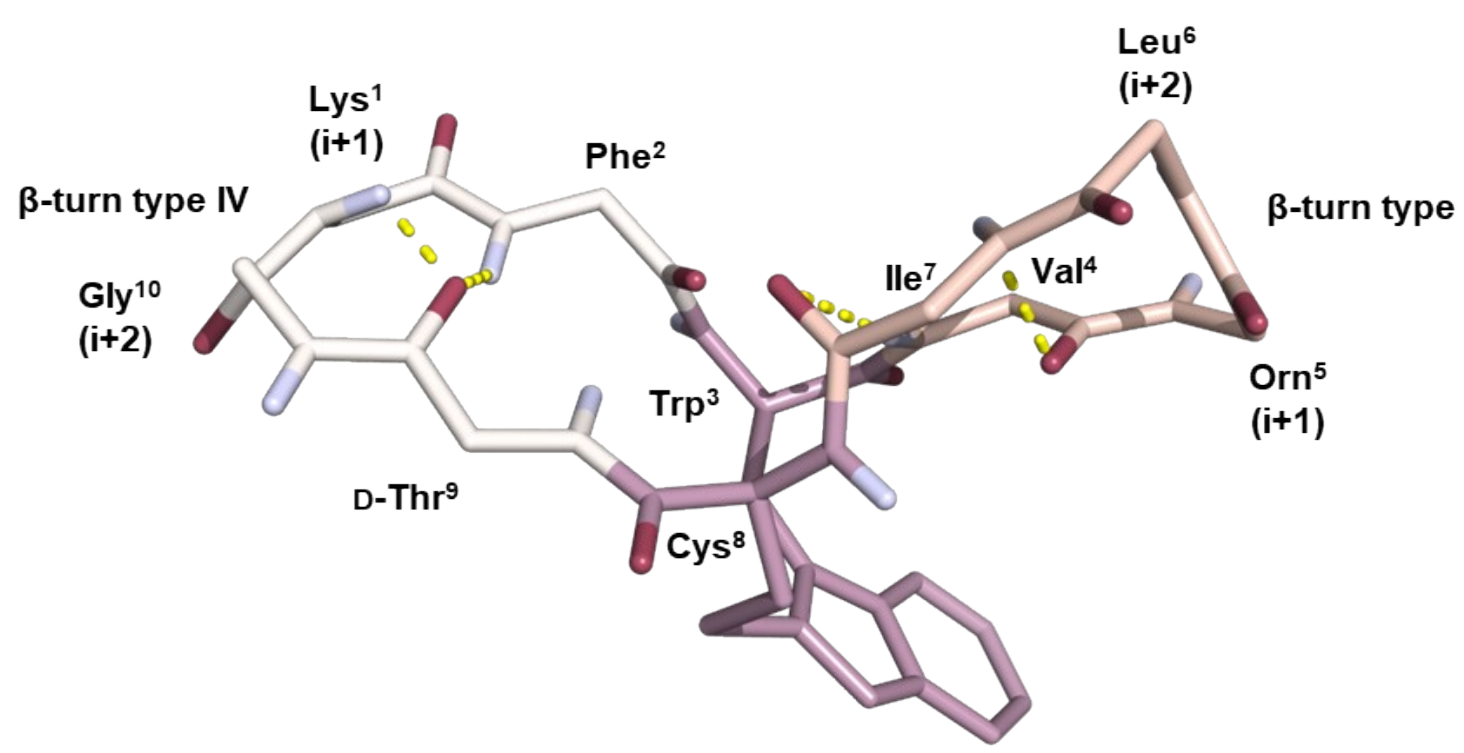

M$_{ansa}$	Residue	Phi	Psi	Type
i+1	Gly	63.9	-98.9	β-turn type IV
i+2	Lys	-152.5	48.1	
i+1	Orn	-52	-35.8	β-turn type I
i+2	Leu	-51.6	-59.2	

7.2.2 CorX$_P$ (1c$_B$, *P*-ansamer of cortinarin X)

Table S27. Overview of CYANA structure calculation.

str	target function	#	upper rms	limits max	#	lower rms	limits max	#	van der sum	Waals max
1	8.75E-03	0	0.0027	0.02	0	0.0000	0.00	0	0.1	0.03
2	8.76E-03	0	0.0029	0.03	0	0.0000	0.00	0	0.1	0.03
3	9.31E-03	0	0.0021	0.02	0	0.0000	0.00	0	0.1	0.04
4	9.33E-03	0	0.0032	0.03	0	0.0000	0.00	0	0.1	0.03
5	1.06E-02	0	0.0037	0.03	0	0.0000	0.00	0	0.1	0.03
6	1.06E-02	0	0.0031	0.03	0	0.0000	0.00	0	0.1	0.04
7	1.06E-02	0	0.0031	0.03	0	0.0000	0.00	0	0.1	0.04
8	1.07E-02	0	0.0032	0.03	0	0.0000	0.00	0	0.1	0.04
9	1.13E-02	0	0.0039	0.04	0	0.0000	0.00	0	0.1	0.03
10	1.17E-02	0	0.0023	0.02	0	0.0000	0.00	0	0.1	0.04
11	1.23E-02	0	0.0035	0.03	0	0.0000	0.00	0	0.1	0.04
12	1.27E-02	0	0.0029	0.03	0	0.0000	0.00	0	0.1	0.04
13	1.30E-02	0	0.0035	0.03	0	0.0000	0.00	0	0.1	0.04
14	1.60E-02	0	0.0049	0.05	0	0.0000	0.00	0	0.2	0.04
15	1.62E-02	0	0.0050	0.05	0	0.0000	0.00	0	0.2	0.04
16	1.62E-02	0	0.0029	0.02	0	0.0000	0.00	0	0.2	0.04
17	2.28E-02	0	0.0041	0.04	0	0.0000	0.00	0	0.2	0.07
18	2.46E-02	0	0.0042	0.03	0	0.0000	0.00	0	0.2	0.07
19	2.61E-02	0	0.0051	0.04	0	0.0000	0.00	0	0.3	0.05
20	2.73E-02	0	0.0045	0.05	0	0.0000	0.00	0	0.3	0.07
Ave	1.44E-02	0	0.0035	0.03	0	0.0000	0.00	0	0.1	0.04
+/-	5.85E-03	0	0.0009	0.01	0	0.0000	0.00	0	0.1	0.01
Min	8.75E-03	0	0.0021	0.02	0	0.0000	0.00	0	0.1	0.03
Max	2.73E-02	0	0.0051	0.05	0	0.0000	0.00	0	0.3	0.07
Cut				0.20			0.20			0.20

RMSDs for residues 1..10:
Average backbone RMSD to mean : 0.29 +/- 0.10 A (0.12..0.45 A; 20 structures)
Average heavy atom RMSD to mean : 0.91 +/- 0.13 A (0.70..1.12 A; 20 structures)

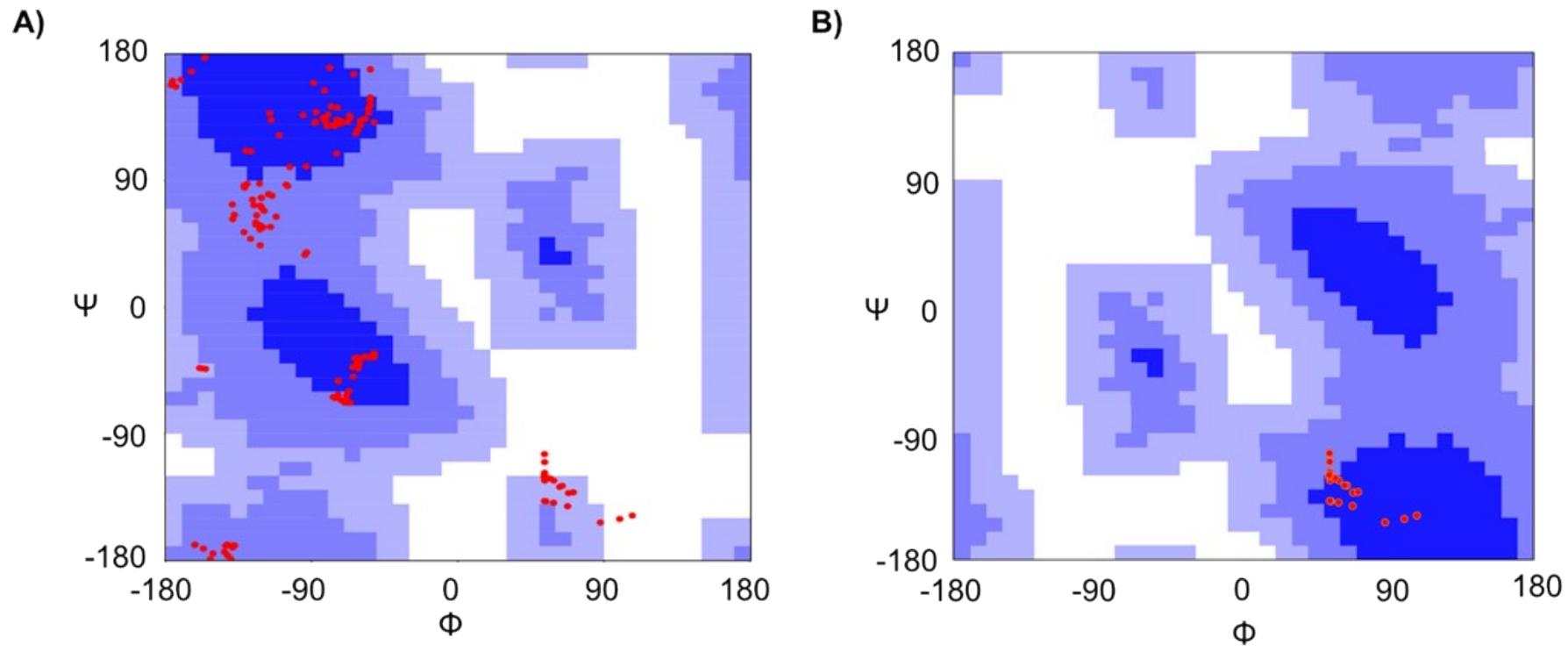

Figure S12. Ramachandran Plot of CorX$_p$ (1c$_B$). A) The blue areas show conformationally allowed regions for L-type amino acids. The red dots represent the dihedrals adopted in the 20 lowest energy conformers from CYANA calculations. B) The blue areas show conformationally allowed regions for D-type amino acids. The red dots represent the dihedrals of D-Thr[9] adopted in the 20 lowest energy conformers from CYANA calculations.

Table S28. Summary of the assessment of 20 calculated conformers of CorX$_P$ (1c$_B$) for allowed and disallowed dihedral angles (generated with CYANA). Favored refers to most favored regions, indicating dihedral angle of residue located in the lowest-energy and the most stable regions of the Ramachandran plot; Additional refers to additionally allowed regions. Generous refers to generously allowed regions; Disallowed refers to disallowed regions. Disallowed AAs refers to those with backbone dihedral angles (Φ and Ψ) falling into the disallowed regions.

Structure	Favored	Additional	Generous	Disallowed	Disallowed residue
1	4	3	1	0	
2	4	3	1	0	
3	4	3	0	1	(D-Thr 9)
4	4	3	1	0	
5	5	2	1	0	
6	4	3	0	1	(D-Thr 9)
7	4	3	0	1	(D-Thr 9)
8	4	3	1	0	
9	4	3	1	0	
10	4	3	0	1	(D-Thr 9)
11	4	3	0	1	(D-Thr 9)
12	4	3	1	0	
13	4	3	0	1	(D-Thr 9)
14	4	3	1	0	
15	4	3	0	1	(D-Thr 9)
16	4	3	1	0	
17	3	3	2	0	
18	1	5	2	0	
19	5	2	1	0	
20	3	3	2	0	
20	3	3	2	0	

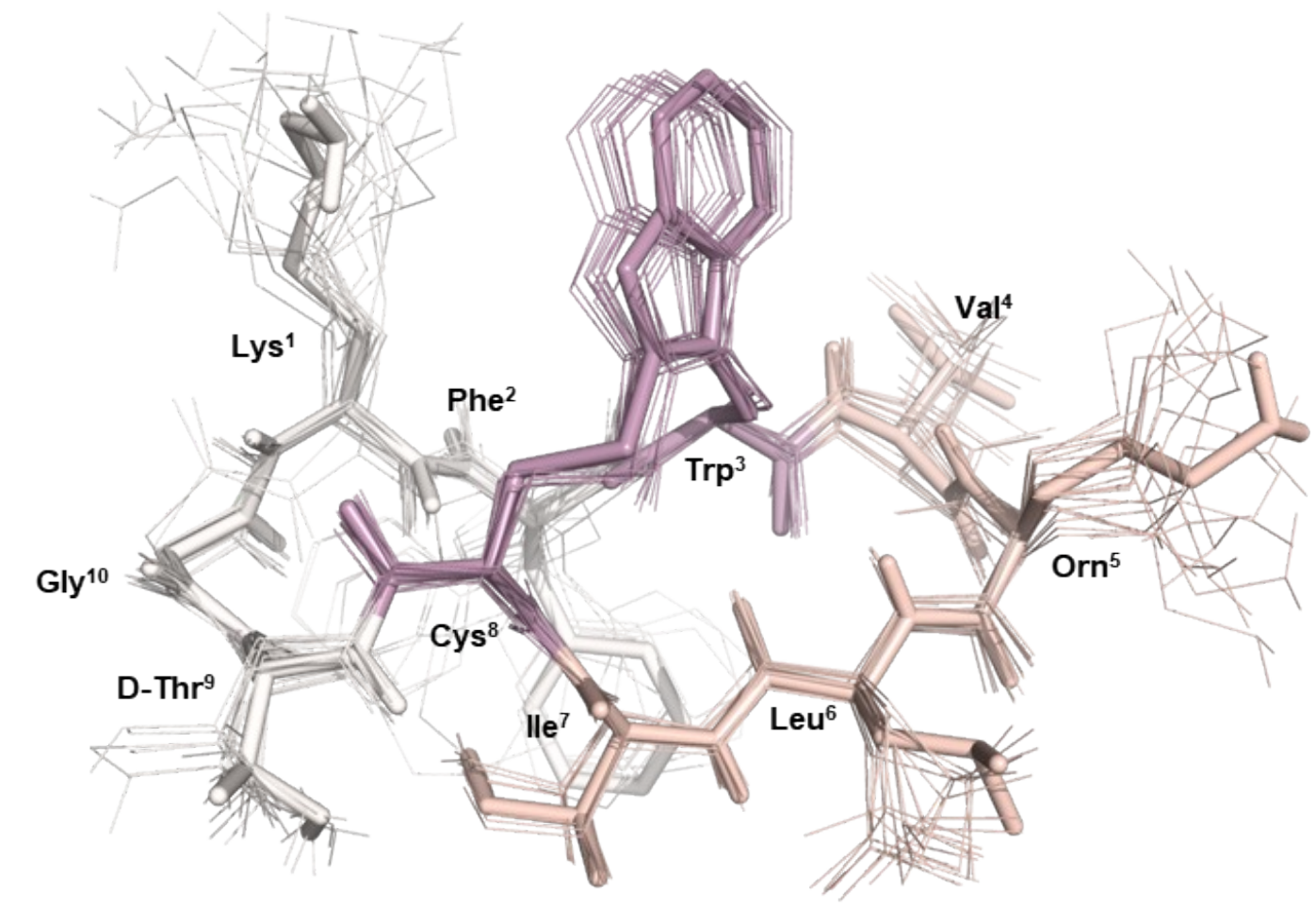

Figure S13. NMR structure ensemble showing the 20 lowest energy conformers of CorX$_P$ (1c$_B$). The conformational ensemble is represented with lines, whereas the lowest-energy conformer is depicted as stick model.

Table S29. Turn motifs in the lowest-energy conformer of CorX$_P$ (1c$_B$). H-bond observed in the lowest-energy conformer is in yellow dash line. H-bond definition: the distance between N and O < 3.5 Å, and the angle of NH⋯O >90 °.[12]

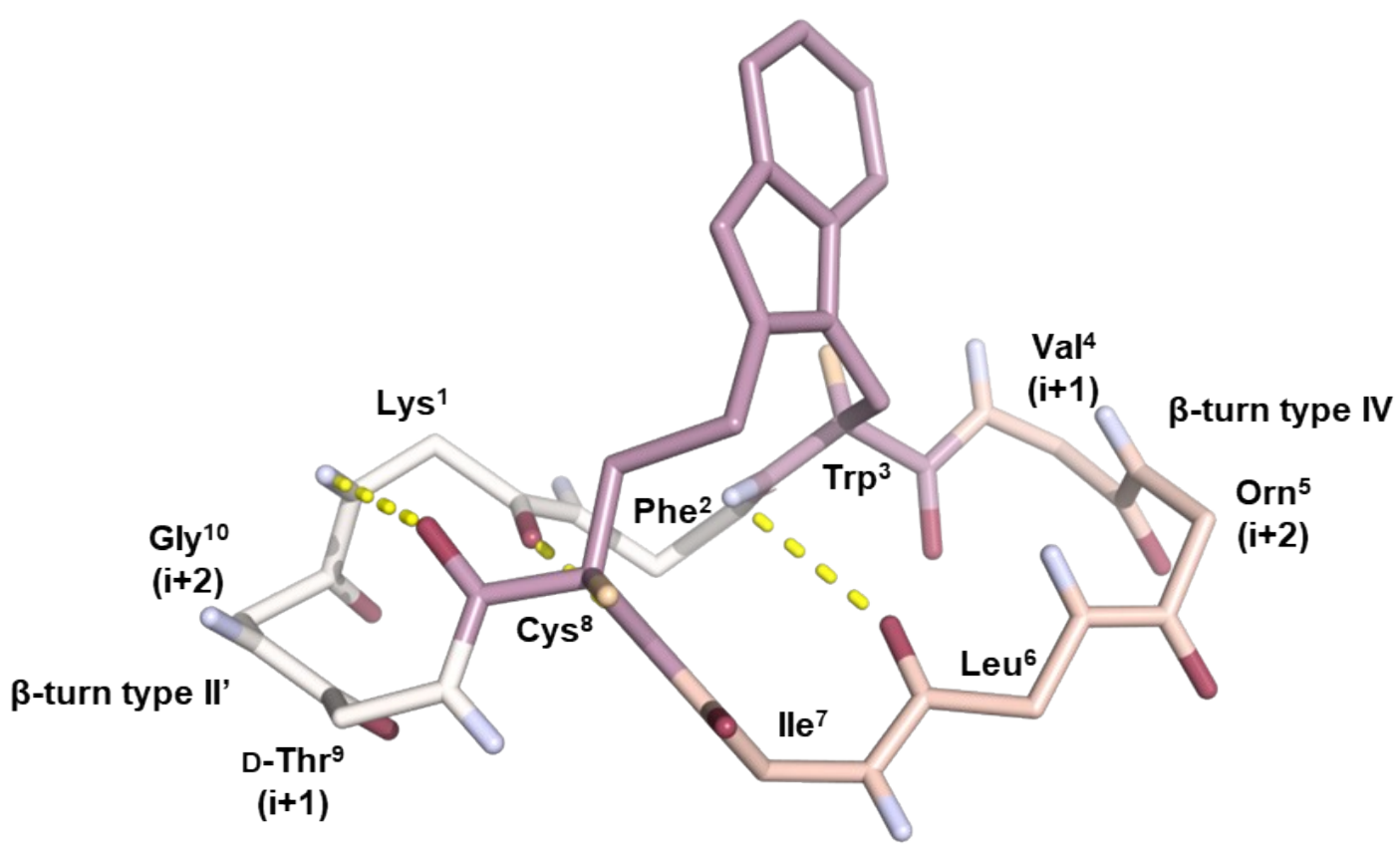

P_{ansa}	Reisdue	Phi	Psi	Type
i+1	D-Thr	64.1	-127.2	β-turn type II'
i+2	Gly	-56.4	-62.5	
i+1	Val	-62	-35.9	β-turn type IV
i+2	Orn	-68.6	-62.6	

8 Variable-temperature (VT) NMR spectroscopy

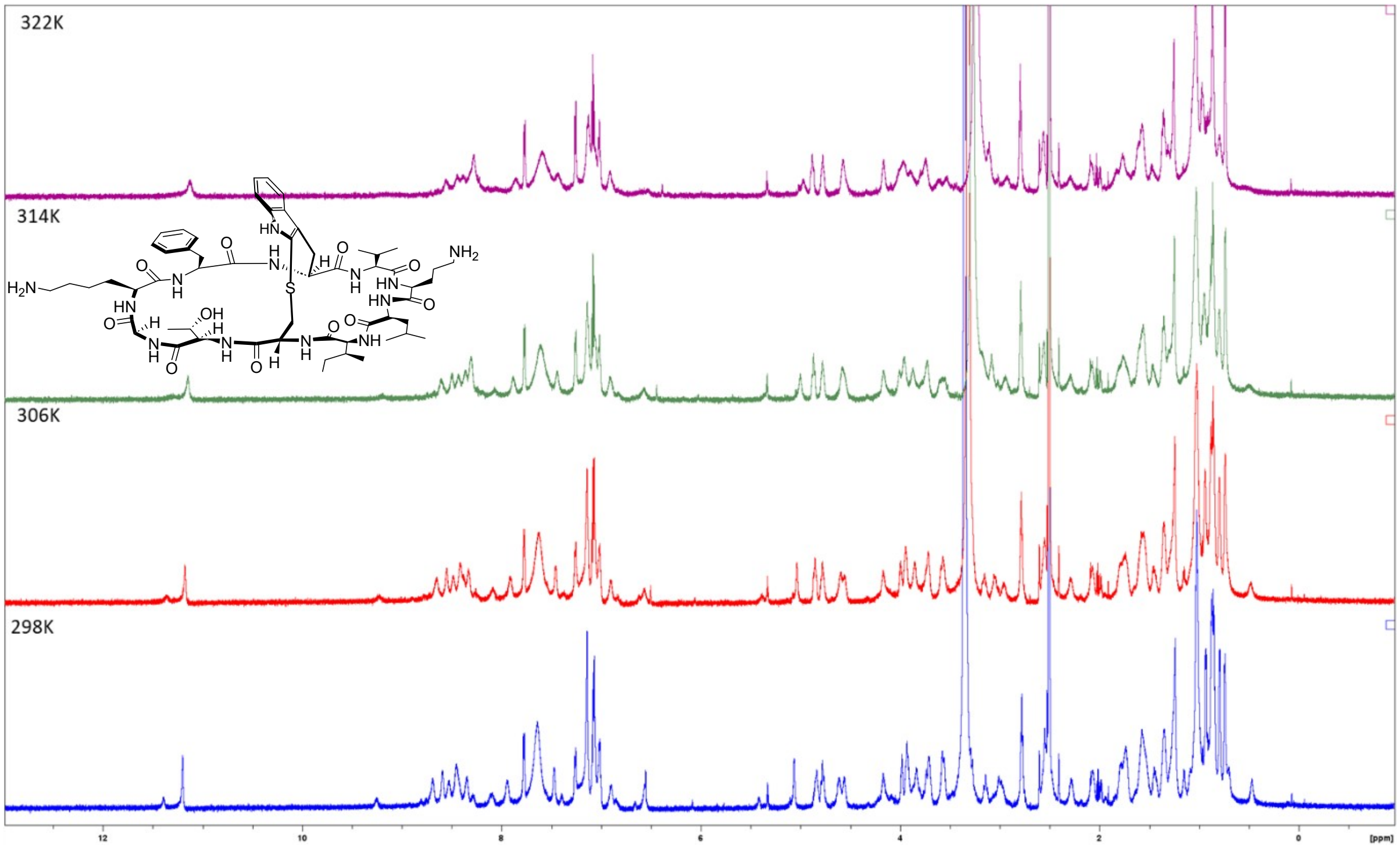

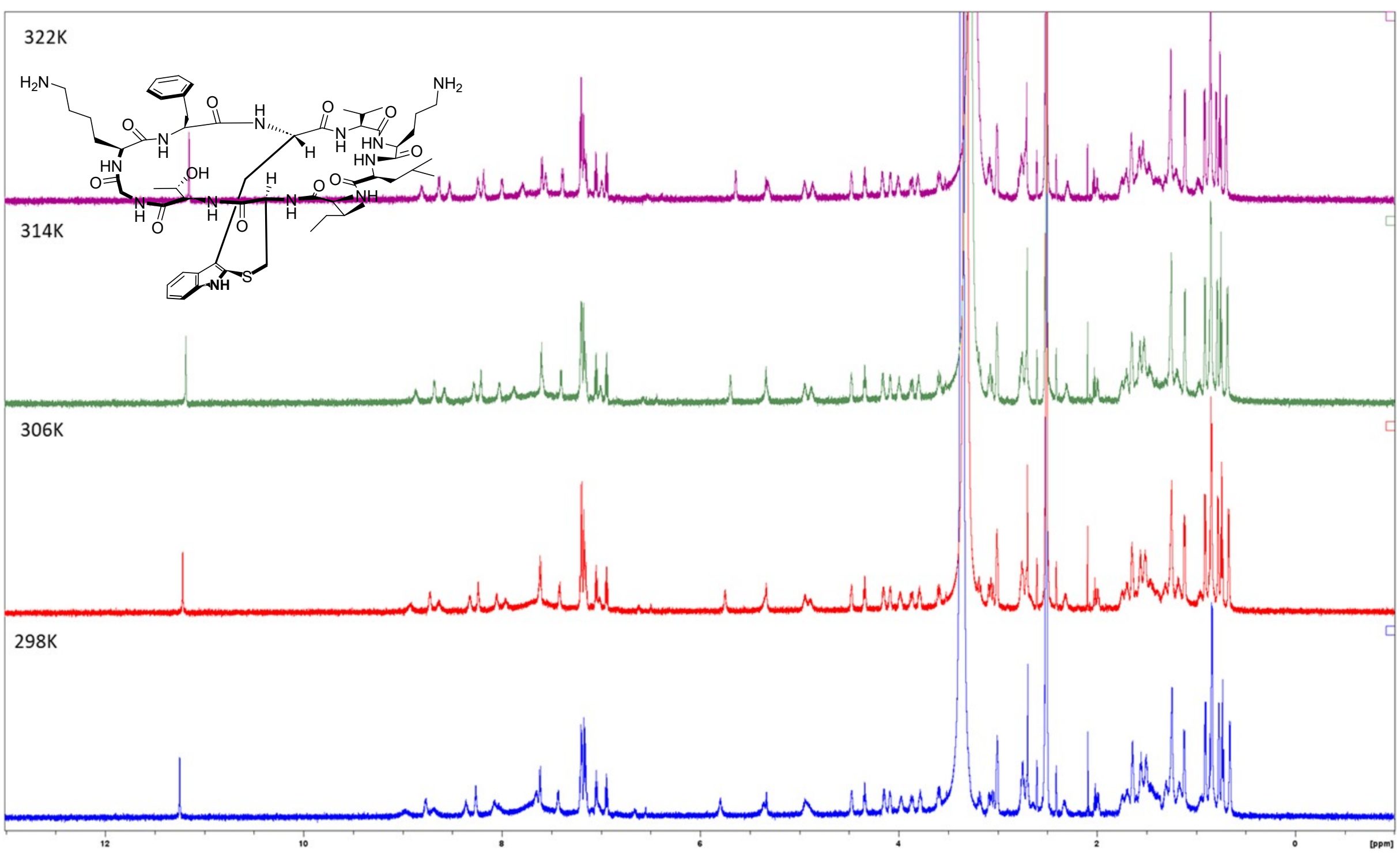

Figure S14. Variable-temperature (VT) ^{1}H-NMR spectroscopy study of CorX$_P$ (*top*) and CorX$_M$ (*bottom*) (temperature range 298 K - 333 K as indicated).

Table S30. Temperature-dependent chemical shift changes $\Delta\delta_{HN}/\Delta T$ (ppb/K) for amide and hydroxyl protons of CorX$_P$ as observed by VT-NMR (temperature range 298 K - 322 K). Amide protons exhibiting small temperature coefficients ($-\Delta\delta_{HN}/\Delta T$ < 3.0 ppb K^{-1}) are indicative of H-bonds (in green), whereas solvent-exposed amides show large temperature coefficients ($-\Delta\delta_{HN}/\Delta T$ > 4.6 ppb K^{-1}, in red). Intermediate values are shown in yellow. In the curve fitting, positive slope ($\Delta\delta_{HN}/\Delta T$ >0) indicates that the chemical shift of amide and hydroxyl protons shift downfield, while negative slope ($\Delta\delta_{HN}/\Delta T$ <0) indicates upfield shifting.

CorX$_P$	298 K	306 K	314 K	322 K	$\Delta\delta_{HN}/\Delta T$ (ppb/K)	Curve fitting
Lys[1]	6.897	6.907	6.910	6.910	0.525	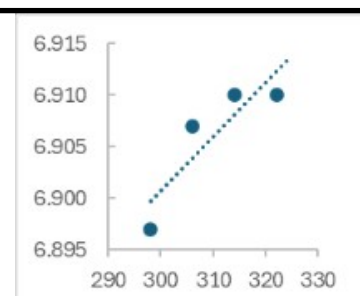
Phe[2]	8.458	8.410	8.360	8.300	-6.550	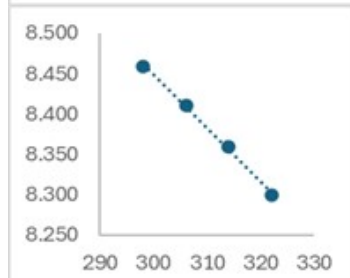
Trp[3]	7.940	7.911	7.882	7.855	-3.550	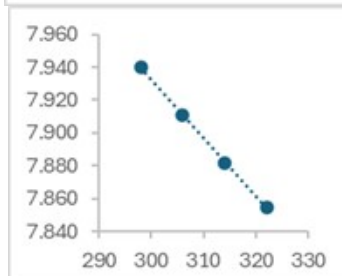
Val[4]	8.596	8.550	8.500	8.440	-6.475	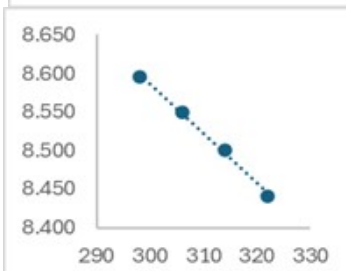
Orn[5]	8.528	8.485	8.430	8.380	-6.238	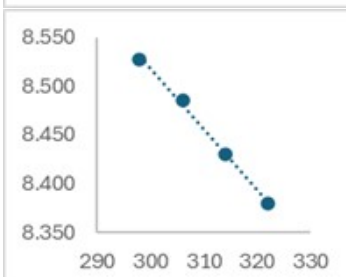
Leu[6]	7.185	7.170	7.160	7.150	-1.438	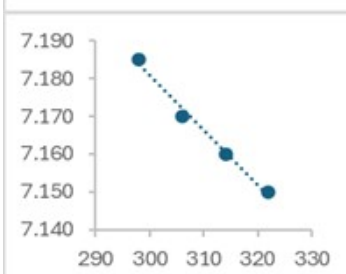
Ile[7]	8.690	8.650	8.600	8.550	-5.875	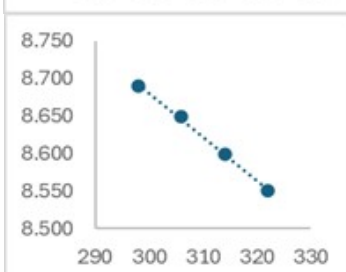
Cys[8]	8.350	8.330	8.300	8.280	-3.000	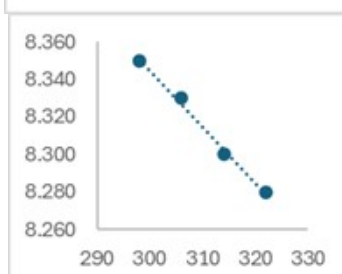
D-Thr9	7.470	7.454	7.440	7.426	-1.825	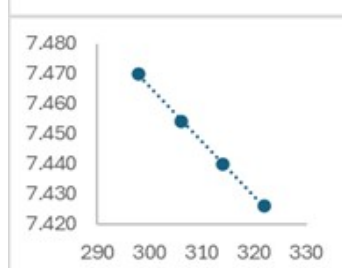
Gly[10]	8.430	8.370	8.320	8.280	-6.250	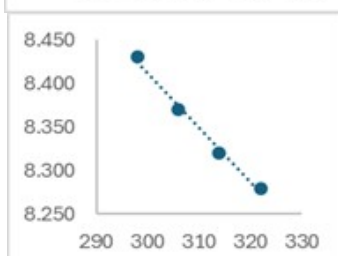

Table S31. Temperature-dependent chemical shift changes $\Delta\delta_{HN}/\Delta T$ (ppb/K) for amide and hydroxyl protons of CorX$_M$ as observed by VT-NMR (temperature range 298 K - 322 K). Amide protons exhibiting small temperature coefficients ($-\Delta\delta_{HN}/\Delta T$ < 3.0 ppb K^{-1}) are indicative of H-bonds (in green), whereas solvent-exposed amides show large temperature coefficients ($-\Delta\delta_{HN}/\Delta T$ > 4.6 ppb K^{-1}, in red). Intermediate values are shown in yellow. In the curve fitting, positive slope ($\Delta\delta_{HN}/\Delta T$ >0) indicates that the chemical shift of amide and hydroxyl protons shift downfield, while negative slope ($\Delta\delta_{HN}/\Delta T$ <0) indicates upfield shifting.

CorX$_M$	298 K	306 K	314 K	322 K	ΔδHN/ΔT (ppb/K)	Curve fitting
Lys[1]	8.358	8.319	8.279	8.237	-5.038	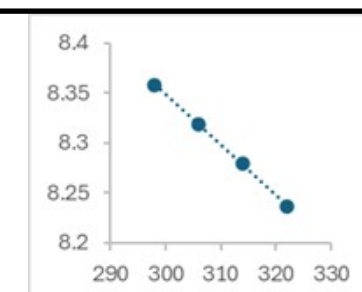
Phe[2]	7.429	7.414	7.400	7.386	-1.788	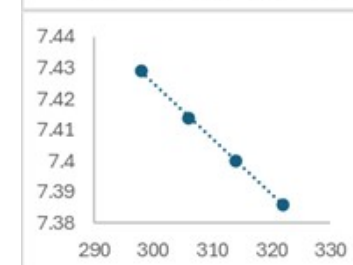
Trp[3]	8.967	8.919	8.862	8.803	-6.863	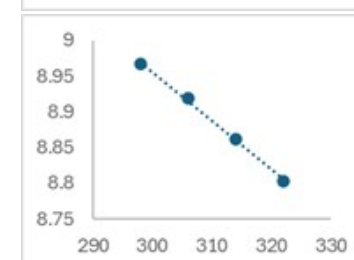
Val[4]	8.071	8.047	8.021	7.998	-3.062	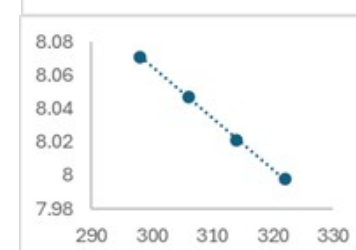
Orn[5]	8.016	7.956	7.871	7.786	-9.688	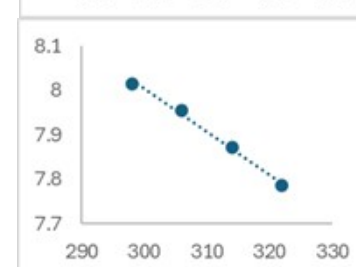
Leu[6]	7.651	7.624	7.587	7.556	-4.025	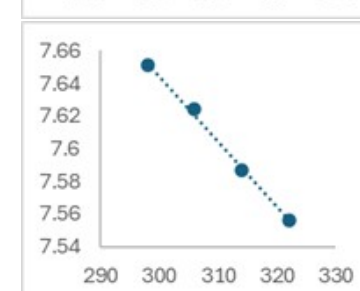
Ile[7]	7.020	7.013	7.002	6.991	-1.225	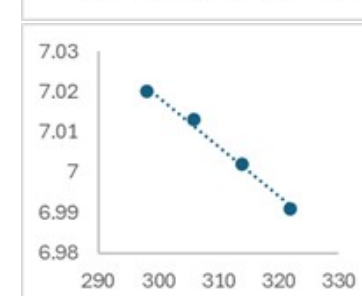
Cys[8]	8.761	8.717	8.677	8.63	-5.413	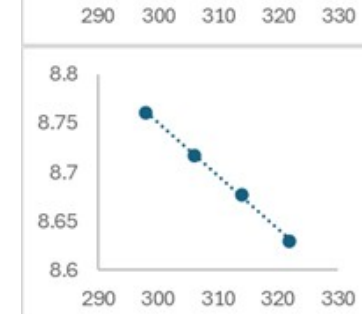
D-Thr[9]	8.675	8.628	8.574	8.524	-6.338	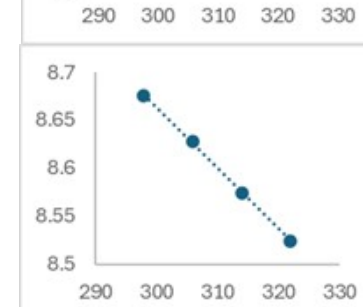
Gly[10]	8.261	8.237	8.206	8.18	-3.425	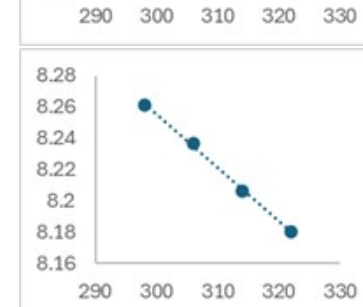

9 Molecular dynamics (MD) simulations

9.1 Computational method

9.1.1 Solvent box

To generate a solvent box of DMSO, a single molecule of DMSO was prepared in Avogadro[13] and parametrized with ACPYPE[14] based on the General Amber Force Field 2 (GAFF2)[15]. Atomic partial charges were assigned using the AM1-BCC method as implemented in Antechamber (AmberTools)[16]. The molecule was then placed into a cubic box with 0.48 nm side length. The box with the molecule was then stacked in all three dimensions to yield a box of 9.6 nm side length, filled with 800 DMSO molecules in a regular grid. This system was energy minimized and equilibrated in the NVT ensemble at 300 K for 10 ns using velocity rescaling with a stochastic term[17]. The system was then equilibrated in the NPT ensemble at 300 K and 1.0 bar using stochastic cell rescaling[18] as a barostat for 100 ns. The resulting solvent box was used to solvate the starting structures.

9.1.2 Simulation system setup

The starting structures, CorX$_P$ and CorX$_M$ were used from CYANA[11] calculations (lowest-energy conformer each, see **Figure S11 and Figure S13**). The molecules were parameterized using ACPYPE based on GAFF2 and atomic partial charges from the AM1-BCC. The following preparation steps were using Gromacs 2021[19–21]. The starting structures were placed into a cubic box with periodic boundary conditions and 1.1 nm between the solute and the box edges. The system was then solvated in DMSO and two chloride ions were added to yield a neutral simulation box. The system was then energy minimized and equilibrated in the NVT ensemble at 300 K for 100 ps, followed by an equilibration in the NPT ensemble at 300 K and 1.0 bar for 10 ns. The same thermostat[17] and barostat[18] as in the production simulations were used.

9.1.3 Molecular dynamics simulation

Molecular dynamics simulations were conducted using Gromacs (2021.1)[19–21]. The systems were propagated using the leap-frog integrator with a time step of 2 fs. Bonds involving hydrogen atoms were constrained using the LINCS algorithm. Periodic boundary conditions were applied in all three spatial directions. Simulations were performed in a cubic box (4.59 nm edge length) containing 802 DMSO molecules, two chloride ions and one cortinarin in the NPT ensemble at 300 K using the velocity-rescale thermostat (with coupling time 1.0 ps) and at 1 bar using the Parrinello–Rahman barostat (with coupling time 2.0 ps). Non-bonded interactions were treated with the Verlet cutoff scheme using a cutoff radius of 1.1 nm and a neighbor list update frequency of 20 steps. Long-range electrostatics were calculated using the Particle Mesh Ewald (PME) method (fourier spacing = 0.12 nm, PME order = 4) with a real-space cutoff of 1.1 nm. Coordinates were recorded every 10 ps during 1 μs of three production runs.

9.1.4 Inter-residual hydrogen distances

To compare MD data with experimental inter-residual NOE distance, hydrogen distances are given as an ensemble average,

$$R_{H_a,H_b}^{MD} = \frac{1}{N_{rep}} \sum^{N_{rep}} \langle r(H_a,H_b)^{-6} \rangle^{-1/6} \tag{1}$$

overall replica N_{rep}. The distances are weighted with respect to the magnetic dipole–dipole interaction between two spins, with $r(H_a,H_b)^{-6}$ dependency. The intensity term is related back to the NOE distance by the power of $\langle r(H_a,H_b)^{-6} \rangle^{-1/6}$.

A deviation between experimental R_{H_a,H_b}^{NOE} and simulation data R_{H_a,H_b}^{MD} is obtained by the difference,

$$\Delta R_{H_a,H_b} = R_{H_a,H_b}^{MD} - R_{H_a,H_b}^{NOE}. \tag{2}$$

A deviation of more than 0.1-0.2 nm violates the NOE threshold, i.e. MD and NOESY data are significantly different.

9.1.5 Solvent-accessible surface area

The Solvent Accessible Surface Area (SASA) can be used to understand the structure function relationship of proteins and their residues. The method measures the surface area in units nm^2 of the protein that is accessible to solvent molecules. The accessible surface area of the molecule is calculated based on the contact points of a sphere with a series of points on the molecule surface. An example is the numerical method called the Shrake-Rupley algorithm[22], which draws 92 points equidistant from each atom of the molecule. SASA values can be computed separately for hydrophilic and hydrophobic residues or with respect to the amide groups.

9.1.6 Hydrogen bonds

Hydrogen bonds were analyzed with Gromacs (2021.1)[19–21] simulation package. The hydrogen bonds were evaluated based on their relative occurrence over the total simulation length. Hydrogen bonds with a relative occurrence greater than 10% were considered significant. Geometrically, a hydrogen bond was defined by a donor-acceptor distance ≤ 0.35 nm and a donor-hydrogen-acceptor angle ≤ 30°.

9.2 Results of MD simulation

Cortinarin X in dimethyl sulfoxide (DMSO) is studied. Unbiased simulations of 1 μs were carried out for each cortinarin starting from both ansamers. The initial structure for each system was derived from CYANA structures. Details about the computational methods are given in **Section 9.1.**

9.2.1 Conformational analysis

The probability density distribution of the *P* and *M*-ansamer identifier $\theta_{residue,\ anchor}$ is investigated in a dihedral analysis. The β-carbon atom (CB) serves as an anchor for both reference planes marked with dashed lines (**Figure S15**).

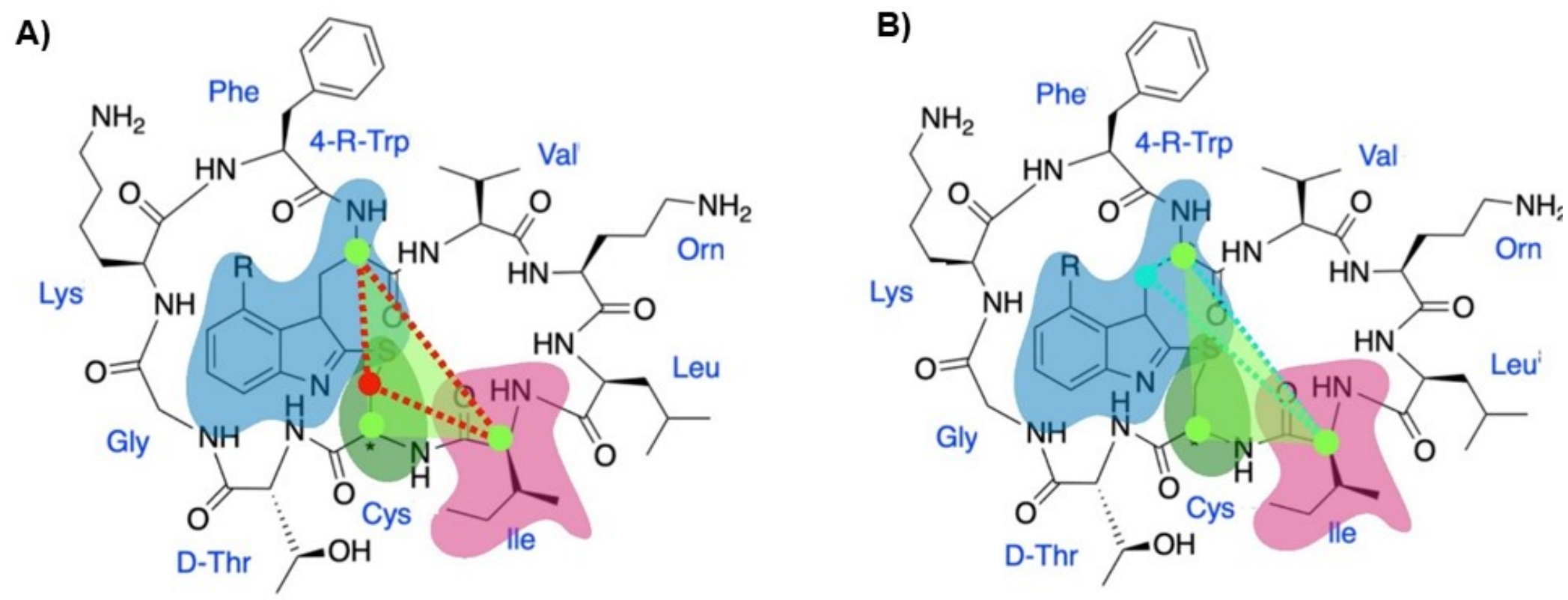

Figure S15. Ansamer dihedrals definition. Ansamer dihedrals with respect to the a) β-carbon atom (Cβ, red) cysteine $\theta_{C, CB}$ and b) to the β-carbon atom (Cβ, cyan) tryptophan $\theta_{W, CB}$.

The results of CorX are from MD simulations with initial structure *P*-ansamer (orange) and *M*-ansamer (hatched). We compare the $CorX_P$ and $CorX_M$ dihedral distributions with that of CorA, CorB and CorC, where simulations with *P*-ansamer as starting structures were considered. The distribution of the two CorX ansamer identifiers only extends to the dihedrals that correspond to the initial configuration. In other words, no probability density spans from the region marked in **Figure S15** in magenta (A) 45-135 ° or B) 270-0 °) to the region marked in turquoise (A) 225-315 ° or B) 0-90 °). Thus, no flip of tryptathionine is observed on the time scalar of the MD simulation.

Interestingly, the identifier distribution matches the most observed identifier values for cortinarin B with $\theta_{A, CB} = 80\,^{\circ}$ and $\theta_{W, CB} = 335\,^{\circ}$. This suggests that the orientation of the thioether bridge relative to the macrocycle is influenced by the type and size of the rest R at the tryptophan.

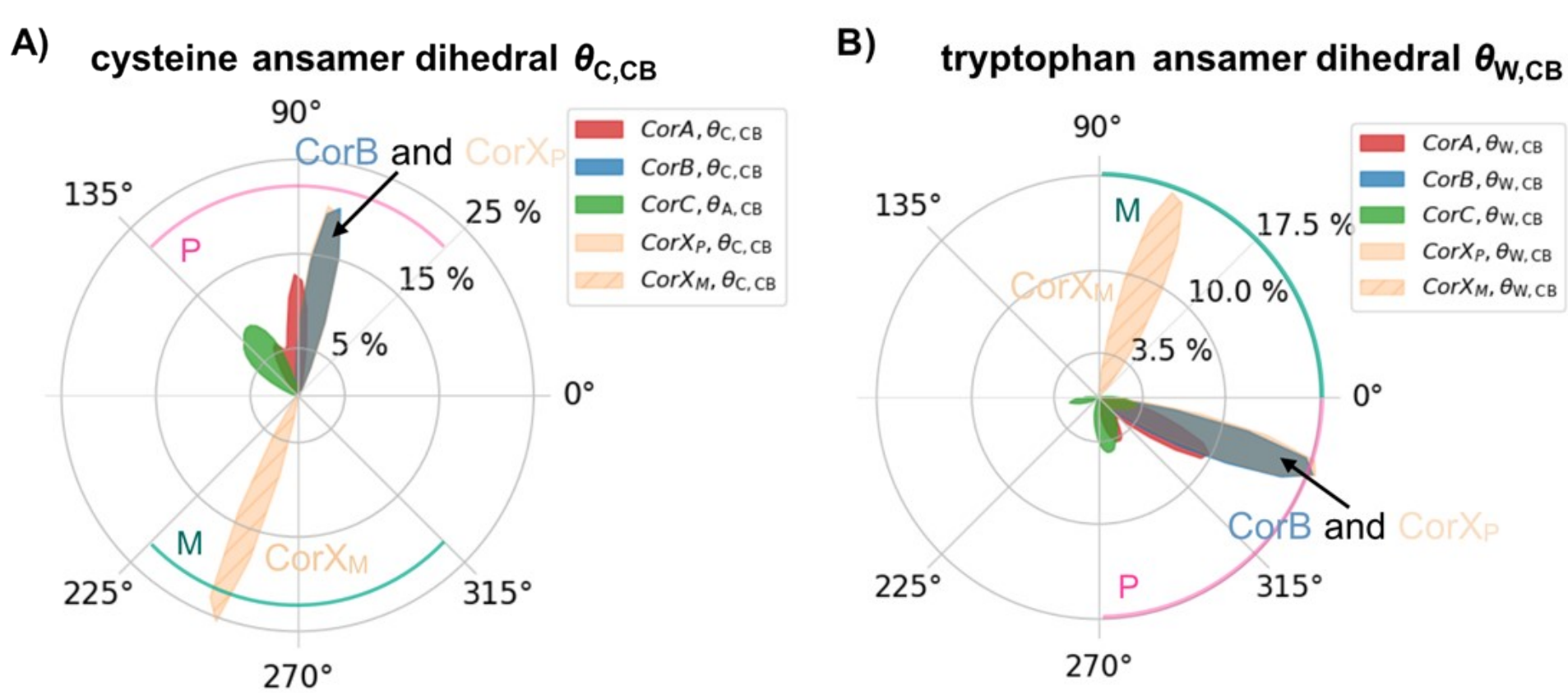

Figure S16. Illustration of ansamer dihedrals probability density. A) In cysteine ansamer dihedral $\theta_{C, CB}$, the allowed dihedral angels for *P*-ansamers is 45-135 ° (magenta), while that for *M*-ansamer is 225-315 ° (turquoise). B) For tryptophan ansamer dihedral $\theta_{W, CB}$, the allowed range of *P*-ansamer is 0-135 ° (magenta), and 225-315 ° is for *M*-ansamer (turquoise). 1 µs MD simulation was performed using *P*-ansamer of CorA (red), CorB (blue), CorC (green), $CorX_P$ (orange), and *M*-ansamer $CorX_M$ (hatched). In A, B), the conformation of CorA, CorB, $CorX_P$ retained within P-ansamer allowed region, while $CorX_M$ retained

in M-ansamer allowed region. The dominant conformation of CorC remained in *P*-ansamer, but a minor population deviated from the dihedral angles range defined for *P*/*M*-ansamer.

9.2.2 Inter-residual hydrogen distance analysis

The deviation $\Delta R_{H_a,H_b}$ (**eq. (2) in Sec. 9.1.5**) between experimental NOESY distances $R^{NOE}_{H_a,H_b}$ (**Table S22 and Table S23**) and simulation ensemble averages $R^{MD}_{H_a,H_b}$ are presented (**Figure S17 and Figure S18**) for all distances below 0.5 nm. The NOE distance threshold, where deviation of more than 0.2 nm is observed and is marked as red line. The characteristic ansamer NOESY signal between the Hα of Trp[3] and Cys[8] appearing just for CorX$_M$ is marked grey (**Figure S18**). The average distance for the Hα of Trp[3] and Cys[8] in CorX$_M$ is 0.22 nm and in CorX$_P$ 0.75 nm.

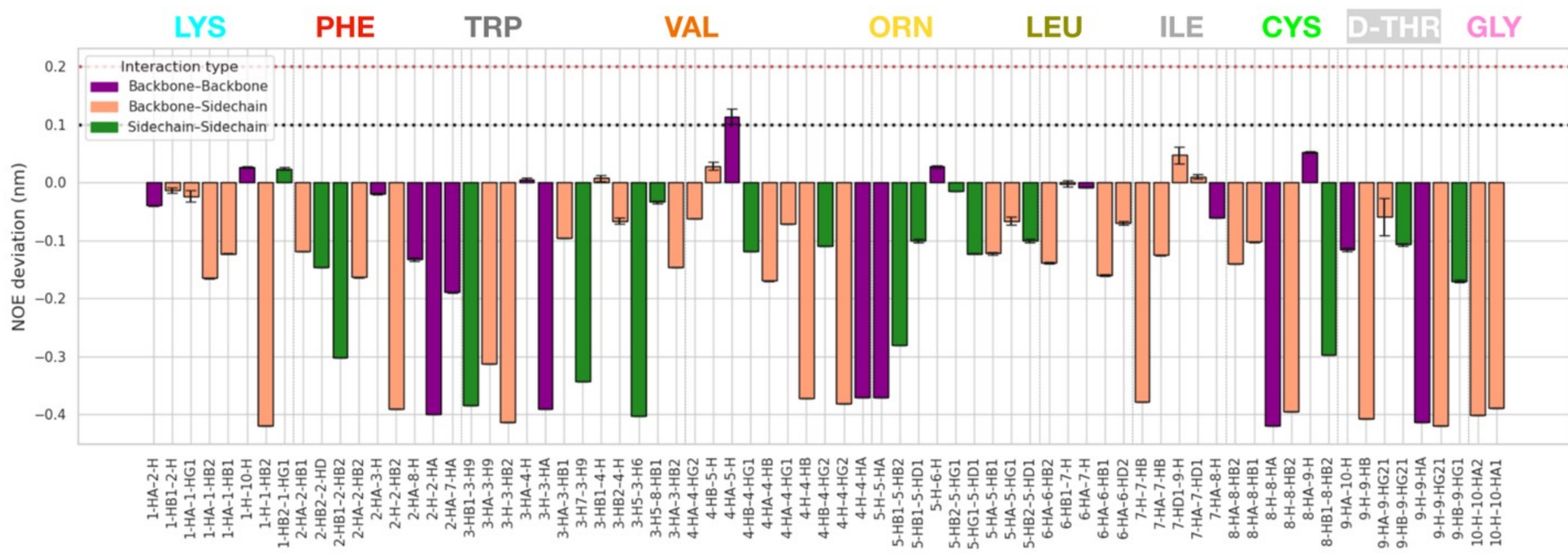

Figure S17. Comparison of NOE and MD data for CorX$_P$. The deviation $\Delta R_{H_a,H_b}$, with highlighted interaction types (purple Backbone-Backbone; orange Backbone-Sidechain; green Sidechain-Sidechain) and sorted by residue.

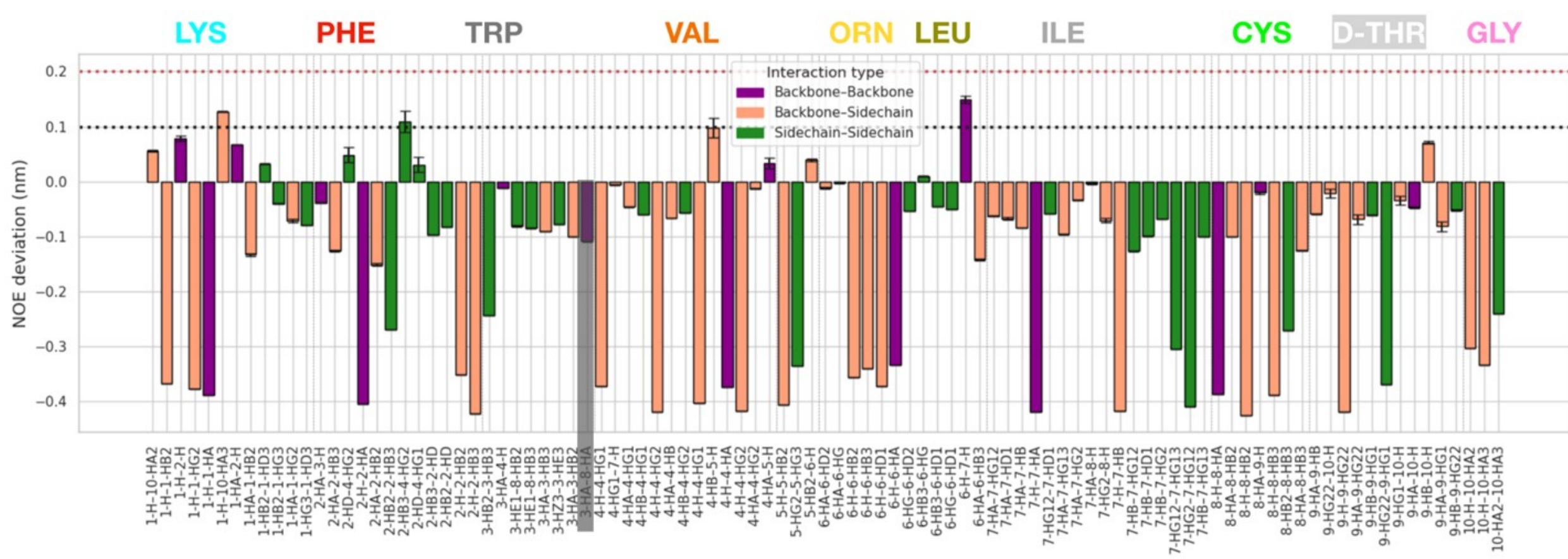

Figure S18. Comparison of NOE and MD data for CorX$_M$. The deviation $\Delta R_{H_a,H_b}$, with highlighted interaction types (purple Backbone-Backbone; orange Backbone-Sidechain; green Sidechain-Sidechain; grey CorX$_M$ specific Hα of Trp[3] and Cys[8]) and sorted by residue.

9.2.3 Solvent accessible surface area (SASA) analysis

Solvent Accessible Surface Area (SASA) results from the MD simulations. Amide groups with a small SASA of less than 0.05 nm^2 are considered buried, while those with a large SASA (greater than 0.15 nm^2) are exposed. Amides with intermediate SASA values exhibit partial accessibility.

Table S32. SASA analysis for $CorX_P$. Mean value and standard deviation of SASA analysis in nm^2 per residue.

Residue	SASA mean nm^2	SASA standard deviation nm^2
Lys^1	0.0082	0.0008
Phe^2	0.0551	0.0009
Trp^3	0.0001	0.0000
Val^4	0.0399	0.0045
Orn^5	0.0111	0.0021
Leu^6	0.0161	0.0006
Ile^7	0.0356	0.0006
Cys^8	0.0000	0.0000
D-Thr^9	0.0009	0.0001
Gly^{10}	0.1020	0.0157

Table S33. SASA analysis for $CorX_M$. Mean value and standard deviation of SASA analysis in nm^2 per residue.

Residue	SASA mean nm^2	SASA standard deviation nm^2
Lys^1	0.0110	0.0048
Phe^2	0.0149	0.0002
Trp^3	0.0677	0.0002
Val^4	0.0002	0.0001
Orn^5	0.0110	0.0039
Leu^6	0.0034	0.0008
Ile^7	0.0032	0.0005
Cys^8	0.0532	0.0018
D-Thr^9	0.0012	0.0004

Gly^{10}	0.0485	0.0152

10 Immunofluorescence microscopy and Western blotting

Immunofluorescence microscopy was carried out as described.[23–26] In brief, cells were grown on 12 mm coverslips. Detection of AQP2 was carried out using custom-made antibody H27 (1:300) directed against the C terminus of AQP2 and secondary antibody Cy3-F(ab')2-anti-rabbit IgG (1:300, BIOZOL Diagnostica, #JIM-711-166-152). For plasma membrane detection, antibodies against the tight junction protein zonula occludens-1 (ZO-1; 1:200, Santa Cruz Biotechnology, #sc-33725) and secondary antibody AlexaFluor plus 647-anti-rat IgG (1:150, Invitrogen, #A48265) were used.[23,27,28] Fluorescence signals were detected using a confocal laser scanning microscope (LSM) 710 (Carl Zeiss, Jena, Germany) with a Plan-Apochromat 40 x / 1,30 oil immersion objective. Image analysis was performed using the software Fiji by Image J (Version 2.16.0 / 1.54p). AQP2 localization was quantified using a single cell quantification approach as described.[29]

Western blotting was carried out as described[23,27], using the following primary and secondary antibodies: AQP2 (1:1000, AQP2 (E-2) HRP, #sc-515770 HRP, Santa Cruz Biotechnology), pSer261-AQP2 (1:2000, Novusbio; #NB100-61100), HSP90 (1:2000, Enzo, Stressgen, #ADI-SPA-830-F), peroxidase anti-rabbit IgG light chain specific (1:1000, Jackson Immuno Research, #211-032-171), POD-anti-mouse IgG (H+L, 1:5000, Jackson Immuno Research, #715-035-151).

11 NMR Spectra

NMR (^{1}H 500 MHz, $CDCl_3$) of *tert*-butyl 3-formyl-4-OMe-1*H*-indole-1-carboxylate (S2a)

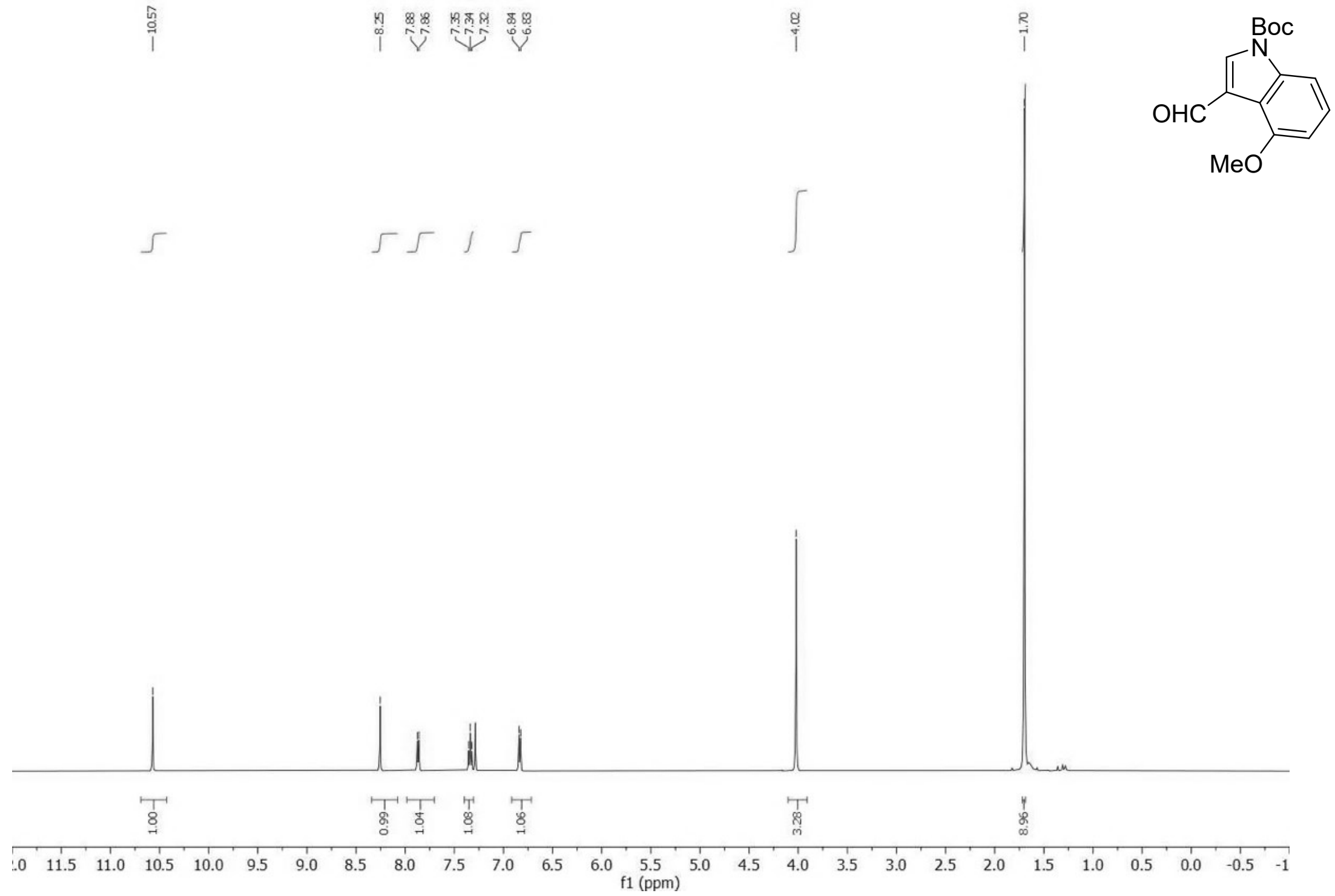

NMR (^{13}C 125 MHz, $CDCl_3$) of *tert*-butyl 3-formyl-4-OMe-1*H*-indole-1-carboxylate (S2a)

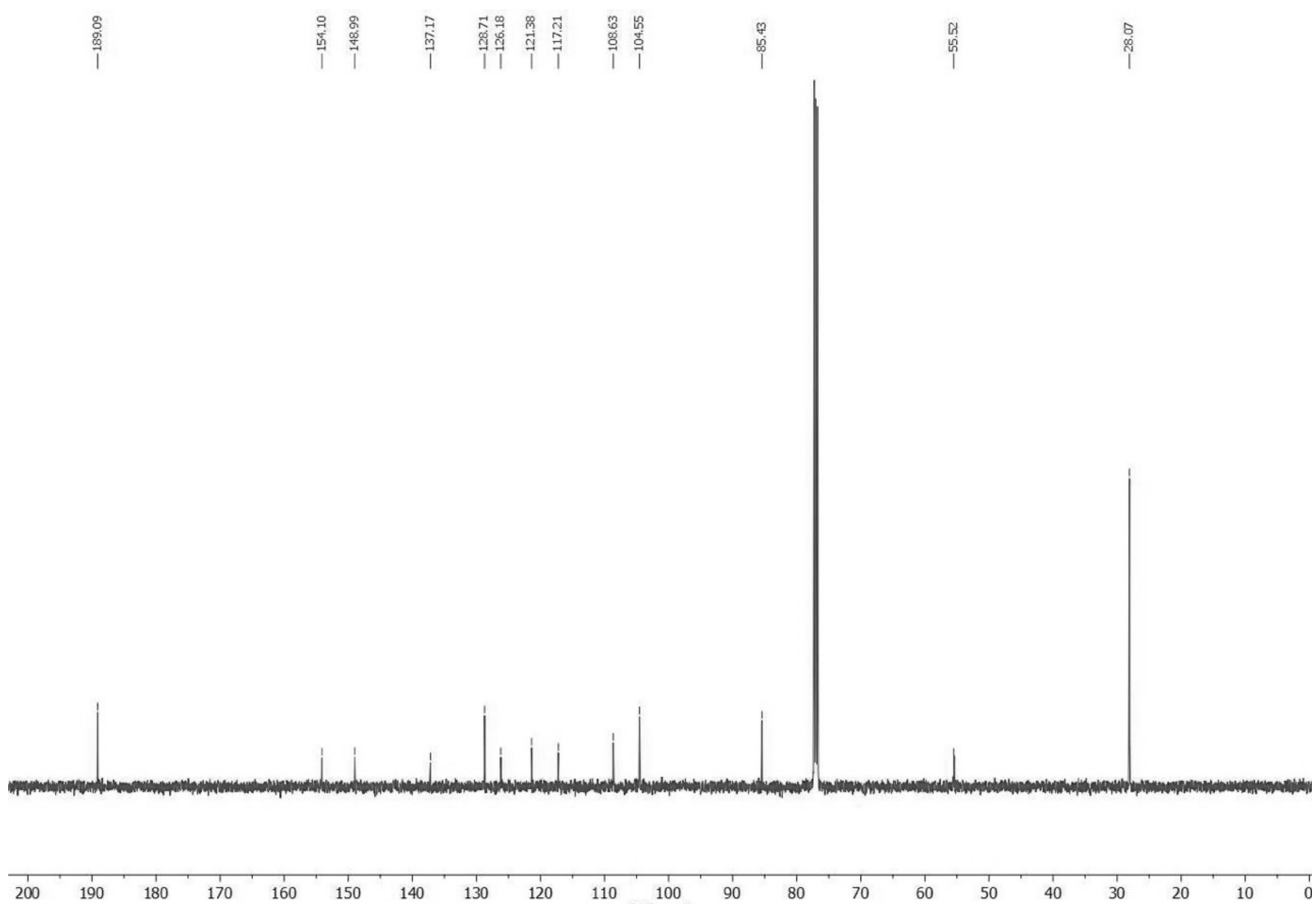

NMR (^{1}H 500 MHz, $CDCl_3$) of *tert*-butyl 3-formyl-4-OBn-1*H*-indole-1-carboxylate (S2b)

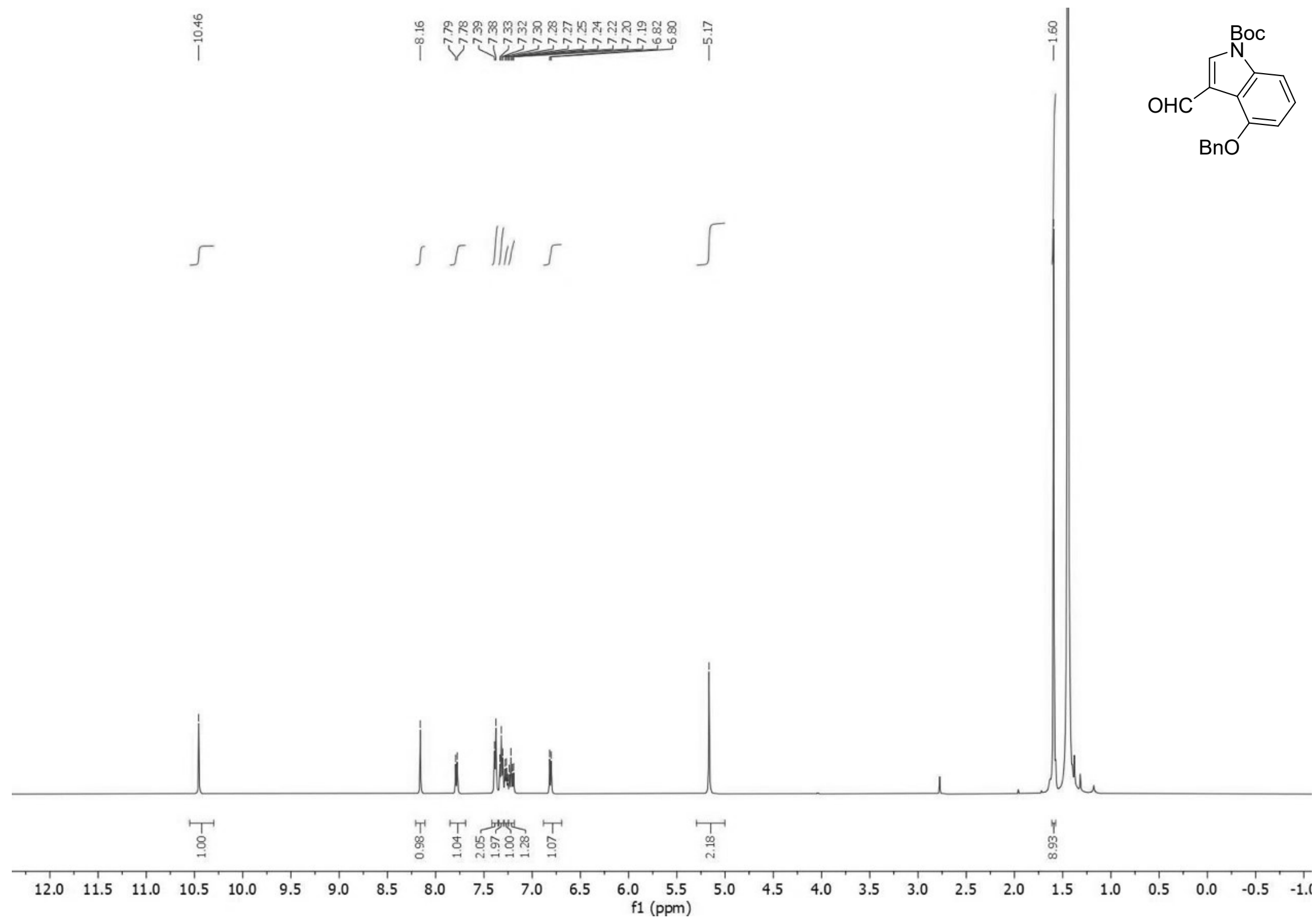

NMR (^{13}C 125 MHz, $CDCl_3$) of *tert*-butyl 3-formyl-4-OBn-1*H*-indole-1-carboxylate (S2b)

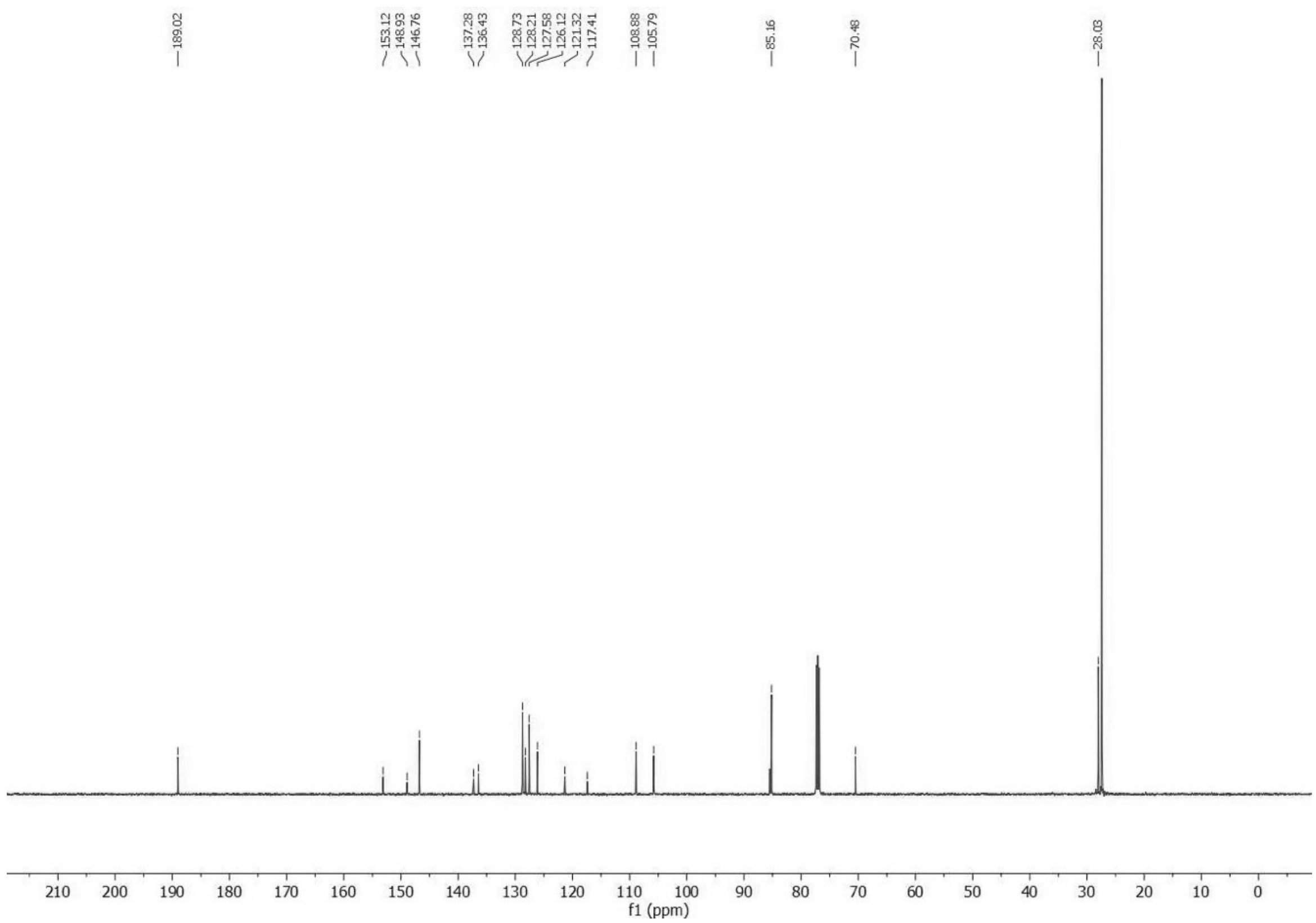

NMR (^{1}H 500 MHz, $CDCl_3$) of *tert*-butyl (*Z*)-3-(2-((*tert*-butoxycarbonyl)amino)-3-methoxy-3-oxoprop-1-en-1-yl)-4-methoxy-1*H*-indole-1-carboxylate (S3a)

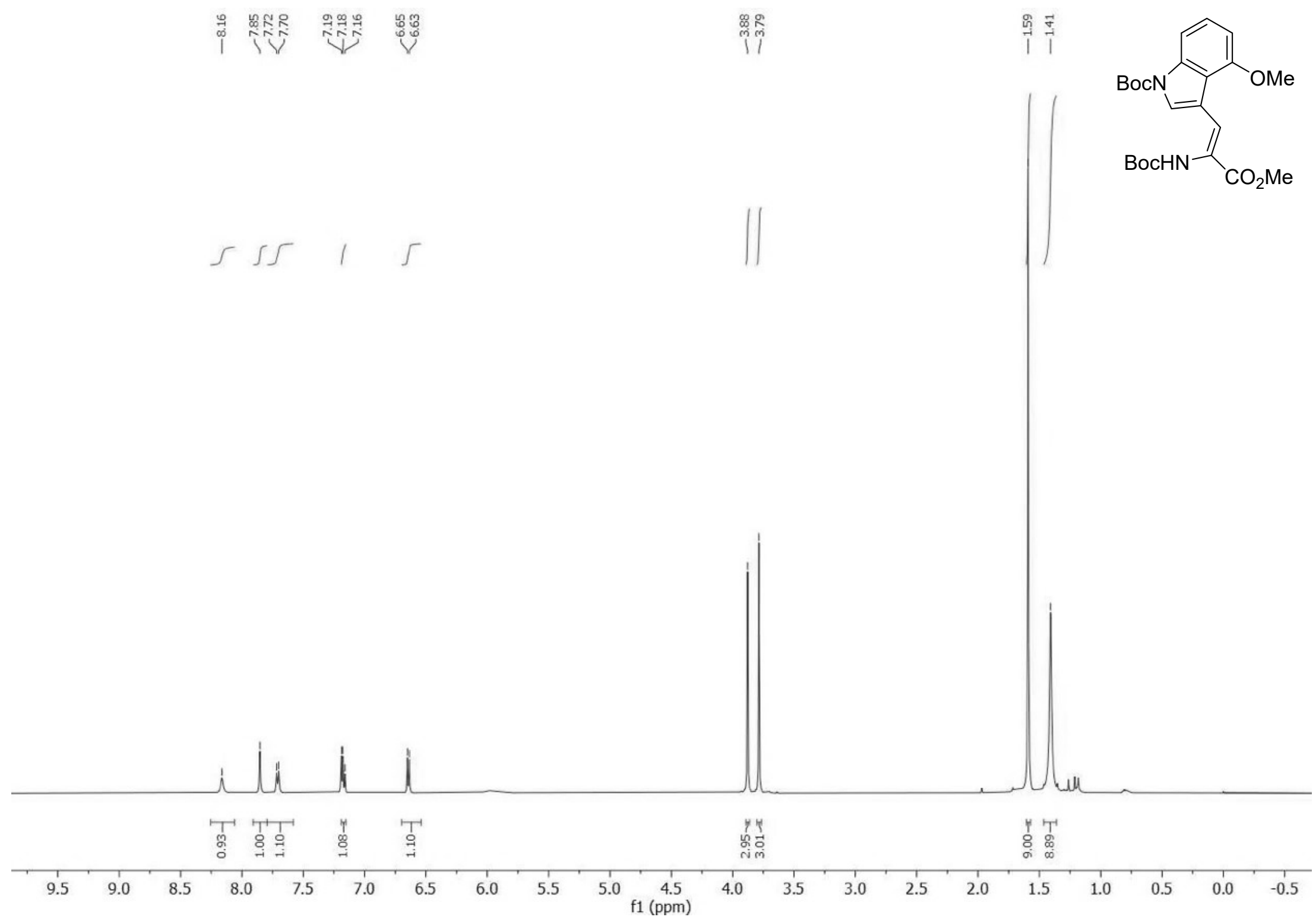

NMR (^{13}C 125 MHz, $CDCl_3$) of *tert*-butyl (*Z*)-3-(2-((*tert*-butoxycarbonyl)amino)-3-methoxy-3-oxoprop-1-en-1-yl)-4-methoxy-1*H*-indole-1-carboxylate (S3a)

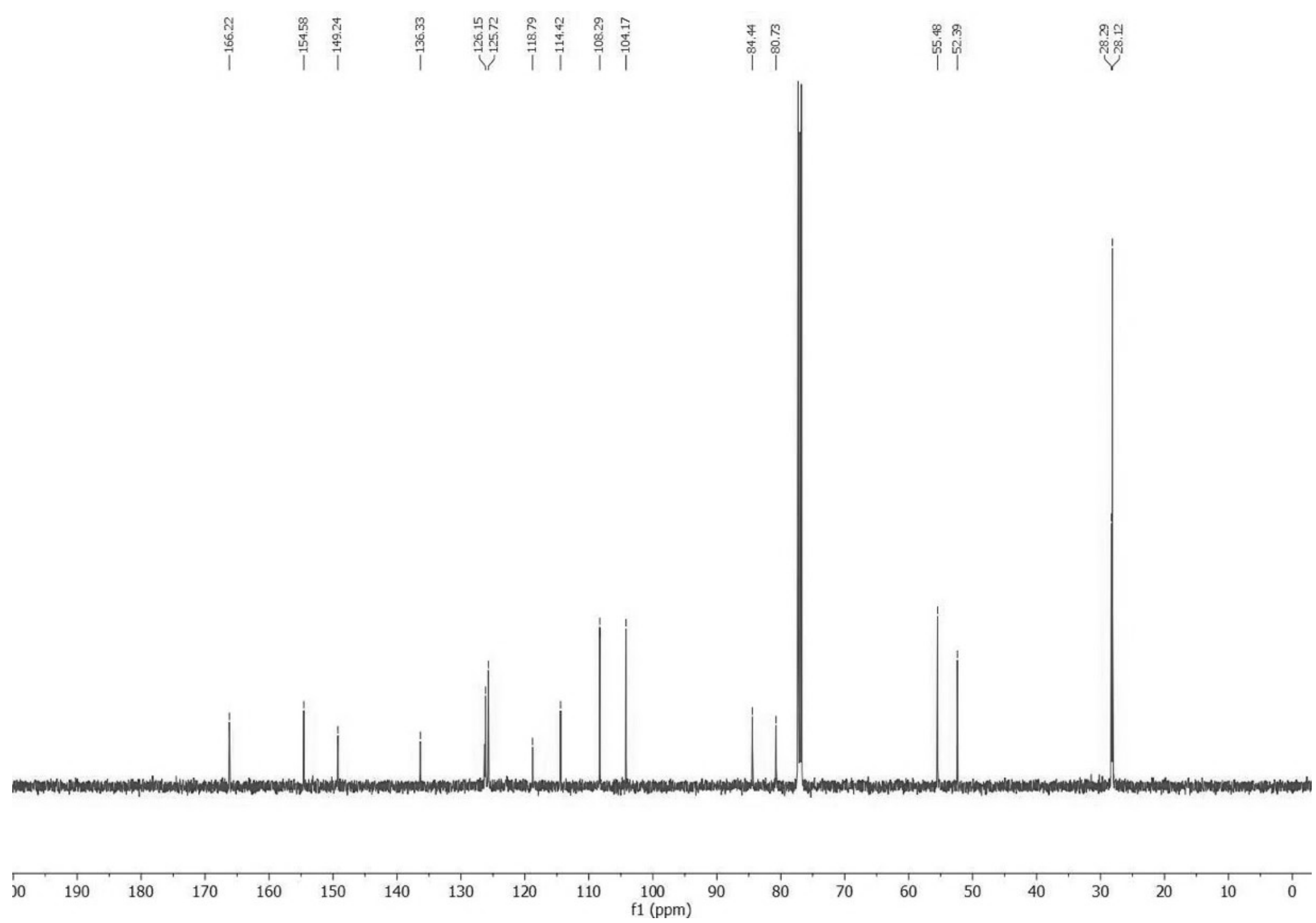

NMR (^{1}H 500 MHz, CDCl$_3$) of *tert*-butyl (*Z*)-4-(benzyloxy)-3-(2-((*tert*-butoxycarbonyl)amino)-3-methoxy-3-oxoprop-1-en-1-yl)-1*H*-indole-1-carboxylate (S3b)

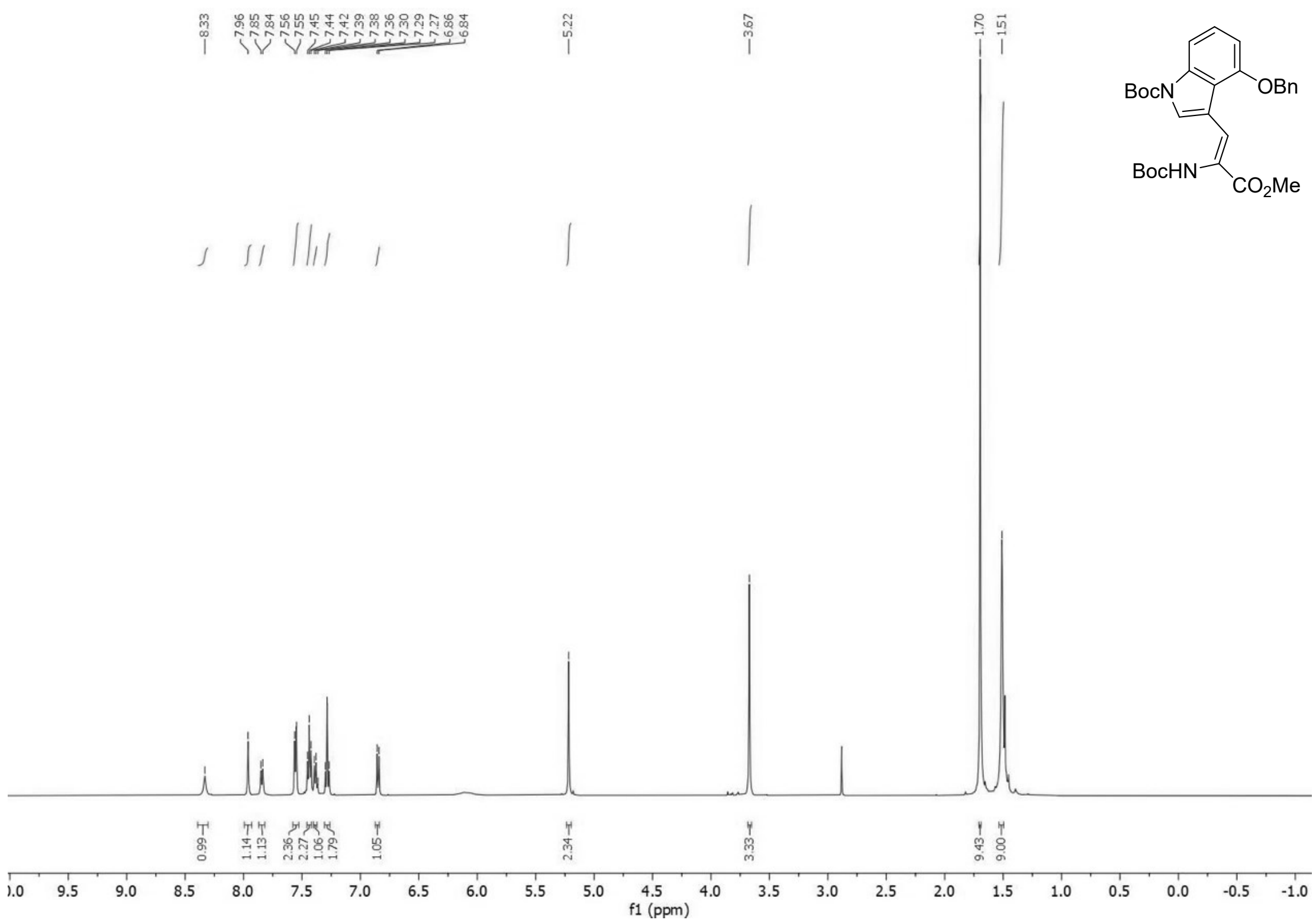

NMR (^{13}C 125 MHz, CDCl$_3$) of *tert*-butyl (*Z*)-4-(benzyloxy)-3-(2-((*tert*-butoxycarbonyl)amino)-3-methoxy-3-oxoprop-1-en-1-yl)-1*H*-indole-1-carboxylate (S3b)

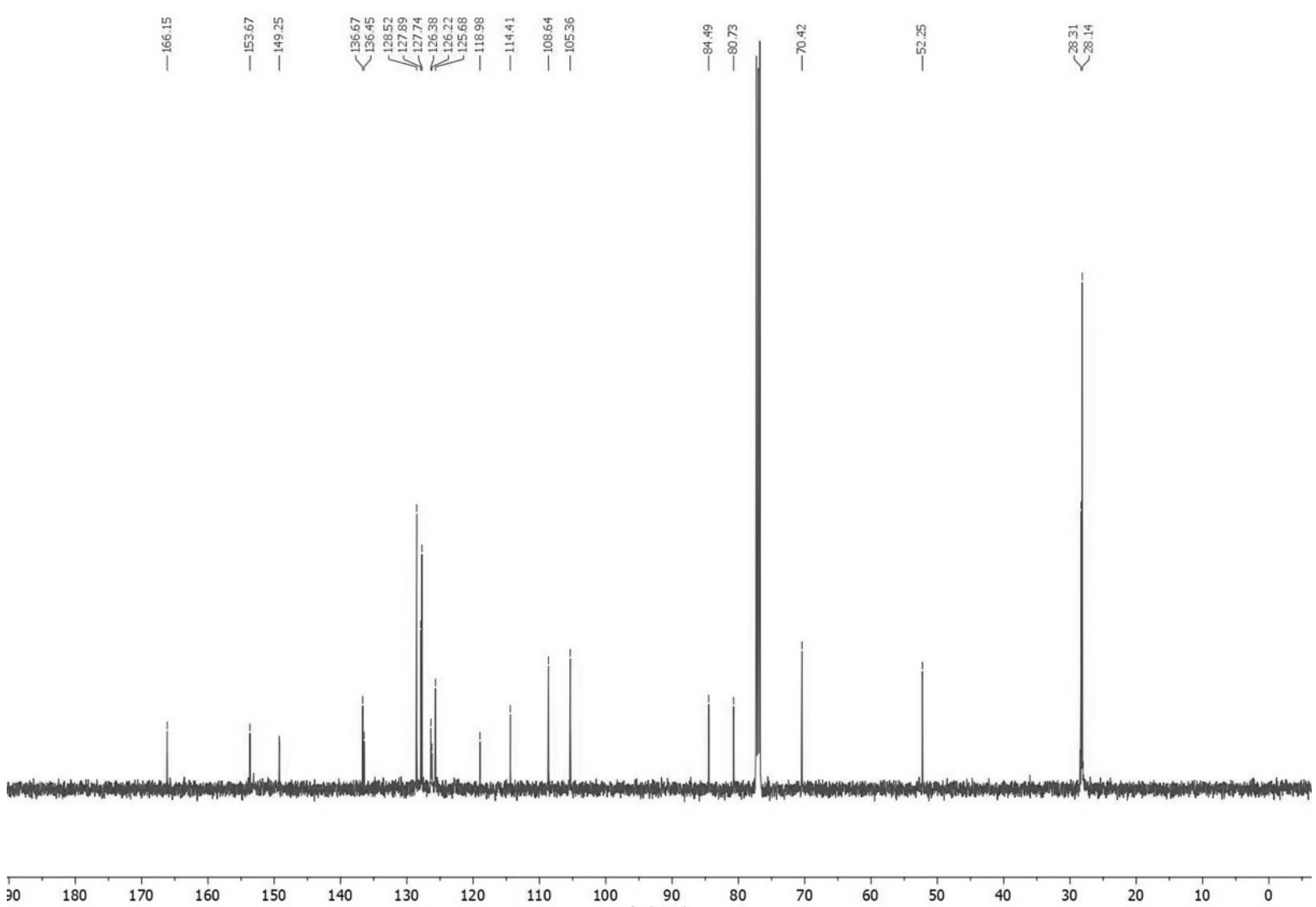

NMR (^{1}H 500 MHz, DMSO-d_6) of Fmoc-L-Trp(4-OMe)-OH (7a)

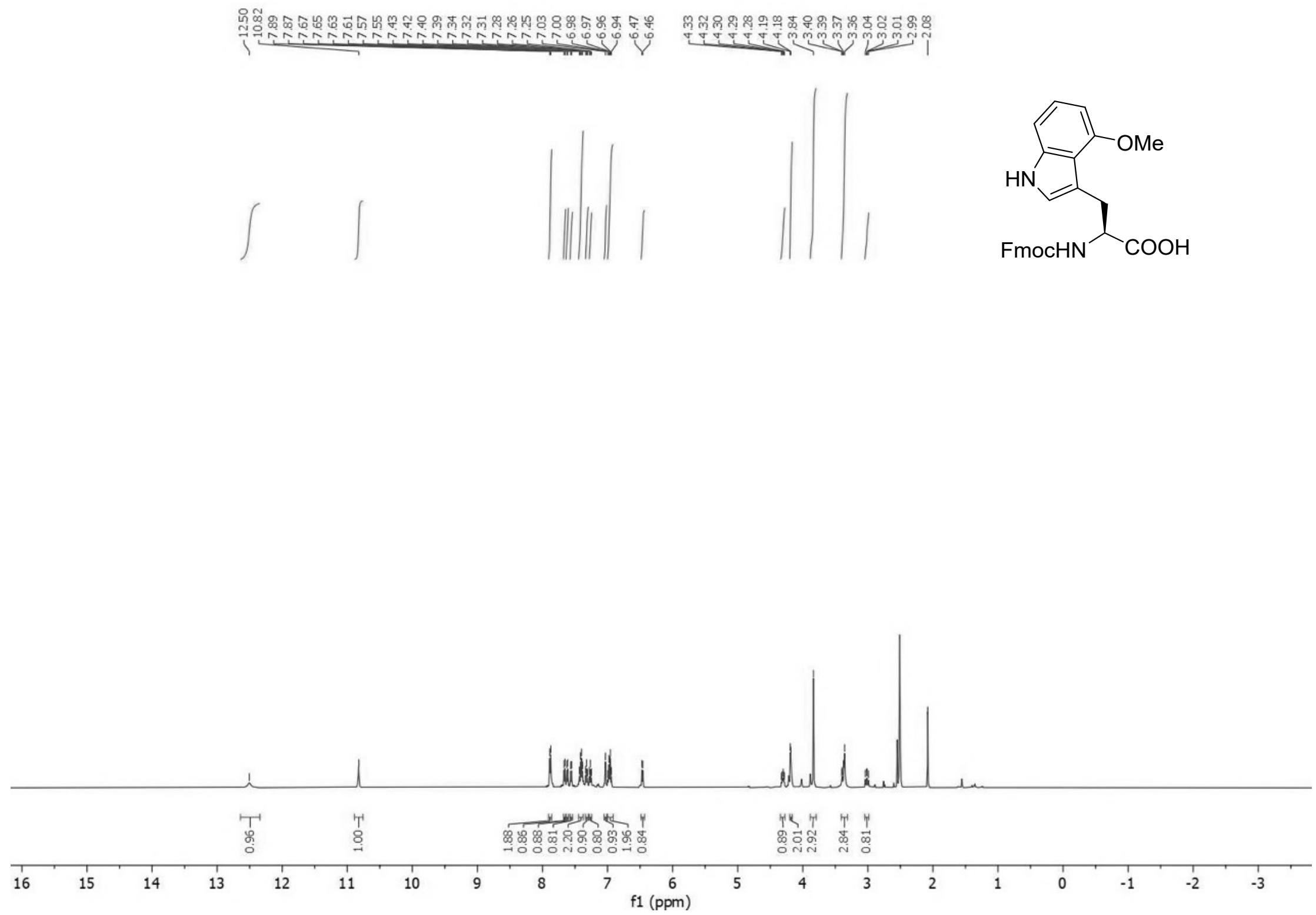

NMR (^{13}C 125 MHz, DMSO-d_6) of Fmoc-L-Trp(4-OMe)-OH (7a)

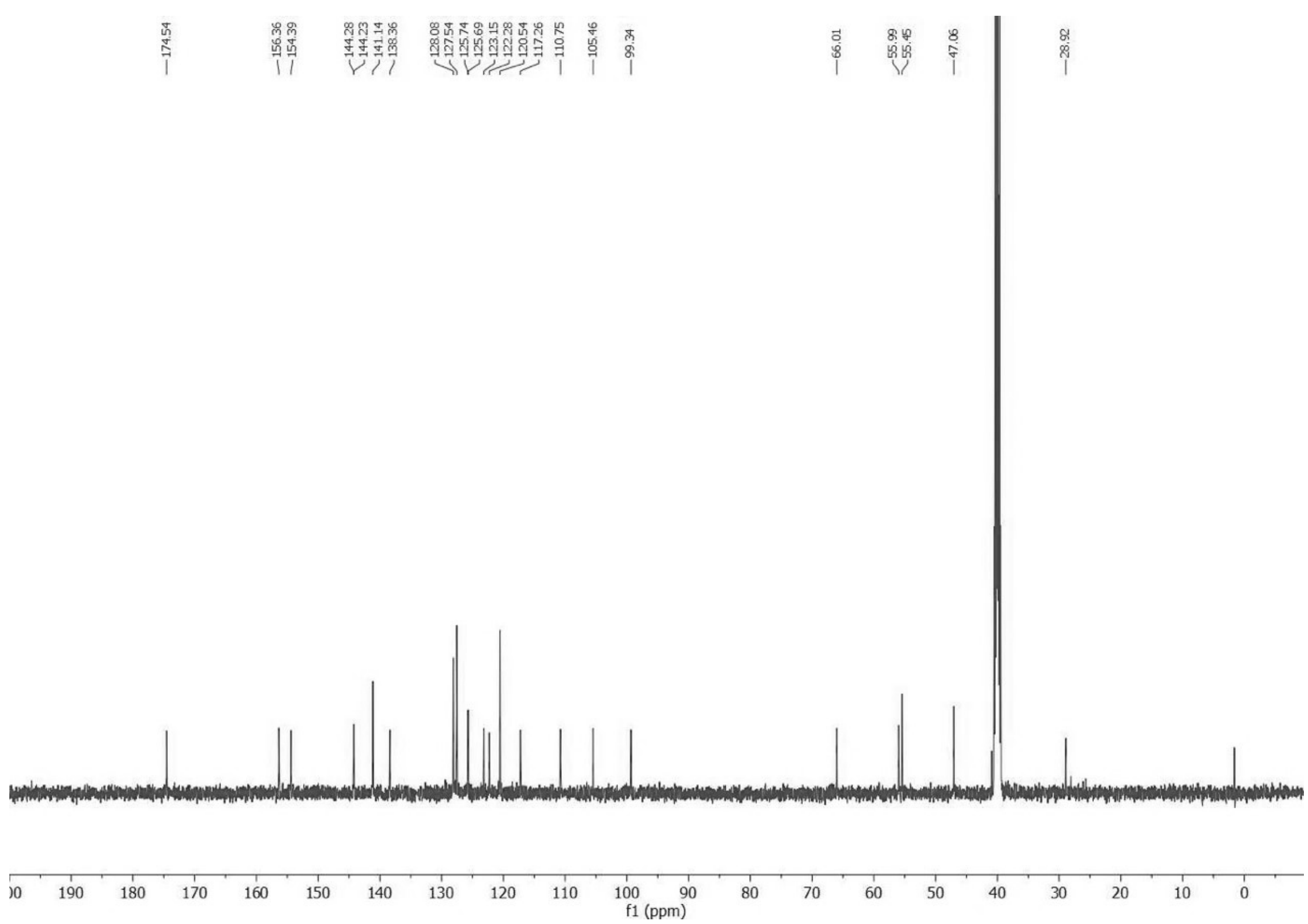

NMR (^{1}H 500 MHz, DMSO-d_6) of Fmoc-L-Trp(4-OBn)-OH (7b)

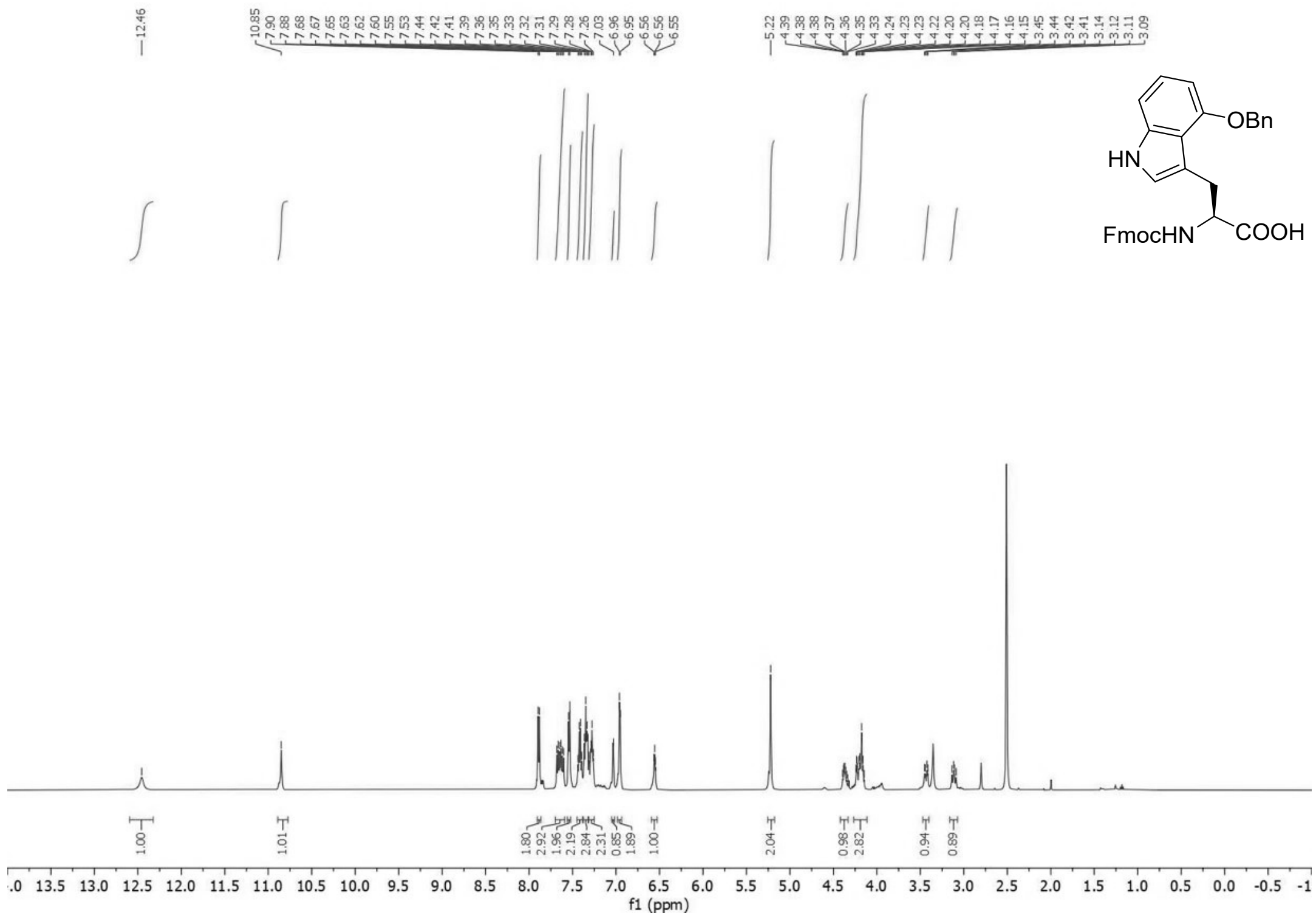

NMR (^{13}C 125 MHz, DMSO-d_6) of Fmoc-L-Trp(4-OBn)-OH (7b)

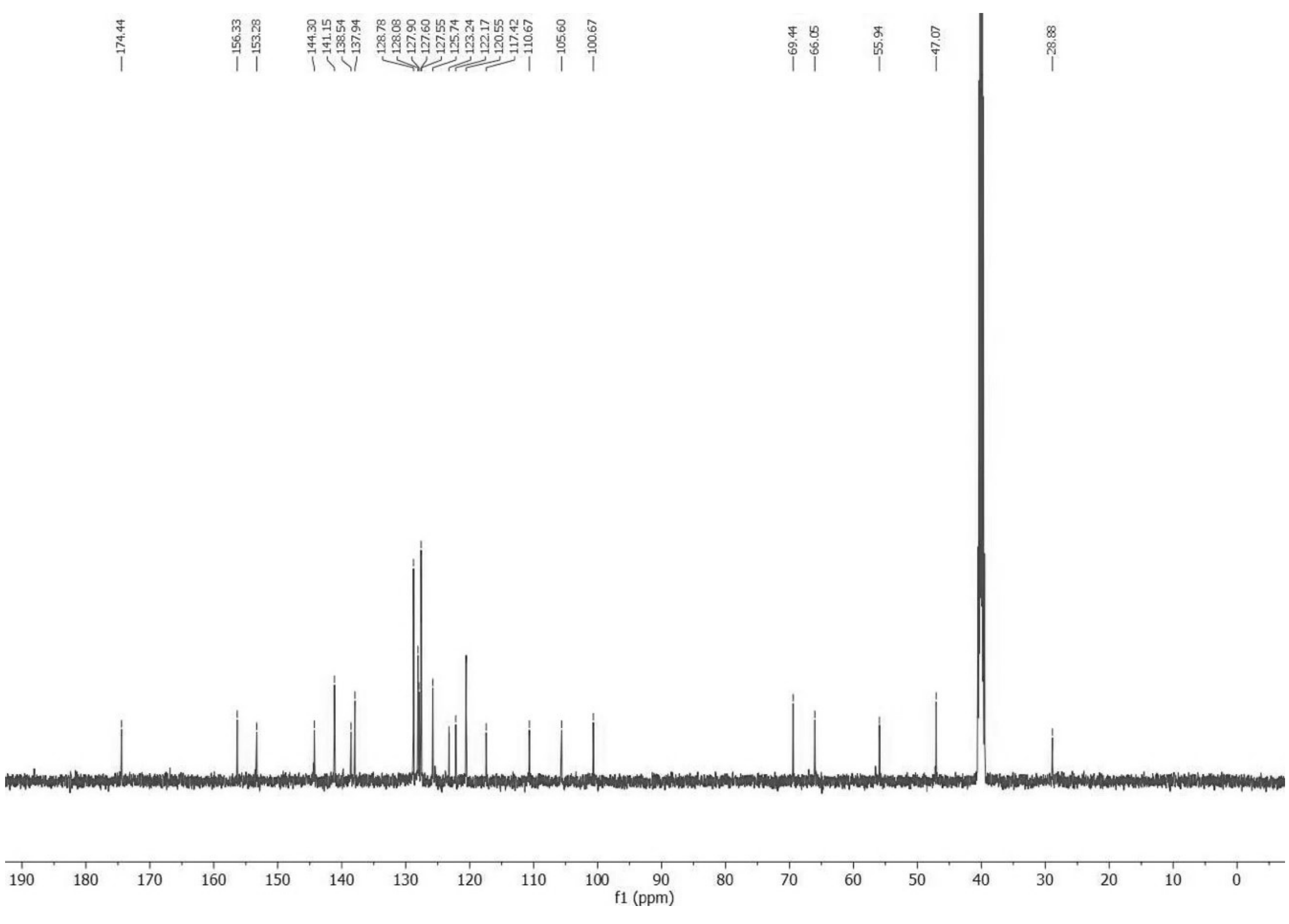

NMR (^{1}H, 700 MHz, DMSO-d_6) of cortinarin A (1a)

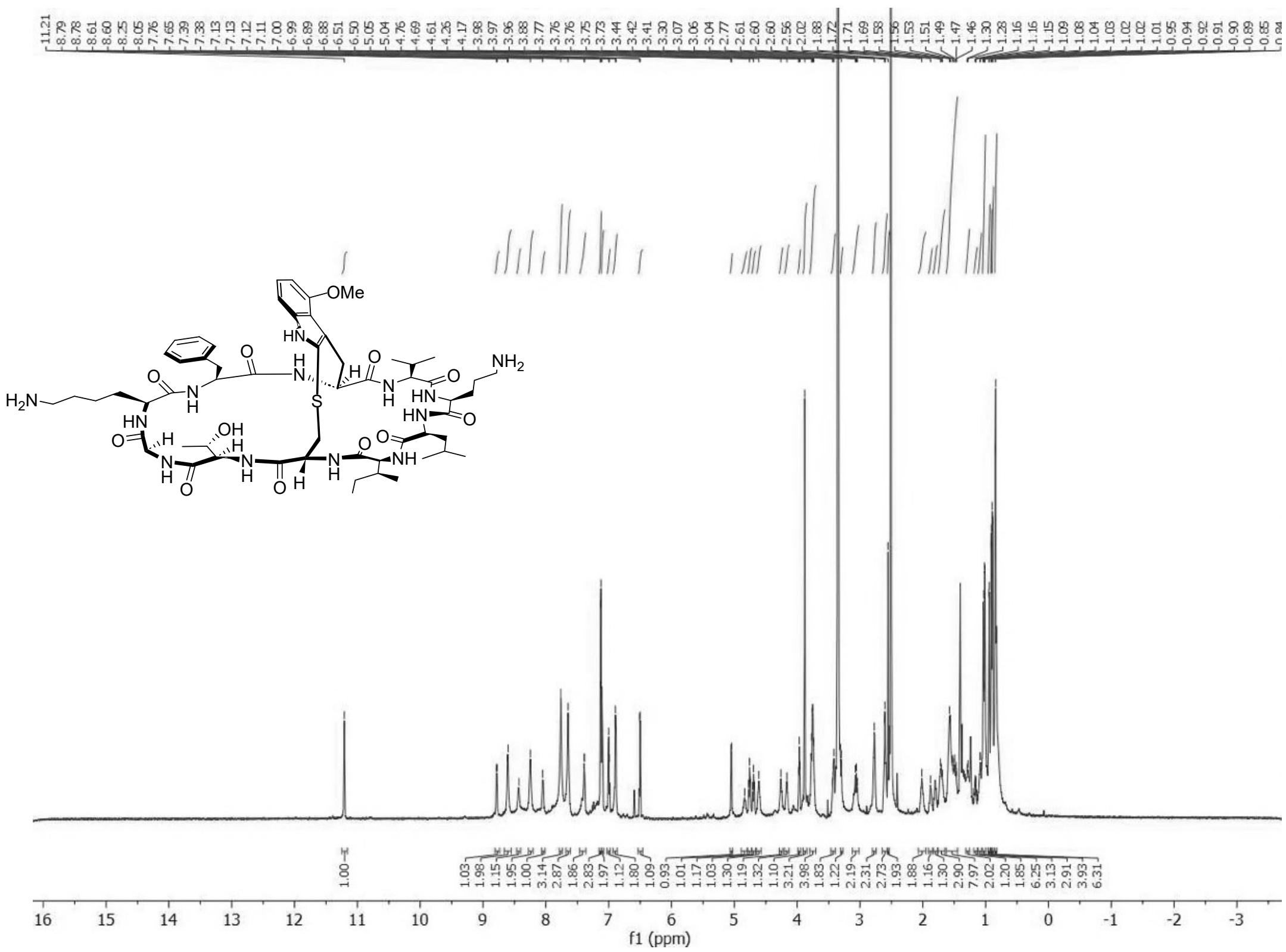

NMR (^{1}H, ^{13}C-HQSC, 126 MHz, DMSO-d_6) of cortinarin A (1a)

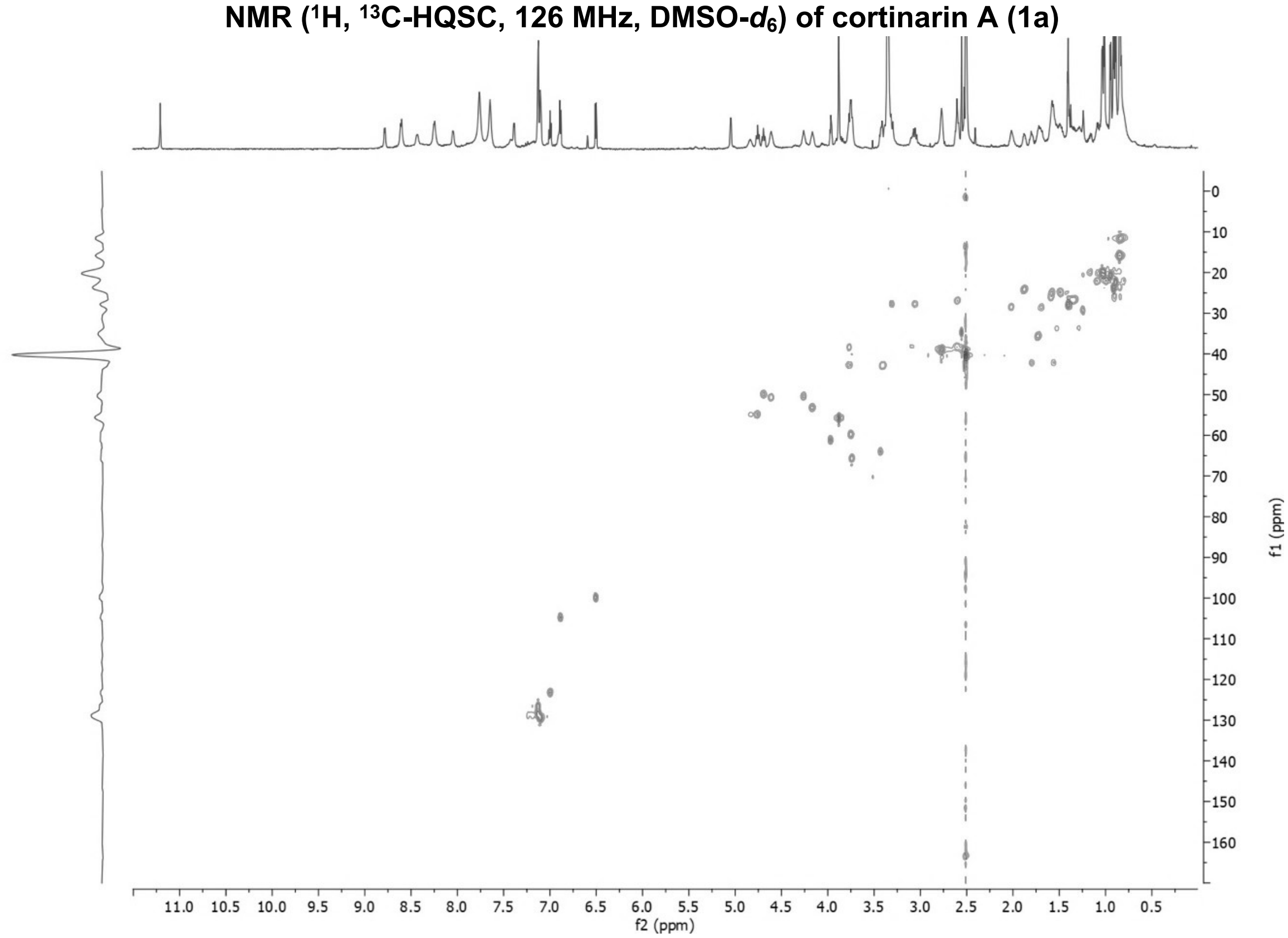

NMR (^{1}H,^{1}H-COSY, 700 MHz, DMSO-d_6) of cortinarin A (1a)

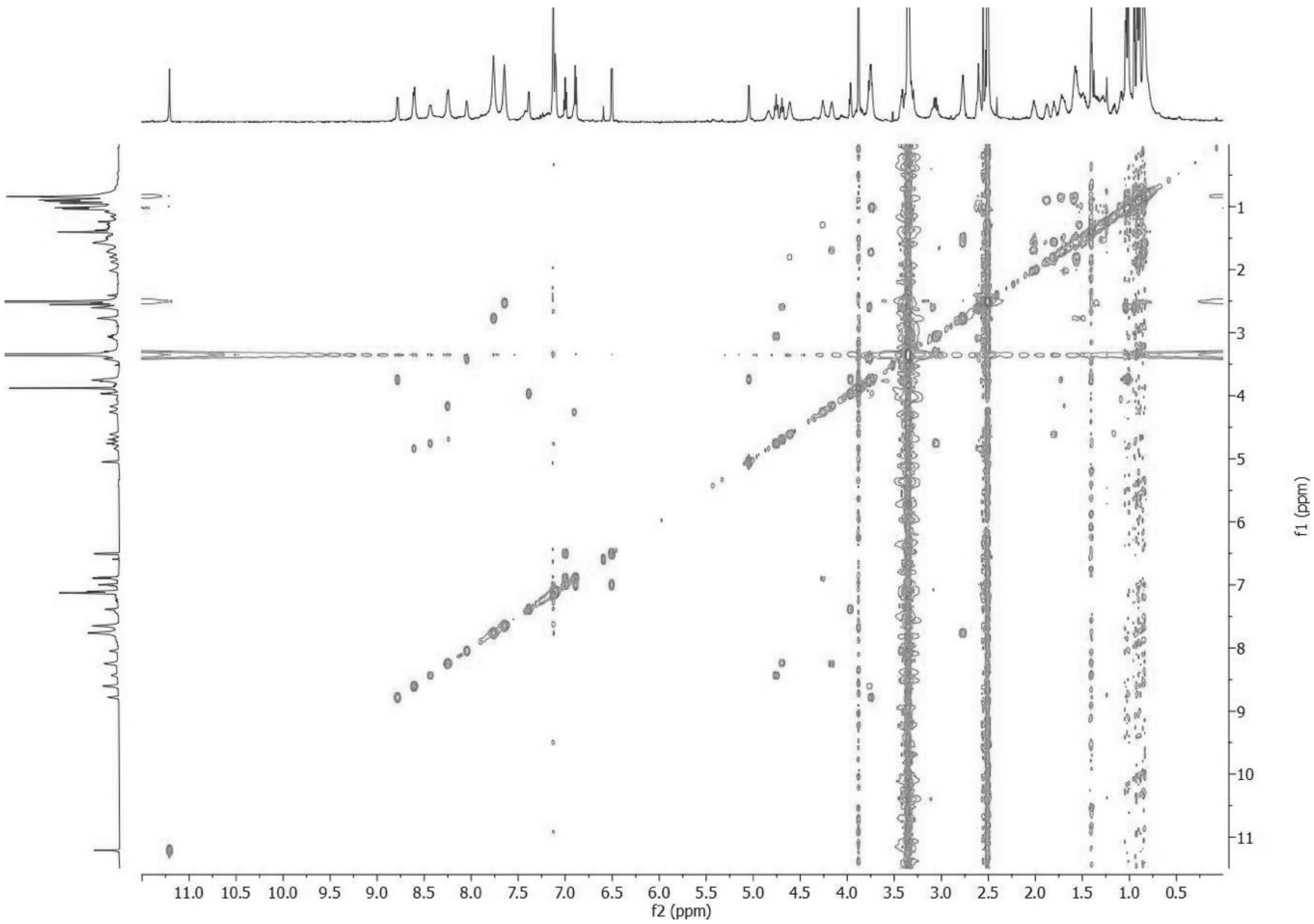

NMR (^{1}H,^{1}H-TOCSY, 700 MHz, DMSO-d_6) of cortinarin A (1a)

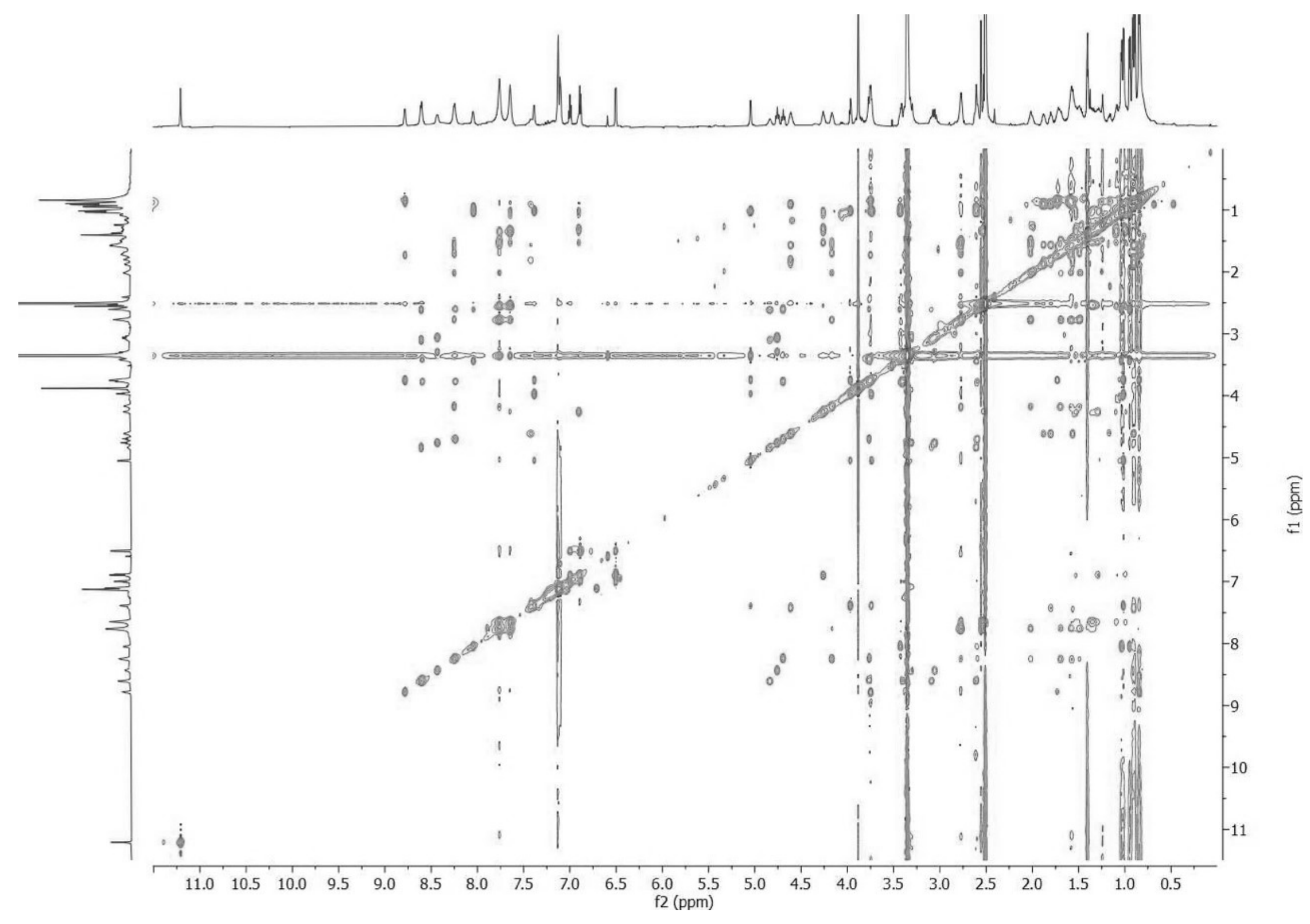

NMR (^{1}H,^{1}H-NOESY, 700 MHz, DMSO-d_6) of cortinarin A (1a)

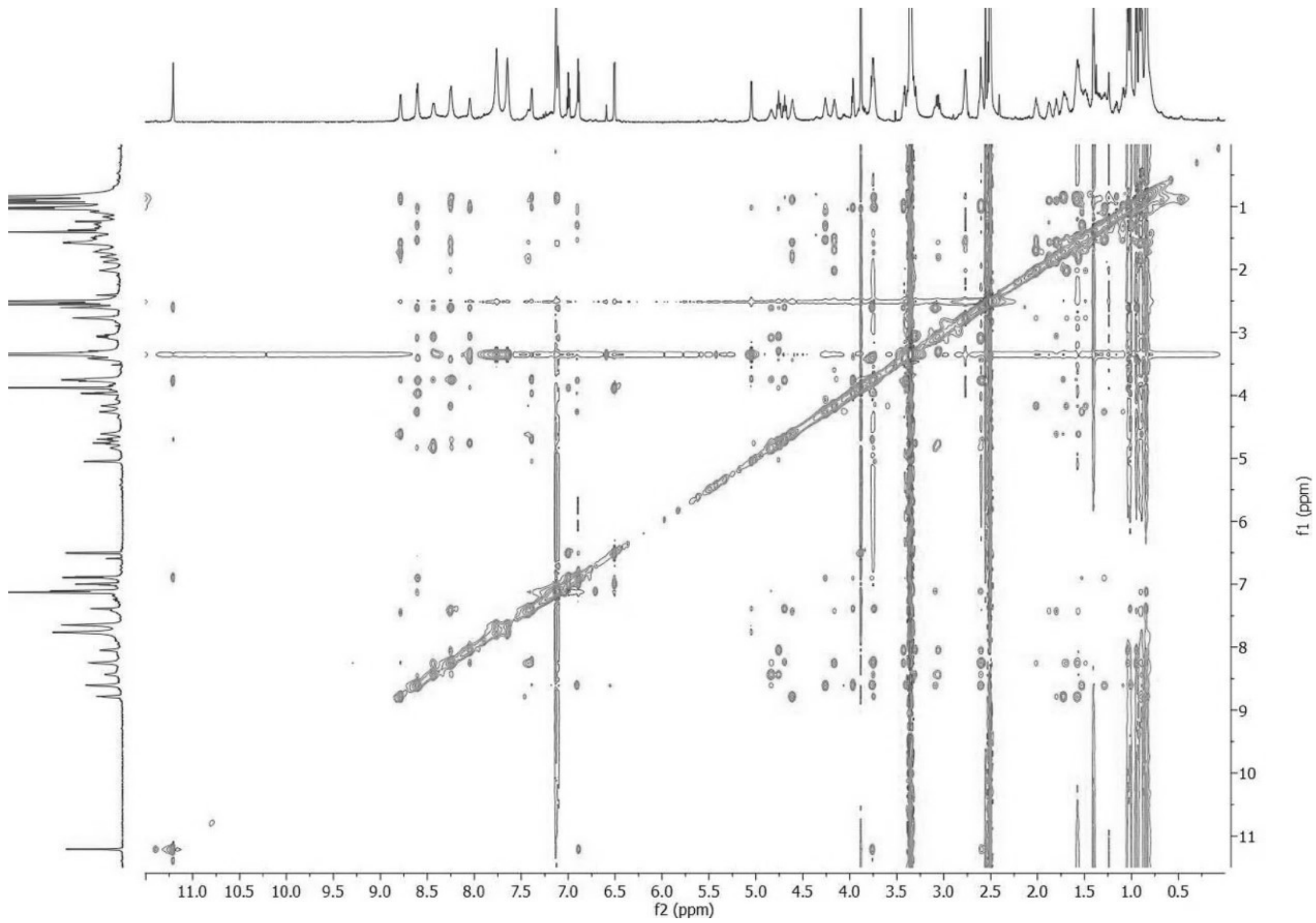

NMR (^{1}H, 700 MHz, DMSO-d_6) of cortinarin B (1b)

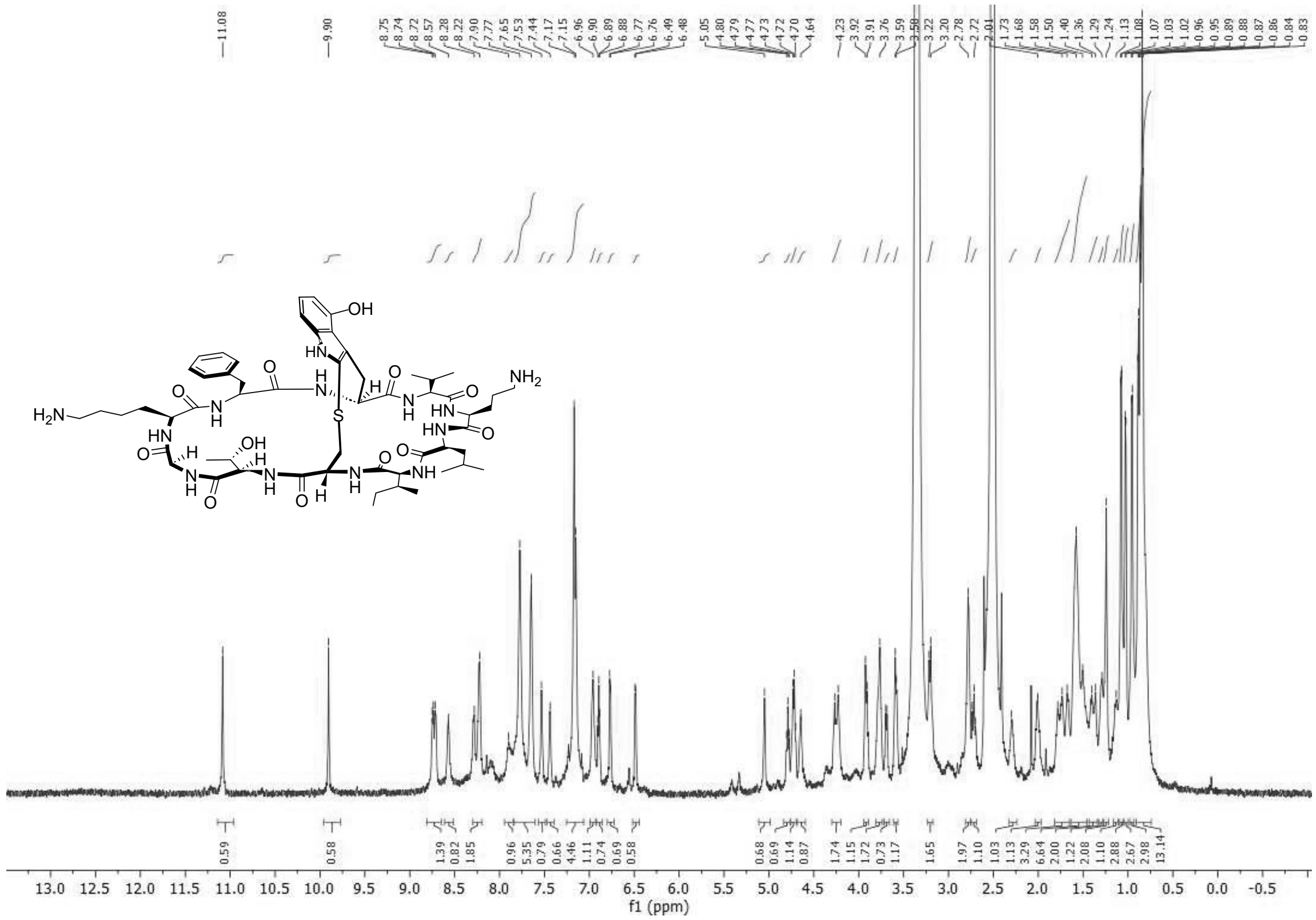

NMR (^{1}H,^{13}C-HQSC, 126 MHz, DMSO-d_6) of cortinarin B (1b)

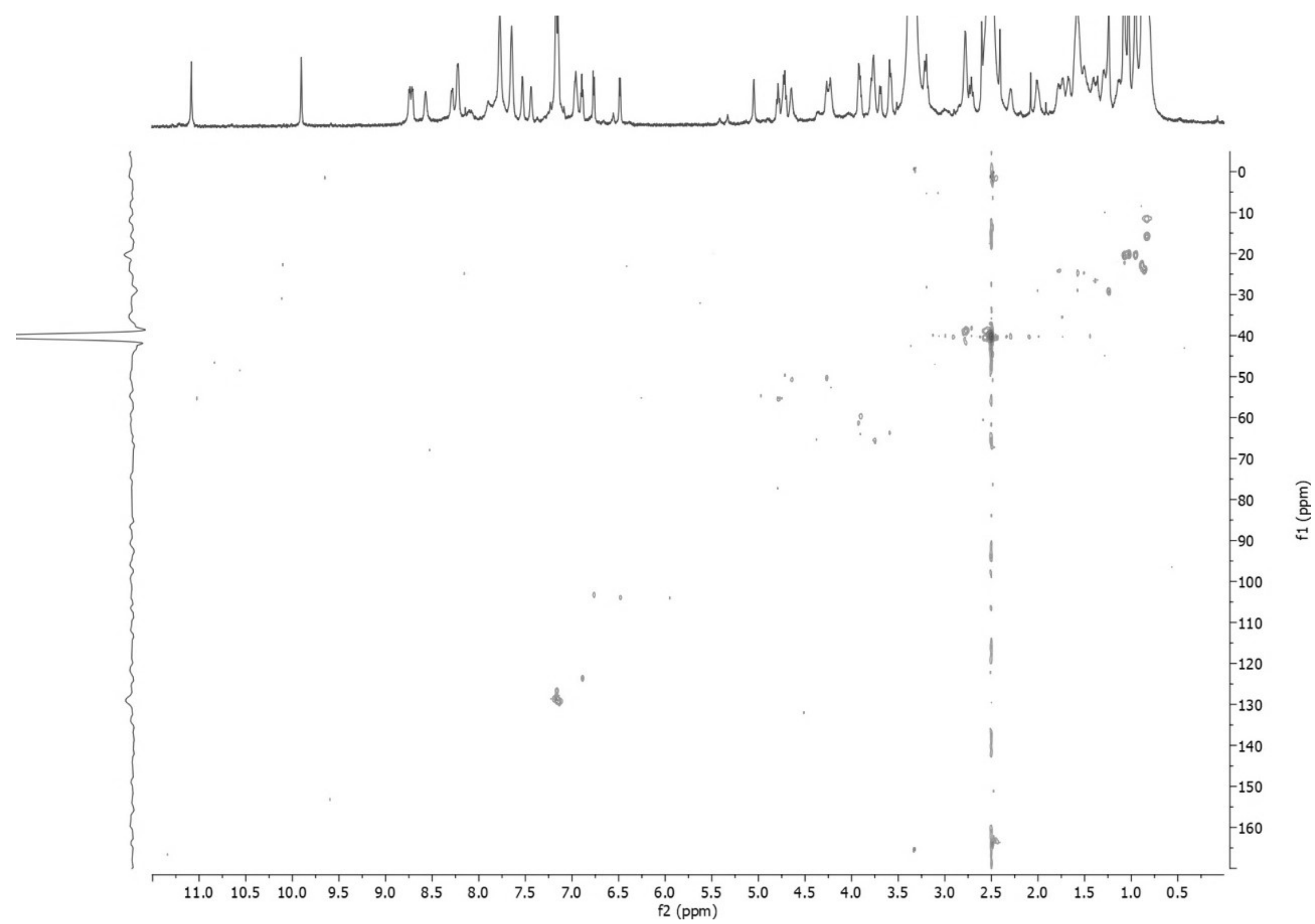

NMR (^{1}H,^{1}H-COSY, 700 MHz, DMSO-d_6) of cortinarin B (1b)

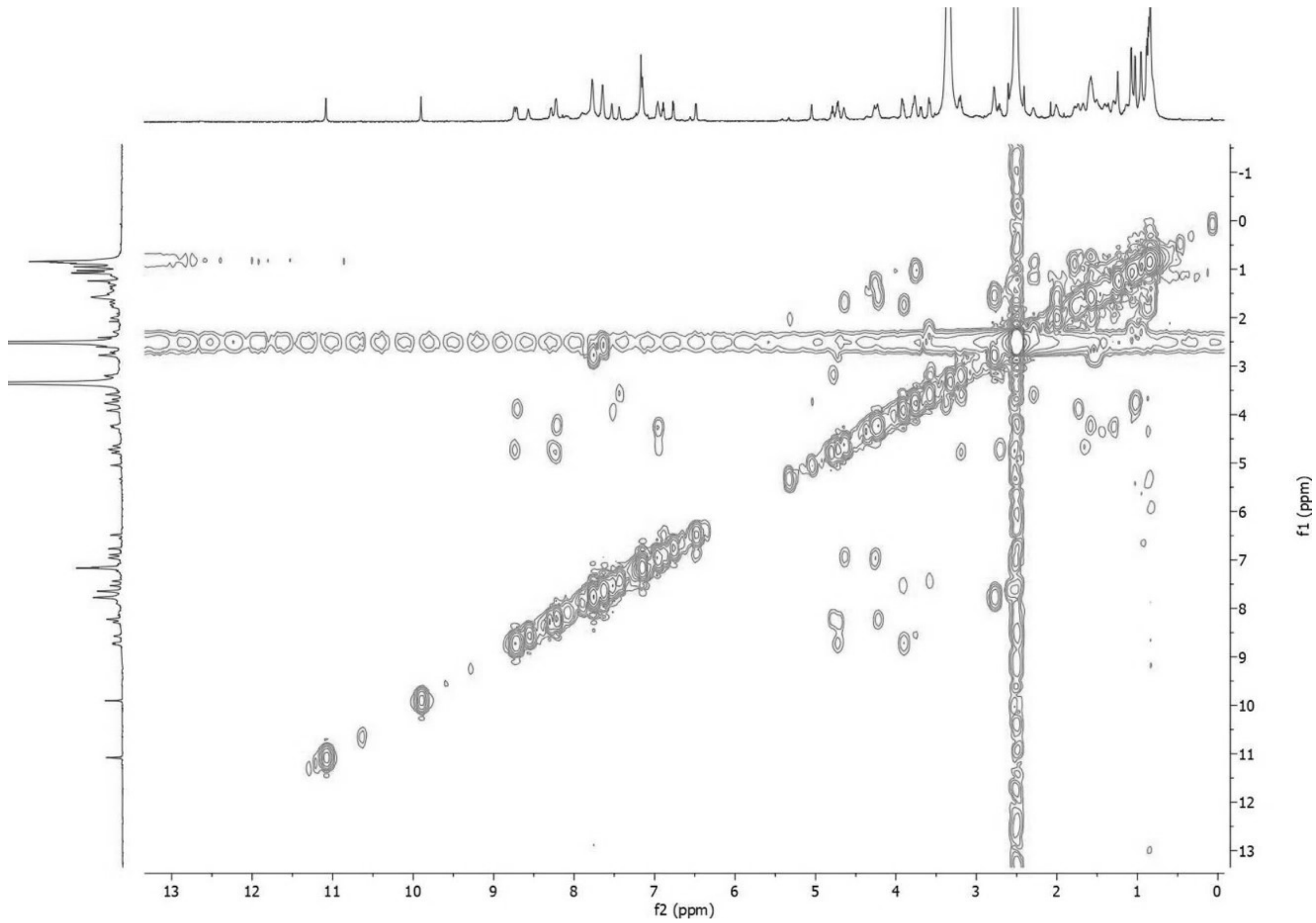

NMR (^{1}H,^{1}H-TOCSY, 700 MHz, DMSO-d_6) of cortinarin B (1b)

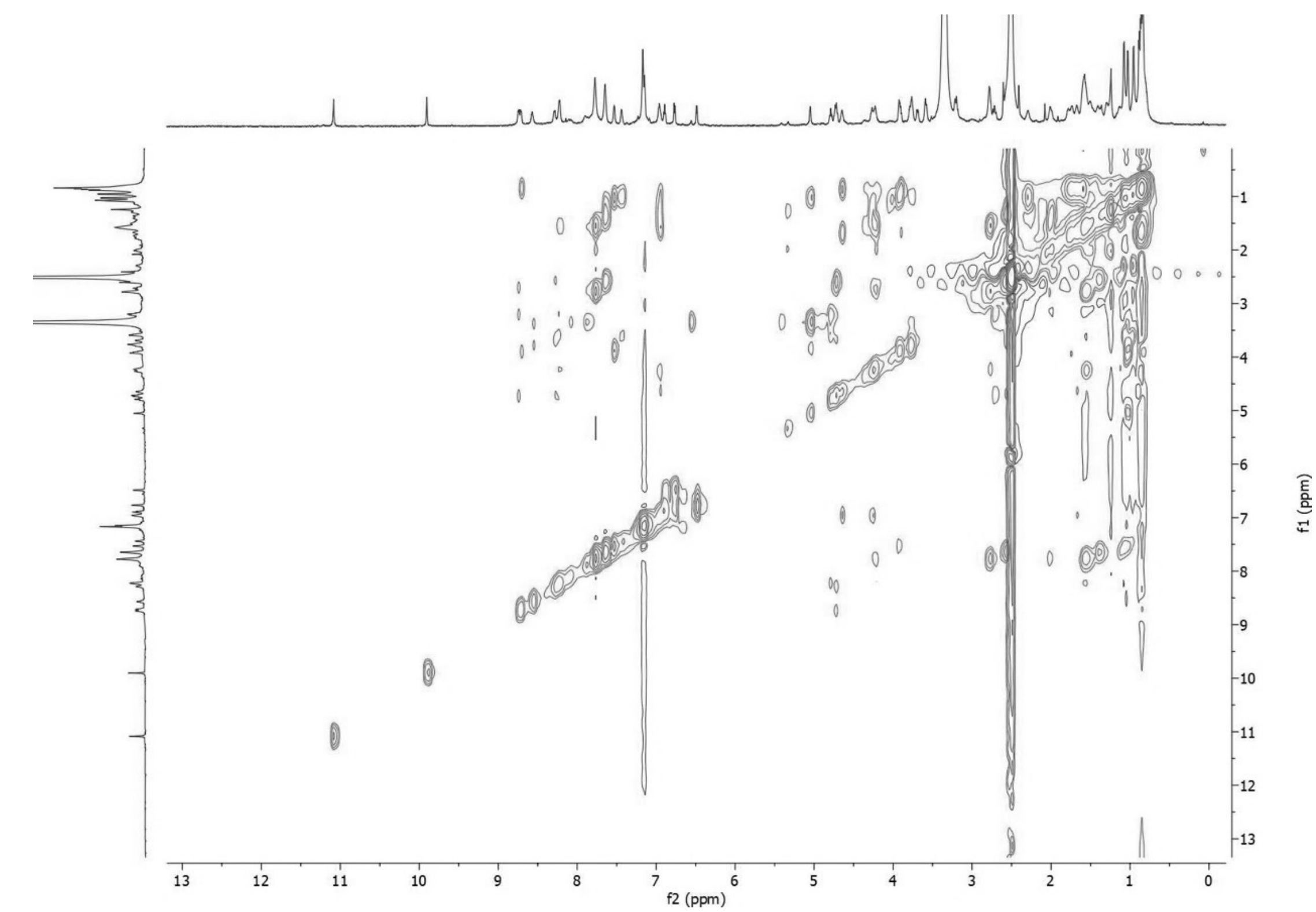

NMR (^{1}H,^{1}H-NOESY, 700 MHz, DMSO-d_6) of cortinarin B (1b)

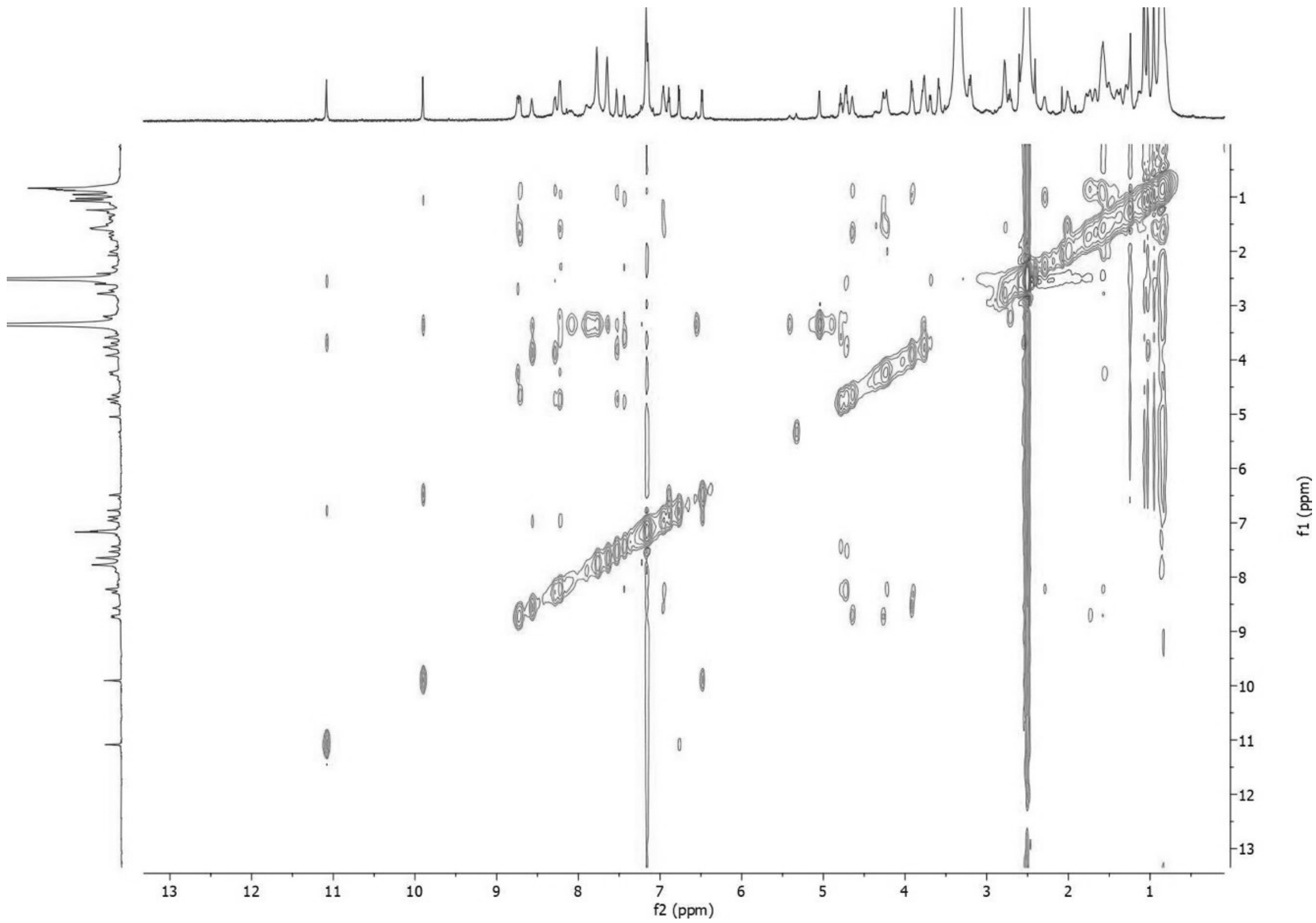

NMR (^{1}H, 700 MHz, DMSO-d_6) of cortinarin C (2)

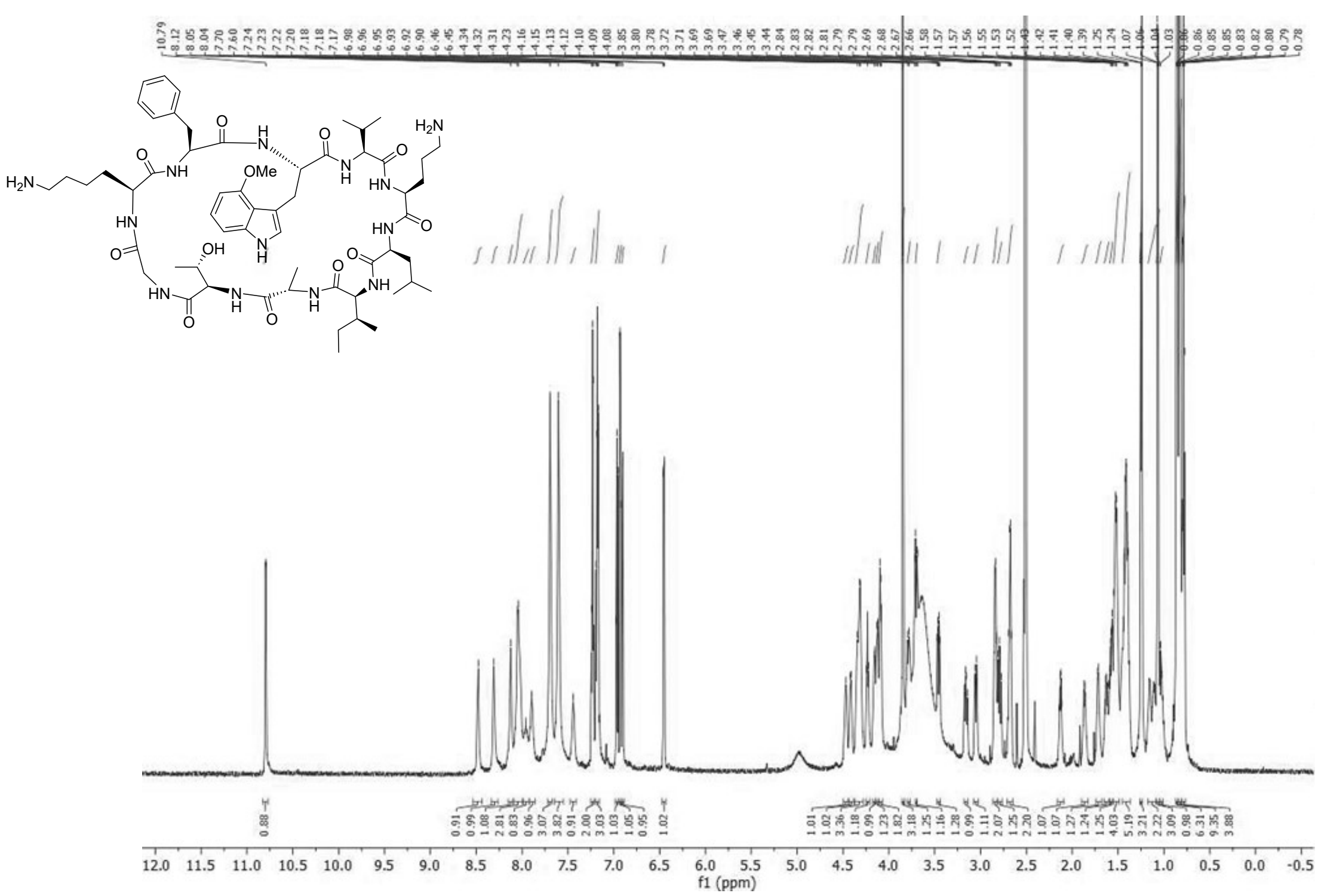

NMR (^{1}H,^{13}C-HQSC, 126 MHz, DMSO-d_6) of cortinarin C (2)

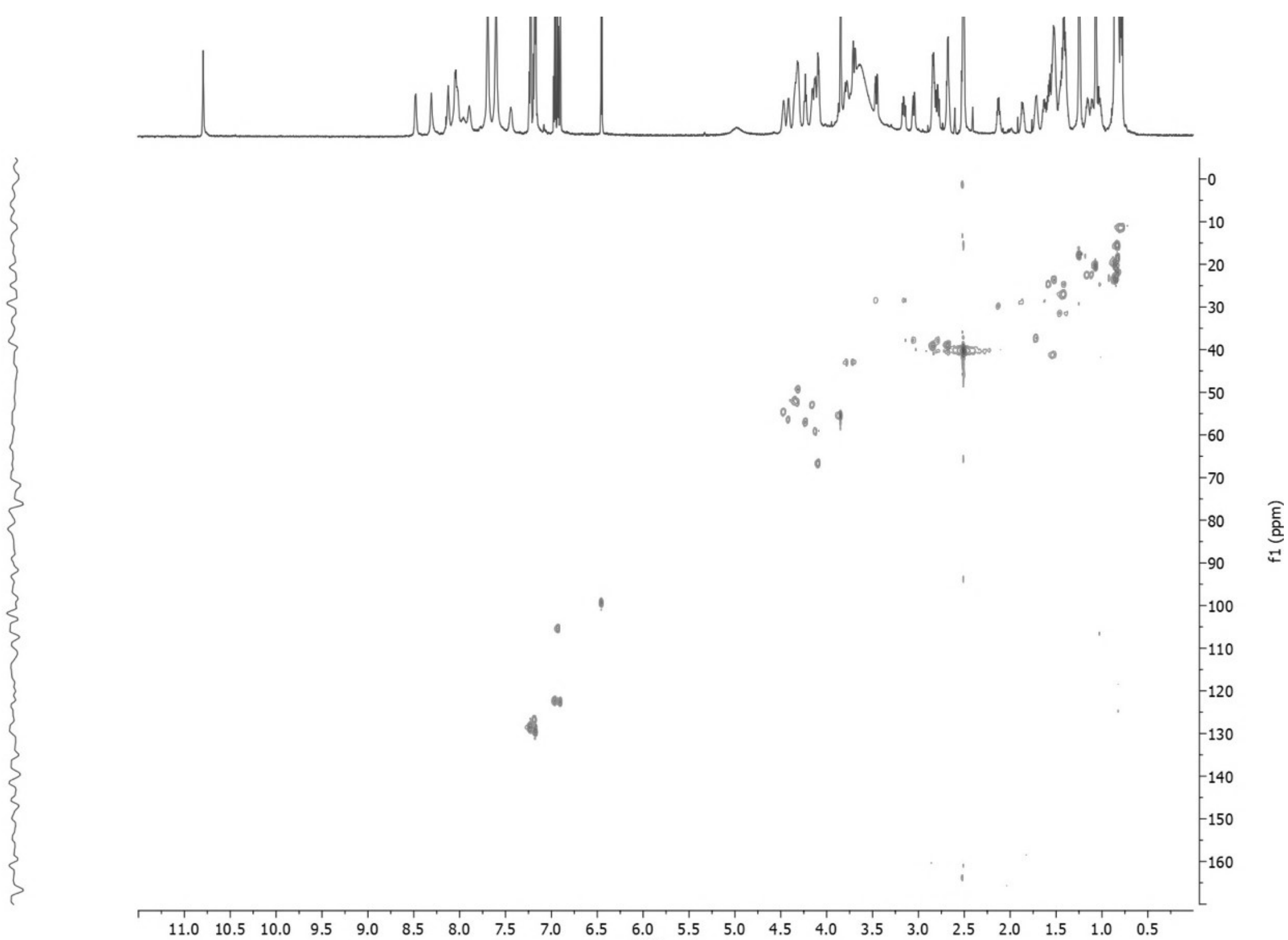

NMR (^1H,^1H-COSY, 700 MHz, DMSO-d_6) of cortinarin C (2)

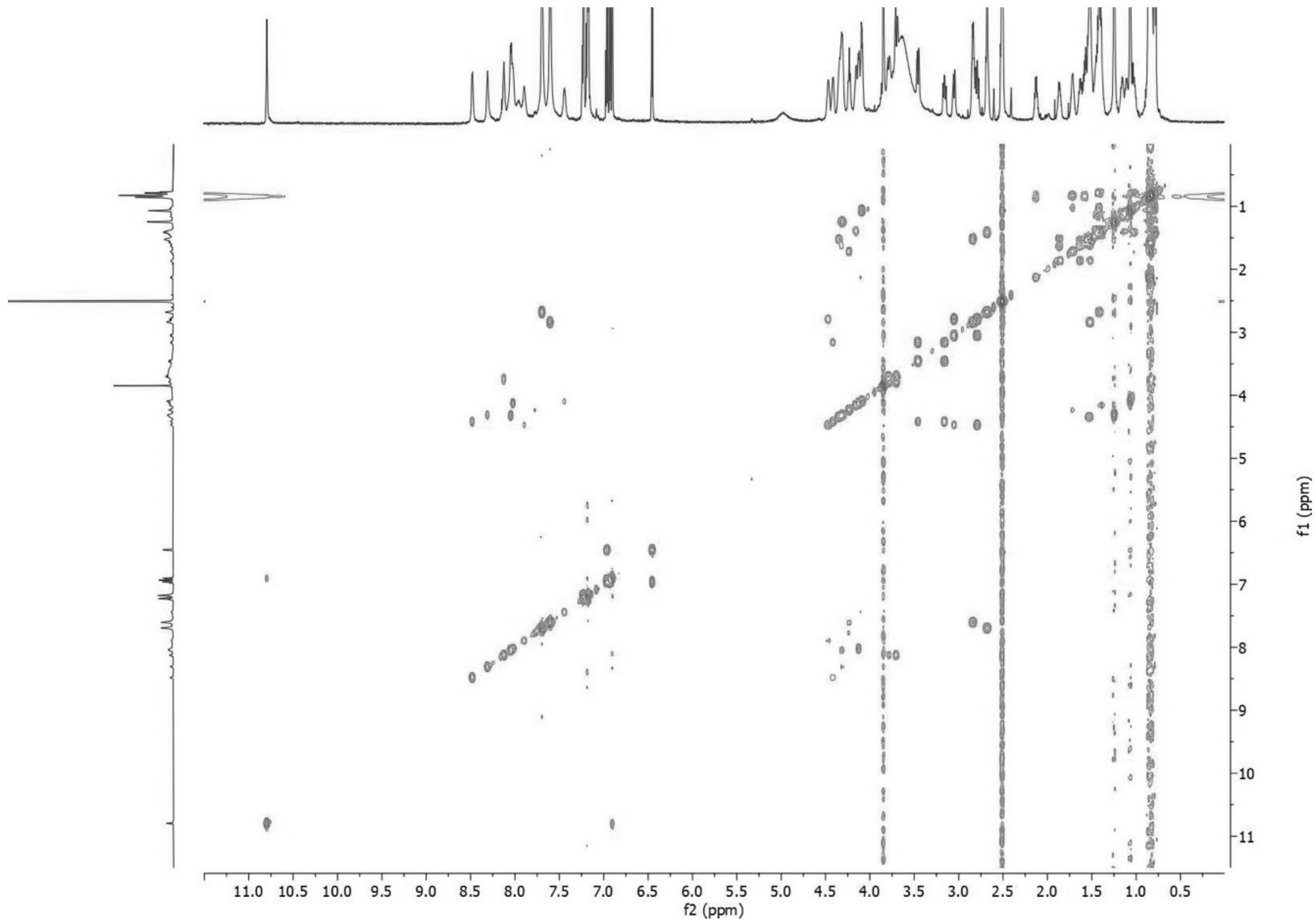

NMR (^1H,^1H-TOCSY, 700 MHz, DMSO-d_6) of cortinarin C (2)

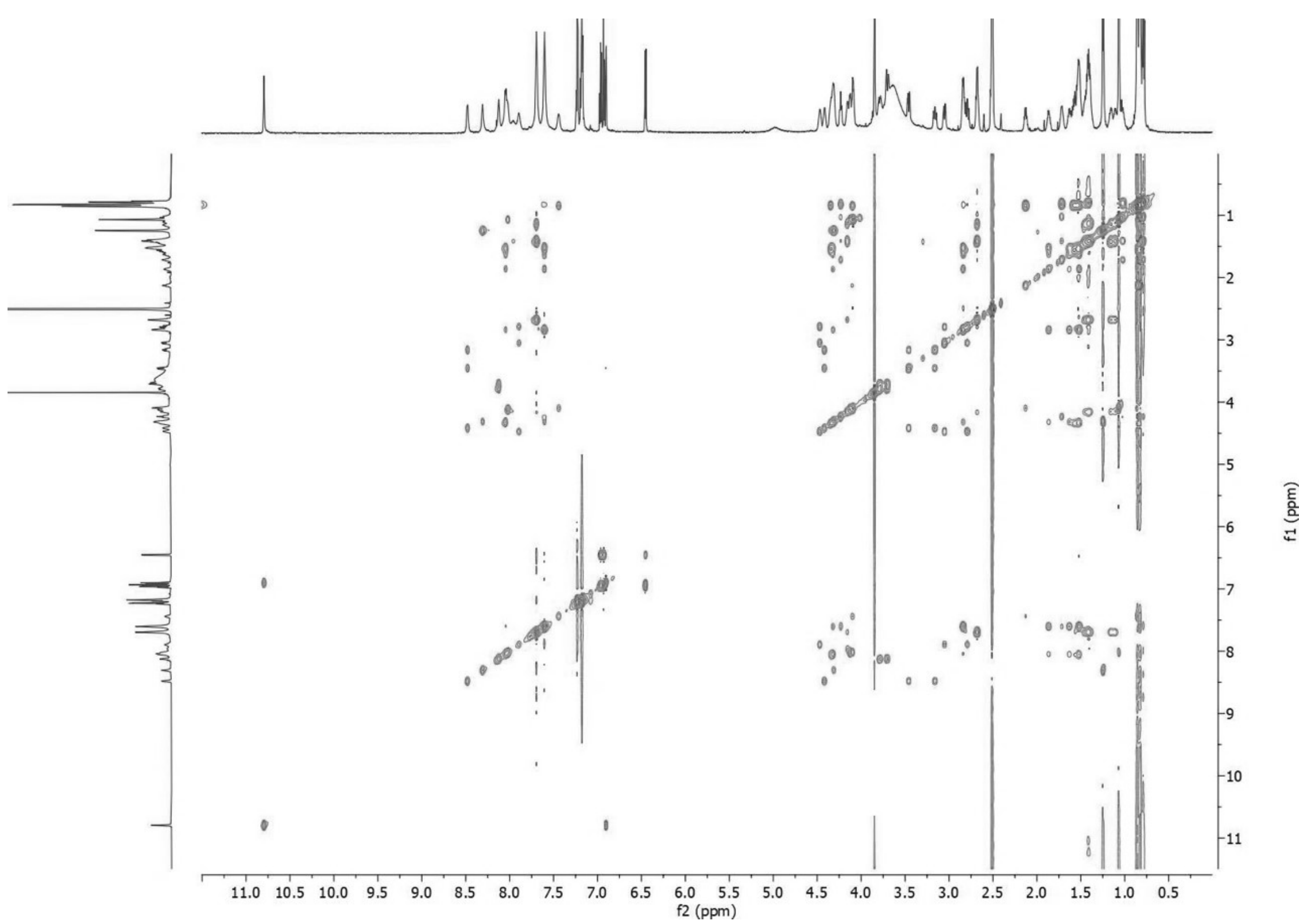

NMR (^{1}H,^{1}H-NOESY, 700 MHz, DMSO-d_6) of cortinarin C (2)

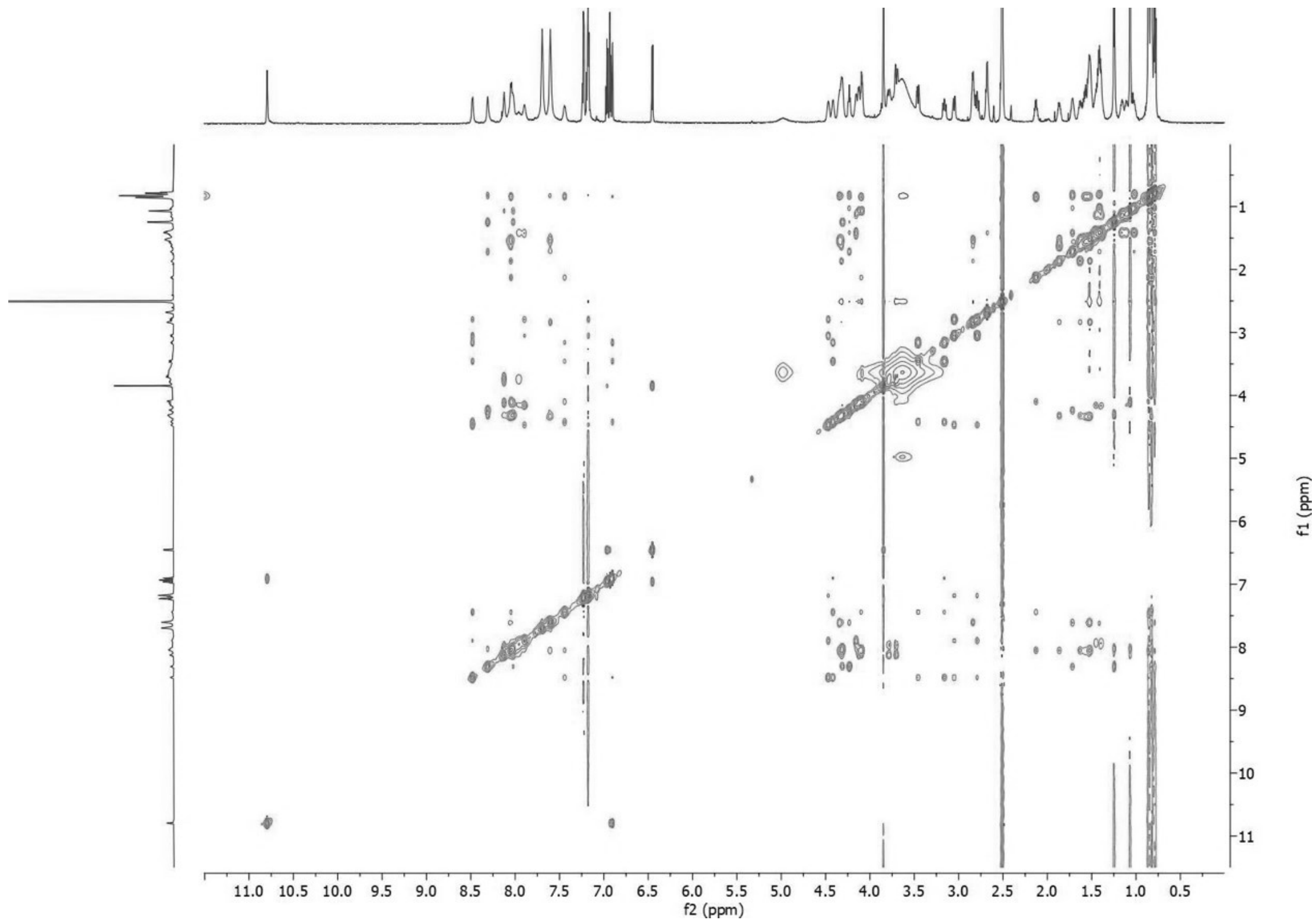

NMR (^1H, 500 MHz, DMSO-d_6) of CorX$_P$ (1c$_B$)

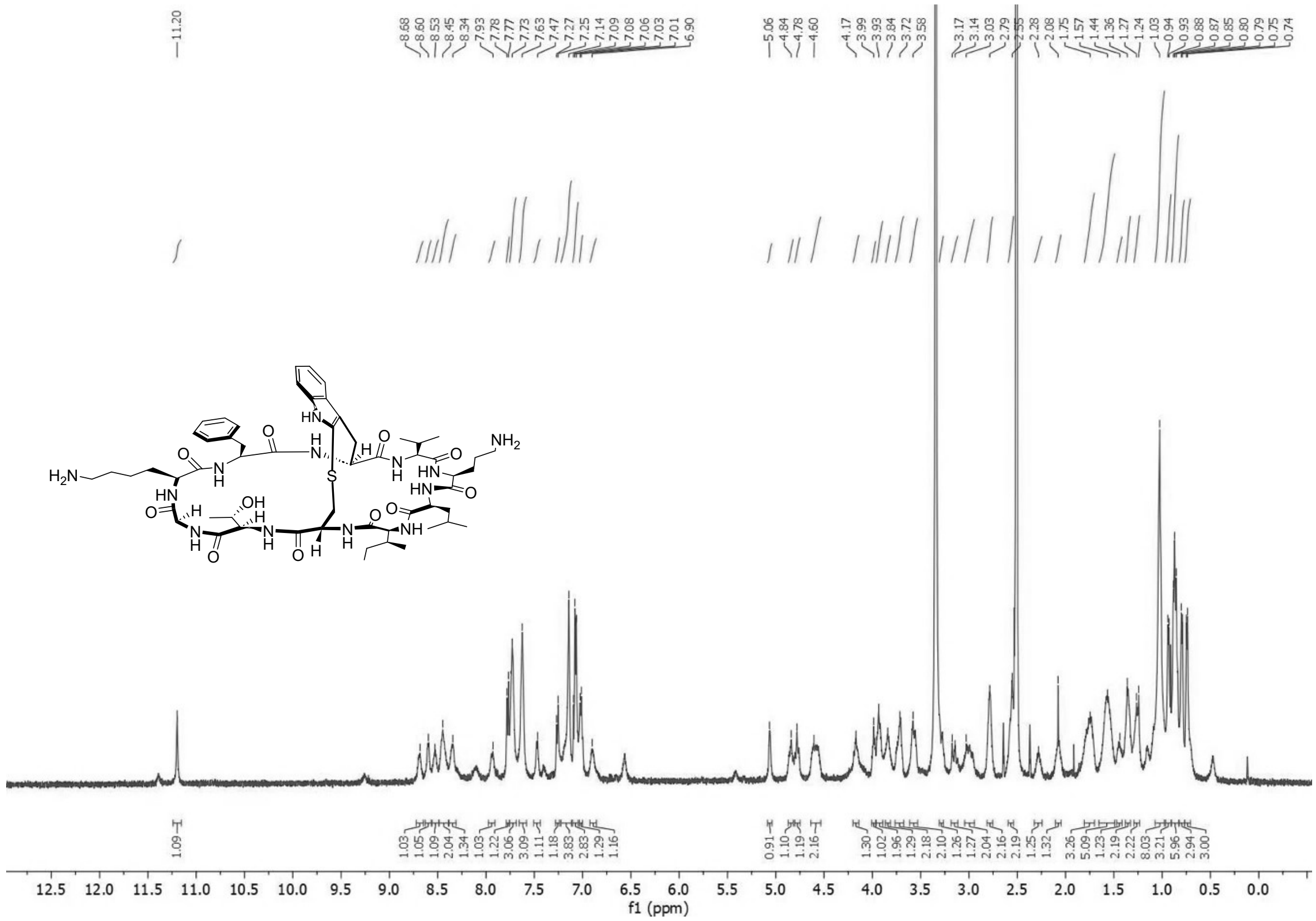

NMR (^1H,^{13}C-HQSC, 126 MHz, DMSO-d_6) of CorX$_P$ (1c$_B$)

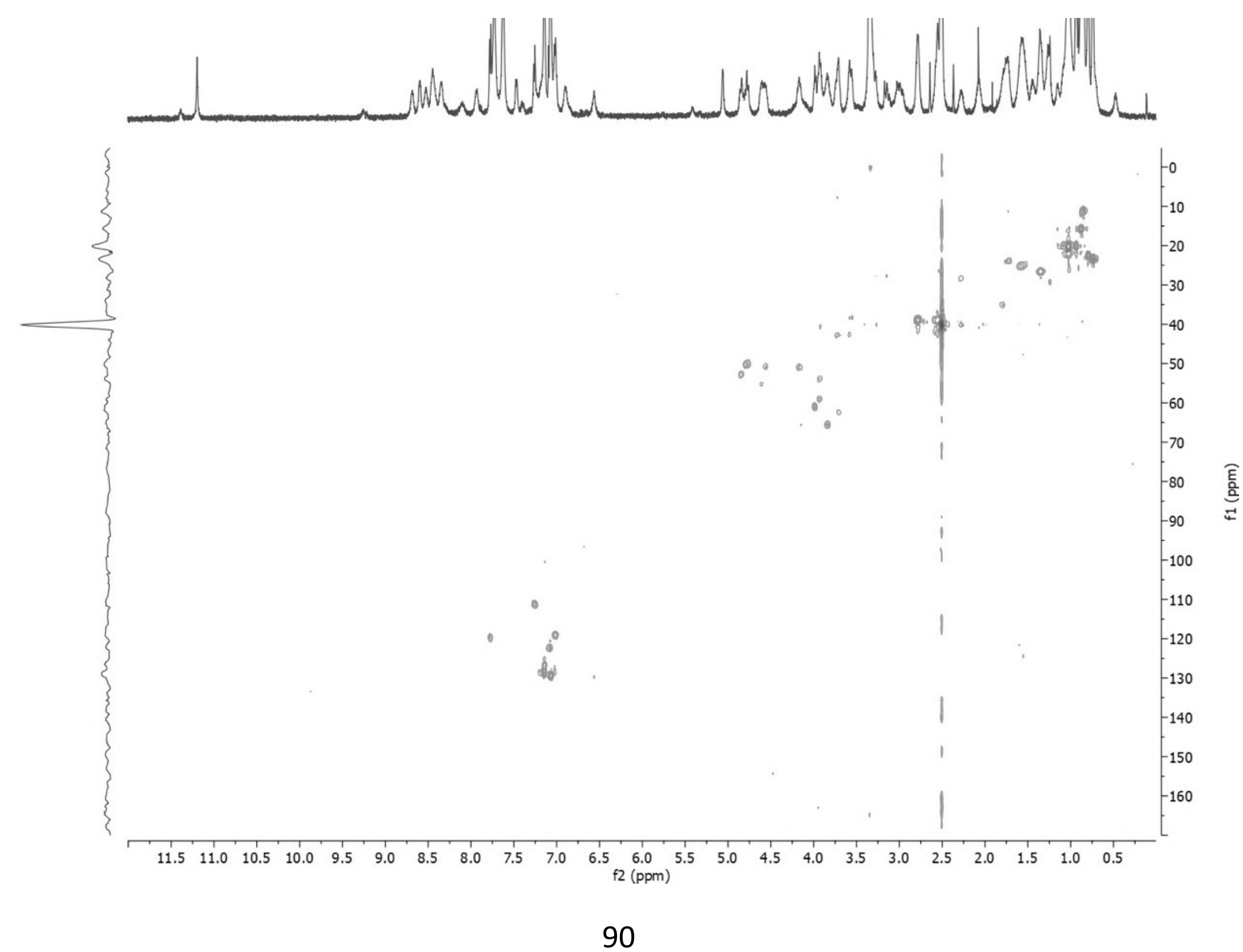

NMR (^1H,^1H-COSY, 500 MHz, DMSO-d_6) of CorX$_P$ (1c$_B$)

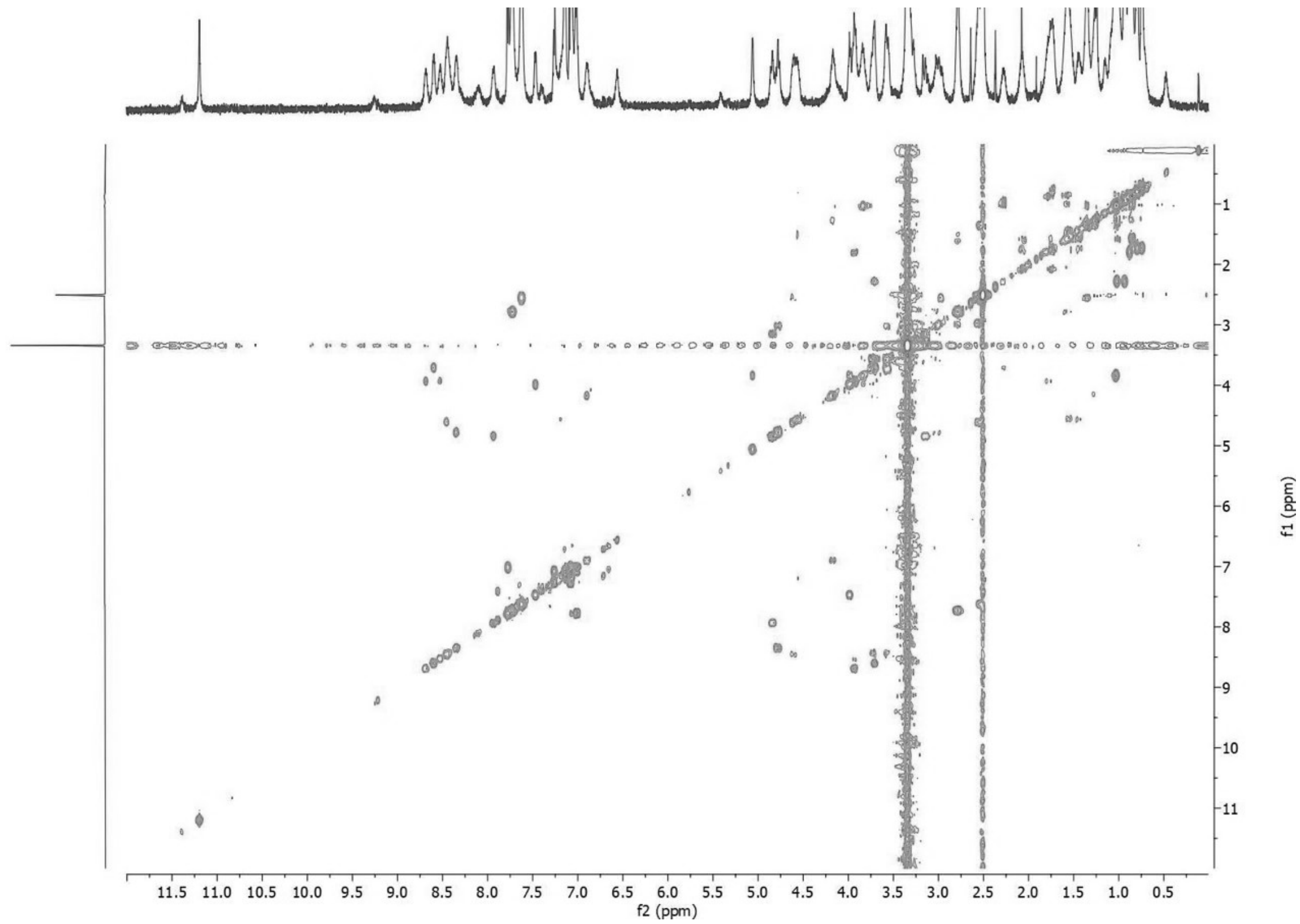

NMR (^1H,^1H-TOCSY, 500 MHz, DMSO-d_6) of CorX$_P$ (1c$_B$)

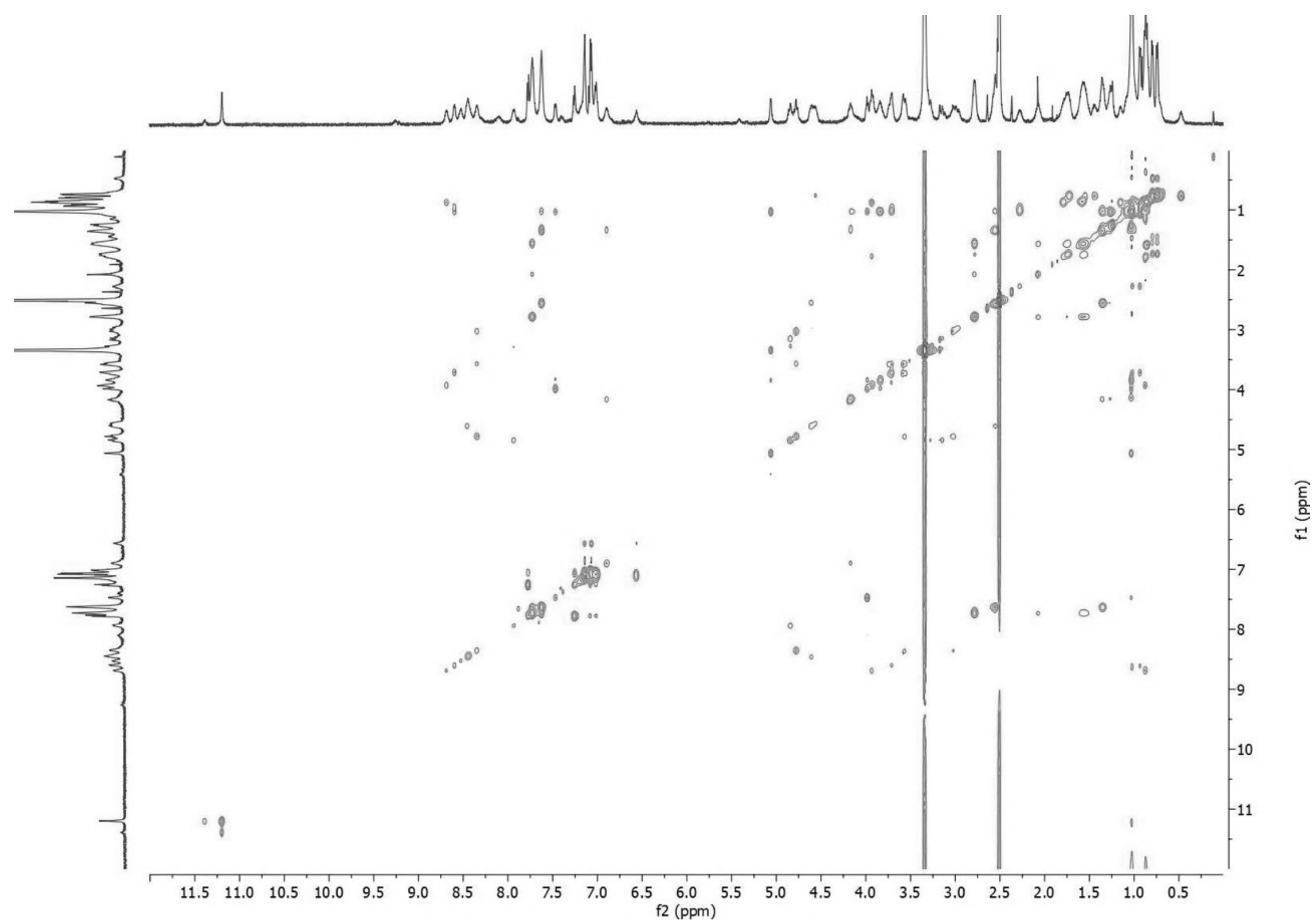

NMR (^{1}H,^{1}H-NOESY, 700 MHz, DMSO-d_6) of CorX$_P$ (1c$_B$)

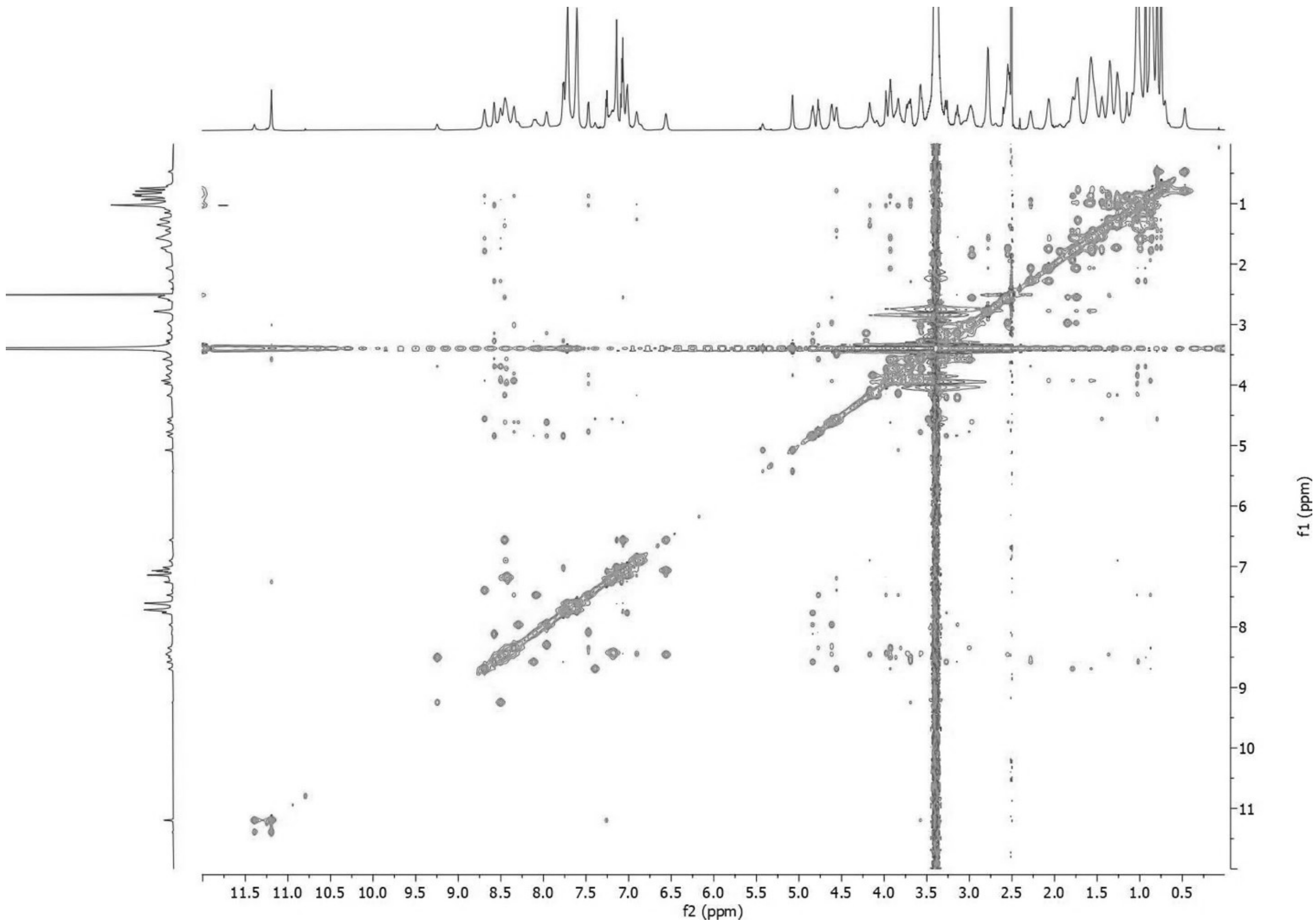

NMR (^{1}H, 500 MHz, DMSO-d_6) of CorX$_M$ (1c$_A$)

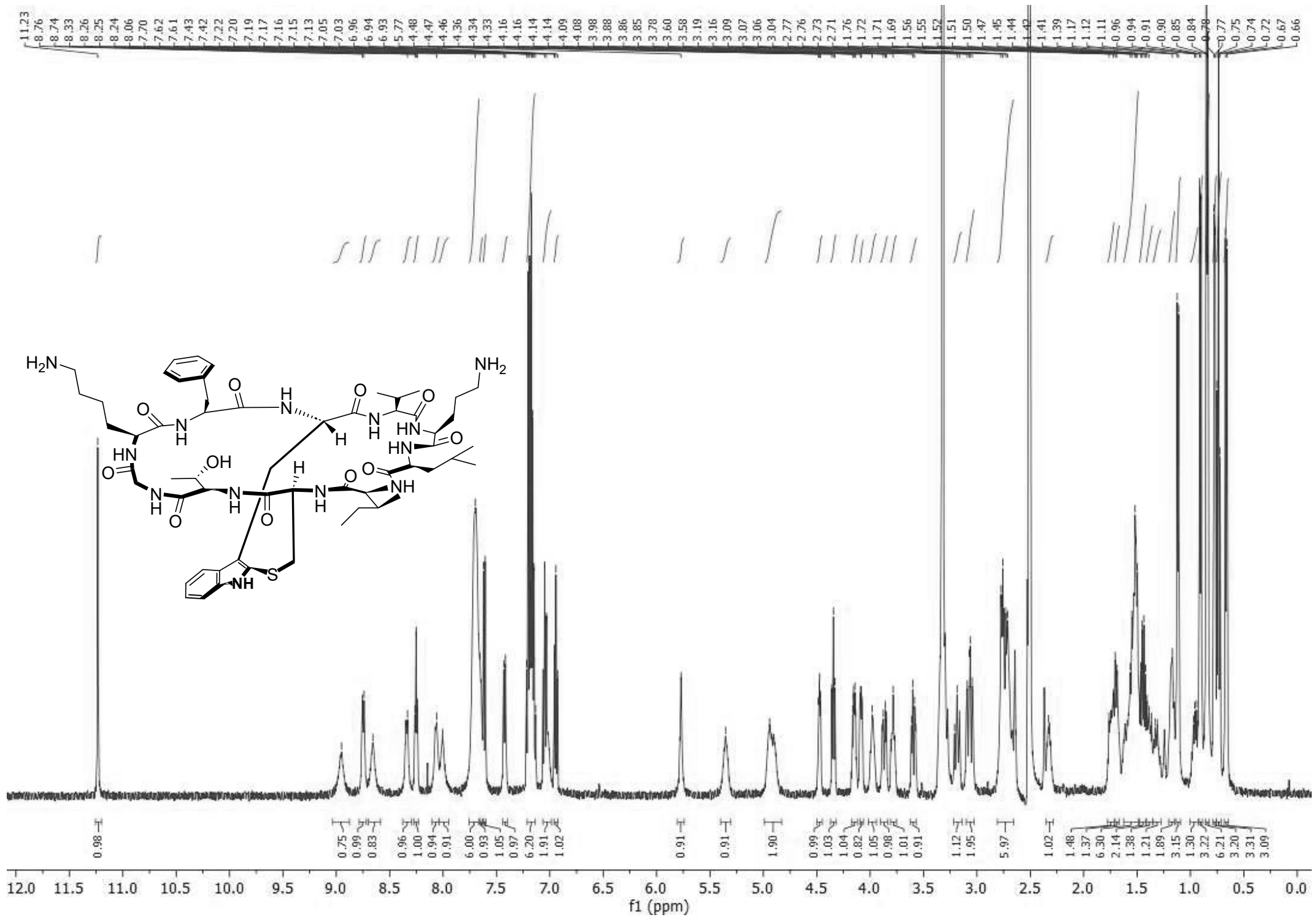

NMR (^{1}H,^{13}C-HQSC, 500 MHz, DMSO-d_6) of CorX$_M$ (1c$_A$)

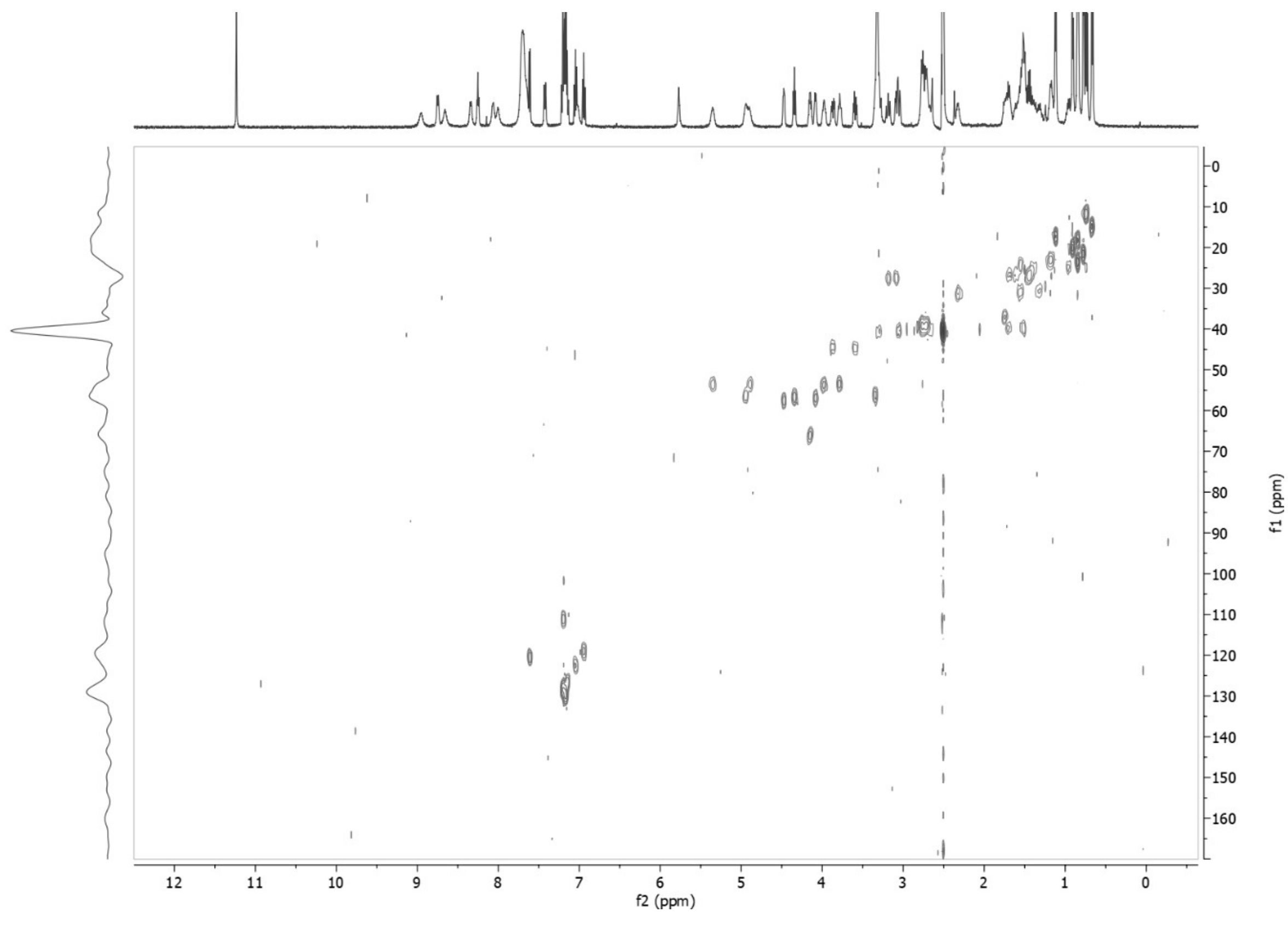

NMR (^{1}H,^{1}H-COSY, 500 MHz, DMSO-d_6) of CorX$_M$ (1c$_A$)

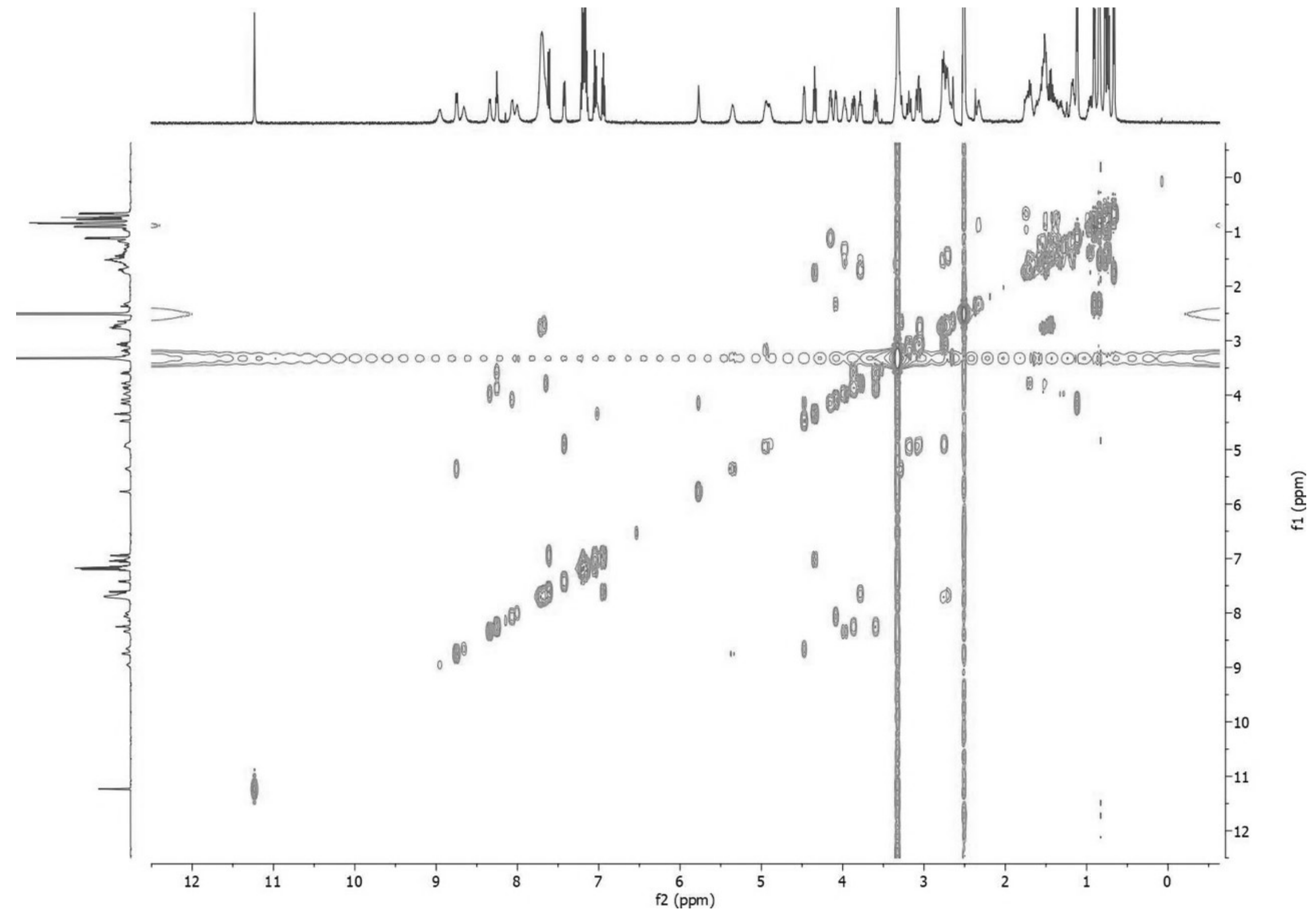

NMR (^{1}H,^{1}H-TOCSY, 500 MHz, DMSO-d_6) of CorX$_M$ (1c$_A$)

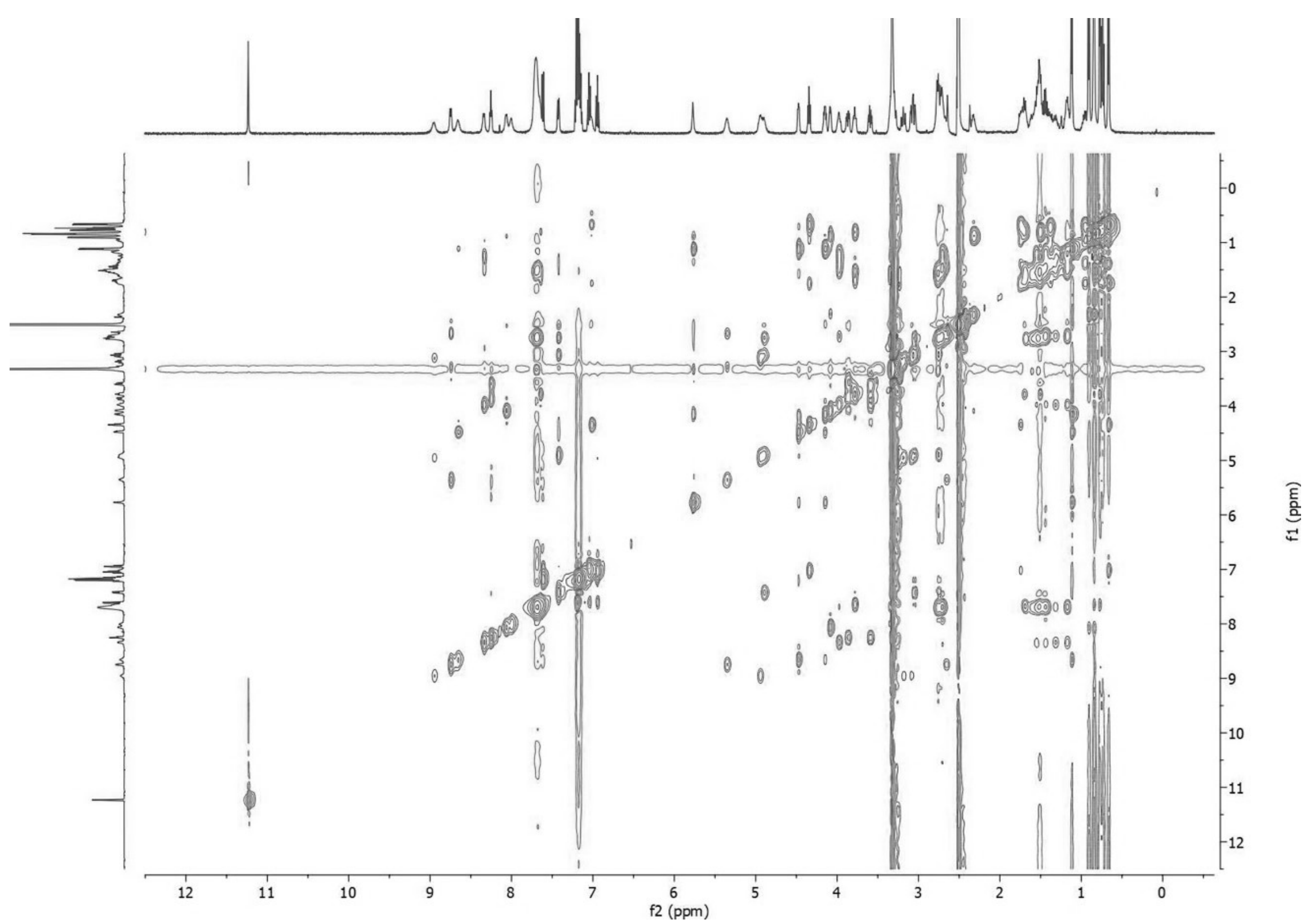

NMR (^1H,^1H-NOESY, 700 MHz, DMSO-d_6) of CorX$_M$ (1c$_A$)

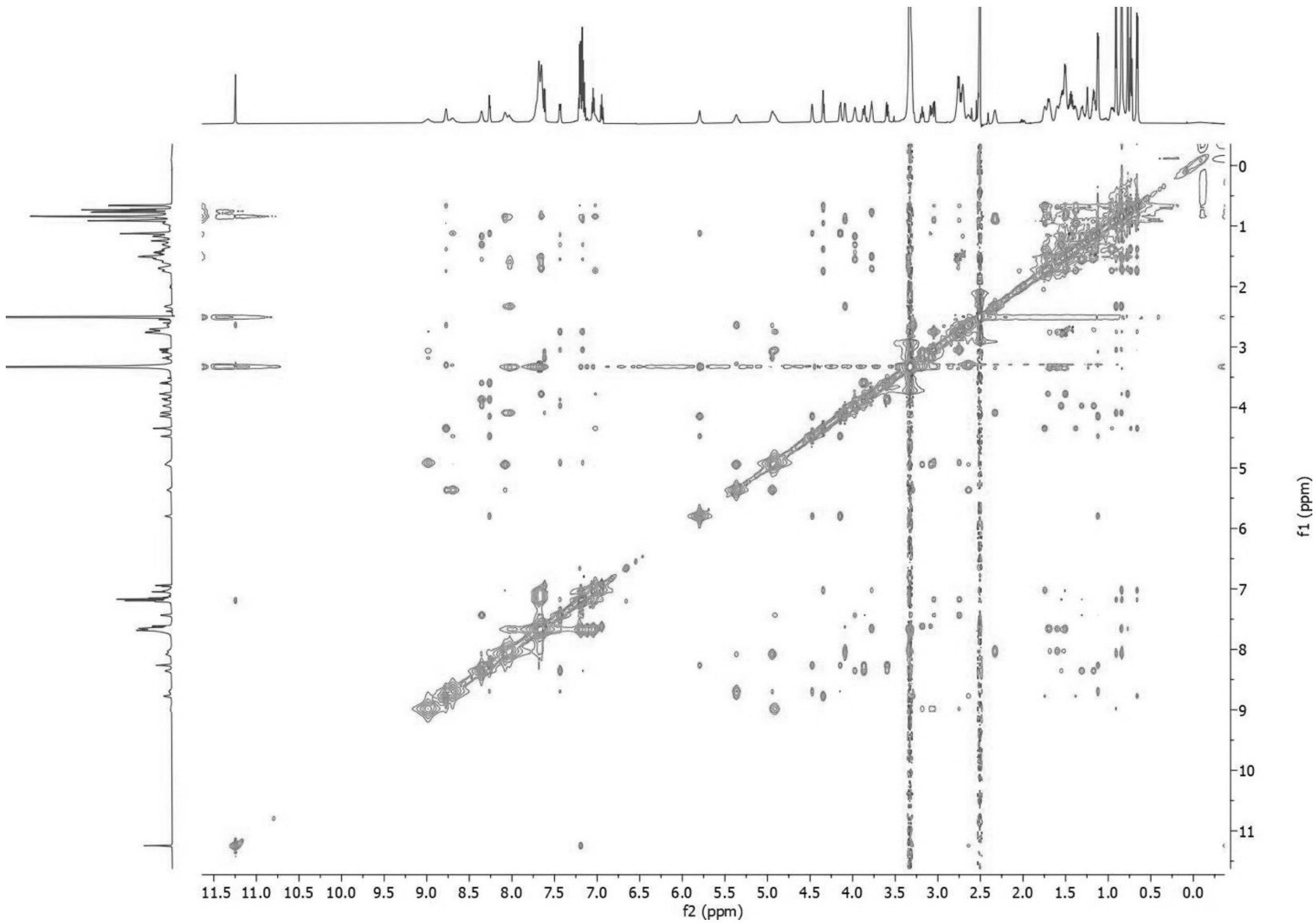

NMR (^{1}H 500 MHz, DMSO-d_6) of CorX-Ala$_P$(S20$_A$)

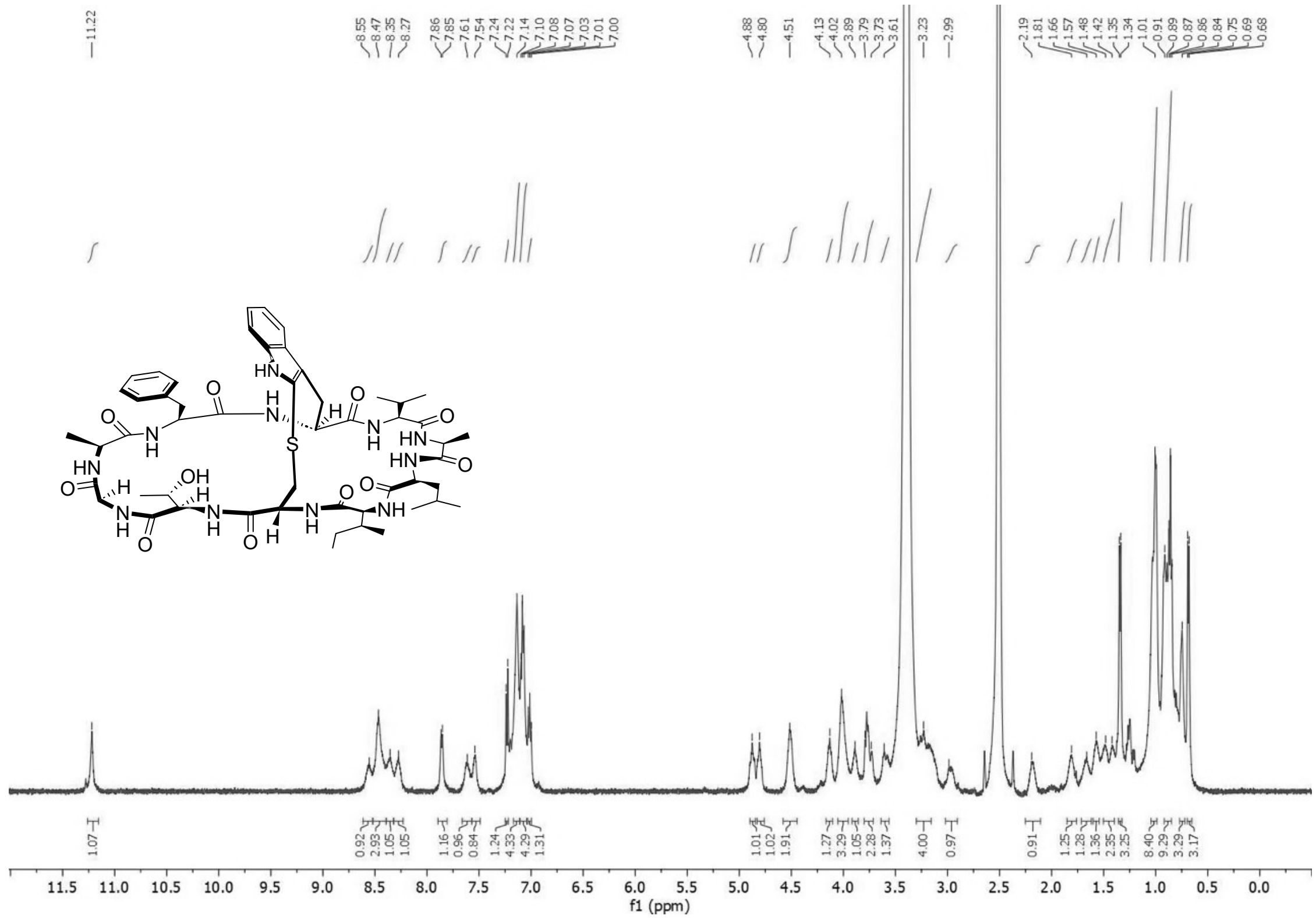

NMR (^{1}H,^{13}C-HQSC, DMSO-d_6) of CorX-Ala$_P$(S20$_A$)

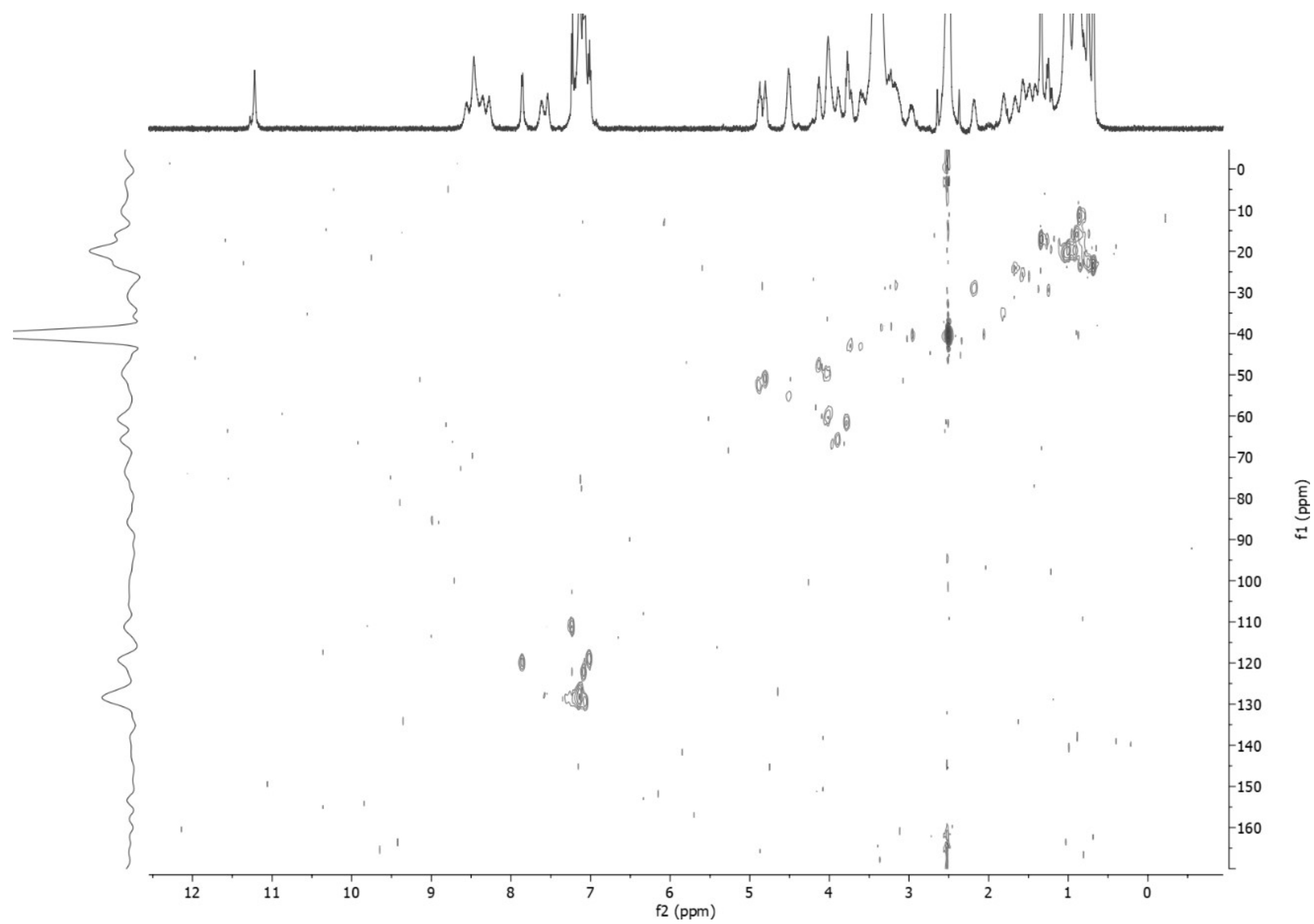

NMR (^{1}H,^{1}H-COSY, 500 MHz, DMSO-d_6) of CorX-Ala$_P$(S20$_A$)

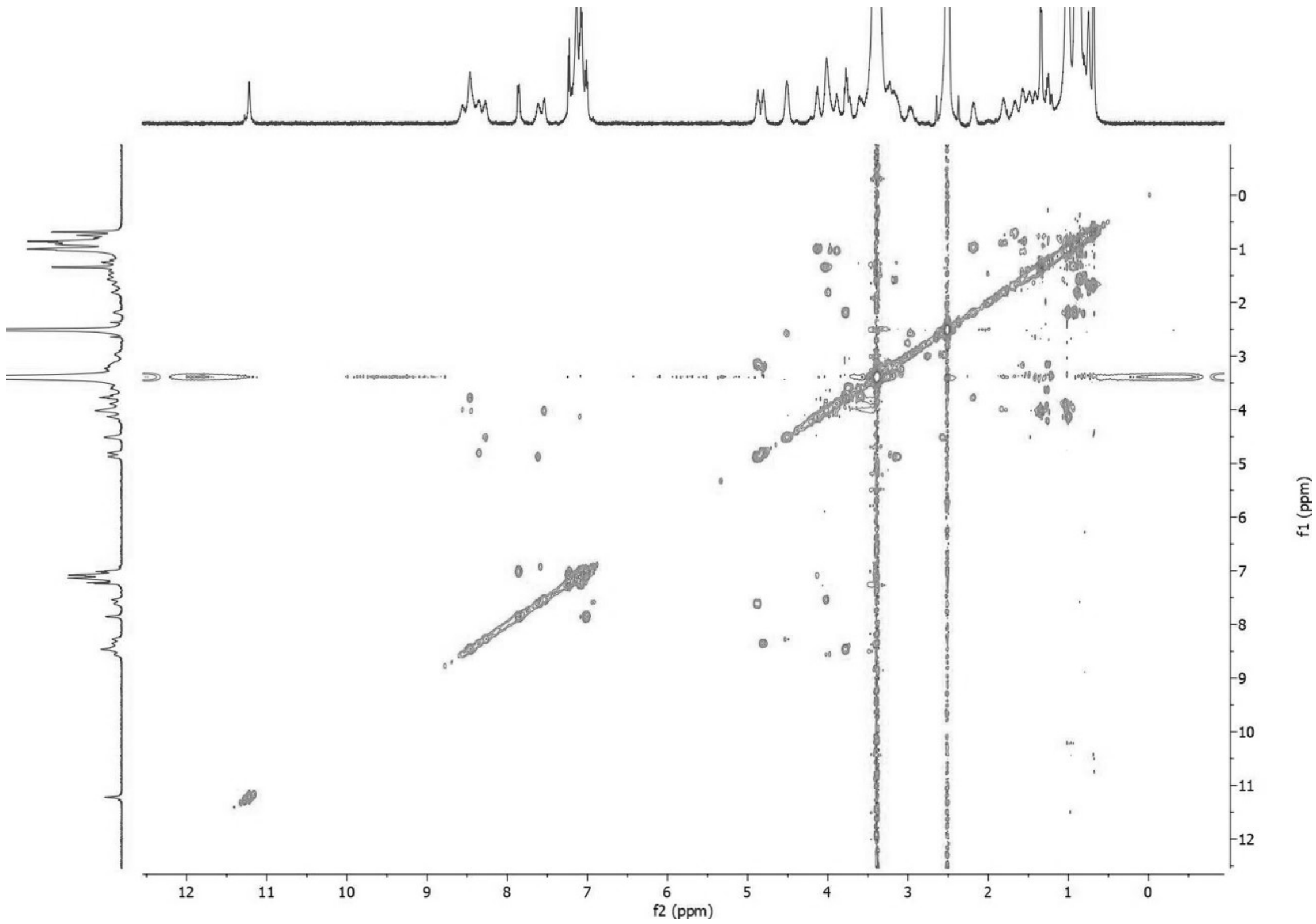

NMR (^{1}H,^{1}H-TOCSY, 500 MHz, DMSO-d_6) of CorX-Ala$_P$(S20$_A$)

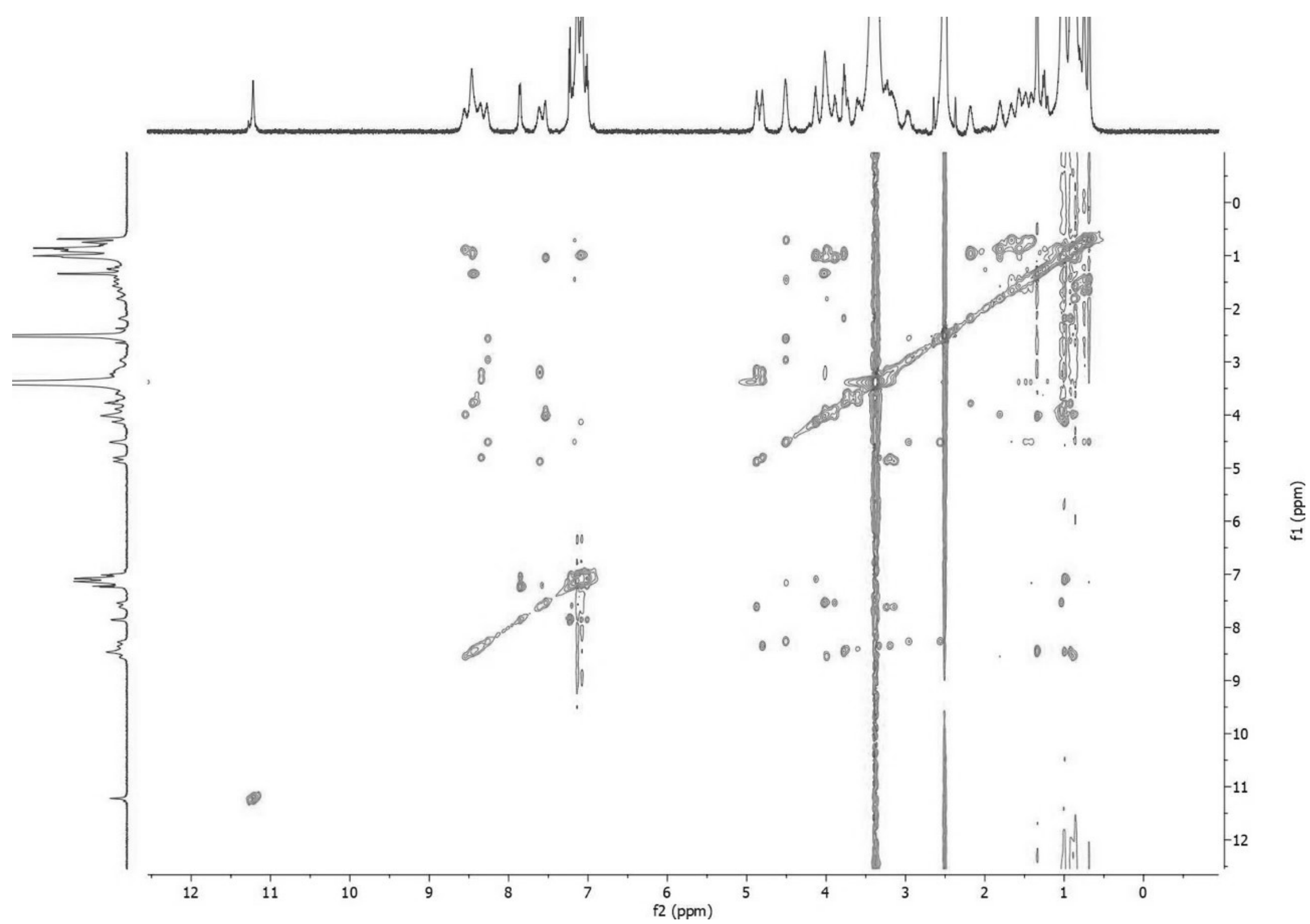

NMR (^1H,^1H-NOESY, 500 MHz, DMSO-d_6) of CorX-Ala$_P$(S20$_B$)

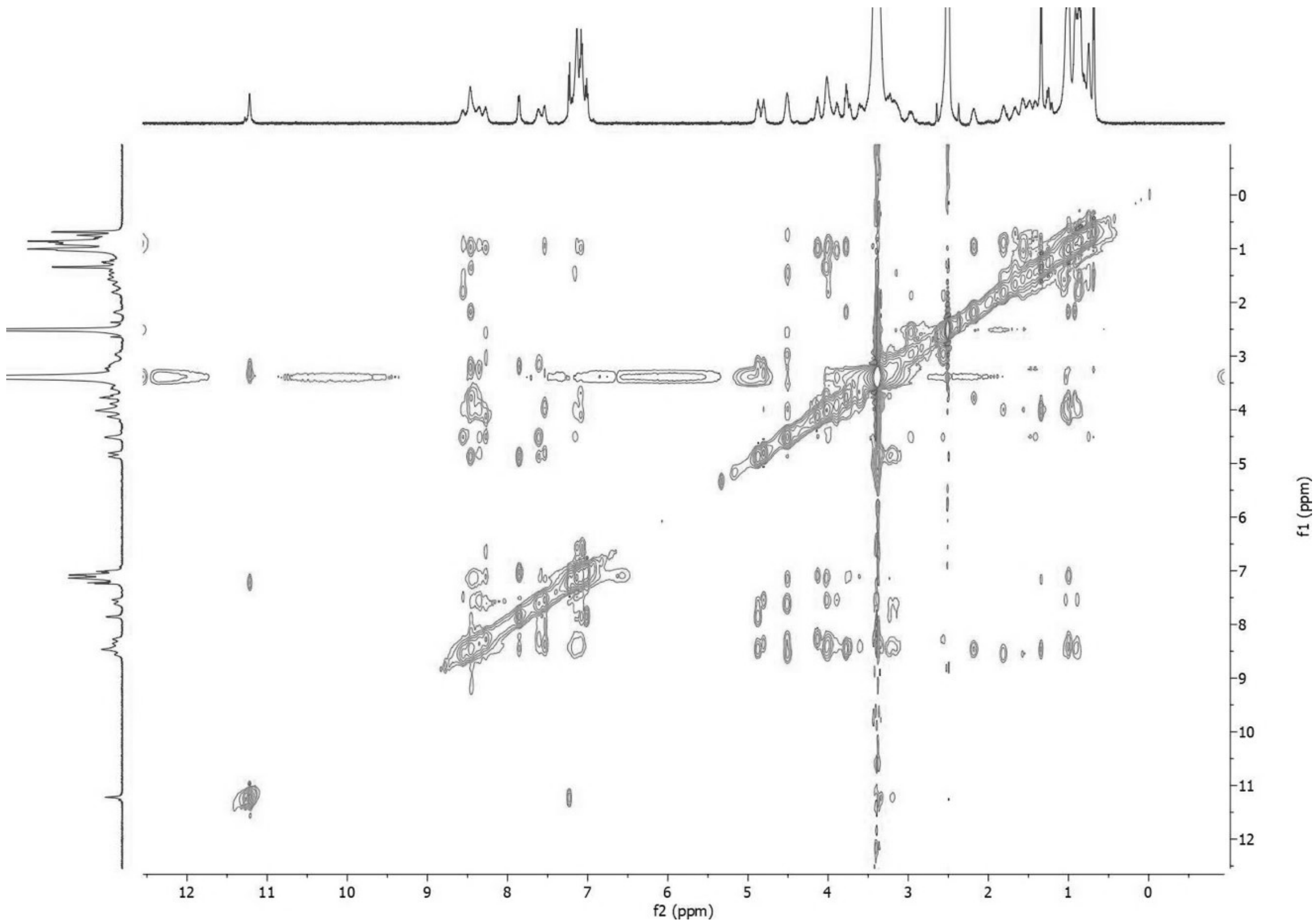

NMR (^1H, 700 MHz, DMSO-d_6) of CorX-Ala$_M$(S20$_B$)

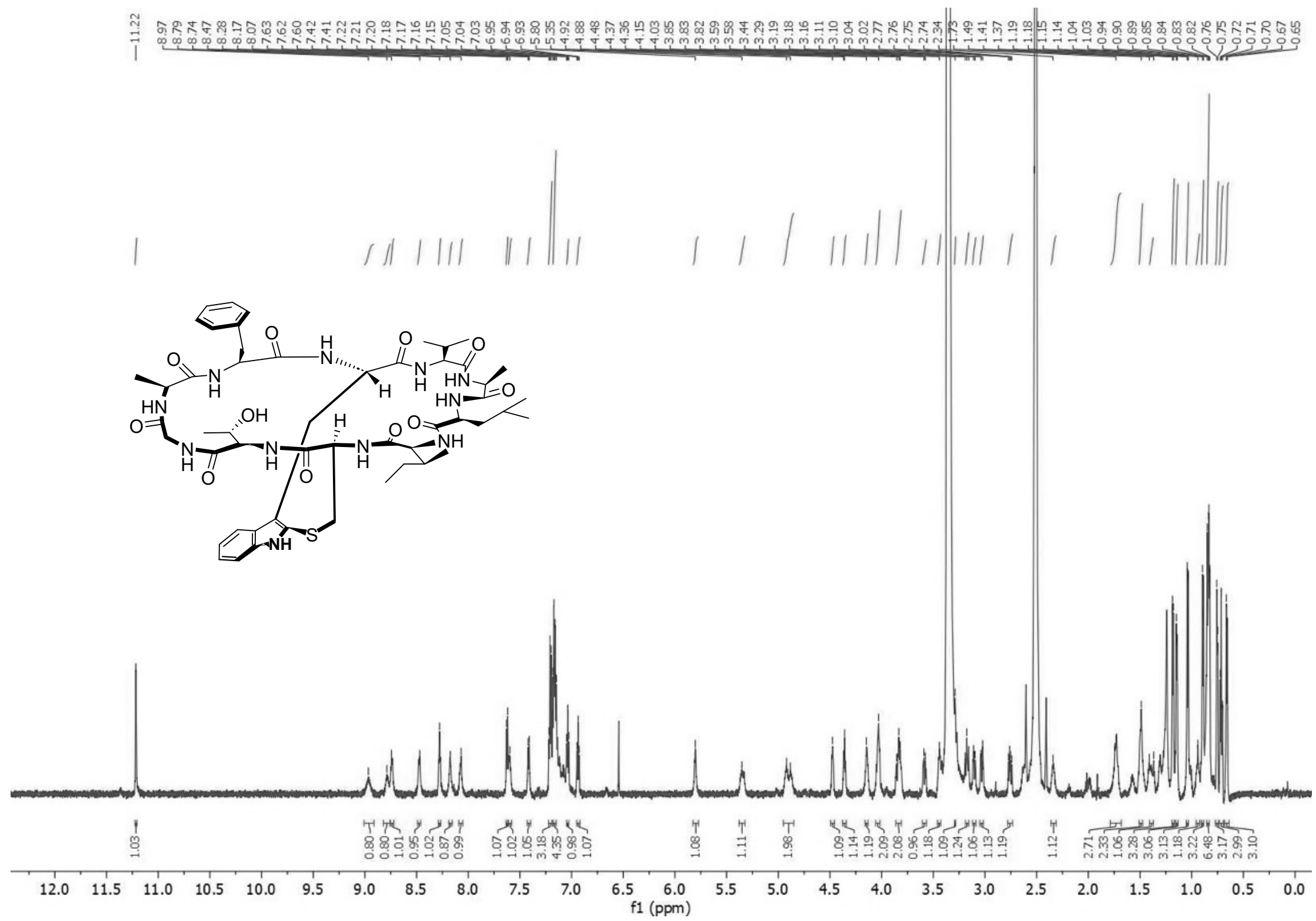

NMR (^1H,^{13}C-HQSC, 126 MHz, DMSO-d_6) of CorX-Ala$_M$(S20$_B$)

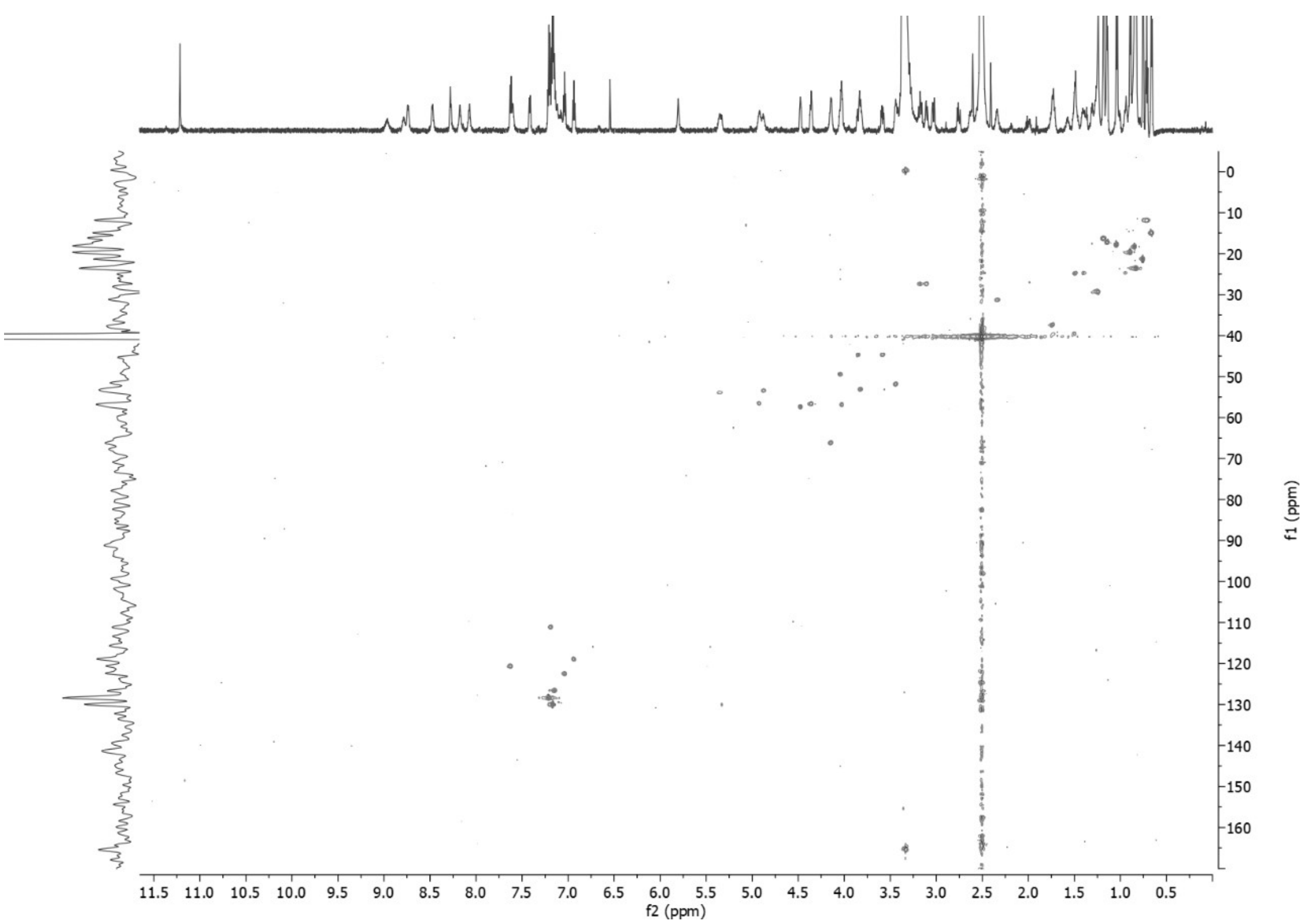

NMR (^{1}H,^{1}H-COSY, 700 MHz, DMSO-d_6) of CorX-Ala$_M$(S20$_B$)

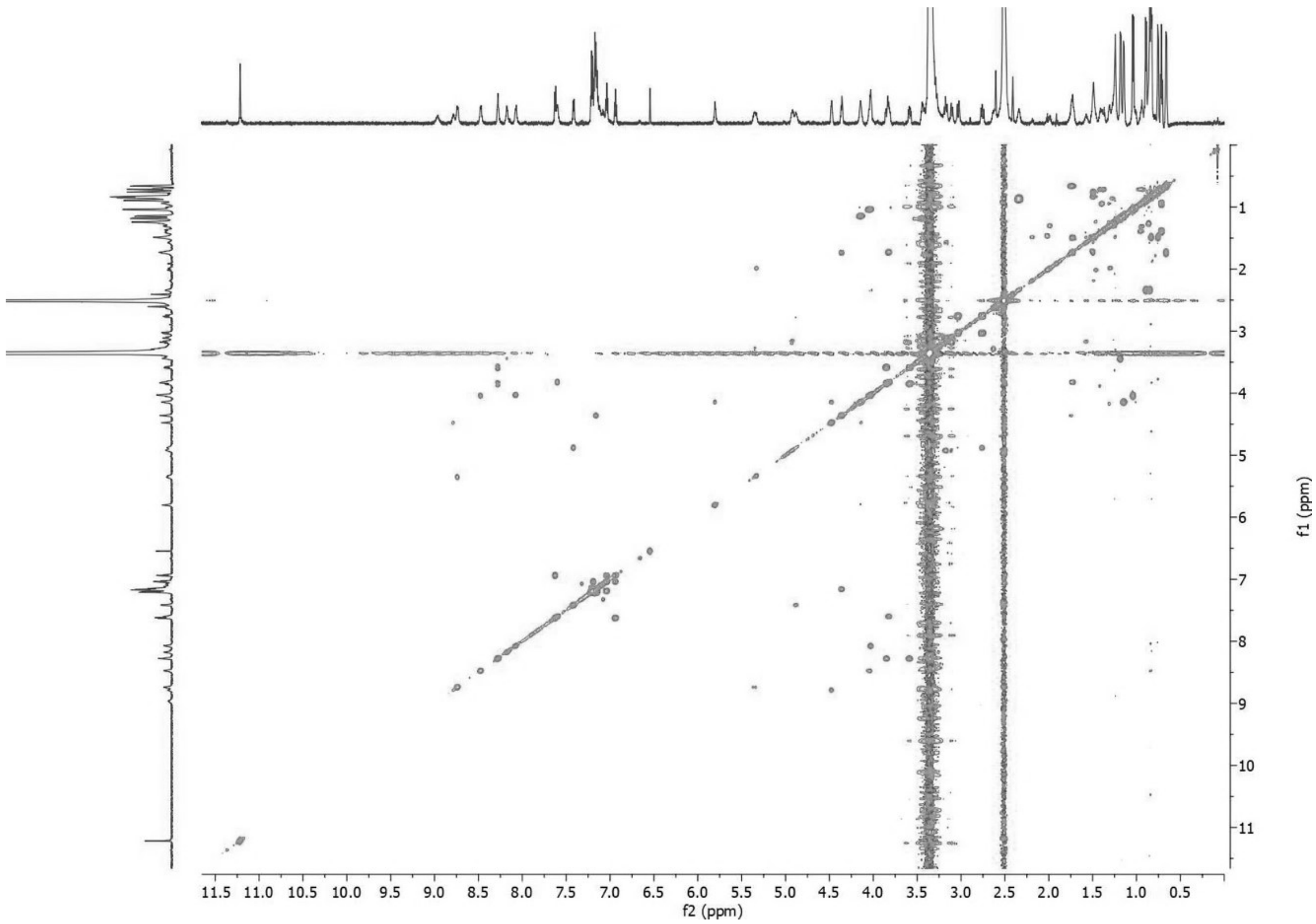

NMR (^{1}H,^{1}H-TOCSY, 700 MHz, DMSO-d_6) of CorX-Ala$_M$(S20$_B$)

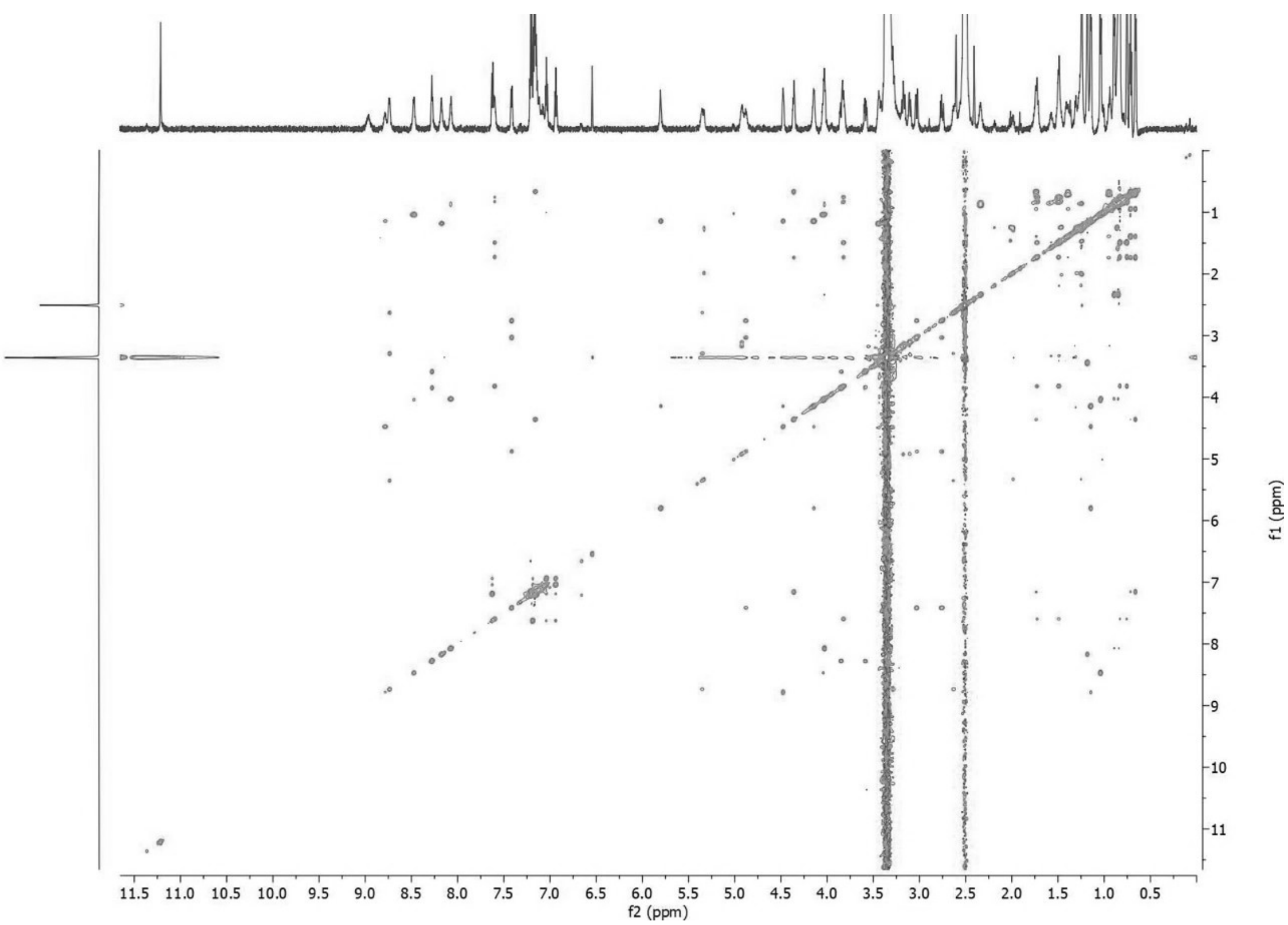

NMR (^{1}H,^{1}H-NOESY, 700 MHz, DMSO-d_6) of CorX-Ala$_M$(S20$_B$)

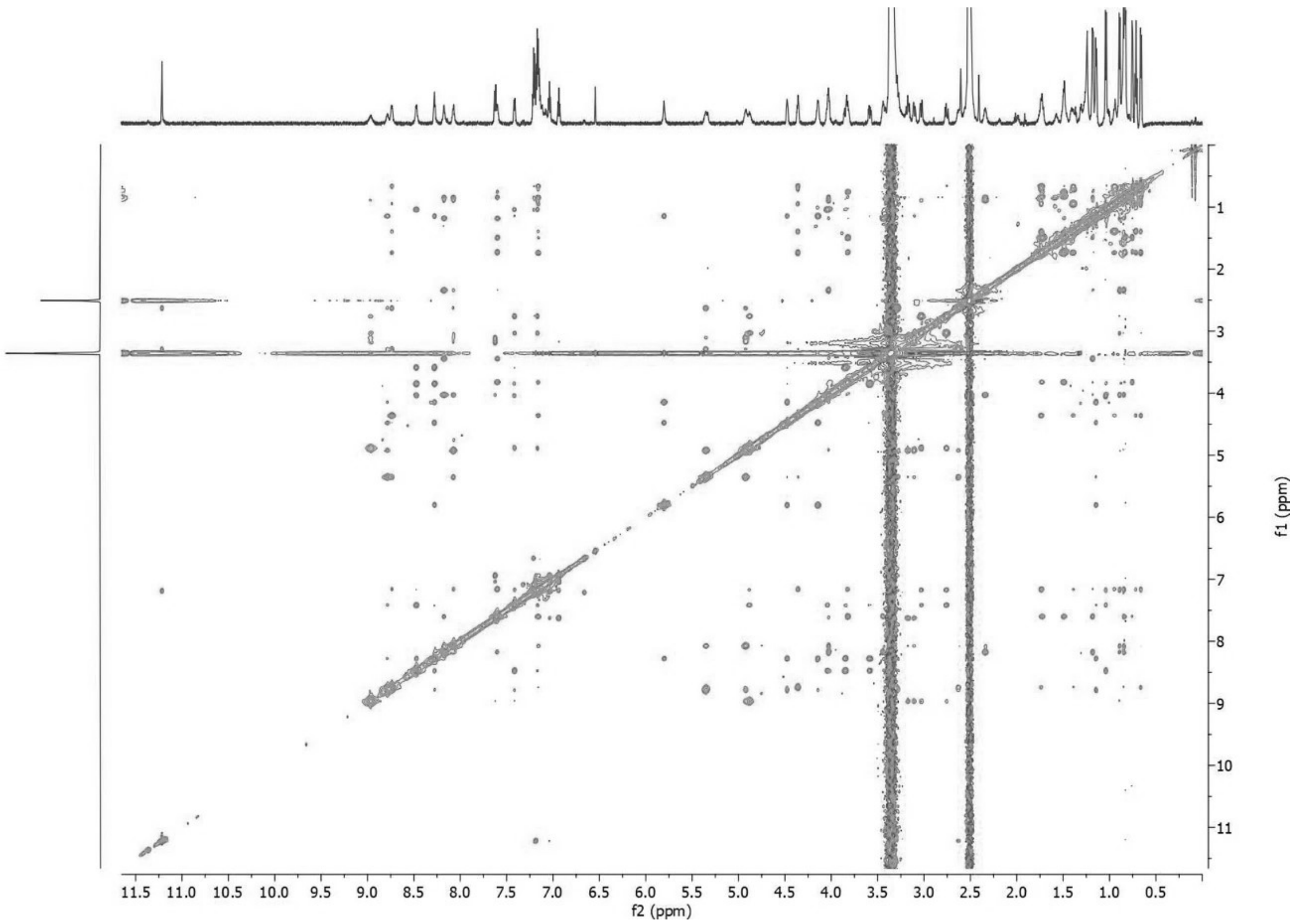

12 Reference

1 White, T. Bruns, S. Lee and J. Taylor, 1990, pp. 315–322.
2 C. Camacho, G. Coulouris, V. Avagyan, N. Ma, J. Papadopoulos, K. Bealer and T. L. Madden, *BMC Bioinformatics*, 2009, **10**, 421.
3 Z. He, P. Long, F. Fang, S. Li, P. Zhang and Z. Chen, *BMC Genomics*, 2020, **21**, 440.
4 G. Yao, C. H. Knittel, S. Kosol, M. T. Wenz, B. G. Keller, H. Gruß, A. C. Braun, C. Lutz, T. Hechler, A. Pahl and R. D. Süssmuth, *J. Am. Chem. Soc.*, 2021, **143**, 14322–14331.
5 K. Fujii, Y. Ikai, H. Oka, M. Suzuki and K. Harada, *Anal. Chem.*, 1997, **69**, 5146–5151.
6 K. Fujii, T. Shimoya, Y. Ikai, H. Oka and K. Harada, *Tetrahedron Lett.*, 1998, **39**, 2579–2582.
7 W. F. Vranken, W. Boucher, T. J. Stevens, R. H. Fogh, A. Pajon, M. Llinas, E. L. Ulrich, J. L. Markley, J. Ionides and E. D. Laue, *Proteins: Struct., Funct., Bioinf.*, 2005, **59**, 687–696.
8 E. M. Yilmaz and P. Güntert, *J. Biomol. NMR*, 2015, **63**, 21–37.
9 A. K. Malde, L. Zuo, M. Breeze, M. Stroet, D. Poger, P. C. Nair, C. Oostenbrink and A. E. Mark, *J. Chem. Theory Comput.*, 2011, **7**, 4026–4037.
10 S. Kuschert, M. Stroet, Y. K.-Y. Chin, A. C. Conibear, X. Jia, T. Lee, C. R. O. Bartling, K. Strømgaard, P. Güntert, K. J. Rosengren, A. E. Mark and M. Mobli, *Magn. Reson.*, 2023, **4**, 57–72.
11 P. Güntert and L. Buchner, *J. Biomol. NMR*, 2015, **62**, 453–471.
12 E. N. Baker and R. E. Hubbard, *Prog. Biophys. Mol. Biol.*, 1984, **44**, 97–179.
13 M. D. Hanwell, D. E. Curtis, D. C. Lonie, T. Vandermeersch, E. Zurek and G. R. Hutchison, *J. Cheminform.*, 2012, **4**, 17.
14 A. W. Sousa da Silva and W. F. Vranken, *BMC Res. Notes*, 2012, **5**, 367.
15 X. He, V. H. Man, W. Yang, T.-S. Lee and J. Wang, *J. Chem. Phys.*, 2020, **153**, 114502.
16 T. J. Macke, W. Svrcek-Seiler and R. A. Brown, *Amber 11 Users' Manual*, 2010.
17 G. Bussi, D. Donadio and M. Parrinello, *J. Chem. Phys.*, 2007, **126**, 014101.
18 M. Bernetti and G. Bussi, *J. Chem. Phys.*, 2020, **153**, 114107.
19 S. Páll, M. J. Abraham, C. Kutzner, B. Hess and E. Lindahl, in *Solving Software Challenges for Exascale*, eds. S. Markidis and E. Laure, Springer International Publishing, Cham, 2015, pp. 3–27.
20 M. J. Abraham, T. Murtola, R. Schulz, S. Páll, J. C. Smith, B. Hess and E. Lindahl, *SoftwareX*, 2015, **1–2**, 19–25.
21 S. Pronk, S. Páll, R. Schulz, P. Larsson, P. Bjelkmar, R. Apostolov, M. R. Shirts, J. C. Smith, P. M. Kasson, D. van der Spoel, B. Hess and E. Lindahl, *Bioinformatics*, 2013, **29**, 845–854.
22 A. Shrake and J. A. Rupley, *J. Mol. Biol.*, 1973, **79**, 351–371.
23 S. Baltzer, T. Bulatov, C. Schmied, A. Krämer, B.-T. Berger, A. Oder, R. Walker-Gray, C. Kuschke, K. Zühlke, J. Eichhorst, M. Lehmann, S. Knapp, J. Weston, J. P. Von Kries, R. D. Süssmuth and E. Klussmann, *IJMS*, 2022, **23**, 763.
24 D. Faust, A. Geelhaar, B. Eisermann, J. Eichhorst, B. Wiesner, W. Rosenthal and E. Klussmann, *JoVE*, 2013, e50366.
25 A. Vossenkämper, P. I. Nedvetsky, B. Wiesner, J. Furkert, W. Rosenthal and E. Klussmann, *Am. J. Physiol. Cell Physiol.*, 2007, **293**, C1129–C1138.
26 K. Maric, B. Wiesner, D. Lorenz, E. Klussmann, T. Betz and W. Rosenthal, *Biophysical Journal*, 2001, **80**, 1783–1790.
27 T. Vukićević, C. Hinze, S. Baltzer, N. Himmerkus, C. Quintanova, K. Zühlke, F. Compton, R. Ahlborn, A. Dema, J. Eichhorst, B. Wiesner, M. Bleich, K. M. Schmidt-Ott and E. Klussmann, *JASN*, 2019, **30**, 795–810.
28 A. Dema, D. Faust, K. Lazarow, M. Wippich, M. Neuenschwander, K. Zühlke, A. Geelhaar, T. Pallien, E. Hallscheidt, J. Eichhorst, B. Wiesner, H. Černecká, O. Popp, P. Mertins, G. Dittmar, J. P. von Kries and E. Klussmann, *Cells*, 2020, **9**, 673.
29 C. Janssen, G. Nadar, T. Soykan, M. Lehmann and E. Klussmann, *Zenodo*, DOI:10.5281/zenodo.14918442.

13 Appendix

Table S 34. The SMILES code for cortinarins derivatives

Structure	SMILES
CorA	O=C(N[C@@H](CC1=CC=CC=C1)C(N[C@@H](CC2=C(NC3=CC=CC(OC)=C32)SC[C@@H](C(N[C@H]([C@@H](O)C)C(NC4)=O)=O)NC5=O)C(N[C@@H](C(C)C)C(N[C@@H](CCCN)C(N[C@@H](CC(C)C)C(N[C@H]5[C@@H](C)CC)=O)=O)=O)=O)=O)[C@H](CCCCN)NC4=O
CorB	O=C(N[C@@H](CC1=CC=CC=C1)C(N[C@@H](CC2=C(NC3=CC=CC(O)=C32)SC[C@@H](C(N[C@H]([C@@H](O)C)C(NC4)=O)=O)NC5=O)C(N[C@@H](C(C)C)C(N[C@@H](CCCN)C(N[C@@H](CC(C)C)C(N[C@H]5[C@@H](C)CC)=O)=O)=O)=O)=O)[C@H](CCCCN)NC4=O
CorC	O=C(N[C@@H](CC1=CC=CC=C1)C(N[C@@H](CC2=CNC3=CC=CC(OC)=C23)C(N[C@@H](C(C)C)C(N[C@@H](CCCN)C(N[C@@H](CC(C)C)C(N[C@@H]([C@@H](C)CC)C(N[C@@H](C)C(N[C@H]([C@@H](O)C)C(NC4)=O)=O)=O)=O)=O)=O)=O)=O)[C@H](CCCCN)NC4=O
CorX$_{P}$	O=C(N[C@@H](CC1=CC=CC=C1)C(N[C@@H](CC2=C(NC3=CC=CC=C32)SC[C@@H](C(N[C@H]([C@@H](O)C)C(NC4)=O)=O)NC5=O)C(N[C@@H](C(C)C)C(N[C@@H](CCCN)C(N[C@@H](CC(C)C)C(N[C@H]5[C@@H](C)CC)=O)=O)=O)=O)=O)[C@H](CCCCN)NC4=O
CorX$_{M}$	O=C(N[C@@H](CC1=CC=CC=C1)C(N[C@@H](CC2=C(NC3=CC=CC=C32)SC[C@@H](C(N[C@H]([C@@H](O)C)C(NC4)=O)=O)NC5=O)C(N[C@@H](C(C)C)C(N[C@@H](CCCN)C(N[C@@H](CC(C)C)C(N[C@H]5[C@@H](C)CC)=O)=O)=O)=O)=O)[C@H](CCCCN)NC4=O
CorX-Ala$_{P}$	O=C(N[C@@H](CC1=CC=CC=C1)C(N[C@@H](CC2=C(NC3=CC=CC=C32)SC[C@@H](C(N[C@H]([C@@H](O)C)C(NC4)=O)=O)NC5=O)C(N[C@@H](C(C)C)C(N[C@@H](C)C(N[C@@H](CC(C)C)C(N[C@H]5[C@@H](C)CC)=O)=O)=O)=O)=O)[C@H](C)NC4=O
CorX-Ala$_{M}$	O=C(N[C@@H](CC1=CC=CC=C1)C(N[C@@H](CC2=C(NC3=CC=CC=C32)SC[C@@H](C(N[C@H]([C@@H](O)C)C(NC4)=O)=O)NC5=O)C(N[C@@H](C(C)C)C(N[C@@H](C)C(N[C@@H](CC(C)C)C(N[C@H]5[C@@H](C)CC)=O)=O)=O)=O)=O)[C@H](C)NC4=O
